# iDNA-Prot|dis: Identifying DNA-Binding Proteins by Incorporating Amino Acid Distance-Pairs and Reduced Alphabet Profile into the General Pseudo Amino Acid Composition

**DOI:** 10.1371/journal.pone.0106691

**Published:** 2014-09-03

**Authors:** Bin Liu, Jinghao Xu, Xun Lan, Ruifeng Xu, Jiyun Zhou, Xiaolong Wang, Kuo-Chen Chou

**Affiliations:** 1 School of Computer Science and Technology, Harbin Institute of Technology Shenzhen Graduate School, Shenzhen, Guangdong, China; 2 Key Laboratory of Network Oriented Intelligent Computation, Harbin Institute of Technology Shenzhen Graduate School, Shenzhen, Guangdong, China; 3 Shanghai Key Laboratory of Intelligent Information Processing, Shanghai, China; 4 Gordon Life Science Institute, Belmont, Massachusetts, United States of America; 5 Stanford University, Stanford, California, United States of America; 6 Center of Excellence in Genomic Medicine Research (CEGMR), King Abdulaziz University, Jeddah, Saudi Arabia; University of Michigan, United States of America

## Abstract

Playing crucial roles in various cellular processes, such as recognition of specific nucleotide sequences, regulation of transcription, and regulation of gene expression, DNA-binding proteins are essential ingredients for both eukaryotic and prokaryotic proteomes. With the avalanche of protein sequences generated in the postgenomic age, it is a critical challenge to develop automated methods for accurate and rapidly identifying DNA-binding proteins based on their sequence information alone. Here, a novel predictor, called “iDNA-Prot|dis”, was established by incorporating the amino acid distance-pair coupling information and the amino acid reduced alphabet profile into the general pseudo amino acid composition (PseAAC) vector. The former can capture the characteristics of DNA-binding proteins so as to enhance its prediction quality, while the latter can reduce the dimension of PseAAC vector so as to speed up its prediction process. It was observed by the rigorous jackknife and independent dataset tests that the new predictor outperformed the existing predictors for the same purpose. As a user-friendly web-server, iDNA-Prot|dis is accessible to the public at http://bioinformatics.hitsz.edu.cn/iDNA-Prot_dis/. Moreover, for the convenience of the vast majority of experimental scientists, a step-by-step protocol guide is provided on how to use the web-server to get their desired results without the need to follow the complicated mathematic equations that are presented in this paper just for the integrity of its developing process. It is anticipated that the iDNA-Prot|dis predictor may become a useful high throughput tool for large-scale analysis of DNA-binding proteins, or at the very least, play a complementary role to the existing predictors in this regard.

## Introduction

DNA-binding proteins are essential ingredients for both eukaryotic and prokaryotic proteomes. They can interact with DNA, and play crucial role in various cellular processes (see, e.g., [1]), performing variety functions, such as transcriptional regulation.

In the early days, the identification of DNA binding proteins was carried out by experimental techniques, including filter binding assays, genetic analysis, chromatin immune precipitation on microarrays, and X-ray crystallography. However, it is both time-consuming and expensive to identify DNA-binding proteins purely based on biochemical experiments alone. Particularly, with the avalanche of biological sequences generated in the postgenomic age, it is highly desired to develop computational methods for fast and effective identifying DNA-binding proteins.

Actually, a few computational methods have been proposed in this regard. They can be roughly categorized into two types of approaches: (i) the structure-based method, and (ii) the sequence-based method. The 1^st^ type is actually using both the structural of proteins and their sequences information for identifying the DNA-binding proteins (see, e.g., [2]–[5]). Although these methods did indeed play an important role in stimulating the development of this area, the structural information of proteins is not always available, particularly for the huge amount of uncharacterized protein sequences generated in the post genomic age. The 2^nd^ type is purely based on the protein sequence information alone (see, e.g., [6]–[15]). These methods did stimulat the development by extending the identification power to cover those proteins without any structural information at all, and by using various modes of pseudo amino acid composition [16] or Chou's PseAAC [17] to take into account some sequence-order effects for enhancing the prediction quality.

It shoud be pointed out that most of existing methods did not provide a web-server, and hence their applications might be limited, particularly for the majority of expermental scientists who were not trained in the field of computational biology. Also, although some of the existing methods did provide a web-server, they took reletively longer computational time for each single prediction. For a high throughput tool in dealing with huge amount of protein sequences, the less time it needs in identifying each query sample, the better and more useful the high throughput tool will be.

The present study was initiated in an attempt to develop a new sequence-based predictor for identiying the DNA-binding proteins from the aforementioned two aspects.

As demonstrated by a series of recent publications [18]–[26] and called by Chou [27], it would make the development of new predictor logically more clear and practically more useful if it is documented according to the following procedures: (i) construct or select a valid benchmark dataset to train and test the predictor; (ii) formulate the samples with an effective mathematical expression that can truly reflect their intrinsic correlation with the target to be predicted; (iii) introduce or develop a powerful algorithm (or engine) to operate the prediction; (iv) properly perform cross-validation tests to objectively evaluate its anticipated accuracy; (v) establish a user-friendly web-server for the predictor that is accessible to the public. Below, we are to describe the new predictor according to the five procedures.

## Materials and Methods

### 2.1. Benchmark Datasets

To develop a statistical predictor, it is first important thing to establish a reliable and stringent benchmark dataset to train and test the predictor. If the benchmark dataset contains some errors, the predictor trained by it must be unreliable and the accuracy tested by it would be completely meaningless. Also, according to a comprehensive review [28], there is no need to separate a benchmark dataset into a training dataset and a testing dataset if the performance of a predictor is tested by the jackknife test or subsampling (K-fold) cross-validation test because the outcome thus obtained is actually from a combination of many different independent dataset tests. Thus, the benchmark dataset for the current study can be formulated as
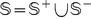
(1)where the positive subset 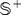 only contains DNA-binding proteins, the negative subset 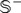 only contains non DNA-binding proteins, and the symbol 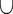 represents the “union” in the set theory. The DNA-binding proteins were extracted from the recent release of Protein Data Bank (PDB) (Dec, 2013) by searching the mmCIF keyword of ‘DNA binding protein’ through the advanced search interface. To construct a high quality and non-redundant positive benchmark dataset, the DNA-binding proteins were filtered strictly according to the following criteria. (i) Proteins with less than 50 residues in length were removed since they might be just fragments. (ii) Proteins containing the residue ‘X’ were removed because they contained unknown residue. (iii) The sequence similarity between any two proteins in 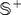 should be lower than 25% by using PISCES [29] to reduce the redundancy. Finally, we got 525 DNA-binding proteins for 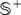. The 550 negative samples in 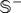, i.e., the non-DNA-binding proteins, were randomly selected from other proteins in PDB and were filtered according to the same criteria as mentioned above. The codes of the 525+550 = 1,075 protein samples as well as their detailed sequences are given in the Supporting Information S1. To the best of our knowledge, the benchmark dataset thus formed is not only the most stringent one but also posses the highest number of DNA-binding proteins, in comparison with the previous benchmark datasets used for developing the existing prediction methods for the same purpose.

### 2.2. PseAAC of Distance-Pairs and Reduced Alphabet Scheme

One of the most challenging problems in computational biology today is how to effectively formulate a biological sequence with a discrete model or a vector, yet still keep considerable sequence order information. This is because, on the one hand, the number of biological sequences with different sequence-orders is extremely high and their lengths vary widely; but on the other hand, all the existing operation engines, such as covariance discriminant (CD) [30]–[32], neural network [33], support vector machine (SVM) [34], [35], random forest [15], [36], conditional random field [26], nearest neighbor (NN) [37], K-nearest neighbor (KNN) [38], OET-KNN [39], [40], Fuzzy K-nearest neighbor [41], [42], ML-KNN algorithm [43], and SLLE algorithm [32], can only handle vector but not length-different sequences. However, a vector defined in a discrete model may totally miss the sequence-order information.

To deal with such a dilemma, the approach of pseudo amino acid composition [16], [44] or Chou's PseAAC [17] was proposed. Ever since it was introduced in 2001 [16], the concept of PseAAC has been rapidly penetrated into almost all the areas of computational proteomics, such as in identifying bacterial virulent proteins [45], predicting super-secondary structure [46], predicting anticancer peptides [47], predicting protein subcellular location [48], predicting membrane protein types [49], discriminating outer membrane proteins [50], analysing genetic sequence [51], identifying cyclin proteins [52], predicting GABA(A) receptor proteins [53], identifying antibacterial peptides [54], predicting anticancer peptides [47], identifying allergenic proteins [55], predicting metalloproteinase family [56], predicting protein structural class [57], identifying GPCRs and their types [58], identifying protein quaternary structural attributes [59], predicting protein submitochondria locations [60], identifying risk type of human papillomaviruses [61], among many others (see a long list of references cited in a 2014 article [62] as well as a 2009 review [63]). Recently, the concept of PseAAC was further extended to represent the feature vectors of DNA and nucleotides [23], [24], [34], [64]. Because it has been widely and increasingly used, recently three types of powerful open access soft-ware, called ‘PseAAC-Builder’ [65], ‘propy’ [66], and ‘PseAAC-General’ [62], were established: the former two are for generating various modes of Chou's special PseAAC; while the 3^rd^ one for those of Chou's general PseAAC.

Given a protein sequence 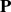 consisting of 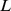 amino acids as formulated by 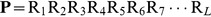
(2)where 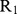 represents the 1^st^ residue, 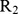
the 2^nd^ residue, …, its PseAAC can be generally formulated as a vector given by [67]

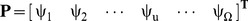
(3)where 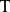 is the transpose operator, while 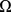 an integer to reflect the vector's dimension. The value of 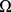 as well as the components 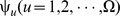 in **Eq. 3** will depend on how to extract the desired information from a protein sequence. Below, let us describe how to extract the useful information from the benchmark datasets to define the protein samples via **Eq. 3**.

In order to capture the sequence-order information for the residues in **P** of **Eq. 2**, let us first introduce a concept called the occurrence frequency of “distance amino acid pair” or just “distance-pair”, as formulated by 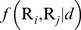
(4)where R*_i_* and R*_j_* can be any of the 20 native amino acids in a protein chain (cf. **Eq. 2**), and *d* represents the distance counted by the number of amino acids between R*_i_* and R*_j_* along the protein chain. Suppose R*_i_* is A (alanine), R*_j_* is K (lysine), and *d* = 3, then 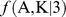 means the occurrence frequency of the A–K pair with its two counterparts separated by 2 residues along the protein chain. Thus, when *d* = 0, **Eq. 4** is reduced to
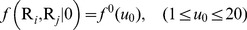
(5)meaning the occurrence frequencies of the 20 native amino acids in the protein or its amino acid composition [68]; when *d* = 1, we have 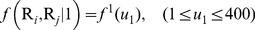
(6)meaning the occurrence frequencies of the nearest residue-pairs [69], [70]; when *d* = 2, we have 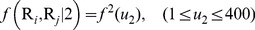
(7)meaning the occurrence frequencies of the second nearest residue-pairs [71]; and so forth.

Accordingly, using the distance-pair concept, the general PseAAC of **Eq. 3** can be uniquely defined as a vector with dimension 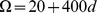 where each component is given by
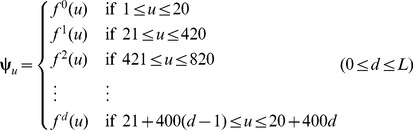
(8)


### 2.3. Reduced Amino Acid Alphabet Scheme

Although the distance-pair approach as described above can incorporate more sequence-order information by gradually increasing the value of integer *d*, the dimension of the PseAAC vector **P** will be rapidly increased as well. For example, when *d* = 100, the dimension of the vector **P** (cf. **Eqs. 3** and **8**) will be 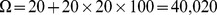. This will cause the high-dimension disaster [72] as reflected by the following disadvantages: (i) unnecessarily increasing the computational time; (ii) misrepresentation due to information redundancy or noise that will lead to poor prediction accuracy; and (iii) the overfitting problem that will make the predictor with a serious bias and extremely low capacity for generalization.

Similar high-dimension disaster problems did also occur in many other areas of bioinformatics. To overcome these problems, the strategy to reduce amino acid alphabet had been adopted by some previous investigators. For instance: Feng et al. [73] used the strategy to improve the prediction quality for identifying the heat shock protein families, and Peterson et al. [74] applied it for protein fold assignment.

Below, we are to propose a reduced alphabet approach to significantly cut down the dimension of the PseAAC vector and improve the predictive performance. Suppose 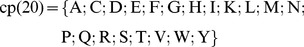
(9)is the original 20 amino acid profile. After testing 164 reduced alphabet schemes downloaded from http://www.rpgroup.caltech.edu/publications/supplements/peterson2009/HP/Welcome.html collected by Peterson et al. [74], we found three amino acid cluster profiles were quite promising for identifying DNA-binding proteins. They are cp(13), cp(14), and cp(15) as defined below
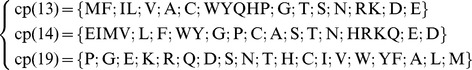
(10)where the single letters without a semicolon (;) to separate them mean belonging to a same cluster. Suppose *n*(c) represents the number of clusters for a given profile, we have
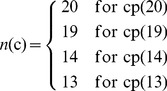
(11)


Now, to make our formulation able to cover the reduced amino acid alphabet profiles, **Eq. 8** should be changed to
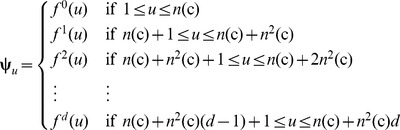
(12)and the corresponding dimension for the general PseAAC of **Eq. 3** would be changed to
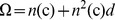
(13)


For example, if using the reduced amino acid alphabet profile cp(13) (or *n*(c) = 13) to replace the conventional 20 amino acid profile cp(20) (or *n*(c) = 20), and the maximum pairwise distance considered is *d* = 3, then the dimension 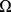 will be reduced from 1,220 to 520.

**Figure 1:**
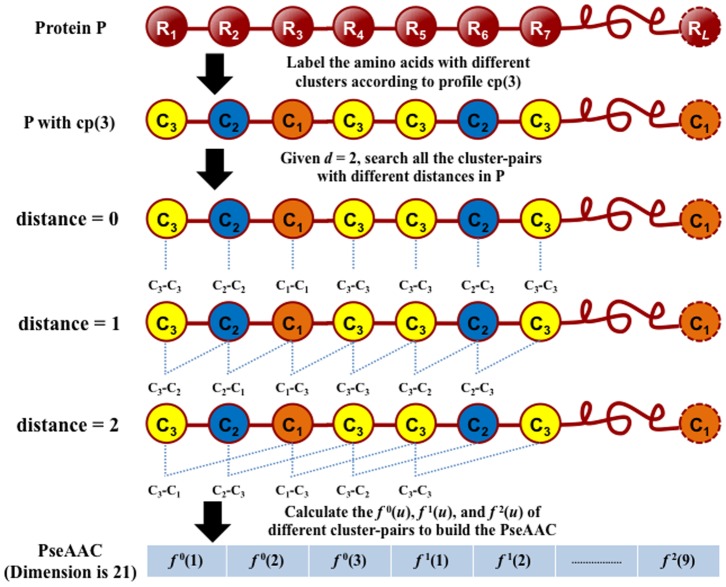
An example to show the process of generating the PseAAC of Distance-Pairs with Reduced Alphabet Scheme cp(3). The characters C_1_, C_2_, and C_3_ represent the three different clusters and are coloured with orange, blue, and yellow, respectively. When the maximum pairwise distance *d* = 2, the occurrence frequencies 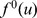, 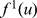, and 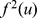 can be derived from Eq. 12 and the corresponding dimension for the PseAAC vector is 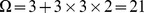. See the test for further explanation.

Shown in **Fig. 1** is a simple example to illustrate how to generate the PseAAC of the distance-pairs for the reduced amino acid alphabet cp(3) as given by
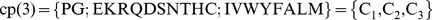
(14)where C_1_, C_2_, and C_3_ represent the three different clusters and are colored in **Fig. 1** with orange, blue, and yellow, respectively. When the maximum pairwise distance *d* = 2, the occurrence frequencies 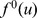, 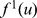, and 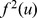 can be derived from **Eq. 12**, and the dimension for the corresponding PseAAC vector is 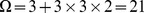.

### 2.4. Support Vector Machine

SVM is based on the structural risk minimization principle from statistical learning theory. SVM has been widely used in the realm of bioinformatics (see, e.g., [19], [20], [22]–[25], [34], [35], [75]–[78]). The basic idea of SVM is to construct a separating hyper-plane so as to maximize the margin between the positive dataset and negative dataset. The nearest two points to the hyper-plane are called support vectors. SVM first constructs a hyper-plane based on the training dataset, and then maps an input vector 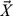 from the input space into a vector in a higher dimensional Hillbert space, where the mapping is determined by a kernel function. A trained SVM can output a class label (in our case, DNA-binding protein or non DNA-binding protein) based on the mapping vector of the input vector. In the current study, the LIBSVM algorithm [79] was employed, which is a software for SVM classification and regression. The kernel function was set as Radial Basis Function (RBF) and the two parameters *C* and 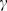 were optimized on the benchmark dataset by adopting the grid tool provide by LIBSVM [79].

For a brief formulation of SVM and how it works, see the papers [80], [81]; for more details about SVM, see a monograph [82].

### 2.5. Evaluation Method of Performance

How to properly examine the prediction quality is a key for developing a new predictor and estimating its potential application value. Generally speaking, to avoid the “memory effect” [28] of the resubstitution test in which a same dataset was used to train and test a predictor, the following three cross-validation methods are often used to examine a predictor for its effectiveness in practical application: independent dataset test, subsampling or K-fold (such as 5-fold, 7-fold, or 10-fold) test, and jackknife test [83]. However, as elaborated by a penetrating analysis and demonstrated by Eqs. 28–30 in [67], considerable arbitrariness exists in the independent dataset test and the K-fold cross validation. Only the jackknife test is the least arbitrary that can always yield a unique result for a given benchmark dataset. Therefore, the jackknife test has been widely recognized and increasingly adopted by investigators to examine the quality of various predictors (see, e.g., [47], [49], [55], [84]–[86]). Accordingly, the jackknife test was also used to examine the performance of the model proposed in the current study. In the jackknife test, each of the proteins in the benchmark dataset is in turn singled out as an independent test sample and all the rule-parameters are calculated without including the one being identified.

Also, in literature a set of four metrics called the sensitivity (Sn), specificity (Sp), accuracy (Acc), and Mathew's correlation coefficient (MCC), are often used to measure the test quality of a predictor from four different angles
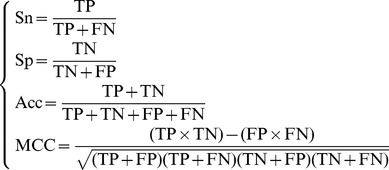
(15)where TP represents the number of the true positive; TN, the number of the true negative; FP, the number of the false positive; FN, the number of the false negative; Sn, the sensitivity; Sp, the specificity; Acc, the accuracy; MCC, the Mathew's correlation coefficient. To most biologists, unfortunately, the four metrics as formulated in **Eq. 15** are not quite intuitive and easy-to-understand, particularly the equation for MCC. Here let us adopt the formulation proposed recently in [26], [34], [71] based on the symbols introduced by Chou [87], [88] in predicting signal peptides. According to the formulation, the same four metrics can be expressed as
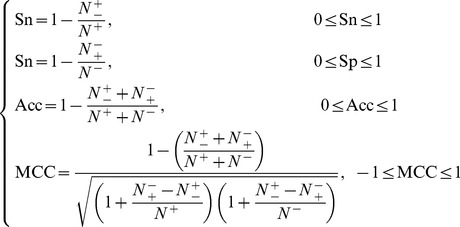
(16)where *N* ^+^is the total number of the DNA-binding proteins investigated whereas 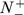 the number of the DNA-binding proteins incorrectly predicted as non DNA-binding proteins; *N* ^−^ the total number of the non DNA-binding proteins investigated whereas 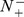 the number of the non DNA-binding proteins incorrectly predicted as the DNA-binding proteins.

According to **Eq. 16** we can easily see the following. When 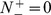 meaning none of the DNA-binding proteins was mispredicted to be a non-DNA-binding protein, we have the sensitivity Sn = 1; while 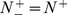 meaning that all the DNA-binding proteins were mispredicted to be the non-DNA-binding proteins, we have the sensitivity Sn = 0. Likewise, when 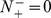 meaning none of the non- DNA-binding proteins was mispredicted, we have the specificity Sp = 1; while 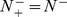 meaning all the non-DNA-binding proteins were incorrectly predicted as DNA-binding proteins, we have the specificity Sp = 0. When 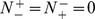 meaning that none of the DNA-binding proteins in the dataset 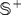
and none of the non-DNA-binding proteins in 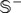 was incorrectly predicted, we have the overall accuracy Acc = 1; while 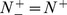
and 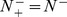 meaning that all the DNA-binding proteins in the dataset 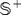
and all the non-DNA-binding proteins in 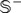 were mispredicted, we have the overall accuracy Acc = 0. The Matthews correlation coefficient (MCC) is usually used for measuring the quality of binary (two-class) classifications. When 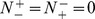 meaning that none of the DNA-binding proteins in the dataset 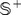
and none of the non-DNA-binding proteins in 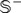 was mispredicted, we have MCC = 1; when 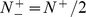 and 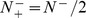 we have MCC = 0 meaning no better than random prediction; when 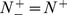
and 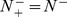
we have MCC = −1 meaning total disagreement between prediction and observation. As we can see from the above discussion, it is much more intuitive and easier to understand when using **Eq. 16** to examine a predictor for its four metrics, particularly for its Mathew's correlation coefficient. It is instructive to point out that the metrics as defined in **Eq. 16** are valid for single label systems; for multi-label systems, a set of more complicated metrics should be used as given in [43].

## Results and Discussion

### 3.1 Impact of the Pairwise Distance on the iDNA-Prot|dis Predictor

**Figure 2:**
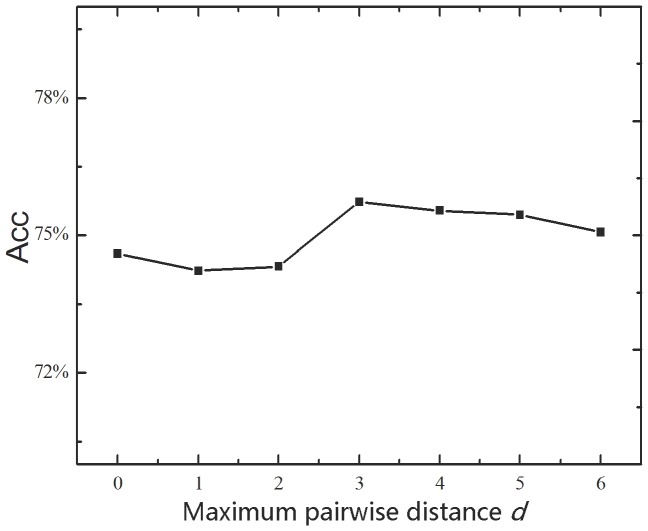
The overall Acc values achieved by iDNA-Prot|dis for cp(20) with different *d* values based on the benchmark dataset through five-cross validation.

There is a parameter, the maximum pairwise distance *d*, in the proposed method iDNA-Prot|dis (see **Eqs. 12–13**), which would affect its performance. The pairwise distance *d* can be any integer between 0 and the length of the longest protein sequence in the training dataset. For the sake of reducing computational time, the optimal value for *d* was derived via the five-cross validation on the benchmark dataset. The overall Acc values with different *d* thus obtained are shown in **Fig. 2**, from which we can see that iDNA-Prot|dis achieves the best performance when *d = 3*. Hereafter, the parameter *d* was set as 3 for further investigation.

### 3.2. Discriminant Visualization and Interpretation

To further investigate the importance of the features and reveal the biological meaning of the feature space in iDNA-Prot|dis, we followed the study [89] to calculate the discriminant weight vector in the feature space. The sequence-specific weight obtained from the SVM training process can be used to calculate the discriminant weight of each feature to measure the importance of the features. Given the weight vectors of the training set with *N* samples obtained from the kernel-based training **A** =  [*a*_1_, *a*_2_, *a*_3_,…, *a_N_*], the feature discriminant weight vector **W** in the feature space can be calculated by the following equation:
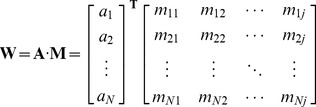
(17)where **M** is the matrix of sequence representatives; **A** is the weight vectors of the training samples; *N* is the number of training samples; *j* is the dimension of the feature vector. The element in **W** represents the discriminative power of the corresponding feature. In order to reveal the biological meaning of the proposed feature space, the sum score of the positive discriminant weights for each amino acid pair was calculated.

**Figure 3:**
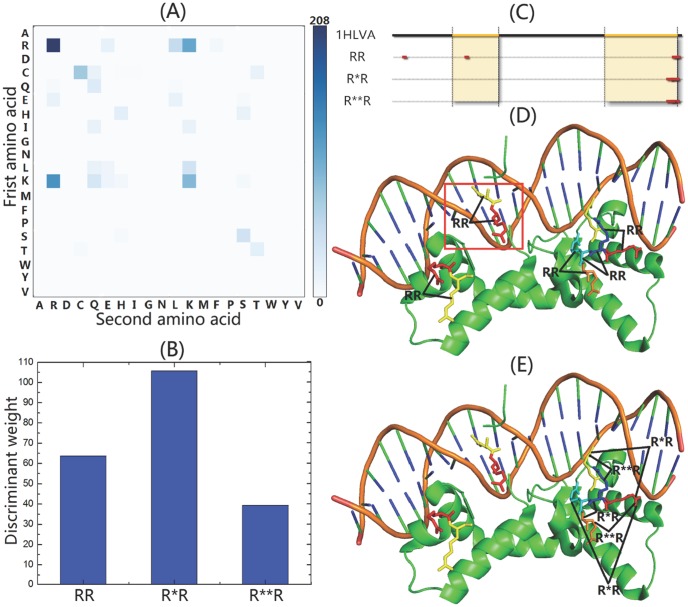
An illustration for discriminant visualization and interpretation. (A) The discriminative power of the 400 amino acid pairs. Each element in this figure represents the sum score of the features with positive discriminant weights for a specific distance amino acid pair with *cp(20)*. The amino acids are identified by their one-letter code. The amino acids labelled by horizontal-axis and vertical-axis indicate the first amino acid and the second amino acid in the pairs, respectively. The adjacent colour bar shows the mapping of sum score values. (B) The different discriminant weights of distance amino acid pairs R-R. There are three kinds of features with positive discriminative power for amino acid pair R-R, including RR, R*R, and R**R with distance 1, 2, 3, respectively. (C) The occurrence distribution of RR, R*R, and R**R in the sequence of protein 1HLVA. The total occurrences of the three features are ten, which are shown in red dots. The two DNA-binding regions (sequence position 28–48, and 97–129) are shown in yellow colour. (D) The distribution of RR in the three dimensional structure of 1HLVA. Only one RR occurs outside of the two DNA-binding regions, which was shown in red square. (E) The distribution of R*R and R**R in the three dimensional structure of 1HLVA.

The discriminative power of all the 400 distance amino acid pairs in iDNA-Prot|dis is depicted in **Fig. 3A**. Each element in this figure represents the sum score of the features with positive discriminant weights for a specific distance amino acid pair. The top three most discriminative amino acid pairs are R-R, K-R, and R-K according to the three darkest spots in **Fig. 3A**, which indicates the importance of amino acid R (Arg) and K (Lys) for DNA-binding protein identification. These results are fully consistent with the previous studies [90]. It is well-known that the positively charged amino acids, such as Arg and Lys are critical for DNA-binding function. This is probably the reason why these two amino acids show strong positive discriminative power. A specific DNA-binding protein 1HLV chain A was selected to investigate if the most discriminative distance amino acid pairs R-R reflect the characteristics of this DNA-binding protein. 1HLV also known as human centromere protein B (CENP-B), is a human centromere component that binds to satellite repeats regions in major grooves of the DNA with its two helix-turn-helix DNA binding domains. The helix-turn-helix structure, which usually appears in repressor proteins and about 20 amino acids in length, is among the most common DNA binding domains that were found in protein. The two DNA-binding regions of 1HLVA protein are located at sequence position 28–48, and 97–129. For iDNA-Prot|dis with *d* = 3, there are three kinds of features with positive discriminative power for distance amino acid pair R-R, including RR, R*R, and R**R with distance 1, 2, 3, respectively. Their discriminant weights are shown in **Fig. 3B**. According to this figure, R*R shows higher discriminative power than other two features. The distributions of the features in the protein sequence of 1HLVA are shown in **Fig. 3C**. The total occurrences of the three kinds of features are ten, interestingly, nine of them occur within the two DNA-binding regions in 1HLVA, indicating R-R indeed reflects the characteristics of this DNA-binding protein, especially for the DNA-binding regions. This is further confirmed by the three dimensional structure shown in **Fig. 3D and E**, only one RR pair is out of DNA-bind region shown in red square, and all the other nine occurrences are within the two DNA-binding regions. In Tanaka et al.'s paper [91], the authors determined 1HLV's DNA binding domain structure with high resolution and found that the arginine rich region of the second domain is indeed critical for the protein helix and DNA major groove interaction by a mechanism known as ‘phosphate bridging by an arginine-rich helix’ (PBAH), which explains the reason why the amino acid pair R-R shows strong discriminative power.

### 3.3. Reduced Amino Acid Alphabet Scheme

A reduced alphabet is any clustering of amino acids based on some measure of their relative similarity, such as physical-chemical properties [30], [92], structural alignment [93], protein alignment, and sequence secondary structure. Recent studies showed that the reduced alphabet scheme can improve the performance and reduce the computational cost of some predictors for protein remote homology detection, fold recognition, protein disordered region prediction [74], [94], [95], etc. In this section, we investigated whether the predictive performance and computational cost of iDNA-Prot|dis can be further improved by employing the reduced alphabet scheme. After testing over 150 reduced alphabet profiles collected by Peterson et al. [74], the three top-performing amino acid profiles and their predictive results are shown in **Table. 1**, from which we can see that the performance of iDNA-Prot|dis is further improved, and it achieved, when using the cluster profile cp(14), the overall accuracy of 77.03% in identifying proteins as DNA-binding proteins and non-DNA-binding proteins. And the corresponding vector dimension used for computation was reduced from 1,220 of cp(20) to 14+14×14×3 = 602 of cp(14). Therefore, the reduced amino acid alphabet approaches are indeed an efficient approach for DNA-binding protein identification, which could not only improve the prediction quality, but also reduce the computational cost as well as the risk of over-fitting.

**Table 1:** The jackknife test results by iDNA-Prot|dis with different amino acid alphabet profiles (cf. Eqs. 9–13) on the benchmark dataset of Eq. 1 (cf. Supporting Information S1).

Cluster profile	Acc (%)	MCC	Sn(%)	Sp(%)	AUC(%)
cp(20)^a^	75.81	0.52	81.14	70.72	83.40
cp(19)^b^	76.46	0.53	82.28	70.90	83.30
cp(14)^c^	**77.30**	**0.54**	79.40	75.27	82.60
cp(13)^d^	77.20	0.54	80.76	73.81	83.10

a The parameters used: *d* = 3, *C* = 4, 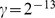.

b The parameters used: *d* = 3, *C* = 4, 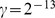.

c The parameters used: *d* = 3, *C* = 2, 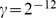.

d The parameters used: *d* = 3, *C* = 64, 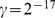.

### 3.4. Comparison with Other Related Methods

Shown in **Table 2** are the jackknife results by iDNA-Prot|dis and four other state-of-the-art methods on the same benchmark dataset. The three other methods are DNAbinder (dimension 21) [96], DNAbinder (dimension 400) [96], DNA-Prot [14] and iDNA-Prot [15]. Among these four methods, DNAbinder (dimension 21), DNAbinder (dimension 400) are profile-based methods. The other two methods are sequence-based methods, in which the features were extracted from protein sequences.

**Table 2:** A comparison of the jackknife test results by iDNA-Prot|dis with the other methods on the benchmark dataset of Eq. 1.

Method	Acc(%)	MCC	Sn(%)	Sp(%)	AUC(%)
iDNA-Prot|dis (cp(14))^a^	77.30	0.54	79.40	75.27	82.60
DNAbinder (dimension 21)^b^	73.95	0.48	68.57	79.09	81.40
DNAbinder (dimension 400)^c^	73.58	0.47	66.47	80.36	81.50
DNA-Prot^d^	72.55	0.44	82.67	59.76	78.90
iDNA-Prot^e^	75.40	0.50	83.81	64.73	76.10

a See the footnote c of Table 1.

b Results obtained by in-house implementation from DNAbinder [96].

c Results obtained by in-house implementation from DNAbinder [96].

d Results obtained by in-house implementation from DNA-Prot [14].

e Results obtained by in-house implementation from iDNA-Prot [15].

**Figure 4:**
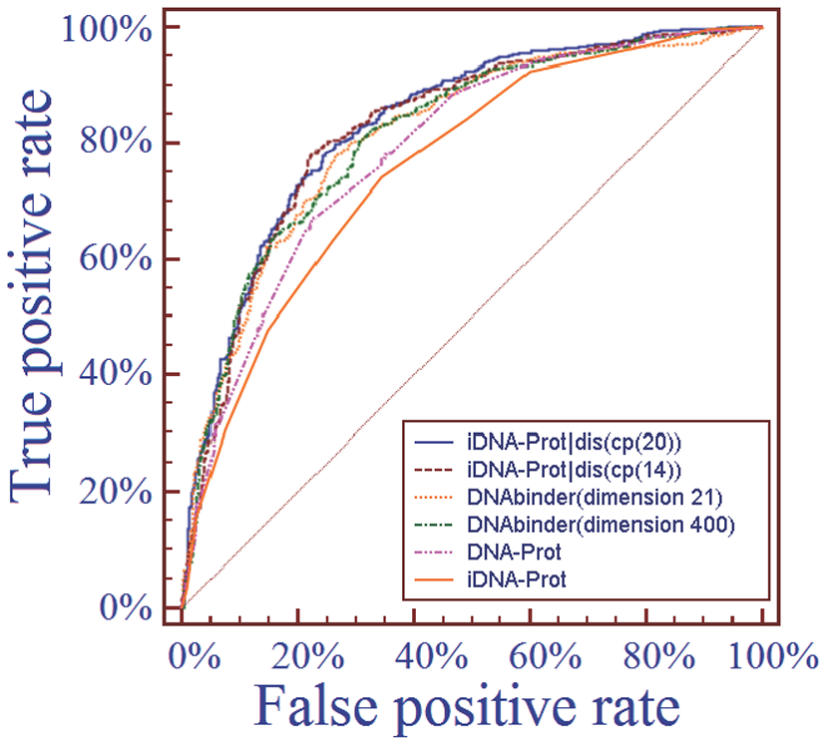
The ROC (receiver operating characteristic) curves obtained by different methods on the benchmark dataset using the jackknife tests. The areas under the ROC curves or AUC are 0.834, 0.826, 0.814, 0.815, 0.789 and 0.761 for iDNA-Prot|dis (cp(20)), iDNA-Prot|dis (cp(14)), DNAbinder (dimension 21), DNAbinder(dimension 400), DNA-Prot and iDNA-Prot, respectively. See the main text for further explanation.

Furthermore, to provide a graphic illustration to show the performances of the four predictors, the corresponding ROC (receiver operating characteristic) curves were drawn in **Fig. 4**, where the horizontal coordinate X is for the false positive rate or 1-Sp, and the vertical coordinate Y is for the true positive rate or Sn. The best possible method would yield a point with the coordinate (0, 1) meaning 0 false positive rate (or 100% specificity), and 0 false negative rate (or 100% sensitivity). Therefore, the (0,1) point is also called a perfect classification. A completely random guess would give a point along a diagonal from the point (0,0) to (1,1). The area under the ROC curve is called AUC, which is often used to indicate the performance quality of a binary classification predictor: the larger the area, the better the prediction quality is.

From **Table 2** and **Fig. 4** we can see that the iDNA-Prot|dis outperformed all the other methods.

### 3.5. Independent Test

**Figure 5:**
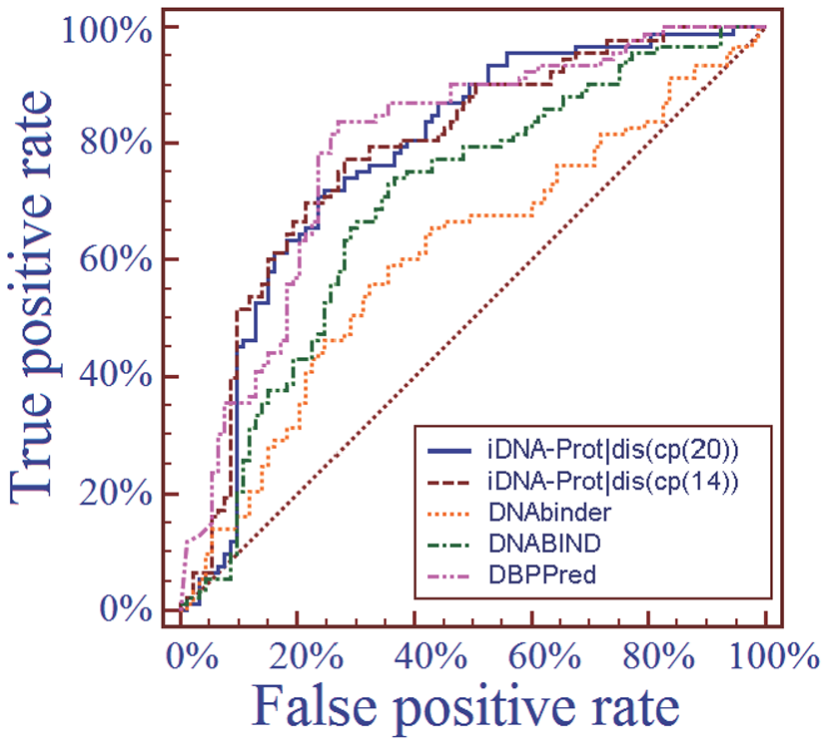
The ROC (receiver operating characteristic) curves obtained by different methods on the independent dataset PDB186. The areas under the ROC curves or AUC are 0.786, 0.779, 0.607, 0.694, and 0.791 for iDNA-Prot|dis(cp(20)), iDNA-Prot|dis(cp(14)), DNAbinder, DNABIND and DBPPred, respectively. See the main text for further explanation.

Moreover, as a demonstration, we also extended the comparison with other methods via an independent dataset test. To realize this, we used the dataset PDB186 recently constructed by Lou et al. [97] as the independent dataset, in which 93 proteins are DNA-binding proteins and 93 proteins are non- DNA-binding proteins. To avoid the homology bias, the NCBI's BLASTCLUST [98] was used to remove those proteins from the benchmark dataset that have more than 25% sequence identity to any protein within a same subset of the PDB186 dataset. Trained with such a reduced benchmark dataset, the iDNA-Prot|dis predictor was used to identify the proteins in the PDB186 dataset. The results thus obtained are given in **Table 3** and **Fig. 5**, where for facilitating comparison, the corresponding results by other methods are also shown the table and figure. It can be clearly seen from there that the new predictor outperformed all the existing predictors for the same purpose.

**Table 3:** A comparison of the results^a^ obtained by iDNA-Prot|dis and the other methods on the independent dataset PDB186.

Methods	Acc(%)	MCC	Sn(%)	Sp(%)	AUC(%)
iDNA-Prot|dis	**72.00**	0.445	79.50	64.50	78.60
iDNA-Prot	67.20	0.344	67.70	66.70	N/A
DNA-Prot	61.80	0.240	69.90	53.80	N/A
DNAbinder	60.80	0.216	57.00	64.50	60.70
DNABIND	67.70	0.355	66.70	68.80	69.40
DNA-Threader	59.70	0.279	23.70	95.70	N/A
DBPPred	76.90	0.538	79.60	74.20	79.10

a The results of iDNA-Prot [15], DNA-Prot [14], DNAbinder [96], DNABIND [102], DNA-Threader [5], and DBPPred [97] were obtained from [97].

### 3.6. Web-Server Guide

As pointed out in [99] and realized in a series of recent publications (see, e.g., [17], [26], [71], [100], [101]), user-friendly and publicly accessible web-servers represent the future direction for developing practically more useful predictors, we have also established a web-server for the current iDNA-Prot|dis predictor. Furthermore, for the convenience of the vast majority of experimental scientists, below let us give a step-by-step guide on how to use the web-server to get their desired results without the need to follow the complicated mathematic equations.

#### Step 1

**Figure 6:**
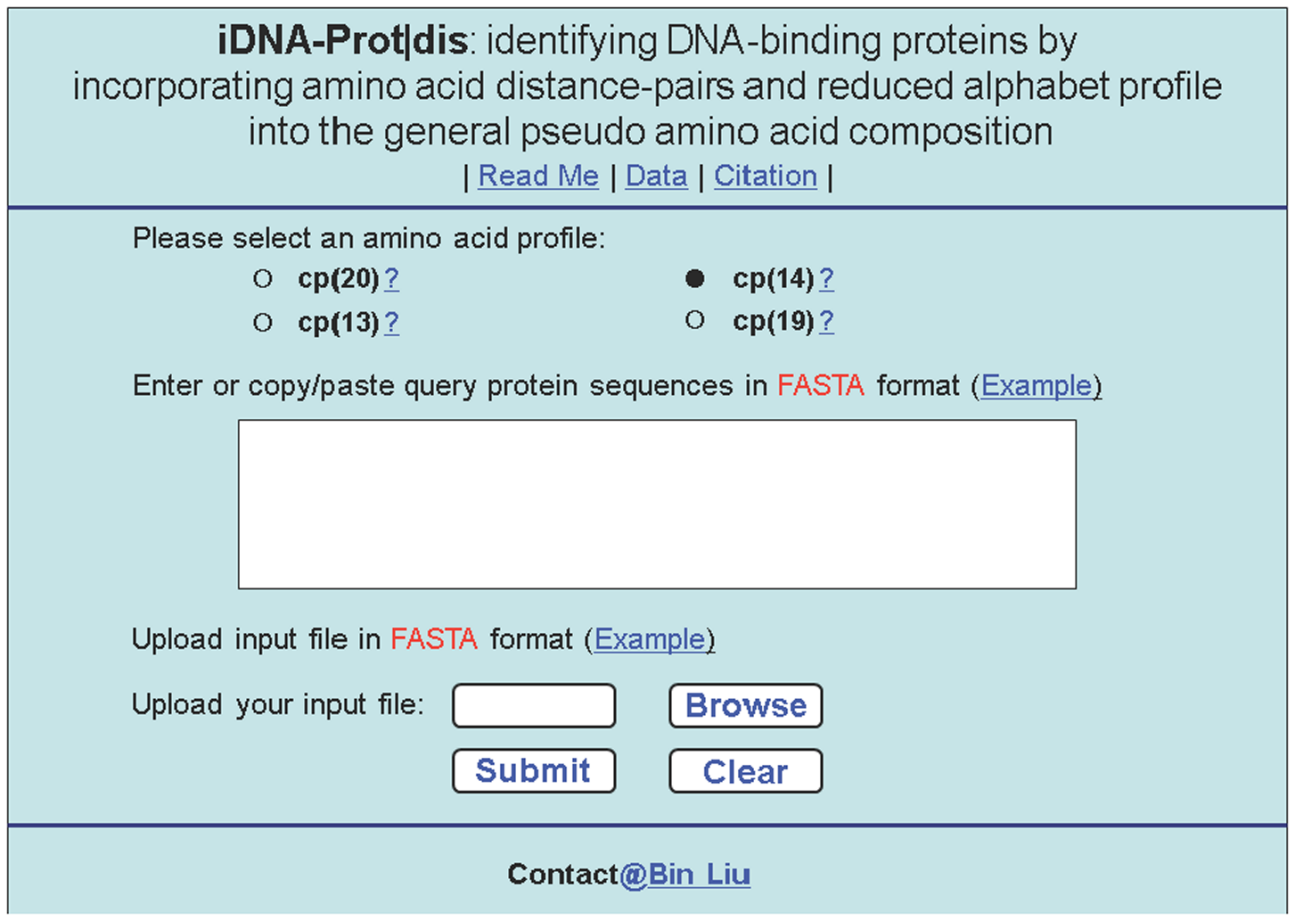
A semi-screenshot to show the top page of the web-server iDNA-Prot|dis, which is available at http://bioinformatics.hitsz.edu.cn/iDNA-Prot_dis/.

Open the web-server by clicking the link at http://bioinformatics.hitsz.edu.cn/iDNA-Prot_dis/ and you will see its top page as shown in **Fig. 6**. Click on the Read Me button to see a brief introduction about the server.

#### Step 2

Check the open circle to select which alphabet profile you are to use for conduct prediction.

#### Step 3

Either type or copy and paste the query protein sequence into the input box at the center of **Fig. 6**, or you can also upload your input data by the Browse button. The input sequence should be in the FASTA format. A sequence in FASTA format consists of a single initial line beginning with the symbol, >, in the first column, followed by lines of sequence data in which nucleotides or amino acids are represented using single-letter codes. Except for the mandatory symbol >, all the other characters in the single initial line are optional and only used for the purpose of identification and description. The sequence ends if another line starting with the symbol > appears; this indicates the start of another sequence. Example sequences in FASTA format can be seen by clicking on the Example button right above the input box.

#### Step 4

Click on the Submit button to see the predicted result. For example, if you use the four query protein sequences in the Example window as the input and select profile “cp(14)” for prediction, after clicking the Submit button, you will see on your screen that the predicted results for the 1^st^ and 2^nd^ proteins are “**DNA-binding Protein**”, and the other two proteins are “**Non DNA-binding Protein**”, fully consistent with experimental observations. However, if you select the alphabet profile “cp(20)” for prediction, the 2^nd^ and 4^th^ proteins cannot be correctly identified, indicating that the reduced alphabet approach can improve the prediction quality of iDNA-Prot|dis.

## Conclusions

DNA-binding proteins play crucial roles in various cellular processes, and hence it is a big challenge to develop a high throughput tool for rapidly and effectively distinguishing them from non-DNA-binding proteins based on their sequence information alone.

One of the most challenging and difficult problems in computational biology today is how to effectively formulate a biological sequence with a discrete model or a vector, yet still keep considerable sequence order information.

To deal with this problem, the predictor iDNA-Prot|dis proposed in this paper was developed by incorporating various distance-pairwise coupling information into the general form of pseudo amino acid composition. To avoid dimension disaster and reduce computational time, the reduced amino acid alphabet strategy was adopted. That is why the new predictor can outperform the existing predictors in identifying DNA-binding proteins with less computational time.

It is anticipated that the iDNA-Prot|dis predictor will become a high throughput tool for both basic research and drug development.

## Supporting Information

Supporting Information S1 **The benchmark dataset.** It contains 1075 protein sequences, of which 525 are DNA-binding proteins (positive samples) and 550 are non-DNA-binding proteins (negative samples). See Eq. 1 and the relevant text for further explanation. The Benchmark dataset is available at http://bioinformatics.hitsz.edu.cn/iDNA-Prot_dis/Resources/benchmark_dataset.pdf.
(PDF)
