# Supplementary material for: iDNA-Prot|dis: Identifying DNA-Binding Proteins by Incorporating Amino Acid Distance-Pairs and Reduced Alphabet Profile into the General Pseudo Amino Acid Composition
Source: PLoS One. 2014 Sep 3;9(9):e106691. doi: 10.1371/journal.pone.0106691 (PMC4153653; doi:10.1371/journal.pone.0106691)
Supplement: Supporting Information S1 — The benchmark dataset. It contains 1075 protein sequences, of which 525 are DNA-binding proteins (positive samples) and 550 are non-DNA-binding proteins (negative samples). See Eq. 1 and the relevant text for further explanation. The Benchmark dataset is available at http://bioinformatics.hitsz.edu.cn/iDNA-Prot_dis/Resources/benchmark_dataset.pdf. (PDF) [file pone.0106691.s001.pdf]

The benchmark dataset $\mathbb{S}$ contains 1,075 protein sequences, which are classified into subset $\mathbb{S}^{+}$ with 525 DNA-binding proteins (positive samples) and subset $\mathbb{S}^{-}$ with 550 non-DNA-binding proteins (negative samples).

---

(1) 525 DNA-binding proteins

>1AKHA
KKEKSPKGKSSISPQARAFLEEVFRRKQSLNSKEKEEVAKKCGITPLQVRVWFINKRMRSK
>1AOII
ATCAATATCCACCTGCAGATTCTACCAAAAGTGTATTTGGAAACTGCTCCATCAAAAGGCATGTT
CAGCTGAATTCAGCTGAACATGCCTTTTGATGGAGCAGTTTCCAAATACACTTTTGGTAGAATCT
GCAGGTGGATATTGAT
>1B6WA
MELPIAPIGRIIKDAGAERVSDDARITLAKILEEMGRDIASEAIKLARHAGRKTIKAEDIELAVR
RFKK
>1C1KA
MIKLRMPAGGERYIDGKSVYKLYLMIKQHMNGKYDVIKYNWCMRVSDAAYQKRRDKYFFQKLSEK
YKLKELALIFISNLVANQDAWIGDISDADALVFYREYIGRLKQIKFKFEEDIRNIYYFSKKVEVS
AFKEIFEYNPKVQSSYIFKLLQSNIISFETFILLDSFLNIIDKHDEQTDNLVWNNYSIKLKAYRK
ILNIDSQKAKNVFIETVKSCKY
>1C6VX
QQSKNSKFKNFRVYYREGRDQLWKGPGELLWKGEGAVLLKVGTDIKVVPRRKAKIIKDYGGGKEV
DSSSHMEDTGEAREVA
>1C6VD
IHGQVNSDLGTWQMDCTHLEGKIVIVAVHVASGFIEAEVIPQETGRQTALFLLKLAGRWPITHLH
TDNGANFASQEVKMVAWWAGIEHTFGVPYNPQSQGVVEAMNHHLKNQIDRIREQANSVETIVLMA
VHCMNHKRRGGIGDMTPAERLINMITTEQEIQFQ
>1CI4B
MTTSQKHRDFVAEPMGEKPVGSLAGIGEVLGKKLEERGFDKAYVVLGQFLVLKKDEDLFREWLKD
TCGANAKQSRDCFGCLREWCDAFL
>1D4UA
MEFDYVICEECGKEFMDSYLMDHFDLPTCDDCRDADDKHKLITKTEAKQEYLLKDCDLEKREPPL
KFIVKKNPHHSQWGDMKLYLKLQIVKRSLEVWGSQEALEEAKEVRQ
>1D8BA
ELNNLRMTYERLRELSLNLGNRMVPPVGNFMPDSILKKMAAILPMNDSAFATLGTVEDKYRRRFK
YFKATIADLSKKRSSE
>1DMLG
MTDSPGGVAPASPVEDASDASLGQPEEGAPCQVVLQGAELNGILQAFAPLRTSLLDSLLVMGDRG
ILIHNTIFGEQVFLPLEHSQFSRYRWRGPTAAFLSLVDQKRSLLSVFRANQYPDLRRVELAITGQ
APFRTLVQRIWTTTSDGEAVELASETLMKRELTSFVVLVPQGTPDVQLRLTRPQLTKVLNATGAD
SATPTTFELGVNGKFSVFTTSTCVTFAAREEGVSSSTSTQVQILSNALTKAGQAAANAKTVYGEN
THRTFSVVVDDCSMRAVLRRLQVGGGTLKFFLTTPVPSLCVTATGPNAVSAVFLLKPQK
>1EE8B
PELPEVETTRRRLRPLVLGQTLRQVVHRDPARYRNTALAEGRRILEVDRRGKFLLFALEGGVELV

AHLGMTGGFRLEPTPHTRAALVLEGRTLYFHDPRRFGRLFGVRRGDYREIPLLLRLGPEPLSEAF
AFPGFFRGLKESARPLKALLLDQRLAAGVGNIYADEALFRARLSPFRPARSLTEEEARRLYRALR
EVLAEAVELGGSTLSDQSYRQPDGLPGGFQTRHAVYGREGLPCPACGRPVERRVVAGRGTHFCPT
CQGEGP
>1EIJA
MRQQLEMQKKQIMMQILTPEARSRLANLRLTRPDFVEQIELQLIQLAQMGRVRSKITDEQLKELL
KRVAGKKREIKISRK
>1F1EA
MAVELPKAAIERIFRQGIGERRLSQDAKDTIYDFVPTMAEYVANAAKSVLDASGKKTLMEEHLKA
LADVLMVEGVEDYDGELFGRATVRRILKRAGIERASSDAVDLYNKLICRATEELGEKAAEYADED
GRKTVQGEDVEKAITYSMPKGGEL
>1F2RI
MELSRGASAPDPDDVRPLKPCLLRRNHSRDQHGVAASSLEELRSKACELLAIDKSLTPITLVLAE
DGTIVDDDDYFLCLPSNTKFVALACNEKWTYNDSD
>1F6VA
GSRIAKRTAINKTKKADVKAIADAWQINGEKELELLQQIAQKPGALRILNHSLRLAAMTAHGKGE
RVNEDYLRQAFRELDLDVDISTLLRN
>1G5HA
WLSGYAGPADGTQQPDAPEHAVAREALVDLCRRRHFLSGTPQQLSTAALLSGCHARFGPLGVELR
KNLASQWWSSMVVFREQVFAVDSLHQEPGSSQPRDSAFRLVSPESIREILQDREPSKEQLVAFLE
NLLKTSGKLRATLLHGALEHYVNCLDLVNRKLPFGLAQIGVCFHPVSNSNQTPSSVTRVGEKTEA
SLVWFTPTRTSSQWLDFWLRHRLLWWRKFAMSPSNFSSADCQDELGRKGSKLYYSFPWGKEPIET
LWNLGDQELLHTYPGNVSTIQGRDGRKNVVPCVLSVSGDVDLGTLAYLYDSFQLAENSFARKKSL
QRKVLKLHPCLAPIKVALDVGKGPTVELRQVCQGLLNELLENGISVWPGYSETVHSSLEQLHSKY
DEMSVLFSVLVTETTLENGLIQLRSRDTTMKEMMHISKLRDFLVKYLASASNVAAALDHHHHHH
>1GDTB
MRLFGYARVSTSQQSLDIQVRALKDAGVKANRIFTDKASGSSSDRKGLDLLRMKVEEGDVILVKK
LDRLGRDTADMIQLIKEFDAQGVSIRFIDDGISTDGEMGKMVVTILSAVAQAERQRILERTNEGR
QEAMAKGVVFGRKRKIDRDAVLNMWQQGLGASHISKTMNIARSTVYKVINESN
>1HCRA
GRPRAINKHEQEQISRLLEKGHPRQQLAIIFGIGVSTLYRYFPASSIKKRMN
>1HKQA
MVDNKVTQSNKLIESSHTLTLNEKRLVLCAASLIDSRKPLPKDGYLTIRADTFAEVFGIDVKHAY
AALDDAATKLFNRDIRRYVKGKVVERMRWVFHVKYREGQGCVELGFSPTIIPHLTMLHKEFTSYQ
LK
>1HLVA
MGPKRRQLTFREKSRIIQEVEENPDLRKGEIARRFNIPPSTLSTILKNKRAILASERKYGVASTC
RKTNKLSPYDKLEGLLIAWFQQIRAAGLPVKGIILKEKALRIAEELGMDDFTASNGWLDRFRRRR
S
>1HQ3H
MSGRGKGGKGLGKGGAKRHRKVLRDNIQGITKPAIRRLARRGGVKRISGLIYEETRGVLKVFLEN
VIRDAVTYTEHAKRKTVTAMDVVYALKRQGRTLYGFGG
>1I11A
GSPHIKRPMNAFMVWAKDERRKILQAFPDMHNSNISKILGSRWKAMTNLEKQPYYEEQARLSKQH

LEKYPDYKYKPRPKRT
>1IGNB
GALPSHNKASFTDEEDEFILDVVRKNPTRRTTHTLYDEISHYVPNHTGNSIRHRFRVYLSKRLEY
VYEVDKFGKLVRDDDGNLIKTKVLPPSIKRKFSADEDYTLAIAVKKQFYRDLFQIDPDTGRSLIT
DEDTPTAIARRNMTMDPNHVPGSEPNFAAYRTQSRRGPIAREFFKHFAEEHAAHTENAWRDRFRK
FLLAYGIDDYISYYEAEKAQNREPEPMKNLTNRPKRPGVPTPGNYNSAAKR
>1IN4A
MSEFLTPERTVYDSGVQFLRPKSLDEFIGQENVKKKLSLALEAAKMRGEVLDHVLLAGPPGLGKT
TLAHIIASELQTNIHVTSGPVLVKQGDMAAILTSLERGDVLFIDEIHRLNKAVEELLYSAIEDFQ
IDIMIGKGPSAKSIRIDIQPFTLVGATTRSGLLSSPLRSRFGIILELDFYTVKELKEIIKRAASL
MDVEIEDAAAEMIAKRSRGTPRIAIRLTKRVRDMLTVVKADRINTDIVLKTMEVLNIDDEGLDEF
DRKILKTIIEIYRGGPVGLNALAASLGVEADTLSEVYEPYLLQAGFLARTPRGRIVTEKAYKHLK
YEVPENRLF
>1IRZA
TAQKKPRVLWTHELHNKFLAAVDHLGVERAVPKKILDLMNVDKLTRENVASHLQKFRVALKKVS
>1IUFA
GIHMGKIKRRAITEHEKRALRHYFFQLQNRSGQQDLIEWFREKFGKDISQPSVSQILSSKYSYLD
NTVEKPWDVKRNRPPKYPLLEAALFEWQVQQGDDATLSGETIKRAAAILWHKIPEYQDQPVPNFS
NGWLEGFRKRHILH
>1IV6A
MTPEKHRARKRQAWLWEEDKNLRSGVRKYGEGNWSKILLHYKFNNRTSVMLKDRWRTMKKLKLIS
SDSED
>1IXCB
MEFRQLKYFIAVAEAGNMAAAAKRLHVSQPPITRQMQALEADLGVVLLERSHRGIELTAAGHAFL
EDARRILELAGRSGDRSRAAARGDVGELSVAYFGTPIYRSLPLLLRAFLTSTPTATVSLTHMTKD
EQVEGLLAGTIHVGFSRFFPRHPGIEIVNIAQEDLYLAVHRSQSGKFGKTCKLADLRAVELTLFP
RGGRPSFADEVIGLFKHAGIEPRIARVVEDATAALALTMAGAASSIVPASVAAIRWPDIAFARIV
GTRVKVPISCIFRKEKQPPILARFVEHVRRSAKD
>1IYMA
AMDDGVECAVCLAELEDGEEARFLPRCGHGFHAECVDMWLGSHSTCPLCRLTVVV
>1J2FB
GAMGSSLDNPTPFPNLGPSENPLKRLLVPGEEWEFEVTAFYRGRQVFQQTISCPEGLRLVGSEVG
DRTLPGWPVTLPDPGMSLTDRGVMSYVRHVLSCLGGGLALWRAGQWLWAQRLGHCHTYWAVSEEL
LPNSGHGPDGEVPKDKEGGVFDLGPFIVDLITFTEGSGRSPRYALWFCVGESWPQDQPWTKRLVM
VKVVPTCLRALVEMARVGGASSLENTVDLHISNSHPLSLTSDQYKAYLQDLVEGMDFQGPGES
>1JE5B
MAKKIFTSALGTAEPYAYIAKPDYGNEERGFGNPRGVYKVDLTIPNKDPRCQRMVDEIVKCHEEA
YAAAVEEYEANPPAVARGKKPLKPYEGDMPFFDNGDGTTTFKFKCYASFQDKKTKETKHINLVVV
DSKGKKMEDVPIIGGGSKLKVKYSLVPYKWNTAVGASVKLQLESVMLVELATFGGGEDDWADEVE
ENGYVASGSAK
>1JEQB
MVRSGNKAAVVLCMDVGFTMSNSIPGIESPFEQAKKVITMFVQRQVFAENKDEIALVLFGTDGTD
NPLSGGDQYQNITVHRHLMLPDFDLLEDIESKIQPGSQQADFLDALIVSMDVIQHETIGKKFEKR
HIEIFTDLSSRFSKSQLDIIIHSLKKCDISLQFFLPFSLGKEDGSGDRGDGPFRLGGHGPSFPLK

GITEQQKEGLEIVKMVMISLEGEDGLDEIYSFSESLRKLCVFKKIERHSIHWPCRLTIGSNLSIR
IAAYKSILQERVKKTWTVVDAKTLKKEDIQKETVYCLNDDDETEVLKEDIIQGFRYGSDIVPFSK
VDEEQMKYKSEGKCFSVLGFCKSSQVQRRFFMGNQVLKVFAARDDEAAAVALSSLIHALDDLDMV
AIVRYAYDKRANPQVGVAFPHIKHNYECLVYVQLPFMEDLRQYMFSSLKNSKKYAPTEAQLNAVD
ALIDSMSLAKKDEKTDTLEDLFPTTKIPNPRFQRLFQCLLHRALHPREPLPPIQQHIWNMLNPPA
EVTTKSQIPLSKIKTLFPLIEAKKKDQVTAQEIFQDNHEDGPTAK
>1JEQA
MSGWESYYKTEGDEEAEEEQEENLEASGDYKYSGRDSLIFLVDASKAMFESQSEDELTPFDMSIQ
CIQSVYISKIISSDRDLLAVVFYGTEKDKNSVNFKNIYVLQELDNPGAKRILELDQFKGQQGQKR
FQDMMGHGSDYSLSEVLWVCANLFSDVQFKMSHKRIMLFTNEDNPHGNDSAKASRARTKAGDLRD
TGIFLDLMHLKKPGGFDISLFYRDIISIAEDEDLRVHFEESSKLEDLLRKVRAKETRKRALSRLK
LKLNKDIVISVGIYNLVQKALKPPPIKLYRETNEPVKTKTRTFNTSTGGLLLPSDTKRSQIYGSR
QIILEKEETEELKRFDDPGLMLMGFKPLVLLKKHHYLRPSLFVYPEESLVIGSSTLFSALLIKCL
EKEVAALCRYTPRRNIPPYFVALVPQEEELDDQKIQVTPPGFQLVFLPFADDKRKMPFTEKIMAT
PEQVGKMKAIVEKLRFTYRSDSFENPVLQQHFRNLEALALDLMEPEQAVDLTLPKVEAMNKRLGS
LVDEFKELVYPPDYNPEGKVTKRKHDNEGSGSKRPKVEYSEEELKTHISKGTLGKFTVPMLKEAC
RAYGLKSGLKKQELLEALTKHFQD
>1JSPB
GSHMRKKIFKPEELRQALMPTLEALYRQDPESLPFRQPVDPQLLGIPDYFDIVKNPMDLSTIKRK
LDTGQYQEPWQYVDDVWLMFNNAWLYNRKTSRVYKFCSKLAEVFEQEIDPVMQSLG
>1K99A
MKKLKKHPDFPKKPLTPYFRFFMEKRAKYAKLHPEMSNLDLTKILSKKYKELPEKKKMKYIQDFQ
REKQEFERNLARFREDHPDLIQNAKKLEHHHHHH
>1KFTA
MGSSHHHHHHSSGLVPRGSHMNTSSLETIEGVGPKRRQMLLKYMGGLQGLRNASVEEIAKVPGIS
QGLAEKIFWSLKH
>1KIXA
MSTAAKQNRSTSRVSKKKTAAPKEGAAKKSDKGHKYEYVELAKASLTSAQPQHFYAVVIDATFPY
KTNQERYICSLKIVDPTLYLKQQKGAGDASDYATLVLYAKRFEDLPIIHRAGDIIRVHRATLRLY
NGQRQFNANVFYSSSWALFSTDKRSVTQEINNQDAVSDTTPFSFSSKHATIEKNEISILQNLRKW
ANQYFSSYSVISSDMYTALNKAQAQKGDFDVVAKILQVHELDEYTNELKLKDASGQVFYTLSLKL
KFPHVRTGEVVRIRSATYDETSTQKKVLILSHYSNIITFIQSSKLAKELRAKIQDDHSVEVASLK
KNVSLNAVVLTEVDKKHAALPSTSLQDLFHHADSDKELQAQDTFRTQFYVTKIEPSDVKEWVKGY
DRKTKKSSSLKGASGKGDNIFQVQFLVKDASTQLNNNTYRVLLYTQDGLGANFFNVKADNLHKNA
DARKKLEDSAELLTKFNSYVDAVVERRNGFYLIKDTKLIY
>1KKXA
MRGSGSHHHHHHGSNNKQYELFMKSLIENCKKRNMPLQSIPEIGNRKINLFYLYMLVQKFGGADQ
VTRTQQWSMVAQRLQISDYQQLESIYFRILLPYERHMISQEGIKETQAKRILQPSLIS
>1KN0A
MSGTEEAILGGRDSHPAAGGGSVLCFGQCQYTAEEYQAIQKALRQRLGPEYISSRMAGGGQKVCY
IEGHRVINLANEMFGYNGWAHSITQQNVDFVDLNNGKFYVGVCAFVRVQLKDGSYHEDVGYGVSE
GLKSKALSLEKARKEAVTDGLKRALRSFGNALGNCILDKDYLRSLNKLPRQLPLEVDLTKAKRQD
LEPSVEEARYNSCRPNM
>1KNUB

LNPESADLRALAKHLYDSYIKSFPLTKAKARAILTGKTTDKSPFVIYDMNSLMMGEDKIKFKHIT
PLQEQSKEVAIRIFQGCQFRSVEAVQEITEYAKSIPGFVNLDLNDQVTLLKYGVHEIIYTMLASL
MNKDGVLISEGQGFMTREFLKSLRKPFGDFMEPKFEFAVKFNALELDDSDLAIFIAVIILSGDRP
GLLNVKPIEDIQDNLLQALELQLKLNHPESSQLFAKLLQKMTDLRQIVTEHVQLLQVIKKTETDM
SLHPLLQEIYKDLY

>1KU9B

MIIMEEAKKLIIELFSELAKIHGLNKSVGAVYAILYLSDKPLTISDIMEELKISKGNVSMSLKKL
EELGFVRKVWIKGERKNYYEAVDGFSSIKDIAKRKHDLIAKTYEDLKKLEEKCNEEEKEFIKQKI
KGIERMKKISEKILEALNDLDN

>1KW4A

METKRVNGTDRPPISSWSVDDVSNFIRELPGCQDYVDDFIQQEIDGQALLRLKEKHLVNAMGMKL
GPALKIVAKVESIKEVRDHHHHHH

>1KZYD

ALEEQRGPLPLNKTLFLGYAFLLTMATTSDKLASRSKLPDGPTGSSEEEEEFLEIPPFNKQYTES
QLRAGAGYILEDFNEAQCNTAYQCLLIADQHCRTRKYFLCLASGIPCVSHVWVHDSCHANQLQNY
RNYLLPAGYSLEEQRILDWQPRENPFQNLKVLLVSDQQQNFLELWSEILMTGGAASVKQHHSSAH
NKDIALGVFDVVVTDPSCPASVLKCAEALQLPVVSQEWVIQCLIVGERIGFKQHPKYKHDYVSH

>1L3AD

MASMTGGQQMGRGSDYFEPQQQQQQQQQPQGASTPKVFVGYSIYKGKAALTVEPRSPEFSPLDS
GAFKLSREGMVMLQFAPAAGVRQYDWSRKQVFSLSVTEIGSIISLGTKDSCEFFHDPNKGRSDEG
RVRKVLKVEPLPDGSGHFFNLSVQNKLINLDENIYIPVTKAEFAVLVSAFNFVMPYLLGWHTAVN
SFKPEDASRSNNANPRSGAELEWNLEHHHHHH

>1L8YA

MGKLPESPKRAEEIWQQSVIGDYLARFKNDRVKALKAMEMTWNNMEKKEKLMWIKKAAEDQKRYE
RELSEMRAPPAATNSSKKLEHHHHHH

>1MH3A

KIEEGKLVIWINGDKGYNGLAEVGKKFEKDTGIKVTVEHPDKLEEKFPQVAATGDGPDIIFWAHD
RFGGYAQSGLLAEITPDKAFQDKLYPFTWDAVRYNGKLIAYPIAVEALSLIYNKDLLPNPPKTWE
EIPALDKELKAKGKSALMFNLQEPYFTWPLIAADGGYAFKYENGKYDIKDVGVDNAGAKAGLTFL
VDLIKNKHMNADTDYSIAEAAFNKGETAMTINGPWAWSNIDTSKVNYGVTVLPTFKGQPSKPFVG
VLSAGINAASPNKELAKEFLENYLLTDEGLEAVNKDKPLGAVALKSYEEELAKDPRIAATMENAQ
KGEIMPNIPQMSAFWYAVRTAVINAASGRQTVDAALAAAQTAAAAAISPQARAFLEQVFRRKQSL
NSKEKEEVAKKCGITPLQVRVWFINKRMRSK

>1MOJA

MSTQKNARATAGEVEGSDALRMDADRAEQCVDALNADLANVYVLYHQLKKHHWNVEGAEFRDLHL
FLGEAAETAEEVADELAERVQALGGVPHASPETLQAEASVDVEDEDVYDIRTSLANDMAIYGDII
EATREHTELAENLGDHATAHMLREGLIELEDDAHHIEHYLEDDTLVTQGALE

>1MP9B

YIIPDEIPYKAVVNIENIVATVTLDQTLDLYAMERSVPNVEYDPDQFPGLIFRLESPKITSLIFK
SGKMVVTGAKSTDELIKAVKRIIKTLKKYGMQLTGKPKIQIQNIVASANLHVIVNLDKAAFLLEN
NMYEPEQFPGLIYRMDEPRVVLLIFSSGKMVITGAKREDEVHKAVKKIFDKLVELDCVKPVEEEE
LEF

>1MSZA

MGSLNGGSPEGVESQDGVDHFRAMIVEFMASKKMQLEFPPSLNSHDRLRVHQIAEEHGLRHDSSG

EGKRRFITVSKRAGSHHHHHH
>1N1JB
GSHMEEIRNLTVKDFRVQELPLARIKKIMKLDEDVKMISAEAPVLFAKAAQIFITELTLRAWIHT
EDNKRRTLQRNDIAMAITKFDQFDFLIDIVPR
>1N1JA
SFREQDIYLPIANVARIMKNAIPQTGKIAKDAKECVQECVSEFISFITSEASERCHQEKRKTING
EDILFAMSTLGFDSYVEPLKLYLQKFRE
>1NGNA
ALSPPRRKSFKKWTPPRSPFNLVQEILFHDPWKLLIATIFLNRTSGKMAIPVLWEFLEKYPSAEV
ARAADWRDVSELLKPLGLYDLRAKTIIKFSDEYLTKQWRYPIELHGIGKYGNDSYRIFCVNEWKQ
VHPEDHKLNKYHDWLWENHEKLSLS
>1NK2P
ASDGLPNKKRKRRVLFTKAQTYELERRFRQQRYLSAPEREHLASLIRLTPTQVKIWFQNHRYKTK
RAQNEKGYEGHP
>1NZPA
MAQPSSQKATNHNLHITEKLEVLAKAYSVQGDKWRALGYAKAINALKSFHKPVTSYQEACSIPGI
GKRMAEKIIEILESGHLRKLDH
>1O57A
MKFRRSGRLVDLTNYLLTHPHELIPLTFFSERYESAKSSISEDLTIIKQTFEQQGIGTLLTVPGA
AGGVKYIPKMKQAEAEEFVQTLGQSLANPERILPGGYVYLTDILGKPSVLSKVGKLFASVFAERE
IDVVMTVATKGIPLAYAAASYLNVPVVIVRKDNKVTEGSTVSINYVSGSSNRIQTMSLAKRSMKT
GSNVLIIDDFMKAGGTINGMINLLDEFNANVAGIGVLVEAEGVDERLVDEYMSLLTLSTINMKEK
SIEIQNGNFLRFFKDNLLKNGETESHHHHHH
>1OQJB
GAMEDMEIAYPITCGESKAILLWKKFVCPGINVKCVKFNDQLISPKHFVHLAGKSTLKDWKRAIR
LGGIMLRKMMDSGQIDFYQHDKVCSNTCRSTK
>1OSVB
AELTVDQQTLLDYIMDSYSKQRMPQEITNKILKEEFSAEENFLILTEMATSHVQILVEFTKRLPG
FQTLDHEDQIALLKGSAVEAMFLRSAEIFNKKLPAGHADLLEERIRKSGISDEYITPMFSFYKSV
GELKMTQEEYALLTAIVILSPDRQYIKDREAVEKLQEPLLDVLQKLCKIYQPENPQHFACLLGRL
TELRTFNHHHAEMLMSWRVNDHKFTPLLCEIWDVQ
>1OY3D
VFGYVTEDGDTALHLAVIHQHEPFLDFLLGFSAGHEYLDLQNDLGQTALHLAAILGEASTVEKLY
AAGAGVLVAERGGHTALHLACRVRAHTCACVLLQPRPSHPRDASDTYLTQSQDCTPDTSHAPAAV
DSQPNPENEEEPRDEDWRLQLEAENYDGHTPLHVAVIHKDAEMVRLLRDAGADLNKPEPTCGRTP
LHLAVEAQAASVLELLLKAGADPTARMYGGRTPLGSALLRPNPILARLLRAHGAPEPEDGGDKLS
PCSSSGSDSDSDNRDEGDEYDD
>1OY3B
TAELKICRVNRNSGSCLGGDEIFLLCDKVQKEDIEVYFTGPGWEARGSFSQADVHRQVAIVFRTP
PYADPSLQAPVRVSMQLRRPSDRELSEPMEFQYLPDTDDRHRIEEKRKRTYETFKSIMKKSPFNG
PTEPRP
>1P1AA
GSHMQVTLKTLQQQTFKIDIDPEETVKALKEKIESEKGKDAFPVAGQKLIYAGKILNDDTALKEY
KIDEKNFVVVMVTKPKAVST

>1P4EC

SQFDILCKTPPKVLVRQFVERFERPSGEKIASCAAELTYLCWMITHNGTAIKRATFMSYNTIISN
SLSFDIVNKSLQFKYKTQKATILEASLKKLIPAWEFTIIPYNGQKHQSDITDIVSSLQLQFESSE
EADKGNSHSKKMLKALLSEGESIWEITEKILNSFEYTSRFTKTKTLYQFLFLATFINCGRFSDIK
NVDPKSFKLVQNKYLGVIIQCLVTETKTSVSRHIYFFSARGRIDPLVYLDEFLRNSEPVLKRVNR
TGNSSSNKQEYQLLKDNLVRSYNKALKKNAPYPIFAIKNGPKSHIGRHLMTSFLSMKGLTELTNV
VGNFSDKRASAVARTTYTHQITAIPDHYFALVSRYYAYDPISKEMIALKDETNPIEEWQHIEQLK
GSAEGSIRYPAWNGIISQEVLDYLSSYINRRIGHHHHHH

>1P4WA

MRGSHHHHHHGSYTPESVAKLLEKISAGGYGDKRLSPKESEVLRLFAEGFLVTEIAKKLNRSIKT
ISSQKKSAMMKLGVDNDIALLNYLSSVSMTPVDK

>1P92A

MKDLVDTTEMYLRTIYELEEEGVTPLRARIAERLEQSGPTVSQTVARMERDGLVVVASDRSLQMT
PTGRTLATAVMRKARLAERLLTDIIGLDINKVHDEACRWEHVMSDEVERRLVKVLKDVSRSPFGN
PIPGLDELGVGNSDAAAPGTRVIDAATSMPRKVRIVQINEIFQVETDQFTQLLDADIRVGSEVEI
VDRDGHITLSHNGKDVELLDDLAHTIRIEEL

>1PGZA

SKSESPKEPEQLRKLFIGGLSFETTDESLRSHFEQWGTLTDCVVMRDPNTKRSRGFGFVTYATVE
EVDAAMNARPHKVDGRVVEPKRAVSREDSQRPGAHLTVKKIFVGGIKEDTEEHHLRDYFEQYGKI
EVIEIMTDRGSGKKRGFAFVTFDDHDSVDKIVIQKYHTVNGHNCEVRKALSKQEMASASSSQRGR

>1PH1B

PQQQSAFKQLYTELFNNEGDFSKVSSNLKKPLKCYVKESYPHFLVTDGYFFVAPYFTKEAVNEFH
AKFPNVNIVDLTDKVIVINNWSLELRRVNSAEVFTSYANLEARLIVHSFKPNLQERLNPTRYPVN
LFRDDEFKTTIQHFRHTALQAAINKTVKGDNLVDISKVADAAGKKGKVDAGIVKASASKGDEFSD
FSFKEGNTATLKIADIFVQEKG

>1PL5S

SNTTEILTSVDVLGTHSQTGTQQSNMYTSTQKTELEIDNKDSVTECSKDMKEDGLSFVDIVLSKA
ASALDEKEKQLAVANEIIRSLSDEVMRNEIRITSLQGDLTFTKKCLENARSQISEKDAKINKLME
KDFQVNKEIKPY

>1POGA

RGSHMRRRKKRTSIETNIRVALEKSFLENQKPTSEEITMIADQLNMEKEVIRVWFCNRRQKEKRI
DI

>1PVEA

GSHMPLEFLRNQPQFQQMRQIIQQNPSLLPALLQQIGRENPQLLQQISQHQEHFIQMLNEPVQEA
GGQGGGG

>1Q1VA

DEPLIKKLKKPPTDEELKETIKKLLASANLEEVTMKQICKKVYENYPTYDLTERKDFIKTTVKEL
ISLEH

>1Q87B

PVNTKRSNGTKRVEFPTTKKSMCIGNSTPNEQETFRAKVDEIWFRLTQKTDGTVMRDFLIEKAAE
YFKQPEQPKQNAIEVISAIMAPQEEQTKSKADLYKFLAMFGPYETIMLKIASLLLISNNKGHWLT
FDPQAEKNANNQRDSISGWFDQNEPNCLILKTPTGIRKIWNKPLIEATGQYLMDENGEKYDSWDK
YFEMKPIETYLTAYPTFAPMHHHHHH

>1QZGB

GPGGEDVIDSLQLNELLNAGEYKIGELTFQSIRSSQELQKKNTIVNLFGIVKDFTPSRQSLHGTK
DWVTTVYLWDPTCDTSSIGLQIHLFSKQGNDLPVIKQVGQPLLLHQITLRSYRDRTQGLSKDQFR
YALWPDFSSNSKDTLCPQPMPRLMKTGDKEEQFALLLNKIWDEQTNKHKNGELLSTS
>1QZQB
MEEYMPTEHHHHHHENLYFQGTSGEGQDIWDMLDKGNPFQFYLTRVSGVKPKYNSGALHIKDILS
PLFGTLVSSAQFNYCFDVDWLVKQYPPEFRKKPILLVHGDKREAKAHLHAQAKPYENISLCQAKL
DIAFGTHHTKMMLLLYEEGLRVVIHTSNLIHADWHQKTQGIWLSPLYPRIADGTHKSGESPTHFK
ADLISYLMAYNAPSLKEWIDVIHKHDLSETNVYLIGSTPGRFQGSQKDNWGHFRLKKLLKDHASS
MPNAESWPVVGQFSSVGSLGADESKWLCSEFKESMLTLGKESKTPGKSSVPLYLIYPSVENVRTS
LEGYPAGGSLPYSIQTAEKQNWLHSYFHKWSAETSGRSNAMPHIKTYMRPSPDFSKIAWFLVTSA
NLSKAAWGALEKNGTQLMIRSYELGVLFLPSAFGLDSFKVKQKFFAGSQEPMATFPVPYDLPPEL
YGSKDRPWIWNIPYVKAPDTHGNMWVPS
>1R5KC
MDPMIKRSKKNSLALSLTADQMVSALLDAEPPILYSEYDPTRPFSEASMMGLLTNLADRELVHMI
NWAKRVPGFVDLTLHDQVHLLECAWLEILMIGLVWRSMEHPGKLLFAPNLLLDRNQGKCVEGMVE
IFDMLLATSSRFRMMNLQGEEFVCLKSIILLNSGVYTFLSSTLKSLEEKDHIHRVLDKITDTLIH
LMAKAGLTLQQQHQRLAQLLLILSHIRHMSNKGMEHLYSMKCKNVVPLYDLLLEMLDAHRLHAPT
S
>1RH6B
MYLTLQEWNARQRRPRSLETVRRWVRESRIFPPPVKDGREYLFHESAVKVDLNRP
>1RI7A
MGSSHHHHHHSSGLVPRGSHMRVPLDEIDKKIIKILQNDGKAPLREISKITGLAESTIHERIRKL
RESGVIKKFTAIIDPEALGYSMLAFILVKVKAGKYSEVASNLAKYPEIVEVYETTGDYDMVVKIR
TKNSEELNNFLDLIGSIPGVEGTHTMIVLKTHKETTELPIK
>1RIFB
MDIKVHFHDFSHVRIDCEESTFHELRDFFSFEADGYRFNPRFRYGNWDGRIRLLDYNRLLPFGLV
GQIKKFCDNFGYKAWIDPQINEKEELSRKDFDEWLSKLEIYSGNKRIEPHWYQKDAVFEGLVNRR
RILNLPTSAGRSLIQALLARYYLENYEGKILIIVPTTALTTQMADDFVDYRLFSHAMIKKIGGGA
SKDDKYKNDAPVVVGTWQTVVKQPKEWFSQFGMMMNDECHLATGKSISSIISGLNNCMFKFGLSG
SLRDGKANIMQYVGMFGEIFKP
>1RW2A
MHHHHHHKLKTEQGGAHFSVSSLAEGSVTSVGSVNPAENFRVLVKQKKASFEEASNQLINHIEQF
LDTNETPYFMKSIDCIRAFREEAIKFSEEQRFNNFLKALQEKVEIKQLNHFWEIVVQDGITLITK
EEASGSSVTAEEAKKFLAPKDK
>1S6MA
MLSHMVLTRQDIGRAASYYEDGADDYYAKDGDASEWQGKGAEELGLSGEVDSKRFRELLAGNIGE
GHRIMRSATRQDSKERIGLDLTFSAPKSVSLQALVAGDAEIIKAHDRAVARTLEQAEARAQARQK
IQGKTRIETTGNLVIGKFRHETSRERDPQLHTHAVILNMTKRSDGQWRALKNDEIVKATRYLGAV
YNAELAHELQKLGYQLRYGKDGNFDLAHIDRQQIEGFSKRTEQIAEWYAARGLDPNSVSLEQKQA
AKVLSRAKKTSVDREALRAEWQATAKELGIDFS
>1SD4B
MTNKQVEISMAEWDVMNIIWDKKSVSANEIVVEIQKYKEVSDKTIRTLITRLYKKEIIKRYKSEN
IYFYSSNIKEDDIKMKTAKTFLNKLYGGDMKSLVLNFAKNEELNNKEIEELRDILNDISKK
>1SE8A

MARGMNHVYLIGALARDPELRYTGNGMAVFEATVAGEDRVIGNDGRERNLPWYHRVSILGKPAEW
QAERNLKGGDAVVVEGTLEYRQWEAPEGGKRSAVNVKALRMEQLGTQPELIQDAGGGVRMSGAMN
EVLVLGNVTRDPEIRYTPAGDAVLSLSIAVNENYQDRQGQRQEKVHYIDATLWRDLAENMKELRK
GDPVMIMGRLVNEGWTDKDGNKRNSTRVEATRVEALARGAGNANSGYAAATPAAPRTQTASSAAR
PTSGGYQSQPSRAANTGSRSGGLDIDQGLDDFPPEEDDLPF
>1SFUB
MDLLSCTVNDAEIFSLVKKEVLSLNTNDYTTAISLSNRLKINKKKINQQLYKLQKEDTVKMVPSN
PPKWFKNYNC
>1SQ8A
MLMGERIRARRIQLGLNQAELAQKVGVDQQAIEQLENGKAKRPRFLPELARALGVAVDWLLNGA
>1T0FC
GSAIKVVKPSDWDSLPDTDLRYIYSQRQPEKTMHERLKGKGVIVDMASLFKQAG
>1T0FA
GSAMAKANSSFSEVQIARRIKEGRGQGHGKDYIPWLTVQEVPSSGRSHRIYSHKTGRVHHLLSDL
ELAVFLSLEWESSVLDIREQFPLLPSDTRQIAIDSGIKHPVIRGVDQVMSTDFLVDCKDGPFEQF
AIQVKPAAALQDERTLEKLELERRYWQQKQIPWFIFTDKEINPVVKENIEWLYSVKTEEVSAELL
AQLSPLAHILQEKGDENIINVCKQVDIAYDLELGKTLSEIRALTANGFIKFNIYKSFRANKCADL
CISQVVNMEELRYVAN
>1T23A
SNTRNFVLRDEDGNEHGVFTGKQPRQAALKAANRGSGTKANPDIIRLRERGTKKVHVFKAWKEIV
DAPKNRPAWMPEKISKPFVKKERIEKLE
>1U2WD
MKKKDTCEIFGYDEEKVNRIQGDLQTVDISGVSQILKAIADENRAKITYALCQDEELCVCDIANI
LGVTIANASHHLRTLYKQGVVNFRKEGKLALYSLGDEHIRQIMMIALAHKKEVKVNV
>1U3EM
MEWKDIKGYEGHYQVSNTGEVYSIKSGKTLKHQIPKDGYHRIGLFKGGKGKTFQVHRLVAIHFCE
GYEEGLVVDHKDGNKDNNLSTNLRWVTQKINVENQMSRGTLNVSKAQQIAKIKNQKPIIVISPDG
IEKEYPSTKCACEELGLTRGKVTDVLKGHRIHHKGYTFRYKLNG
>1U78A
MPRGSALSDTERAQLDVMKLLNVSLHEMSRKISRSRHCIRVYLKDPVSYGTSKRAPRRKALSVRD
ERNVIRAASNSCKTARDIRNELQLSASKRTILNVIKRSGVIVRQKLRPAPLLSADHKLKRLEFAK
NNMGTHHHHHH
>1U9NA
MGSSHHHHHHSSGLVPRGSHVTTSAASQASLPRGRRTARPSGDDRELAILATAENLLEDRPLADI
SVDDLAKGAGISRPTFYFYFPSKEAVLLTLLDRVVNQADMALQTLAENPADTDRENMWRTGINVF
FETFGSHKAVTRAGQAARATSVEVAELWSTFMQKWIAYTAAVIDAERDRGAAPRTLPAHELATAL
NLMNERTLFASFAGEQPSVPEARVLDTLVHIWVTSIYGENR
>1UB4C
GPHMIHSSVKRWGNSPAVRIPATLMQALNLNIDDEVKIDLVDGKLIIEPVRKEPVFTLAELVNDI
TPENLHENIDWGEPKDKEVW
>1UB4B
VSRYVPDMGDLIWVDFDPTKGSEQAGHRPAVVLSPFMYNNKTGMCLCVPCTTQSKGYPFEVVLSG
QERDGVALADQVKSIAWRARGATKKGTVAPEELQLIKAKINVLIG
>1UDVB

MTEKLNEIVVRKTKNVEDHVLDVIVLFNQGIDEVILKGTGREISKAVDVYNSLKDRLGDGVQLVN
VQTGSEVRDRRRISYILLRLKRVY
>1UFID
GSHMPVPSFGEAMAYFAMVKRYLTSFPIDDRVQSHILHLEHDLVHVTRKNHARQAGVRGLGHQS
>1UKLF
RSSINDKIIELKDLVMGTDAKMHKSGVLRKAIDYIKYLQQVNHKLRQENMVLKLANQKNKL
>1UKLB
MELITILEKTVSPDRLELEAAQKFLERAAVENLPTFLVELSRVLANPGNSQVARVAAGLQIKNSL
TSKDPDIKAQYQQRWLAIDANARREVKNYVLQTLGTETYRPSSASQCVAGIACAEIPVSQWPELI
PQLVANVTNPNSTEHMKESTLEAIGYICQDIDPEQLQDKSNEILTAIIQGMRKEEPSNNVKLAAT
NALLNSLEFTKANFDKESERHFIMQVVCEATQCPDTRVRVAALQNLVKIMSLYYQYMETYMGPAL
FAITIEAMKSDIDEVALQGIEFWSNVCDEEMDLAIEASEAAEQGRPPEHTSKFYAKGALQYLVPI
LTQTLTKQDENDDDDDWNPCKAAGVCLMLLSTCCEDDIVPHVLPFIKEHIKNPDWRYRDAAVMAF
GSILEGPEPNQLKPLVIQAMPTLIELMKDPSVVVRDTTAWTVGRICELLPEAAINDVYLAPLLQC
LIEGLSAEPRVASNVCWAFSSLAEAAYEAADVADDQEEPATYCLSSSFELIVQKLLETTDRPDGH
QNNLRSSAYESLMEIVKNSAKDCYPAVQKTTLVIMERLQQVLQMESHIQSTSDRIQFNDLQSLLC
ATLQNVLRKVQHQDALQISDVVMASLLRMFQSTAGSGGVQEDALMAVSTLVEVLGGEFLKYMEAF
KPFLGIGLKNYAEYQVCLAAVGLVGDLCRALQSNILPFCDEVMQLLLENLGNENVHRSVKPQILS
VFGDIALAIGGEFKKYLEVVLNTLQQASQAQVDKSDFDMVDYLNELRESCLEAYTGIVQGLKGDQ
ENVHPDVMLVQPRVEFILSFIDHIAGDEDHTDGVVACAAGLIGDLCTAFGKDVLKLVEARPMIHE
LLTEGRRSKTNKAKTLATWATKELRKLKNQA
>1UL1Z
GIQGLAKLIADVAPSAIRENDIKSYFGRKVAIDASMSIYQFLIAVRQGGDVLQNEEGETTSHLMG
MFYRTIRMMENGIKPVYVFDGKPPQLKSGELAKRSERRAEAEKQLQQAQAAGAEQEVEKFTKRLV
KVTKQHNDECKHLLSLMGIPYLDAPSEAEASCAALVKAGKVYAAATEDMDCLTFGSPVLMRHLTA
SEAKKLPIQEFHLSRILQELGLNQEQFVDLCILLGSDYCESIRGIGPKRAVDLIQKHKSIEEIVR
RLDPNKYPVPENWLHKEAHQLFLEPEVLDPESVELKWSEPNEEELIKFMCGEKQFSEERIRSGVK
RLSKSRQGSTQGRLDDFFKVTGSLSSAKRKEPEPKGSTKKKAKTGAAGKFKRGK
>1UL4A
GSSGSSGLRLCQVDRCTADMKEAKLYHRRHKVCEVHAKASSVFLSGLNQRFCQQCSRFHDLQEFD
EAKRSCRRRLAGHNERRRKSSGESGPSSG
>1ULYA
MAKKVKVITDPEVIKVMLEDTRRKILKLLRNKEMTISQLSEILGKTPQTIYHHIEKLKEAGLVEV
KRTEMKGNLVEKYYGRTADVFYINLYLGDEELRYIARSRLKTKIDIFKRLGYQFEENELLNIMDR
MSQKEFDATVRISKYIEEKEDALKDFSNEDIIHAIEWLSTAELARDEEYLELLKRLGSILKR
>1USTA
KKEEASSKSYRELIIEGLTALKERKGSSRPALKKFIKENYPIVGSASNFDLYFNNAIKKGVEAGD
FEQPKGPAGAVKLAKKKSPEVKKEKEVS
>1UVHD
MTSFTIPGLSDKKASDVADLLQKQLSTYNDLHLTLKHVHWNVVGPNFIGVHEMIDPQVELVRGYA
DEVAERIATLGKSPKGTPGAIIKDRTWDDYSVERDTVQAHLAALDLVYNGVIEDTRKSIEKLEDL
DLVSQDLLIAHAGELEKFQWFVRAHLESAGGQLTHEGQSTEKGAADKARRKSA
>1V63A
GSSGSSGPKKPPMNGYQKFSQELLSNGELNHLPLKERMVEIGSRWQRISQSQKEHYKKLAEEQQR

QYKVHLDLWVKSLSPQDRAAYKEYISNKRKSGPSSG
>1VJFA
MGSDKIHHHHHHMKTRADLFAFFDAHGVDHKTLDHPPVFRVEEGLEIKAAMPGGHTKNLFLKDAK
GQLWLISALGETTIDLKKLHHVIGSGRLSFGPQEMMLETLGVTPGSVTAFGLINDTEKRVRFVLD
KALADSDPVNFHPLKNDATTAVSQAGLRRFLAALGVEPMIVDFAAMEVVG
>1WEOA
GSSGSSGPKPLKNLDGQFCEICGDQIGLTVEGDLFVACNECGFPACRPCYEYERREGTQNCPQCK
TRYKRLRGSPRVEGDEDEEDIDSGPSSG
>1WEPA
GSSGSSGMALVPVYCLCRQPYNVNHFMIECGLCQDWFHGSCVGIEEENAVDIDIYHCPDCEAVFG
PSIMKNWHSGPSSG
>1WEUA
GSSGSSGSPEYGMPSVTFGSVHPSDVLDMPVDPNEPTYCLCHQVSYGEMIGCDNPDCSIEWFHFA
CVGLTTKPRGKWFCPRCSQESGPSSG
>1WEWA
GSSGSSGEDPFQPEIKVRCVCGNSLETDSMIQCEDPRCHVWQHVGCVILPDKPMDGNPPLPESFY
CEICRLTSGPSSG
>1WG2A
GSSGSSGPSRPVRPNNRCFSCNKKVGVMGFKCKCGSTFCGSHRYPEKHECSFDFKEVGSGPSSG
>1WG6A
GSSGSSGLKGEPDCYALSLESSEQLTLEIPLNDSGSAGLGVSLKGNKSRETGTDLGIFIKSIIHG
GAAFKDGRLRMNDQLIAVNGETLLGKSNHEAMETLRRSMSMEGNIRGMIQLVILRRSGPSSG
>1WH5A
GSSGSSGSSAEAGGGIRKRHRTKFTAEQKERMLALAERIGWRIQRQDDEVIQRFCQETGVPRQVL
KVWLHNNKHSGPSSG
>1WI3A
GSSGSSGPRSRTKISLEALGILQSFIHDVGLYPDQEAIHTLSAQLDLPKHTIIKFFQNQRYHVKH
SGPSSG
>1WIJA
GSSGSSGSQFVLQDLQDATLGSLLSSLMQHCDPPQRKYPLEKGTPPPWWPTGNEEWWVKLGLPKS
QSPPYRKPHDLKKMWKVGVLTAVINHMLPDIAKIKRHVRQSKCLQDKMTAKESAIWLAVLNQEES
LIQQSGPSSG
>1WJ2A
GSSGSSGVQTTSEVDLLDDGYRWRKYGQKVVKGNPYPRSYYKCTTPGCGVRKHVERAATDPKAVV
TTYEGKHNHDLPA
>1WJVA
GSSGSSGMVFFTCNACGESVKKIQVEKHVSNCRNCECLSCIDCGKDFWGDDYKSHVKCISEGQKY
GGKGYEAKSGPSSG
>1WPKA
MKKATCLTDDQRWQSVLARDPNADGEFVFAVRTTGIFCRPSCRARHALRENVSFYANASEALAAG
FRPCKRCQPEKANAQQHRLDKITHACRLLEQETPVTLEALADQVAMSPFHLHRLFKATTGMTPKA
WQQAWRARRLRESLAK
>1X3CA
GSSGSSGRKKPVSQSLEFPTRYSPYRPYRCVHQGCFAAFTIQQNLILHYQAVHKSDLPAFSAEVE

EESGPSSG
>1X51A
GSSGSSGPRKASRKPPREESSATCVLEQPGALGAQILLVQRPNSGLLAGLWEFPSVTWEPSEQLQ
RKALLQELQRWAGPLPATHLRHLGEVVHTFSHIKLTYQVYGLALEGQTPVTTVPPGARWLTQEEF
HTAAVSTAMKKVFRVYQGQSGPSSG
>1X57A
GSSGSSGDRVTLEVGKVIQQGRQSKGLTQKDLATKINEKPQVIADYESGRAIPNNQVLGKIERAI
GLKLRGKDIGKPIEKGPRAKSGPSSG
>1X6FA
GSSGSSGLKRDFIILGNGPRLQNSTYQCKHCDSKLQSTAELTSHLNIHNEEFQKRAKRQERRKQL
LSKQKYADGAFADFKQESGPSSG
>1XCBA
MKVPEAAISRLITYLRILEELEAQGVHRTSSEQLGELAQVTAFQVRKDLSYFGSYGTRGVGYTVP
VLKRELRHILGLNRKWGLCIVGMGRLGSALADYPGFGESFELRGFFDVDPEKVGRPVRGGVIEHV
DLLPQRVPGRIEIALLTVPREAAQKAADLLVAAGIKGILNFAPVVLEVPKEVAVENVDFLAGLTR
LSFAILNPKWREEMMG
>1XD7A
MSLINSRLAVAIHILSLISMDEKTSSEIIADSVNTNPVVVRRMISLLKKADILTSRAGVPGASLK
KDPADISLLEVYRAVQKQEELFAVHENPNPKCPVGKKIQNALDETFESVQRAMENELASKSLKDV
MNHLFEGGSHHHHHH
>1XNAA
MPEIRLRHVVSCSSQDSTHCAENLLKADTYRKWRAAKAGEKTISVVLQLEKEEQIHSVDIGNDGS
AFVEVLVGSSAGGAGEQDYEVLLVTSSFMSPSESRSGSNPNRVRMFGPDKLVRAAAEKRWDRVKI
VCSQPYSKDSPFGLSFVRFHSPPDKDEAEAPSQKVTVTKLGQFRVKEEEESAN
>1XP8A
GSHMSKDATKEISAPTDAKERSKAIETAMSQIEKAFGKGSIMKLGAESKLDVQVVSTGSLSLDLA
LGVGGIPRGRITEIYGPESGGKTTLALAIVAQAQKAGGTCAFIDAEHALDPVYARALGVNTDELL
VSQPDNGEQALEIMELLVRSGAIDVVVVDSVAALTPRAEIEGDMGDSLPGLQARLMSQALRKLTA
ILSKTGTAAIFINQVREKIGVMYGNPETTTGGRALKFYASVRLDVRKIGQPTKVGNDAVANTVKI
KTVKNKVAAPFKEVELALVYGKGFDQLSDLVGLAADMDIIKKAGSFYSYGDERIGQGKEKTIAYI
AERPEMEQEIRDRVMAAIRAGNAGEAPALAPAPAAPEAAEA
>1XV9B
PVQLSKEQEELIRTLLGAHTRHMGTMFEQFVQFRPPAHLFIHHQPLPTLAPVLPLVTHFADINTF
MVLQVIKFTKDLPVFRSLPIEDQISLLKGAAVEICHIVLNTTFCLQTQNFLCGPLRYTIEDGARV
GFQVEFLELLFHFHGTLRKLQLQEPEYVLLAAMALFSPDRPGVTQRDEIDQLQEEMALTLQSYIK
GQQRRPRDRFLYAKLLGLLAELRSINEAYGYQIQHIQGLSAMMPLLQEICS
>1XWRD
MVRANKRNEALRIESALLNKIAMLGTEKTAEAVGVDKSQISRWKRDWIPKFSMLLAVLEWGVVDD
DMARLARQVAAILTNKKRPAATERSEQIQMEF
>1Y6UA
AGHMKQTDIPIWERYTLTIEEASKYFRIGENKLRRLAEENKNANWLIMNGNRIQIKRKQFEKIID
TLDAI
>1YD6A
MNERLKEKLAVLPEQPGCYLMKDKHGTVIYVGKAKSLKERVRSYFTGTHDGKTQRLVEEIADFEY

IVTSSNAEALILEMNLIKKHDPKYNVMLKDDKSY

>1YDXA

MGHHHHHHHHHHSSGHIDDDDKHMTPKLKLNNNINWTKRTIDSLFDLKKGEMLEKELITPEGKYE
YFNGGVKNSGRTDKFNTFKNTISVIVGGSCGYVRLADKNFFCGQSNCTLNLLDPLELDLKFAYYA
LKSQQERIEALAFGTTIQNIRISDLKELEIPFTSNKNEQHAIANTLSVFDERLENLASLIEINRK
LRDEYAHKLFSLDEAFLSHWKLEALQSQMHEITLGEIFNFKSGKYLKSEERLEEGKFPYYGAGID
NTGFVAEPNTEKDTISIISNGYSLGNIRYHEIPWFNGTGSIALEPMNNEIYVPFFYCALKYLQKD
IKERMKSDDSPFLSLKLAGEIKVPYVKSFQLQRKAGKIVFLLDQKLDQYKKELSSLTVIRDTLLK
KLFPDMTERTKSIKDY

>1YIOA

MTAKPTVFVVDDDMSVREGLRNLLRSAGFEVETFDCASTFLEHRRPEQHGCLVLDMRMPGMSGIE
LQEQLTAISDGIPIVFITAHGDIPMTVRAMKAGAIEFLPKPFEEQALLDAIEQGLQLNAERRQAR
ETQDQLEQLFSSLTGREQQVLQLTIRGLMNKQIAGELGIAEVTVKVHRHNIMQKLNVRSLANLVH
LVEKYESFERGVS

>1YJMC

MSQLGSRGRLWLQSPTGGPPPIFLPSDGQALVLGRGPLTQVTDRKCSRNQVELIADPESRTVAVK
QLGVNPSTVGVHELKPGLSGSLSLGDVLYLVNGLYPLTLRWEELS

>1YSEA

GSHVSRSMNKPLEQQVSTNTEVSSEIYQWVRDELKRAGISQAVFARVAFNRTQGLLSEILRKEED
PKTASQSLLVNLRAMQNFLQLPEAERDRIYQDERERSLNAASAMGPAPLISTPPSRPPQVKTATI
ATERNGKPENN

>1YUAA

MNGEVAPPKEDPVPLPELPCEKSDAYFVLRDGAAGVFLAANTFPKSRETRAPLVEELYRFRDRLP
EKLRYLADAPQQDPEGNKTMVRFSRKTKQQYVSSEKDGKATGWSAFYVDGKWVEGKK

>1YUIA

PKAKRAKHPPGTEKPRSRSQSEQPATCPICYAVIRQSRNLRRHLELRHFAKPGV

>1Z1BB

MGRRRSHERRDLPPNLYIRNNGYYCYRDPRTGKEFGLGRDRRIAITEAIQANIELFSGHKHKPLT
ARINSDNSVTLHSWLDRYEKILASRGIKQKTLINYMSKIKAIRRGLPDAPLEDITTKEIAAMLNG
YIDEGKAASAKLIRSTLSDAFREAIAEGHITTNHVAATRAAKSKVRRSRLTADEYLKIYQAAESS
PCWLRLAMELAVVTGQRVGDLCEMKWSDIVDGYLYVEQSKTGVKIAIPTALHIDALGISMKETLD
KCKEILGGETIIASTRREPLSSGTVSRYFMRARKASGLSFEGDPPTFHELRSLSARLYEKQISDK
FAQHLLGHKSDTMASQYRDDRGREWDKIEIK

>1Z4HA

MQHELQPDSLVDLKFIMADTGFGKTFIYDRIKSGDLPKAKVIHGRARWLYRDHCEFKNKLLSRAN
G

>1Z91A

MENKFDHMKLENQLSFLLYASSREMTKQYKPLLDKLNITYPQYLALLLLWEHETLTVKKMGEQLY
LDSGTLTPMLKRMEQQGLITRKRSEEDERSVLISLTEDGALLKEKAVDIPGTILGLSKQSGEDLK
QLKSALYTLLETLHQKN

>1ZAEB

HMDKTVNLSACEVAVLDLYEQSNIRIPSDIIEDLVNQRLQSEQEVLNYIETQRTYWKLENQKKLY
RGSLK

>1ZI0B

TQEDVVVTLSHQGYVKYQPLSEYEAQRRGGKGKSAARIKEEDFIDRLLVANTHDHILCFSSRGRV
YSMKVYQLPEATRGARGRPIVNLLPLEQDERITAILPVTEFEEGVKVFMATANGTVKKTVLTEFN
RLRTAGKVAIKLVDGDELIGVDLTSGEDEVMLFSAEGKVVRFKESSVRAMGCNTTGVRGIRLGEG
DKVVSLIVPRGDGAILTATQNGYGKRTAVAEYPTKSRATKGVISIKVTERNGLVVGAVQVDDCDQ
IMMITDAGTLVRTRVSEISIVGRNTQGVILIRTAEDENVVGLQRVAE
>1ZP7B
MIRYPNGKTFQPKHSVSSQNSQKRAPSYSNRGMTLEDDLNETNKYYLTNQIAVIHKKPTPVQIVN
VHYPKRSAAVIKEAYFKQSSTTDYNGIYKGRYIDFEAKETKNKTSFPLQNFHDHQIEHMKQVKAQ
DGICFVIISAFDQVYFLEADKLFYFWDRKEKNGRKSIRKDELEETAYPISLGYAPRIDYISIIEQ
LYFSPSSGAKG
>1ZRJA
GSSGSSGMDVRRLKVNELREELQRRGLDTRGLKAELAERLQAALSGPSSG
>1ZS3A
MITKLMIDEKYAKELDKAEIDHHKPTAGAMLGHVLSNLFIENIRLTQAGIYAKSPVKCEYLREIA
QREVEYFFKISDLLLDENEIVPSTTEEFLKYHKFITEDPKAKYWTDEDLLESFIVDFQAQNMFIT
RAIKLANKEEKFALAAGVVELYGYNLQVIRNLAGDLGKSVADFHDEDEDNDN
>1ZZKA
GAMGPIITTQVTIPKDLAGSIIGKGGQRIKQIRHESGASIKIDEPLEGSEDRIITITGTQDQIQN
AQYLLQNSVKQYSGKFF
>2A1IA
MGSSHHHHHHSQDPAKSNSIIVSPRQRGNPVLKFVRNVPWEFGDVIPDYVLGQSTCALFLSLRYH
NLHPDYIHGRLQSLGKNFALRVLLVQVDVKDPQQALKELAKMCILADCTLILAWSPEEAGRYLET
YKAYEQKPADLLMEKL
>2A1JB
MGSSHHHHHHSQDPADLLMEKLEQDFVSRVTECLTTVKSVNKTDSQTLLTTFGSLEQLIAASRED
LALCPGLGPQKARRLFDVLHEPFLKV
>2A1JA
MPQDFLLKMPGVNAKNCRSLMHHVKNIAELAALSQDELTSILGNAANAKQLYDFIHTSFAEVV
>2ADLB
MKQRITVTVDSDSYQLLKAYDVNISGLVSTTMQNEARRLRAERWKVENQEGMVEVARFIEMNGSF
ADENKDW
>2AIFA
GSSQNEASEDTGFNPKAFPLASPDLNNKIINLVQQACNYKQLRKGANEATKALNRGIAEIVLLAA
DAEPLEILLHLPLVCEDKNTPYVFVRSKVALGRACGVSRPVIAAAITSKDGSSLSSQITELKDQI
EQILV
>2ALCA
GSMADTRRRQNHSCDPCRKGKRRCDAPENRNEANENGWVSCSNCKRWNKDCTFNWLSSQRSKNSS
>2AN7B
MSRLTIDMTDQQHQSLKALAALQGKTIKQYALERLFPGDADADQAWQELKTMLGNRINDGLAGKV
STKSVGEILDEELSGDRA
>2AQLB
MNRVEVKVKIPEELKPWLVDDWDLITRQKQLFYLPAKKNVDSILEDYANYKKSRGNTDNKEYAVN
EVVAGIKEYFNVMLGTQLLYKFERPQYAEILADHPDAPMSQVYGAPHLLRLFVRIGAMLAYTPLD
EKSLALLLNYLHDFLKYLAKNSATLFSASDYEVAPPEYHRKAV

>2ATQB
MKGFSSEDKGEWKLKLDASGNGQAVIRFLPAKTDDALPFAILVNHGFKKNGKWYIETCSSTHGDY
DSCPVCQYISKNDLYNTNKTEYSQLKRKTSYWANILVVKDPQAPDNEGKVFKYRFGKKIWDKINA
MIAVDTEMGETPVDVTCPWEGANFVLKVKQVSGFSNYDESKFLNQSAIPNIDDESFQKELFEQMV
DLSEMTSKDKFKSFEELNTKFNQVLGTAALGGAAAAAAS
>2ATQA
MKEFYLTVEQIGDSIFERYIDSNGRERTREVEYKPSLFAHCPESQATKYFDIYGKPCTRKLFANM
RDASQWIKRMEDIGLEALGMDDFKLAYLSDTYNYEIKYDHTKIRVANFDIEVTSPDGFPEPSQAK
HPIDAITHYDSIDDRFYVFDLLNSPYGNVEEWSIEIAAKLQEQGGDEVPSEIIDKIIYMPFDNEK
ELLMEYLNFWQQKTPVILTGWNVESFAIPYVYNRIKNIFGESTAKRLSPHRKTRVKVIENMYGSR
EIITLFGISVLDYIDLYKKFSFTNQPSYSLDYISEFELNVGKLKYDGPISKLRESNHQRYISYNI
IAVYRVLQIDAKRQFINLSLDMGYYAKIQIQSVFSPIKTWDAIIFNSLKEQNKVIPQGRSHPVQP
YPGAFVKEPIPNRYKYVMSFDLTSLYPSIIRQVNISPETIAGTFKVAPLHDYINAVAERPSDVYS
CSPNGMMYYKDRDGVVPTEITKVFNQRKEHKGYMLAAQRNGEIIKEALHNPNLSVDEPLDVDYRF
DFSDEIKEKIKKLSAKSLNEMLFRAQRTEVAGMTAQINRKLLINSLYGALGNVWFRYYDLRNATA
ITTFGQMALQWIERKVNEYLNEVCGTEGEAFVLYGDTDSIYVSADKIIDKVGESKFRDTNHWVDF
LDKFARERMEPAIDRGFREMCEYMNNKQHLMFMDREAIAGPPLGSKGIGGFWTGKKRYALNVWDM
EGTRYAEPKLKIMGLETQKSSTPKAVQKALKECIRRMLQEGEESLQEYFKEFEKEFRQLNYISIA
SVSSANNIAKYDVGGFPGPKCPFHIRGILTYNRAIKGNIDAPQVVEGEKVYVLPLREGNPFGDKC
IAWPSGTEITDLIKDDVLHWMDYTVLLEKTFIKPLEGFTSAAKLDYEKKASLFDMFDF
>2AXLA
MDDSEDTSWDFGPQAFKLLSAVDILGEKFGIGLPILFLRGSNSQRLADQYRRHSLFGTGKDQTES
WWKAFSRQLITEGFLVEVSRYNKFMKICALTKKGRNWLHKANTESQSLILQANEELCPKKLLLPS
SKTVSSGTKEHCYN
>2AY0A
MGTTTMGVMLDDATRERIKSAATRIDRTPHWLIKQAIFSYLEQLENSDTLPEHHHHHH
>2AY0B
MGTTTMGVMLDDATRERIKSAATRIDRTPHWLIKQAIFSYLEQLENSDTLPEHHHHHH
>2AY0C
MGTTTMGVMLDDATRERIKSAATRIDRTPHWLIKQAIFSYLEQLENSDTLPEHHHHHH
>2B0LC
GSSHHHHHHMSKAVVQMAISSLSYSELEAIEHIFEELDGNEGLLVASKIADRVGITRSVIVNALR
KLESAGVIESRSLGMKGTYIKVLNNKFLIELENLKSH
>2BA3B
SDSAVRKKSEVRQKTVVRTLRFSPVEDETIRKKAEDSGLTVSAYIRNAALN
>2BDEA
MDTHKVFVNRIINMRKIKLIGLDMDHTLIRYNSKNFESLVYDLVKERLAESFHYPEEIKKFKFNF
DDAIRGLVIDSKNGNILKLSRYGAIRLSYHGTKQISFSDQKKIYRSIYVDLGDPNYMAIDTSFSI
AFCILYGQLVDLKDTNPDKMPSYQAIAQDVQYCVDKVHSDGTLKNIIIKNLKKYVIREKEVVEGL
KHFIRYGKKIFILTNSEYSYSKLLLDYALSPFLDKGEHWQGLFEFVITLANKPRFFYDNLRFLSV
NPENGTMTNVHGPIVPGVYQGGNAKKFTEDLGVGGDEILYIGDHIYGDILRLKKDCNWRTALVVE
ELGEEIASQIRALPIEKKIGEAMAIKKELEQKYVDLCTRSIDESSQQYDQEIHDLQLQISTVDLQ
ISRLLQEQNSFYNPKWERVFRAGAEESYFAYQVDRFACIYMEKLSDLLEHSPMTYFRANRRLLAH
DIDIAAALEHHHHHH

>2BNZA
MAKKDIMGDKTVRVRADLHHIIKIETAKNGGNVKEVMDQALEEYIRKYLPDKL
>2COBA
GSSGSSGRGRYRQYNSEILEEAISVVMSGKMSVSKAQSIYGIPHSTLEYKVKERLGTLKNPPKKK
MKLMR
>2CXYA
GSSGSSGEKITKVYELGNEPERKLWVDRYLTFMEERGSPVSSLPAVGKKPLDLFRLYVCVKEIGG
LAQVNKNKKWRELATNLNVGTSSSAASSLKKQYIQYLFAFECKIERGEEPPPEVFSTGDT
>2D7LA
GSSGSSGRPKTGFQMWLEENRSNILSDNPDFSDEADIIKEGMIRFRVLSTEERKVWANKAKGETA
SEGTEAKKRKSGPSSG
>2D8MA
GSSGSSGEPRRPRAGPEELGKILQGVVVVLSGFQNPFRSELRDKALELGAKYRPDWTRDSTHLIC
AFANTPKYSQVLGLGGRIVRKEWVLDCHRMRRRLPSQRYLMAGPGSSSEEDEASHSGGSGPSSG
>2D9HA
GSSGSSGLQCEICGFTCRQKASLNWHQRKHAETVAALRFPCEFCGKRFEKPDSVAAHRSKSHPAL
LLAPQESSGPSSG
>2DA6A
GSSGSSGRNRFKWGPASQQILYQAYDRQKNPSKEEREALVEECNRAECLQRGVSPSKAHGLGSNL
VTEVRVYNWFANRRKEEAFRQKLAMDAYSSNSGPSSG
>2DGZA
GSSGSSGSSQPVISAQEQETQIVLYGKLVEARQKHANKMDVPPAILATNKILVDMAKMRPTTVEN
VKRIDGVSEGKAAMLAPLWEVIKHFCQTNSVQTDLFSSTKPQSGPSSG
>2DIGA
GSSGSSGMPSRKFADGEVVRGRWPGSSLYYEVEILSHDSTSQLYTVKYKDGTELELKENDIKSGP
SSG
>2DINA
GSSGSSGKKTEWSREEEEKLLHLAKLMPTQWRTIAPIIGRTAAQCLEHYEFLLDKAAQRDSGPSS
G
>2DMPA
GSSGSSGAYPDFAPQKFKEKTQGQVKILEDSFLKSSFPTQAELDRLRVETKLSRREIDSWFSERR
KLRDSMEQAVLDSMGSGKSGPSSG
>2DMQA
GSSGSSGKRMRTSFKHHQLRTMKSYFAINHNPDAKDLKQLAQKTGLTKRVLQVWFQNARAKFRRN
LLRQENGGVSGPSSG
>2DPDB
MKEEKRSSTGFLVKQRAFLKLYMITMTEQERLYGLKLLEVLRSEFKEIGFKPNHTEVYRSLHELL
DDGILKQIKVKKEGAKLQEVVLYQFKDYEAAKLYKKQLKVELDRSKKLIEKALSDNF
>2DQBA
MRFSREALLELEASRLAPYAQKARDTRGRAHPEPESLYRTPYQKDRDRILHTTAFRRLEYKTQVL
PGWAGDYYRTRLTHTLEVAQVSRSIARALGLNEDLTEAIALSHDLGHPPFGHTGEHVLNALMQDH
GGFEHNAQALRILTHLEVRYPGFRGLNLTYEVLEGIATHEAAYSPGFKPLYEGQGTLEAQVVDLS
DAIAYAAHDLDDGFRAGLLHPEELKEVELLQALALEEGLDLLRLPELDRRVLVRQLLGYFITAAI
EATHRRVEEAGVQSAEAVRRHPSRLAALGEEAEKALKALKAFLMERFYRHPEVLRERRKAEAVLE

GLFAAYTRYPELLPREVQAKIPEEGLERAVCDYIAGMTDRFALEAYRRLSP
>2E5RA
GSSGSSGVFHPVECSYCHSESMMGFRYRCQQCHNYQLCQDCFWRGHAGGSHSNQHQMKEYTSW
>2E6RA
GSSGSSGHSSAQFIDSYICQVCSRGDEDDKLLFCDGCDDNYHIFCLLPPLPEIPRGIWRCPKCIL
AECKQPPEAFGFEQATQEYSLSGPSSG
>2EBIA
KKRAETWVQDETRSLIMFRRGMDGLFNTSKSNKHLWEQISSKMREKGFDRSPDMCTDKWRNLLKE
FKKAKHHDRGNGSAKMSYYKE
>2ELHA
GSSGSSGMNIRMGTKGKRPLRSLTPRDKIHAIQRIHDGESKASVARDIGVPESTLRGWCKNEDKL
RFMSRQSATDNLCADALGDKMD
>2EO0B
MYIVNSNKSRGSSVERYIVSRLRDKGFAVIRAPASGSKRKDHVPDIIALKSGVIILIEVKSRKNG
QKIYIEKEQAEGIREFAKRSGGELFLGVKLPKMLRFIKFDMLRQTEGGNYAIDLETVEKGMELED
LVRYVESKISRTLDSFL
>2EWTA
MSSEYAKQLGAKLRAIRTQQGLSLHGVEEKSQGRWKAVVVGSYERGDRAVTVQRLAELADFYGVP
VQELLP
>2F2EB
MVKRTSHKQASCPVARPLDVIGDGWSMLIVRDAFEGLTRFGEFQKSLGLAKNILAARLRNLVEHG
VMVAVPAESGSHQEYRLTDKGRALFPLLVAIRQWGEDYFFAPDESHVRLVERDSGQPVPRLQVRA
GDGSPLAAEDTRVSRD
>2F7NA
MTKKSTKSEAASKTKKSGVPETGAQGVRAGGADHADAAHLGTVNNALVNHHYLEEKEFQTVAETL
QRNLATTISLYLKFKKYHWDIRGRFFRDLHLAYDEFIAEIFPSIDEQAERLVALGGSPLAAPADL
ARYSTVQVPQETVRDARTQVADLVQDLSRVGKGYRDDSQACDEANDPVTADMYNGYAATIDKIRW
MLQAIMDDERLD
>2FC7A
GSSGSSGQQMQAESGFVQHVGFKCDNCGIEPIQGVRWHCQDCPPEMSLDFCDSCSDCLHETDIHK
EDHQLEPIYRSSGPSSG
>2FE3B
MAAHELKEALETLKETGVRITPQRHAILEYLVNSMAHPTADDIYKALEGKFPNMSVATVYNNLRV
FRESGLVKELTYGDASSRFDFVTSDHYHAICENCGKIVDFHYPGLDEVEQLAAHVTGFKVSHHRL
EIYGVCQECSKKENH
>2FMYA
ATQMRLTDTNLLEVLNSEEYSGVLKEFREQRYSKKAILYTPNTERNLVFLVKSGRVRVYLAYEDK
EFTLAILEAGDIFCTHTRAFIQAMEDTTILYTDIRNFQNIVVEFPAFSLNMVKVLGDLLKNSLTI
INGLVFKDARLRLAEFLVQAAMDTGLKVPQGIKLELGLNTEEIALMLGTTRQTVSVLLNDFKKMG
ILERVNQRTLLLKDLQKLKEFSSGV
>2FPHX
GIYQHFSIEDRPFLDKGMEWIKKVEDSYAPFLTPFINPHQEKLLKILAKTYGLACSSSGEFVSSE
YVRVLLYPDYFQPEFSDFEISLQEIVYSNKFEYLTHAKILGTVINQLGIERKLFGDILVDEERAQ
IMINQQFLLLFQDGLKKIGRIPVSLEERPFTEKID

>2FU4B
MTDNNTALKKAGLKVTLPRLKILEVLQEPDNHHVSAEDLYKRLIDMGEEIGLATVYRVLNQFDDA
GIVTRHNFEGGKSVFELT
>2FWRA
MGSSHHHHHHSSGLVPRGSHMQMIAEIYYERGTIVVKGDAHVPHAKFDSRSGTYRALAFRYRDII
EYFESNGIEFVDNAADPIPTPYFDAEISLRDYQEKALERWLVDKRGCIVLPTGSGKTHVAMAAIN
ELSTPTLIVVPTLALAEQWKERLGIFGEEYVGEFSGRIKELKPLTVSTYDSAYVNAEKLGNRFML
LIFDEVHHLPAESYVQIAQMSIAPFRLGLTATFEREDGRHEILKEVVGGKVFELFPDSLAGKHLA
KYTIKRIFVPLAEDERVEYEKREKVYKQFLRARGITLRRAEDFNKIVMASGYDERAYEALRAWEE
ARRIAFNSKNKIRKLREILERHRKDKIIIFTRHNELVYRISKVFLIPAITHRTSREEREEILEGF
RTGRFRAIVSSQVLDEGIDVPDANVGVIMSGSGSAREYIQRLGRILRPSKGKKEAVLYELISRGT
GEVNTARRRKNAAKGAA
>2G9WB
MAKLTRLGDLERAVMDHLWSRTEPQTVRQVHEALSARRDLAYTTVMAVLQRLAKKNLVLQIRDDR
AHRYAPVHGRDELVAGLMVDALAQAEDSGSRQAALVHFVERVGADEADALRRALAELEAGHGNRP
PAGAATET
>2GFUA
KAKNLNGGLRRSVAPAAPTSSDFSPGDLVWAKMEGYPWWPSLVYNHPFDGTFIREKGKSVRVHVQ
FFDDSPTRGWVSKRLLKPYTGSKSKEAQKGGHFYSAKPEILRAMQRADEALNKDKIKRLELAVSD
EPSE
>2GMGA
AHHHHHHGSATRREKIIELLLEGDYSPSELARILDMRGKGSKKVILEDLKVISKIAKREGMVLLI
KPAQCRKCGFVFKAEINIPSRCPKCKSEWIEEPRFKLERK
>2H6BB
MSVEGLGKDFCGAIIPDNFFPIEKLRNYTQMGLIRDFAKGSAVIMPGEEITSMIFLVEGKIKLDI
IFEDGSEKLLYYAGGNSLIGKLYPTGNNIYATAMEPTRTCWFSEKSLRTVFRTDEDMIFEIFKNY
LTKVAYYARQVAEMNTYNPTIRILRLFYELCSSQGKRVGDTYEITMPLSQKSIGEITGVHHVTVS
RVLACLKRENILDKKKNKIIVYNLGELKHLSEQTSYYSDPNSSSVDKLAAALDHH
>2H9UA
MACEGAPEVRIGRKPVMNYVLAILTTLMEQGTNQVVVKARGRNINRAVDAVEIVRKRFAKNIEIK
DIKIDSQEIEVQTPEGQTRTRRVSSIEICLEKAGESA
>2HGVA
GSSHHHHHHMALLQKTRIINSMLQAAAGKPVNFKEMAETLRDVIDSNIFVVSRRGKLLGYSINQQ
IENDRMKKMLEDRQFPEEYTKNLFNVPETSSNLDINSEYTAFPVENRDLFQAGLTTIVPIIGGGE
RLGTLILSRLQDQFNDDDLILAEYGATVVGMEIL
>2HKVA
GMTDWQQALDRHVGVGVRTTRDLIRLIQPEDWDKRPISGKRSVYEVAVHLAVLLEADLRIATGAT
ADEMAQFYAVPVLPEQLVDRLDQSWQYYQDRLMADFSTETTYWGVTDSTTGWLLEAAVHLYHHRS
QLLDYLNLLGYDIKLDLFE
>2HQLF
GGGGGGMLNRVFLEGEIESSCWSVKKTGFLVTIKQMRFFGERLFTDYYVIYANGQLAYELEKHTK
KYKTISIEGILRTYLERKSEIWKTTIEIVKIFNPKNEIVIDYKEI
>2HUEA
PLGSPNSSIVSLLGIKVLNNPAKFTDPYEFEITFECLESLKHDLEWKLTYVGSSRSLDHDQELDS

ILVGPVPVGVNKFVFSADPPSAELIPASELVSVTVILLSCSYDGREFVRVGYYVNNEYDEEELRE
NPPAKVQVDHIVRNILAEKPRVTRFNIVWDNENEGDLYPPEQPGV
>2I13B
ISEFGSSSSVAQAALEPGEKPYACPECGKSFSRSDHLAEHQRTHTGEKPYKCPECGKSFSDKKDL
TRHQRTHTGEKPYKCPECGKSFSQRANLRAHQRTHTGEKPYACPECGKSFSQLAHLRAHQRTHTG
EKPYKCPECGKSFSREDNLHTHQRTHTGEKPYKCPECGKSFSRRDALNVHQRTHTGKKTS
>2IJGX
MNDHIHRVPALTEEEIDSVAIKTFERYALPSSSSVKRKGKGVTILWFRNDLRVLDNDALYKAWSS
SDTILPVYCLDPRLFHTTHFFNFPKTGALRGGFLMECLVDLRKNLMKRGLNLLIRSGKPEEILPS
LAKDFGARTVFAHKETCSEEVDVERLVNQGLKRVGNSTKLELIWGSTMYHKDDLPFDVFDLPDVY
TQFRKSVEAKCSIRSSTRIPLSLGPTPSVDDWGDVPTLEKLGVEPQEVTRGMRFVGGESAGVGRV
FEYFWKKDLLKVYKETRNGMLGPDYSTKFSPWLAFGCISPRFIYEEVQRYEKERVANNSTYWVLF
ELIWRDYFRFLSIKCGNSLFHLGGPRNVQGKWSQDQKLFESWRDAKTGYPLIDANMKELSTTGFM
SNRGRQIVCSFLVRDMGLDWRMGAEWFETCLLDYDPCSNYGNWTYGAGVGNDPREDRYFSIPKQA
QNYDPEGEYVAFWLQQLRRLPKEKRHWPGRLMYMDTVVPLKHGNGPMAGGSKSGGGFRGSHSGRR
SRHNGP
>2INGX
GPRMSMVVSGLTPEEFMLVYKFARKHHITLTNLITEETTHVVMKTDAEFVCERTLKYFLGIAGGK
WVVSYFWVTQSIKERKMLNEHDFEVRGDVVNGRNHQGPKRARESQDRKIFRGLEICCYGPFTNKP
TDQLEWMVQLCGASVVKELSSFTLGTGVHPIVVVQPDAWTEDNGFHAIGQMCEAPVVTREWVLDS
VALYQCQELDTYLIPQIP
>2IO4B
MMKAKVIDAVSFSYILRTVGDFLSEANFIVTKEGIRVSGIDPSRVVFLDIFLPSSYFEGFEVSQE
KEIIGFKLEDVNDILKRVLKDDTLILSSNESKLTLTFDGEFTRSFELPLIQVESTQPPSVNLEFP
FKAQLLTITFADIIDELSDLGEVLNIHSKENKLYFEVIGDLSTAKVELSTDNGTLLEASGADVSS
SYGMEYVANTTKMRRASDSMELYFGSQIPLKLRFKLPQEGYGDFYIAPRAD
>2IO4A
MVKIVYPNAKDFFSFINSITNVTDSIILNFTEDGIFSRHLTEDKVLMAIMRIPKDVLSEYSIDSP
TSVKLDVSSVKKILSKASSKKATIELTETDSGLKIIIRDEKSGAKSTIYIKAEKGQVEQLTEPKV
NLAVNFTTDESVLNVIAADVTLVGEEMRISTEEDKIKIEAGEEGKRYVAFLMKDKPLKELSIDTS
ASSSYSAEMFKDAVKGLRGFSAPTMVSFGENLPMKIDVEAVSGGHMIFWIAPRL
>2JD3B
MDDERKRKKYTLYLHPEKAADFQTLEAIESVPRSERGELFRNAFISGMALHQLDPRLPVLLTAIL
SEEFSADQVVTLLSQTTGWKPSQADIRAVLTELGASQSVEKMPPSATDSVQEAMNDVRLKMKKLF
>2JMPA
MGGGGGGMEQFNAFKSLLKKHYEKTIGFHDKYIKDINRFVFKNNVLLILLENEFARNSLNDNSEI
IHLAESLYEGIKSVNFVNEQDFFFNLAKLEENSRDTLYQNSG
>2JPCA
LRERQVLKLIDEGYTNHGISEKLHISIKTVETHRMNMMRKLQVHKVTELLNCARRMRLIEY
>2JR1A
FNVKQKSEITALVKEVTPPRKAPSKAKREAPIKYWLPHSGATWSGRGKIPKPFEAWIGTAAYTAW
KAKHPDEKFPAFPG
>2JTMA
MSSGKKPVKVKTPAGKEAELVPEKVWALAPKGRKGVKIGLFKDPETGKYFRHKLPDDYPI

>2JULA
MQRTKEAVKASDGNLLGDPGRIPLSKRESIKWQRPRFTRQALMRCCLIKWILSSAAPQGSDSSDS
ELELSTVRHQPEGLDQLQAQTKFTKKELQSLYRGFKNECPTGLVDEDTFKLIYSQFFPQGDATTY
AHFLFNAFDADGNGAIHFEDFVVGLSILLRGTVHEKLKWAFNLYDINKDGCITKEEMLAIMKSIY
DMMGRHTYPILREDAPLEHVERFFQKMDRNQDGVVTIDEFLETCQKDENIMNSMQLFENVI
>2JX3A
FTIAQGKGQKLCEIERIHFFLSKKKTDELRNLHKLLYNRPGTVSSLKKNVGQFSGFPFEKGSVQY
KKKEEMLKKFRNAMLKSICEVLDLERSGVNSELVKRILNFLMHPKPSGKPLPKSKKTCSKGSKKE
R
>2K4BA
MSYYHHHHHHDYDIPTTENLYFQGAMNEVEFNVSNAELIVMRVIWSLGEARVDEIYAQIPQELEW
SLATVKTLLGRLVKKEMLSTEKEGRKFVYRPLME
>2K5VA
MNYKISELMPNLSGTINAEVVAAYPKKEFSRKDGTKGQLKSLFLKDDTGSIRGTLWNELADFEVK
KGDIAEVSGYVKQGYSGLEISVDNIGIIEKSLEHHHHHH
>2K6GA
KRTNYQAYRSYLNREGPKALGSKEIPKGAENCLEGLIFVITGVLESIERDEAKSLIERYGGKVTG
NVSKKTNYLVMGRDSGQSKSDKAAALGTKIIDEDGLLNLIRNLE
>2K75A
SDLVKIRDVSLSTPYVSVIGKITGIHKKEYESDGTTKSVYQGYIEDDTARIRISSFGKQLQDSDV
VRIDNARVAQFNGYLSLSVGDSSRIESVNVNIPLEHHHHHH
>2K86A
GSSSRRNAWGNLSYADLITRAIESSPDKRLTLSQIYEWMVRCVPYFKDKGDSNSSAGWKNSIRHN
LSLHSRFMRVQNEGTGKSSWWIINPDGGKSGKAPRRRA
>2K9IB
GRPYKLLNGIKLGVYIPQEWHDRLMEIAKEKNLTLSDVCRLAIKEYLDNHDKQKK
>2K9NA
KVKFTEEEDLKLQQLVMRYGAKDWIRISQLMITRNPRQCRERWNNYINPALRTDPWSPEEDMLLD
QKYAEYGPKWNKISKFLKNRSDNNIRNRWMMIARHRAKHQKS
>2KEBA
MGSSHHHHHHGSSLEVLFQGPGSMSASAQQLAEELQIFGLDCEEALIEKLVELCVQYGQNEEGMV
GELIAFCTSTHKVGLTSEILNSFEHEFLSKRLSKAR
>2KHQA
MITFADYFYQWYEVNKLPHVSESTKRHYESAYKHIKDHFRHKLLKDIKRTEYQKFLNEYGLTHSY
ETIRKLNSYIRNAFDDAIHEGYVIKNPTYKAELHASVLEHHHHHH
>2KI2A
MRNIYVGNLVYSATSEQVKELFSQFGKVFNVKLIYDRETKKPKGFGFVEMQEESVSEAIAKLDNT
DFMGRTIRVTEANPKKSLEHHHHHH
>2KIWA
TFKQVADDWLKQYANDVKVSSVRAREKAIQHAIERFNTKPIQTIKKHDYQRFVDDISAQYSKNYV
DSIVASTNMIFKYAYDTRLIKAMPSEGIKRPKKKVSVELEHHHHHH
>2KJ8A
SSNNNSFSAIYKEWYEHKKQVWSVGYATELAKMFDDDILPIIGGLEIQDIEPMQLLEVIRRFEDR
GAMERANKARRRCGEVFRYAIVTGRAKYNPAPDLADAMKGYRKKNLEHHHHHH

>2KKOB
MAGQSDRKAALLDQVARVGKALANGRRLQILDLLAQGERAVEAIATATGMNLTTASANLQALKSG
GLVEARREGTRQYYRIAGEDVARLFALVQVVADEHLEHHHHHH
>2KKPA
MIEPSKITVEQWLNRWLTDYAKPHLRQSTWESYETVLRLHVIPTLGSIPLKKLQPADIQRLYASK
LESGLSPTRVRYIHVVLHEAMSQARESGLLLQNPTEAAKPPRHPLEHHHHHH
>2KKVA
MENSGAYTFETIAREWHESNKRWSEDHRSRVLRYLELYIFPHIGSSDIRQLKTSHLLAPIKEVDT
SGKHDVAQRLQQRVTAIMRYAVQNDYIDSNPASDMAGALSTTKARHYPLEHHHHHH
>2KNGA
SGSGRGRGAIDREQSAAIREWARRNGHNVSTRGRIPADVIDAYHAATLEHHHHHH
>2KW3C
GHGTGSFGDRPARPTLLEQVLNQKRLSLLRSPEVVQFLQKQQQLLNQQVLEQRQQQFPGTSM
>2KW3B
GAGEPTTLLQRLRGTISKAVQNKVEGILQDVQKFSDNDKLYLYLQLPSGPTTGDKSSEPSTLSNE
EYM
>2KWQA
GPMGMQSIREQSCRVVTCKTCKYTHFKPKETCVSENHDFHWHNGVKRFFKCPCGNRTISLDRLPK
KHCSTCGLFKWERVGMLKEKTGPKLGG
>2L1PA
MGHHHHHHSHMLPPEQWSHTTVRNALKDLLKDMNQSSLAKECPLSQSMISSIVNSTYYANVSAAK
CQEFGRWYKHFKKTKDMM
>2L3NA
SVSILRSSVNHREVDEAIDNILRYTNSTEQQFLEAMESTGGRVRIAIAKLLSKQTSGGSGGSKLG
GSGGSRKDLSVKGMLYDSDSQQILNRLRERVSGSTAQSA
>2L49B
MSNTISEKIVLMRKSEYLSRQQLADLTGVPYGTLSYYESGRSTPPTDVMMNILQTPQFTKYTLWF
MTNQIAPESGQIAPALAHFGQNETTSPHSGQKTG
>2L92A
MSTVPKYRDPATGKTWSGRGRQPAWLGNDPAAFLIQPDLPAILEHHHHHH
>2L93A
AARPAKYSYVDENGETKTWTGQGRTPAVIKKAMEEQGKQLEDFLIKELEHHHHHH
>2LFHB
MGHHHHHHSHMGGGKGPAAEEPLSLLDDMNHCYSRLRELVPGVPRGTQLSQVEILQRVIDYILDL
QVV
>2LJ6A
MPSSKPLAEYARKRDFRQTPEPSGRKPRKDSTGLLRYCVQKHDASRLHYDFRLELDGTLKSWAVP
KGPCLDPAVKRLAVQVEDHPLDYADFEGSIPQGHYGAGDVIVWDRGAWTPLDDPREGLEKGHLSF
ALDGEKLSGRWHLIRTNLRGKQSQWFLVKAKDGEARSLDRFDVLKER
>2LLHA
GSHMQESFKKQEKTPKTPKGPSSVEDIKAKMQASIEKGGSLPKVEAKFINYVKNCFRMTDQEAIQ
DLWQWRKSL
>2LSSA
MATNIVGKVKWYNSTKNFGFIEQDNGGKDVFVHKSAVDAAGLHSLEEGQDVIFDLEEKQGKAYAV

NLRIK
>2LTTB
MADKLKFEIIEELIVLSENAKGWRKELNRVSWNDAEPKYDIRTWSPDHEKMGKGITLSEEEFGVL
LKELGNKLEHHHHHH
>2LUAA
SPPKPKCRCGISGSSNTLTTCRNSRCPCYKSYNSCAGCHCVGCKNPHKEDYV
>2LUYA
HMGKNDNDALIMCMRCRKVKGIDSYSKTQWSKTFTFVRGRTVSVSDPKVICRTCQPKQHDSIWCT
ACQQTKGINEFSKAQRHVLDPRCQICVHSQRN
>2LW1A
GSHMKAETVKRSSSKLSYKLQRELEQLPQLLEDLEAKLEALQTQVADASFFSQPHEQTQKVLADM
AAAEQELEQAFERWEYLEALKNGG
>2LYJB
MIINNLKLIREKKKISQSELAALLEVSRQTINGIEKNKYNPSLQLALKIAYYLNTPLEDIFQWQP
E
>2M14A
PSHSGAAIFEKVSGIIAINEDVSPAELTWRSTDGDKVHTVVLSTIDKLQATPASSEKMMLRLIGK
VDESKKRKDNEGNEVVPKPQRHMFSFNNRTVMDNIKMTLQQIISRYKDADGNSS
>2M8EA
ASMGKSKEISQDLRKKIVDLHKSGSSLGAISKRLKVPRSSVQTIVRKYKHHGTTQHH
>2MA1A
HDAPLFEALRAWRLQKAKELSLPPYTIFHDATLKTIAELRPGSHATLGTVSGVGGRKLAAYGDEV
LQVVRDSSGG
>2MAMA
ADEPAYLTVGTDVSAKYRGAFCEAKIKTVKRLVKVKVLLKQDNTTQLVQDDQVKGPLRVGAIVET
RTSDGSFQEAIISKLTDASWYTVVFDDGDERTLRRTSLCLKGERHFAESETLD
>2MH3B
MKPKTASEHRKSSKPIMEKRRRARINESLSQLKTLILDALKKDSSRHSKLEKADILEMTVKHLRN
LQRAQ
>2NMUA
MAGDPNSMTVSHHNASTARFYALRLLPGQEVFSQLHAFVQQNQLRAAWIAGCTGSLTDVALRYAG
QEATTSLTGTFEVISLNGTLELTGEHLHLAVSDPYGVMLGGHMMPGCTVRTTLELVIGELPALTF
SRQPCAISGYDELHISSRLEHHHHHH
>2NOGB
MVSEPKVPKAPRPPKQPNVQDFQFFPPRLFELLEKEILYYRKTIGYKVPRNPDLPNSAQVQKEEQ
LKIDEAEPLNDEELEEKEKLLTQGFTNWNKRDFNQFIKANEKWGRDDIENIAREVEGKTPEEVIE
YSAVFWERCNELQDIEKTMAQIERGEARIQRRISILEHHHHHH
>2NP2B
MSFSRRPKVTKSDIVDQIALNIKNNNLKLEKKYIRLVIDAFFEELKSNLCSNNVIEFRSFGTFEV
RKRKGRLNARNPQTGEYVKVLDHHVAYFRPGKDLKERVWGIKG
>2O3CC
GSHMEAPILYEDPPEKLTSKDGRAANMKITSWNVDGLRAWVKKNGLDWVRKEDPDILCLQETKCA
EKALPADITAMPEYPHKYWAGSEDKEGYSGVAMLCKTEPLNVTYGIGKEEHDKEGRVITAEFPDF
FLVTAYVPNASRGLVRLDYRKTWDVDFRAYLCGLDARKPLVLCGDLNVAHQEIDLKNPKGNRKNA

GFTPEEREGFTQLLEAGFTDSFRELYPDQAYAYTFWTYMMNARSKNVGWRLDYFVLSSALLPGLC
DSKIRNTAMGSDHCPITLFLAV
>2O8BB
MGSAPQNSESQAHVSGGGDDSSRPTVWYHETLEWLKEEKRRDEHRRRPDHPDFDASTLYVPEDFL
NSCTPGMRKWWQIKSQNFDLVICYKVGKFYELYHMDALIGVSELGLVFMKGNWAHSGFPEIAFGR
YSDSLVQKGYKVARVEQTETPEMMEARCRKMAHISKYDRVVRREICRIITKGTQTYSVLEGDPSE
NYSKYLLSLKEKEEDSSGHTRAYGVCFVDTSLGKFFIGQFSDDRHCSRFRTLVAHYPPVQVLFEK
GNLSKETKTILKSSLSCSLQEGLIPGSQFWDASKTLRTLLEEEYFREKLSDGIGVMLPQVLKGMT
SESDSIGLTPGEKSELALSALGGCVFYLKKCLIDQELLSMANFEEYIPLDSDTVSTTRSGAIFTK
AYQRMVLDAVTLNNLEIFLNGTNGSTEGTLLERVDTCHTPFGKRLLKQWLCAPLCNHYAINDRLD
AIEDLMVVPDKISEVVELLKKLPDLERLLSKIHNVGSPLKSQNHPDSRAIMYEETTYSKKKIIDF
LSALEGFKVMCKIIGIMEEVADGFKSKILKQVISLQTKNPEGRFPDLTVELNRWDTAFDHEKARK
TGLITPKAGFDSDYDQALADIRENEQSLLEYLEKQRNRIGCRTIVYWGIGRNRYQLEIPENFTTR
NLPEEYELKSTKKGCKRYWTKTIEKKLANLINAEERRDVSLKDCMRRLFYNFDKNYKDWQSAVEC
IAVLDVLLCLANYSRGGDGPMCRPVILLPEDTPPFLELKGSRHPCITKTFFGDDFIPNDILIGCE
EEEQENGKAYCVLVTGPNMGGKSTLMRQAGLLAVMAQMGCYVPAEVCRLTPIDRVFTRLGASDRI
MSGESTFFVELSETASILMHATAHSLVLVDELGRGTATFDGTAIANAVVKELAETIKCRTLFSTH
YHSLVEDYSQNVAVRLGHMACMVENECEDPSQETITFLYKFIKGACPKSYGFNAARLANLPEEVI
QKGHRKAREFEKMNQSLRLFREVCLASERSTVDAEAVHKLLTLIKEL
>2O8BA
MAVQPKETLQLESAAEVGFVRFFQGMPEKPTTTVRLFDRGDFYTAHGEDALLAAREVFKTQGVIK
YMGPAGAKNLQSVVLSKMNFESFVKDLLLVRQYRVEVYKNRAGNKASKENDWYLAYKASPGNLSQ
FEDILFGNNDMSASIGVVGVKMSAVDGQRQVGVGYVDSIQRKLGLCEFPDNDQFSNLEALLIQIG
PKECVLPGGETAGDMGKLRQIIQRGGILITERKKADFSTKDIYQDLNRLLKGKKGEQMNSAVLPE
MENQVAVSSLSAVIKFLELLSDDSNFGQFELTTFDFSQYMKLDIAAVRALNLFQGSVEDTTGSQS
LAALLNKCKTPQGQRLVNQWIKQPLMDKNRIEERLNLVEAFVEDAELRQTLQEDLLRRFPDLNRL
AKKFQRQAANLQDCYRLYQGINQLPNVIQALEKHEGKHQKLLLAVFVTPLTDLRSDFSKFQEMIE
TTLDMDQVENHEFLVKPSFDPNLSELREIMNDLEKKMQSTLISAARDLGLDPGKQIKLDSSAQFG
YYFRVTCKEEKVLRNNKNFSTVDIQKNGVKFTNSKLTSLNEEYTKNKTEYEEAQDAIVKEIVNIS
SGYVEPMQTLNDVLAQLDAVVSFAHVSNGAPVPYVRPAILEKGQGRIILKASRHACVEVQDEIAF
IPNDVYFEKDKQMFHIITGPNMGGKSTYIRQTGVIVLMAQIGCFVPCESAEVSIVDCILARVGAG
DSQLKGVSTFMAEMLETASILRSATKDSLIIIDELGRGTSTYDGFGLAWAISEYIATKIGAFCMF
ATHFHELTALANQIPTVNNLHVTALTTEETLTMLYQVKKGVCDQSFGIHVAELANFPKHVIECAK
QKALELEEFQYIGESQGYDIMEPAAKKCYLEREQGEKIIQEFLSKVKQMPFTEMSEENITIKLKQ
LKAEVIAKNNSFVNEIISRIKVTT
>2O99A
GHMSRNLLAIVHPILRNLMEESGETVNMAVLDQSDHEAIIIDQVQCTHLMRMSAPIGGKLPMHAS
GAGKAFLAQLSEEQVTKLLHRKGLHAYTHATLVSPVHLKEDLAQTRKRGYSFDDEEHALGLRCLA
ACIFDEHREPFAAISISGPISRITDDRVTEFGAMVIKAAKEVTLAYGGMRGS
>2OBPB
GMSDPGNEQNGDGIDPAIVEVLLVLREAGIENGATPWSLPKIAKRAQLPMSVLRRVLTQLQAAGL
ADVSVEADGRGHASLTQEGAALAAQLFPDPF
>2OCJD
SSSVPSQKTYQGSYGFRLGFLHSGTAKSVTCTYSPALNKMFCQLAKTCPVQLWVDSTPPPGTRVR

AMAIYKQSQHMTEVVRRCPHHERCSDSDGLAPPQHLIRVEGNLRVEYLDDRNTFRHSVVVPYEPP
EVGSDCTTIHYNYMCNSSCMGGMNRRPILTIITLEDSSGNLLGRNSFEVRVCACPGRDRRTEEEN
LRKKGEPHHELPPGSTKRALPNNT
>2OD5A
GMTGAVETESMKTVRIREKIKKFLGDRPRNTAEILEHINSTMRHGTTSQQLGNVLSKDKDIVKVG
YIKRSGILSGGYDICEWATRNWVAEHCPEWTEGQPIILNEEGDFTLGPLPE
>2OKFA
GMSARDVFHEVVKTALKKDGWQITDDPLTISVGGVNLSIDLAAQKLIAAERQGQKIAVEVKSFLK
QSSAISEFHTALGQFINYRGALRKVEPDRVLYLAVPLTTYKTFFQLDFPKEIIIENQVKMLVYDV
EQEVIFQWIN
>2OWYB
MWFRNLLVYRLTQDLQLDADSLEKALGEKSARPCASQELTTYGFTAPFGKGPDAPLVHVSQDFFL
ISARKEERILPGSVVRDALKEKVDEIEAQQMRKVYKKERDQLKDEIVQTLLPRAFIRRSSTFAAI
APSLGLILVDSASAKKAEDLLSTLREALGSLPVRPLSVKVAPTATLTDWVKTQEAAGDFHVLDEC
ELRDTHEDGGVVRCKRQDLTSEEIQLHLTAGKLVTQLSLAWSDKLSFVLDDKLAVKRLRFEDLLQ
EQAEKDGGEDALGQLDASFTLMMLTFAEFLPALFEALGGEEIPQGV
>2OZ9R
AQQSPYSAAMAEQRHQEWLRFVDLLKNAYQNDLHLPLLNLMLTPDEREALGTRVRIVEELLRGEM
SQRELKNELGAGIATITRGSNSLKAAPVELRQWLEEVLLKSD
>2OZEA
MIQYYYTKKEWGVVMEKEELKILEELRRILSNKNEAIVILNNYFKGGVGKSKLSTMFAYLTDKLN
LKVLMIDKDLQATLTKDLAKTFKVELPRVNFYEGLKNGNLASSIVHLTDNLDLIPGTFDLMLLPK
LTRSWTFENESRLLATLLAPLKSDYDLIIIDTVPTPSVYTNNAIVASDYVMIPLQAEEESTNNIQ
NYISYLIDLQEQFNPGLDMIGFVPYLVDTDSATIKSNLEELYKQHKEDNLVFQNIIKRSNKVSTW
SKNGITEHKGYDKKVLSMYKNVFFEMLERIIQLENEKE
>2P2UB
SLSRRKPNPVIVADIRQAEGALAEIATIDRKVGEIEAQMNEAIDAAKARASQKSAPLLARRKELE
DGVATFATLNKTEMFKDRKSLDLGFGTIGFRLSTQIVQMSKITKDMTLERLRQFGISEGIRIKED
VNKEAMQGWPDERLEMVGLKRRTTDAFYIEINREEVADTAA
>2P5KA
MNKGQRHIKIREIITSNEIETQDELVDMLKQDGYKVTQATVSRDIKELHLVKVPTNNGSYKYSL
>2P5MC
MQRFNPLSKLKRALMDAFVKIDSASHMIVLKTMPGNAQAIGALMDNLDWDEMMGTICGDDTILII
CRTPEDTEGVKNRLLELL
>2P6RA
MKVEELAESISSYAVGILKEEGIEELFPPQAEAVEKVFSGKNLLLAMPTAAGKTLLAEMAMVREA
IKGGKSLYVVPLRALAGEKYESFKKWEKIGLRIGISTGDYESRDEHLGDCDIIVTTSEKADSLIR
NRASWIKAVSCLVVDEIHLLDSEKRGATLEILVTKMRRMNKALRVIGLSATAPNVTEIAEWLDAD
YYVSDWRPVPLVEGVLCEGTLELFDGAFSTSRRVKFEELVEECVAENGGVLVFESTRRGAEKTAV
KLSAITAKYVENEGLEKAILEENEGEMSRKLAECVRKGAAFHHAGLLNGQRRVVEDAFRRGNIKV
VVATPTLAAGVNLPARRVIVRSLYRFDGYSKRIKVSEYKQMAGRAGRPGMDERGEAIIIVGKRDR
EIAVKRYIFGEPERITSKLGVETHLRFHSLSIICDGYAKTLEELEDFFADTFFFKQNEISLSYEL
ERVVRQLENWGMVVEAAHLAPTKLGSLVSRLYIDPLTGFIFHDVLSRMELSDIGALHLICRTPDM
ERLTVRKTDSWVEEEAFRLRKELSYYPSDFSVEYDWFLSEVKTALCLKDWIEEKDEDEICAKYGI

APGDLRRIVETAEWLSNAMNRIAEEVGNTSVSGLTERIKHGVKEELLELVRIRHIGRVRARKLYN
AGIRNAEDIVRHREKVASLIGRGIAERVVEGISVKSLNPESAAALEHHHHHH
>2PG4A
GMDDETLRLQFGHLIRILPTLLEFEKKGYEPSLAEIVKASGVSEKTFFMGLKDRLIRAGLVKEET
LSYRVKTLKLTEKGRRLAECLEKCRDVLGS
>2PI2E
MGHHHHHHHHHHSSGHIEGRHMVDMMDLPRSRINAGMLAQFIDKPVCFVGRLEKIHPTGKMFILS
DGEGKNGTIELMEPLDEEISGIVEVVGRVTAKATILCTSYVQFKEDSHPFDLGLYNEAVKIIHDF
PQFYPLGIVQHD
>2PI2A
MWNSGFESYGSSSYGGAGGYTQSPGGFGSPAPSQAEKKSRARAQHIVPCTISQLLSATLVDEVFR
IGNVEISQVTIVGIIRHAEKAPTNIVYKIDDMTAAPMDVRQWVDTDDTSSENTVVPPETYVKVAG
HLRSFQNKKSLVAFKIMPLEDMNEFTTHILEVINAHMVLSKANSQPSAGRAPISNPGMSEAGNFG
GNSFMPANGLTVAQNQVLNLIKACPRPEGLNFQDLKNQLKHMSVSSIKQAVDFLSNEGHIYSTVD
DDHFKSTDAE
>2PKHH
SNAARGHRHTCKVMVLKEEAAGSERALALDMREGQRVFHSLIVHFENDIPVQIEDRFVNAQVAPD
YLKQDFTLQTPYAYLSQVAPLTEGEHVVEAILAEADECKLLQIDAGEPCLLIRRRTWSGRQPVTA
ARLIHPGSRHRLEGRFTK
>2PNHB
GSHMTNRLVLSGTVCRAPLRKVSPSGIPHCQFVLEHRSVQEAAGFHRQAWCQMPVIVSGHENQAI
THSITVGSRITVQGFISCHKAKNGLSKMVLHAEQIELIDSGD
>2Q2KB
MGSSHHHHHHSSGLVPGSHMDKKETKHLLKIKKEDYPQIFDFLENVPRGTKTAHIREALRRYIEE
IGENP
>2Q79A
TTPIVHLKGDANTLKCLRYRFKKHCTLYTAVSSTWHWTGHNVKHKSAIVTLTYDSEWQRDQFLSQ
VKIPKTITVSTGFMSIGGGTGGGSGGGS
>2QLCA
MNLKVKGARDVFEYMKGRIPDETKEHLFVLFLSTKNQILRHETITIGTLTASLIHPREIFKAAIR
ESAHSIILVHNHPSGDVQPSNADKQVTSILKKAGDLLQIELLDHVIVGNNDWFSFRDHALL
>2QPYA
CQPIFLNVLEAIEPGVVCAGHDNNQPDSFAALLSSLNELGERQLVHVVKWAKALPGFRNLHVDDQ
MAVIQYSWMGLMVFAMGWRSFTNVNSRMLYFAPDLVFNEYRMHKSRMYSQCVRMRHLSQEFGWLQ
ITPQEFLCMKALLLFSIIPVDGLKNQKFFDELRMNYIKELDRIIACKRKNPTSCSRRFYQLTKLL
DSVQPIARELHQFTFDLLIKSHMVSVDFPEMMAEIISVQVPKILSGKVKPIYFHTQ
>2QSFX
GSGNASSGALGTTGGATDAAQGGPPGSIGLTVEDLLSLRQVVSGNPEALAPLLENISARYPQLRE
HIMANPEVFVSMLLEAVGDNMQDVMEGADDMVEGEDIEVTGEAAAAGLGQGEGEGSFQVDYTPED
DQAISRLCELGFERDLVIQVYFACDKNEEAAANILFSDHAD
>2QSHA
GSSRAMGNEVAGVEDISVEIKPSSKRNSDARRTSRNVCSNEERKRRKYFHMLYLVCLMVHGFIRN
EWINSKRLSRKLSNLVPEKVFELLHPQKDEELPLRSTRKLLDGLKKCMELWQKHWKITKKYDNEG
LYMRTWKEIEMSANNKRKFKTLKRSDFLRAVSKGHGDPDISVQGFVAMLRACNVNARLIMSCQPP

DFTNMKIDTSLNGNNAYKDMVKYPIFWCEVWDKFSKKWITVDPVNLKTIEQVRLHSKLAPKGVAC
CERNMLRYVIAYDRKYGCRDVTRRYAQWMNSKVRKRRITKDDFGEKWFRKVITALHHRKRTKIDD
YEDQYFFQRDESEGIPDSVQDLKNHPYYVLEQDIKQTQIVKPGCKECGYLKVHGKVGKVLKVYAK
RDIADLKSARQWYMNGRILKTGSRCKKVIKRTVGRPKGEAEEEDERLYSFEDTELYIPPLASASG
EITKNTFGNIEVFAPTMIPGNCCLVENPVAIKAARFLGVEFAPAVTSFKFERGSTVKPVLSGIVV
AKWLREAIETAIDGIEFI
>2QUQA
KLITASSSKEYLPDLLLFWQNYEYWITNIGLYKTKQRDLTRTPANLDTDTEECMFWMNYLQKDQS
FQLMNFAMENLGALYFGSIGDISELYLRVEQYWDRRADKNHSVDGKYWDALIWSVFTMCIYYMPV
EKLAEIFSVYPLHEYLGSNKRLNWEDGMQLVMCQNFARCSLFQLKQCDFMAHPDIRLVQAYLILA
TTTFPYDEPLLANSLLTQCIHTFKNFHVDDFRPLLNDDPVESIAKVTLGRIFYRLCGCDYLQSGP
RKPIALHTEVSSLLQHAAYLQDLPNVDVYREENSTEVLYWKIISLDRDLDQYLNKSSKPPLKTLD
AIRRELDIFQYKVDSLEEDFRSNNSRFQKFIALFQISTVSWKLFKMYLIYYDTADSLLKVIHYSK
VIISLIVNNFHAKSEFFNRHPMVMQTITRVVSFISFYQIFVESAAVKQLLVDLTELTANLPTIFG
SKLDKLVYLTERLSKLKLLWDKVQLLDSGDSFYHPVFKILQNDIKIIELKNDEMFSLIKGLGSLV
PLNKLRQESLLEEEDENNTEPSDFRTIVEEFQSEYNISDILS
>2RH3A
IQVFLSARPPAPEVSKIYDNLILQYSPSKSLQMILRRALGDFENMLADGSFRAAPKSYPIPHTAF
EKSIIVQTSRMFPVSLIEAARNHFDPLGLETARAFGHKLATAALACFFAREKATNS
>2ROHA
GSPFADPNSLALANVPLSRSKRPDFGQRRIRRPFTVAEVELLVEAVEHLGTGRWRDVKFRAFENV
HHRTYVDLKDKWKTLVHTASIAPQQRRGAPVPQELLDRVLAAQAYWSVDSSGRIVTL
>2RRDA
GIPEFKQKALVAKVSQREEMVKKCLGELTEVCKSLGKVFGVHYFNIFNTVTLKKLAESLSSDPEV
LLQIDGVTEDKLEKYGAEVISVLQKYSEWTSPAEDS
>2RT6A
MKTALLLEKLEGQLATLRQRCAPVSQFATLSARFDRHLFQTRATTLQACLDEAGDNLAALRHAVE
QQQLPQVAWLAEHLAAQLEAIAREASAWSLREW
>2VL6C
MEIPSKQIDYRDVFIEFLTTFKGNNNQNKYIERINELVAYRKKSLIIEFSDVLSFNENLAYEIIN
NTKIILPILEGALYDHILQLDPTYQRDIEKVHVRIVGIPRVIELRKIRSTDIGKLITIDGILVKV
TPVKERIYKATYKHIHPDCMQEFEWPEDEEMPEVLEMPTICPKCGKPGQFRLIPEKTKLIDWQKA
VIQERPEEVPSGQLPRQLEIILEDDLVDSARPGDRVKVTGILDIKQDSPVKRGSRAVFDIYMKVS
SIEVSQKV
>2WKCD
GTIITVTAQANEKNTRTVSTAKGDKKIISVPLFEKEKGSNVKVAYGSAFLPDFIQLGDTVTVSGR
VQAKESGEYVNYNFVFPTVEKVFITNDNSSQSQAKQDLFGGSEPIEVNSEDLPF
>2WP0D
MDTNNNIEKEILALVKQNPKVSLIEYENYFSQLKYNPNASKSDIAFFYAPNQVLCTTITAKYGAL
LKEILSQNKVGMHLAHSVDVRIEVAPKIQINAQSNINYKAIKTSVKD
>2WP0B
MKNFYDWIKEFIRDQGEFIAQQSGWLELERSSYAKLIAQTISHVLNGGSLLVSADSSRHWFLNYI
LSNLNPKDLKERPLLSVIDFNASSFYPKNDANLSLATIEMTYQNPMFWHVGKIENEGLKTILLSK
IPSFLWLFEELKEDCLLLKEHDSLLDYKLLQLFKLFENALFSVLYNKVTL

>2XE0B
NTKYNKEFLLYLAGFVDGDGSIIAQIKPRASNKFAHQLSLTFAVTQKTQRRWFLDKLVDEIGVGY
VYDSGSVSDYRLSEIKPLHNFLTQLQPFLKLKQKQANLVLAIIEQLPSAKASPDAFLEVCTWVDQ
IAALNDSKTRATTSATVRAALD
>2XIWB
MRGSHHHHHHGSVKVKFLLNGEEKEVDTSKIRDVSRQGKNVKFLYNDNGKYGAGNVDEKDAPKEL
LDMLARAEREKKLN
>2XMAA
GSHMTYVILPLEMKKGRGYVYQLEYHLIWCVKYRHQVLVGEVADGLKDILRDIAAQNGLEVITME
VMPDHVHLLLSATPQQAIPDFVKALKGASARRMFVAYPQLKEKLWGGNLWNPSYCILTVSENTRA
QIQKYIESQHDKE
>2YPFA
VDLRTLAYSQQQQEKIKPKVRSTVAQHHEALVAHAFTHAHIVALSQHPAALATVAVKYQDMIAAL
PEATHEAIVAVAKQWSGARALEALLTVAGELRGPPLQLDTGQLLKIAKRGGVTAVEAVHAWRNAL
TGAPLNLTPEQVVAIASHDGGKQALETVQRLLPVLCQAHGLTPQQVVAIASNGGGKQALETVQRL
LPVLCQAHGLTPEQVVAIASNIGGKQALETVQALLPVLCQAHGLTPQQVVAIASNGGGKQALETV
QRLLPVLCQAHGLTPEQVVAIASNIGGKQALETVQALLPVLCQAHGLTPEQVVAIASNIGGKQAL
ETVQALLPVLCQAHGLTPEQVVAIASNIGGKQALETVQALLPVLCQAHGLTPEQVVAIASHDGGK
QALETVQRLLPVLCQAHGLTPEQVVAIASHDGGKQALETVQRLLPVLCQAHGLTPQQVVAIASNG
GGKQALETVQRLLPVLCQAHGLTPEQVVAIASNIGGKQALETVQALLPVLCQAHGLTPEQVVAIA
SNIGGKQALETVQALLPVLCQAHGLTPEQVVAIASHDGGKQALETVQRLLPVLCQAHGLTPEQVV
AIASHDGGKQALETVQRLLPVLCQAHGLTPEQVVAIASHDGGKQALETVQRLLPVLCQAHGLTPQ
QVVAIASNGGGKQALETVQRLLPVLCQAAGLTPEQVVAIASHDGGKQALETVQRLLPVLCQAHGL
TPQQVVAIASNGGGRPALESIVAQLSRPDPGSSAALEHHHHHH
>2YRQA
GSSGSSGMGKGDPKKPRGKMSSYAFFVQTCREEHKKKHPDASVNFSEFSKKCSERWKTMSAKEKG
KFEDMAKADKARYEREMKTYIPPKGETKKKFKDPNAPKRPPSAFFLFCSEYRPKIKGEHPGLSIG
DVAKKLGEMWNNTAADDKQPYEKKAAKLKEKYEKDIAAYRAKG
>2YVAB
MQERIKACFTESIQTQIAAAEALPDAISRAAMTLVQSLLNGNKILCCGNGTSAANAQHFAASMIN
RFETERPSLPAIALNTDNVVLTAIANDRLHDEVYAKQVRALGHAGDVLLAISTRGNSRDIVKAVE
AAVTRDMTIVALTGYDGGELAGLLGPQDVEIRIPSHRSARIQEMHMLTVNCLCDLIDNTLFPHQD
D
>2Z3XC
AKLLIPQAASAIEQMKLEIASEFGVQLGAETTSRANGSVGGEITKRLVRLAQQNMGGQFHGQQ
>2Z4RC
MKERILQEIKTRVNRKSWELWFSSFDVKSIEGNKVVFSVGNLFIKEWLEKKYYSVLSKAVKVVLG
NDATFEITYEAFEPHSSYSEPLVKKRAVLLTPLNPDYTFENFVVGPGNSFAYHAALEVAKHPGRY
NPLFIYGGVGLGKTHLLQSIGNYVVQNEPDLRVMYITSEKFLNDLVDSMKEGKLNEFREKYRKKV
DILLIDDVQFLIGKTGVQTELFHTFNELHDSGKQIVICSDREPQKLSEFQDRLVSRFQMGLVAKL
EPPDEETRKSIARKMLEIEHGELPEEVLNFVAENVDDNLRRLRGAIIKLLVYKETTGKEVDLKEA
ILLLKDFIKPNRVKAMDPIDELIEIVAKVTGVPREEILSNSRNVKALTARRIGMYVAKNYLKSSL
RTIAEKFNRSHPVVVDSVKKVKDSLLKGNKQLKALIDEVIGEISRRALSG
>2ZDSF

MPRNFTLFTGQWADLPLEEVCRLARDFGYDGLELACWGDHFEVDKALADPSYVDSRHQLLDKYGL
KCWAISNHLVGQAVCDAIIDERHEAILPARIWGDGDAEGVRQRAAAEIKDTARAAARLGVDTVIG
FTGSAIWHLVAMFPPAPESMIERGYQDFADRWNPILDVFDAEGVRFAHEVHPSEIAYDYWTTHRA
LEAVGHRPAFGLNFDPSHFVWQDLDPVGFLWDFRDRIYHVDCKEARKRLDGRNGRLGSHLPWGDP
RRGWDFVSAGHGDVPWEDVFRMLRSIDYQGPVSVEWEDAGMDRLQGAPEALTRLKAFDFEPPSAS
FDAAFNSLEHHHHHH

>2ZQEA

MREVKEVDLRGLTVAEALLEVDQALEEARALGLSTLRLLHGKGTGALRQAIREALRRDKRVESFA
DAPPGEGGHGVTVVALRP

>2ZTCD

MASMTGGQQMGRGSEFMIASVRGEVLEVALDHVVIEAAGVGYRVNATPATLATLRQGTEARLITA
MIVREDSMTLYGFPDGETRDLFLTLLSVSGVGPRLAMAALAVHDAPALRQVLADGNVAALTRVPG
IGKRGAERMVLELRDKVGVAATGGALSTNGHAVRSPVVEALVGLGFAAKQAEEATDTVLAANHDA
TTSSALRSALSLLGKAR

>3AL2A

GPLGSLKKQYIFQLSSLNPQERIDYCHLIEKLGGLVIEKQCFDPTCTHIVVGHPLRNEKYLASVA
AGKWVLHRSYLEACRTAGHFVQEEDYEWGSSSILDVLTGINVQQRRLALAAMRWRKKIQQRQESG
IVEGAFSGWKVILHVDQSREAGFKRLLQSGGAKVLPGHSVPLFKEATHLFSDLNKLKPDDSGVNI
AEAAAQNVYCLRTEYIADYLMQESPPHVENYCLPEAISFI

>3AQQD

MSSEPPPPPQPPTHQASVGLLDTPRSRERSPSPLRGNVVPSPLPTRRTRTFSATVRASQGPVYKG
VCKCFCRSKGHGFITPADGGPDIFLHISDVEGEYVPVEGDEVTYKMCSIPPKNEKLQAVEVVITH
LAPGTKHETWSGHVISS

>3ASKA

SLYKVNEYVDARDTNMGAWFEAQVVRVTRKAPSRPALEEDVIYHVKYDDYPENGVVQMNSRDVRA
RARTIIKWQDLEVGQVVMLNYNPDNPKERGFWYDAEISRKRETRTARELYANVVLGDDSLNDCRI
IFVDEVFKIERPGEGSPMVDNPMRRKSGPSCKHCKDDVNRLCRVCACHLCGGRQDPDKQLMCDEC
DMAFHIYCLDPPLSSVPSEDEWYCPECRNDA

>3AXJB

MPKNGGAGHRNTAPRKRQIPAAQLDEDSPIVQQFRIYSNELIMKHDRHERIVKLSRDITIESKRI
IFLLHSIDSRKQNKEKVLEEARQRLNKLIAVNFRAVALELRDQDVYQFRSSYSPGLQEFIQAYTY
MEYLCHEDAEGENETKSVSDWQAIQAVMQYVEESSQPKEEPTEGEDVQAIAQVESPKKFQFFVDP
TEYILGLSDLTGELMRRCINSLGSGDTDTCLDTCKALQHFYSGYISLNCQRARELWRKITTMKQS
VLKAENVCYNVKVRGGEAAKWGATFDQKPADEVDEGFY

>3B0CT

GSTREPEIASSLIKQIFSHYVKTPVTRDAYKIVEKCSERYFKQISSDLEAYSQHAGRKTVEMADV
ELLMRRQGLVTDKMPLHVLVERHLPLEYRKLLIPIAVSGNKVIPCK

>3BOSB

GMRSNRVTQHPPLQLSLPVHLPDDETFTSYYPAAGNDELIGALKSAASGDGVQAIYLWGPVKSGR
THLIHAACARANELERRSFYIPLGIHASISTALLEGLEQFDLICIDDVDAVAGHPLWEEAIFDLY
NRVAEQKRGSLIVSASASPMEAGFVLPDLVSRMHWGLTYQLQPMMDDEKLAALQRRAAMRGLQLP
EDVGRFLLNRMARDLRTLFDVLDRLDKASMVHQRKLTIPFVKEMLRL

>3BRFA

SGPLGSGDSVQSLTSDRMIDFLSNKEKYECVISIFHAKVAQKSYGNEKRFFCPPPCIYLIGQGWK

LKKDRVAQLYKTLKASAQKDAAIENDPIHEQQATELVAYIGIGSDTSERQQLDFSTGKVRHPGDQ
RQDPNIYDYCAAKTLYISDSDKRKYFDLNAQFFYGCGMEIGGFVSQRIKVISKPSKKKQSMKNTD
CKYLCIASGTKVALFNRLRSQTVSTRYLHVEGNAFHASSTKWGAFTIHLFDDERGLQETDNFAVR
DGFVYYGSVVKLVDSVTGIALPRLRIRKVDKQQVILDASCSEEPVSQLHKCAFQMIDNELVYLCL
SHDKIIQHQATAINEHRHQINDGAAWTIISTDKAEYRFFEAMGQVANPISPCPVVGSLEVDGHGE
ASRVELHGRDFKPNLKVWFGATPVETTFRSEESLHCSIPPVSQVRNEQTHWMFTNRTTGDVEVPI
SLVRDDGVVYSSGLTFSYKSLER

>3BS3A

SNAMSNNQQMMLNRIKVVLAEKQRTNRWLAEQMGKSENTISRWCSNKSQPSLDMLVKVAELLNVD
PRQLINGKIKI

>3BTPB

MVIIKLNANKNMPVLAVEKPQEIHKEELSDHHQSNGFTSLDLEMIELENFVLHCPLPEENLAG

>3BTPA

MDPKAEGNGENITETAAGNVETSDFVNLKRQKREGVNSTGMSEIDMTGSQETPEHNMHGSPTHTD
DLGPRLDADMLDSQSSHVSSSAQGNRSEVENELSNLFAKMALPGHDRRTDEYILVRQTGQDKFAG
TTKCNLDHLPTKAEFNASCRLYRDGVGNYYPPPLAFERIDLPEQLAAQLHNLEPREQSKQCFQYK
LEVWNRAHAEMGITGTDIFYQTDKNIKLDRNYKLRPEDRYIQTEKYGRREIQKRYEHQFQAGSLL
PDILIKTPQNDIHFSYRFAGDAYANKRFEEFERAIKTKYGSDTEIKLKSKSGIMHDSKYLESWER
GSADIRFAEFAGENRAHNKQFPAATVNMGRQPDGQGGMTRDRHVSVDYLLQNLPNSPWTQALKEG
KLWDRVQVLARDGNRYMSPSRLEYSDPEHFTQLMDQVGLPVSMGRQSHANSVKFEQFDRQAAVIV
ADGPNLREVPDLSPEKLQQLSQKDVLIADRNEKGQRTGTYTNVVEYERLMMKLPSDAAQLLAEPS
DRYSRAFVRPEPALPPISDSRRTYESRPRGPTVNSL

>3BU8B

GAGEARLEEAVNRWVLKFYFHEALRAFRGSRYGDFRQIRDIMQALLVRPLGKEHTVSRLLRVMQC
LSRIEEGENLDCSFDMEAELTPLESAINVLEMIKTEFTLTEAVVESSRKLVKEAAVIICIKNKEF
EKASKILKKHMSKDPTTQKLRNDLLNIIREKNLAHPVIQNFSYETFQQKMLRFLESHLDDAEPYL
LTMAKKALKSESAASSTGKEDKQPAPGPVEKPPREPARQL

>3C1DB

GPAYARLLDRAVRILAVRDHSEQELRRKLAAPIMGKNGPEEIDATAEDYERVIAWCHEHGYLDDS
RFVARFIASRSRKGYGPARIRQELNQKGISREATEKAMREADIDWAALARDQATRKYGEPLPTVF
SEKVKIQRFLLYRGYLMEDIQDIWRNFAD

>3C1YB

MGSSHHHHHHSSGLVPRGSHMGVKSLVPQELIEKIKLISPGTELRKALDDIINANFGALIFLVDD
PKKYEDVIQGGFWLDTDFSAEKLYELSKMDGAIVLSEDITKIYYANVHLVPDPTIPTGETGTRHR
TAERLAKQTGKVVIAVSRRRNIISLYYKNYKYVVNQVDFLISKVTQAISTLEKYKDNFNKLLSEL
EVLELENRVTLADVVRTLAKGFELLRIVEEIRPYIVELGEEGRLARMQLRELTEDVDDLLVLLIM
DYSSEEVEEETAQNILQDFITRREPSPISISRVLGYDVQQAAQLDDVLVSARGYRLLKTVARIPL
SIGYNVVRMFKTLDQISKASVEDLKKVEGIGEKRARAISESISSLKHRKTSE

>3CNBC

MSLNVKNDFSILIIEDDKEFADMLTQFLENLFPYAKIKIAYNPFDAGDLLHTVKPDVVMLDLMMV
GMDGFSICHRIKSTPATANIIVIAMTGALTDDNVSRIVALGAETCFGKPLNFTLLEKTIKQLVEQ
KKATSEGHHHHHH

>3D6WB

GKSVVTLKTTDGWIPVPFSKVMYLEAKDKKTYVNAEELTGTHKYSLQEFEYLLPKDSFIRCHRSF

IVNVNHIKAIYPDTHSTFLLSMDNGERVPVSQSYASYFRKLLGFGS
>3DPJA
SNAMVQAQTRDQIVAAADELFYRQGFAQTSFVDISAAVGISRGNFYYHFKTKDEILAEVIRLRLA
RTAQMLADWQGTGDSPRARIASFIDLMIMNRAKITRYGCPVGSLCTELSKLDHAAQGQANGLFTL
FRDWLQRQFAEAGCTTEAPALAMHLLARSQGAATLAQSFHDEGFLRSEVADMHRWLDNTLPMTT
>3EI4F
MHHHHHHRRLVPRGSGGRQKTSEIVLRPRNKRSRSPLELEPEAKKLCAKGSGPSRRCDSDCLWVG
LAGPQILPPCRSIVRTLHQHKLGRASWPSVQQGLQQSFLHTLDSYRILQKAAPFDRRATSLAWHP
THPSTVAVGSKGGDIMLWNFGIKDKPTFIKGIGAGGSITGLKFNPLNTNQFYASSMEGTTRLQDF
KGNILRVFASSDTINIWFCSLDVSASSRMVVTGDNVGNVILLNMDGKELWNLRMHKKKVTHVALN
PCCDWFLATASVDQTVKIWDLRQVRGKASFLYSLPHRHPVNAACFSPDGARLLTTDQKSEIRVYS
ASQWDCPLGLIPHPHRHFQHLTPIKAAWHPRYNLIVVGRYPDPNFKSCTPYELRTIDVFDGNSGK
MMCQLYDPESSGISSLNEFNPMGDTLASAMGYHILIWSQEEARTRK
>3EIVD
MAGETVITVVGNLVDDPELRFTPSGAAVAKFRVASTPRTFDRQTNEWKDGESLFLTCSVWRQAAE
NVAESLQRGMRVIVQGRLKQRSYEDREGVKRTVYELDVDEVGASLRSATAKVTKTSGQGRGGQGG
YGGGGGGQGGGGWGGGPGGGQQGGGAPADDPWATGGAPAGGQQGGGGQGGGGWGGGSGGGGGYSD
EPPF
>3EQXB
GMEWQAEQAYNHLPPLPLDSKLAELAETLPILKACIPARAALAELKQAGELLPNQGLLINLLPLL
EAQGSSEIENIVTTTDKLFQYAQEDSQADPMTKEALRYRTALYQCFTQLSNRPLCVTTALEICST
IKSVQMDVRKVPGTSLTNQATGEVIYTPPAGESVIRDLLSNWEAFLHNQDDVDPLIKMAMAHYQF
EAIHPFIDGNGRTGRVLNILYLIDQQLLSAPILYLSRYIVAHKQDYYRLLLNVTTQQEWQPWIIF
ILNAVEQTAKWTTHKIAAARELIAHTTEYVRQQLPKIYSHELVQVIFEQPYCRIQNLVESGLAKR
QTASVYLKQLCDIGVLEEVQSGKEKLFVHPKFVTLMTKDSNQFSRYAL
>3EUSB
QAMTKTLRTPEHVYLCQRLRQARLDAGLTQADLAERLDKPQSFVAKVETRERRLDVIEFAKWMAA
CEGLDVVSEIVATIAEGRAQA
>3EYIB
HMASPQFSQQREEDIYRFLKDNGPQRALVIAQALGMRTAKDVNRDLYRMKSRHLLDMDEQSKAWT
IYRWTIY
>3EZ2A
MSDSSQLHKVAQRANRMLNVLTEQVQLQKDELHANEFYQVYAKAALAKLPLLTRANVDYAVSEME
EKGYVFDKRPAGSSMKYAMSIQNIIDIYEHRGVPKYRDRYSEAYVIFISNLKGGVSKTVSTVSLA
HAMRAHPHLLMEDLRILVIDLDPQSSATMFLSHKHSIGIVNATSAQAMLQNVSREELLEEFIVPS
VVPGVDVMPASIDDAFIASDWRELCNEHLPGQNIHAVLKENVIDKLKSDYDFILVDSGPHLDAFL
KNALASANILFTPLPPATVDFHSSLKYVARLPELVKLISDEGCECQLATNIGFMSKLSNKADHKY
CHSLAKEVFGGDMLDVFLPRLDGFERCGESFDTVISANPATYVGSADALKNARIAAEDFAKAVFD
RIEFIRSN
>3F1ZA
GASKAFYSAGDKLFQPGDDAVASMQTYSVAQFLQPFTLNPAKASSDYLGKWVKVRGVIVDIRRKS
GIAGSYYFIVTMRDEQNKTDKRLTFNFGSHNSADVEALSNGSVATIVGQVHQVQDSTIPTLQNPK
VVK
>3F6CB

SLNAIIIDDHPLAIAAIRNLLIKNDIEILAELTEGGSAVQRVETLKPDIVIIDVDIPGVNGIQVL
ETLRKRQYSGIIIIVSAKNDHFYGKHCADAGANGFVSKKEGMNNIIAAIEAAKNGYCYFPFSLNR
FVGS
>3FDQB
MPKSEIRKLLQEIKKQVDNPGNSSTTEIKKMASEAGIDEQTAEEIYHLLTEFYQAVEEHGGIEKY
MHSNISWLKIELELLSACYQIAILEDMKVLDISEMLSLNDLRIFPKTPSQLQNTYYKLKKELIQV
EDIPKNKPGRKRKTQKNTKKEKTNIFGKVVPALEHHHHHH
>3FDQA
MPKSEIRKLLQEIKKQVDNPGNSSTTEIKKMASEAGIDEQTAEEIYHLLTEFYQAVEEHGGIEKY
MHSNISWLKIELELLSACYQIAILEDMKVLDISEMLSLNDLRIFPKTPSQLQNTYYKLKKELIQV
EDIPKNKPGRKRKTQKNTKKEKTNIFGKVVPALEHHHHHH
>3FHWB
MNTLELSARVLECGAMRHTPAGLPALELLLVHESEVVEAGHPRRVELTISAVALGDLALLLADTP
LGTEMQVQGFLAPARKDSVKVKLHLQQARRIAGSMGRDPLVGLEHHHHHH
>3FRQA
GMPRPKLKSDDEVLEAATVVLKRCGPIEFTLSGVAKEVGLSRAALIQRFTNRDTLLVRMMERGVE
QVRHYLNAIPIGAGPQGLWEFLQVLVRSMNTRNDFSVNYLISWYELQVPELRTLAIQRNRAVVEG
IRKRLPPGAPAAAELLLHSVIAGATMQWAVDPDGELADHVLAQIAAILCLMFPEHDDFQLLQAHA
>3FYMA
MKTVGEALKGRRERLGMTLTELEQRTGIKREMLVHIENNEFDQLPNKNYSEGFIRKYASVVNIEP
NQLIQAHQDEIPSNQAEWDEVITVFNNNKDLDYKSKSKEPIQLLVIMGITVLITLLLWIMLVLIF
>3G1CA
MNNKLKTQAVEQLFQAILSLKDLDEAYDFFEDVCTINEILSLSQRFEVAKMLREHRTYLDIAEKT
GASTATISRVNRSLNYGNDGYDRVFERLGMLEKESEDNK
>3GIOB
MGSSHHHHHHSSGLVPRGSHMPNTSQRNSFLQDVPYWMLQNRSEYITQGVDSSHIVDGKKTEEIE
KIATKRATIRVAQNIVHKLKEAYLSKTNRIKQKITNEMFIQMTQPIYDSLMNVDRLGIYINPNNE
EVFALVRARGFDKDALSEGLHKMSLDNQAVSILVAKVEEIFKDSVNYGDVKVPIAM
>3GN5B
GHMKCPVCHQGEMVSGIKDIPYTFRGRKTVLKGIHGLYCVHCEESIMNKEESDAFMAQVKAFRAS
VNAETVAPEFIVKVRKKLSLTQKEASEIFGGGVNAFSRYEKGNAQPHPSTIKLLRVLDKHPELLN
EIR
>3GVAB
MRMDEFYTKVYDAVCEIPYGKVSTYGEIARYVGMPSYARQVGQAMKHLHPETHVPWHRVINSRGT
ISKRDISAGEQRQKDRLEEEGVEIYQTSLGEYKLNLPEYMWKPGSHHHHHH
>3GXQB
ENSVFFGKKKKVSLHLLVDPDMKDEIIKYAQEKDFDNVSQAGREILKKGLEQIA
>3GYDB
MGSDKIHHHHHHENLYFQGMYPDLVHLGGADKYFEEILEIVNKIKLFGDFSNEEVRYLCSYMQCY
AAPRDCQLLTEGDPGDYLLLILTGEVNVIKDIPNKGIQTIAKVGAGAIIGEMSMIDGMPRSASCV
ASLPTDFAVLSRDALYQLLANMPKLGNKVLIRLLQLLTARFRESYDRILPKTLGELI
>3GZ5B
GSHMTEAEYLANYDPKAFKAQLLTVDAVLFTYHDQQLKVLLVQRSNHPFLGLWGLPGGFIDETCD
ESLEQTVLRKLAEKTAVVPPYIEQLCTVGNNSRDARGWSVTVCYTALMSYQACQIQIASVSDVKW

WPLADVLQMPLAFDHLQLIEQARERLTQKALYSLVPGFALSEPFTLPELQHVHEVLLGKPIQGKS
FRRRVEQADLLIDTGLKRTERGRPANLYCLKPDTASYRFLRNLEC
>3H4LB
GSMTQIHQINDIDVHRITSGQVITDLTTAVKELVDNSIDANANQIEIIFKDYGLESIECSDNGDG
IDPSNYEFLALKHYTSKIAKFQDVAKVQTLGFRGEALSSLCGIAKLSVITTTSPPKADKLEYDMV
GHITSKTTTSRNKGTTVLVSQLFHNLPVRQKEFSKTFKRQFTKCLTVIQGYAIINAAIKFSVWNI
TPKGKKNLILSTMRNSSMRKNISSVFGAGGMRGLEEVDLVLDLNPFKNRMLGKYTDDPDFLDLDY
KIRVKGYISQNSFGCGRNSKDRQFIYVNKRPVEYSTLLKCCNEVYKTFNNVQFPAVFLNLELPMS
LIDVNVTPDKRVILLHNERAVIDIFKTTLSDYYNRQELALPK
>3HI2D
GSHMEKRTPHTRLSQVKKLVNAGQVRTTRSALLNADELGLDFDGMCNVIIGLSESDFYKSMTTYS
DHTIWQDVYRPRLVTGQVYLKITVIHDVLIVSFKEK
>3HOSA
MSSFVPNKEQTRTVLIFCFHLKKTAAESHRMLVEAFGEQVPTVKTCERWFQRFKSGDFDVDDKEH
GKPPKRYEDAELQALLDEDDAQTQKQLAEQLEVSQQAVSNRLREMGKIQKVGRWVPHELNERQME
RRKNTCEILLSRYKRKSFLHRIVTGDEKWIFFVNPKRKKSYVDPGQPATSTARPNRFGKKTMLCV
WWDQSGVIYYELLKPGETVNAARYQQQLINLNRALQRKRPEYQKRQHRVIFLHDNAPSHTARAVR
DTLETLNWEVLPHAAYSPDLAPSDYHLFASMGHALAEQRFDSYESVKKWLDEWFAAKDDEFYWRG
IHKLPERWEKCVASDGKYFE
>3HTAD
MGSSHHHHHHSSGLVPRGSHMPRRHDPERRQRIIDAAIRVVGQKGIAGLSHRTVAAEADVPLGST
TYHFATLDDLMVAALRQANEGFARVVAAHPALSDPEADLSGELARVLGEWLGGDRTGVELEYELY
LAALRRPALRPVAAEWAEGVGALLAARTDPTTARALVAVLDGICLQVLLTDTPYDEEYAREVLTR
LIPVPATRDGRGPGSHPPATAG
>3I54D
MGSSHHHHHHSSGLGGTENLYFQSHMDEILARAGIFQGVEPSAIAALTKQLQPVDFPRGHTVFAE
GEPGDRLYIIISGKVKIGRRAPDGRENLLTIMGPSDMFGELSIFDPGPRTSSATTITEVRAVSMD
RDALRSWIADRPEISEQLLRVLARRLRRTNNNLADLIFTDVPGRVAKQLLQLAQRFGTQEGGALR
VTHDLTQEEIAQLVGASRETVNKALADFAHRGWIRLEGKSVLISDSERLARRAR
>3II2A
GSEMSVVEYEVVSKNLTSKMSHELLFSVKKRWFVKPFRHDRQLGKLHYKLLPGNYIKFGLYVLKN
QDYARFEIAWVHVDKDGKIEERTVYSIETYWHIFIDIENDLNCPYVLAKFIEMRPEFHKTAWVEE
SNYSIAEDDIQMVESIKRYLERKIASD
>3II6X
GAMGSKISNIFEDVEFCVMSGTDSQPKPDLENRIAEFGGYIVQNPGPDTYCVIAGSENIRVKNII
LSNKHDVVKPAWLLECFKTKSFVPWQPRFMIHMCPSTKEHFAREYDCYGDSYFIDTDLNQLKEVF
SGIKNSNEQTPEEMASLIADLEYRYSWDCSPLSMFRRHTVYLDSYAVINDLSTKNEGTRLAIKAL
ELRFHGAKVVSCLAEGVSHVIIGEDHSRVADFKAFRRTFKRKFKILKESWVTDSIDKCELQEENQ
YLI
>3II6A
MERKISRIHLVSEPSITHFLQVSWEKTLESGFVITLTDGHSAWTGTVSESEISQEADDMEMEKGK
YVGELRKALLSGAGPADVYTFNFSKESCYFFFEKNLKDVSFRLGSFNLEKVENPAEVIRELICYC
LDTTAENQAKNEHLQKENERLLRDWNDVQGRFEKCVSAKEALETDLYKRFILVLNEKKTKIRSLH
NKLLNAAQ

>3IO5B

GSHMDVVRTKIPMMNIALSGEITGGMQSGLLILAGPSKSFKSNFGLTMVSSYMRQYPDAVCLFYD
SEFGITPAYLRSMGVDPERVIHTPVQSLEQLRIDMVNQLDAIERGEKVVVFIDSLGNLASKKETE
DALNEKVVSDMTRAKTMKSLFRIVTPYFSTKNIPCIAINHTYETQEMFSKTVMGGGTGPMYSADT
VFIIGKRQIKDGSDLQGYQFVLNVEKSRTVKEKSKFFIDVKFDGGIDPYSGLLDMALELGFVVKP
KNGWYAREFLDEETGEMIREEKSWRAKDTNCTTFWGPLFKHQPFRDAIKRAYQLGAIDSNEIVEA
EVDELINS

>3IV5B

MFEQRVNSDVLTVSTVNSQDQVTQKPLRDSVKQALKNYFAQLNGQDVNDLYELVLAEVEQPLLDM
VMQYTRGNQTRAALMMGINRGTLRKKLKKYGMN

>3IVPD

MRKKEDKYDFRALGLAIKEARKKQGLTREQVGAMIEIDPRYLTNIENKGQHPSLQVLYDLVSLLN
VSVDEFFLPASSQVKSTKRRQLENKIDNFTDADLVIMESVADGIVKSKEVGEMAGENLYFQ

>3JU0A

MSLTDSKVKNAKSLEKEYKLTDGFGMHLLVHPNGSKYWRLSYRFEKKQRLLALGVYPAVSLADAR
QRRDEAKKLLAAGIDPSAKKQADNKTIQEKRNNTRLEHHHHHH

>3K2AB

GSGIFPKVATNIMRAWLFQHLTHPYPSEEQKKQLAQDTGLTILQVNNWFINARRRIVQPMIDQSN
RA

>3K4XA

GPHMASMLEAKFEEASLFKRIIDGFKDCVQLVNFQCKEDGIIAQAVDDSRVLLVSLEIGVEAFQE
YRCDHPVTLGMDLTSLSKILRCGNNTDTLTLIADNTPDSIILLFEDTKKDRIAEYSLKLMDIDAD
FLKIEELQYDSTLSLPSSEFSKIVRDLSQLSDSINIMITKETIKFVADGDIGSGSVIIKPFVDME
HPETSIKLEMDQPVDLTFGAKYLLDIIKGSSLSDRVGIRLSSEAPALFQFDLKSGFLQFFLAPKF
NDEGSNSQSNGSGALEAKFEEASLFKRIIDGFKDCVQLVNFQCKEDGIIAQAVDDSRVLLVSLEI
GVEAFQEYRCDHPVTLGMDLTSLSKILRCGNNTDTLTLIADNTPDSIILLFEDTKKDRIAEYSLK
LMDIDADFLKIEELQYDSTLSLPSSEFSKIVRDLSQLSDSINIMITKETIKFVADGDIGSGSVII
KPFVDMEHPETSIKLEMDQPVDLTFGAKYLLDIIKGSSLSDRVGIRLSSEAPALFQFDLKSGFLQ
FFLAPKFNDEGSNSQASNSGALEAKFEEASLFKRIIDGFKDCVQLVNFQCKEDGIIAQAVDDSRV
LLVSLEIGVEAFQEYRCDHPVTLGMDLTSLSKILRCGNNTDTLTLIADNTPDSIILLFEDTKKDR
IAEYSLKLMDIDADFLKIEELQYDSTLSLPSSEFSKIVRDLSQLSDSINIMITKETIKFVADGDI
GSGSVIIKPFVDMEHPETSIKLEMDQPVDLTFGAKYLLDIIKGSSLSDRVGIRLSSEAPALFQFD
LKSGFLQFFLAPKFNDEE

>3KDEC

MKYCKFCCKAVTGVKLIHVPKCAIKRKLWEQSLGCSLGENSQICDTHFNDSQWKAAPAKGQTFKR
RRLNADAVPSKV

>3KHKB

MSLDIEQQFLNDLDNQLWRAADKLRSNLDAANYKHVVLGLIFLKYVSDAFEERQQELTELFQKDD
DDNIYYLPREDYDSDEAYQQAIAEELEIGDYYTEKNVFWVPKTARWNKLRDVITLPTGSVIWQDE
QGEDVKLRSVSWLIDNAFDDIEKANPKLKGILNRISQYQLDADKLIGLINEFSLTSFNNPEYNGE
KLNLKSKDILGHVYEYFLGQFALAEGKQGGQYYTPKSIVTLIVEMLEPYKGRVYDPAMGSGGFFV
SSDKFIEKHANVKHYNASEQKKQISVYGQESNPTTWKLAAMNMVIRGIDFNFGKKNADSFLDDQH
PDLRADFVMTNPPFNMKDWWHEKLADDPRWTINTNGEKRILTPPTGNANFAWMLHMLYHLAPTGS
MALLLANGSMSSNTNNEGEIRKTLVEQDLVECMVALPGQLFTNTQIPACIWFLTKDKNAKNGKRD

RRGQVLFIDARKLGYMKDRVLRDFKDEDIQKLADTFHNWQQEWSEENNQAGFCFSADLALIRKND
FVLTPGRYVGAEAEEDEGHHHHHH
>3KJOA
MSLVPATNYIYTPLNQLKGGTIVNVYGVVKFFKPPYLSKGTDYCSVVTIVDQTNVKLTCLLFSGN
YEALPIIYKNGDIVRFHRLKIQVYKKETQGITSSGFASLTFEGTLGAPIIPRTSSKYFNFTTEDH
KMVEALRVWASTHMSPSWTLLKLCDVQPMQYFDLTCQLLGKAEVDGASFLLKVWDGTRTPFPSWR
VLIQDLVLEGDLSHIHRLQNLTIDILVYDNHVHVARSLKVGSFLRIYSLHTKLQSMNSENQTMLS
LEFHLHGGTSYGRGIRVLPESNSDVDQLKKDLESANLTA
>3KOJB
MGHHHHHHSHMNSCILQATVVEAPQLRYAQDNQTPVAEMVVQFPGLSSKDAPARLKVVGWGAVAQ
ELQDRCRLNDEVVLEGRLRINSLLKPDGNREKQTELTVTRVHH
>3LDAA
MSQVQEQHISESQLQYGNGSLMSTVPADLSQSVVDGNGNGSSEDIEATNGSGDGGGLQEQAEAQG
EMEDEAYDEAALGSFVPIEKLQVNGITMADVKKLRESGLHTAEAVAYAPRKDLLEIKGISEAKAD
KLLNEAARLVPMGFVTAADFHMRRSELICLTTGSKNLDTLLGGGVETGSITELFGEFRTGKSQLC
HTLAVTCQIPLDIGGGEGKCLYIDTEGTFRPVRLVSIAQRFGLDPDDALNNVAYARAYNADHQLR
LLDAAAQMMSESRFSLIVVDSVMALYRTDFSGRGELSARQMHLAKFMRALQRLADQFGVAVVVTN
QVVAQVDGGMAFNPDPKKPIGGNIMAYSSTTRLGFKKGKGCQRLCKVVDSPCLPEAECVFAIYED
GVGDPREEDE
>3LHKD
SNAKIIGYARVSFNAQKDDLERQIQLIKSYAEENGWDIQILKDIGSGLNEKRKNYKKLLKMVMNR
KVEKVIIAYPDRLTRFGFETLKEFFKSYGTEIVIINKKHKTPQEELVEDLITIVSHFAGKLYGMH
SHKYKKLTKTVKEIVREEDAKEKE
>3M03C
MSNIGIRDLAVQFSCIEAVNMASKILKSYESSLPQTQQVDLDLSRPLFTSAALLSACKILKLKVD
KNKMVATSGVKKAIFDRLCKQLEKIGQQVD
>3M8EB
MGSSHHHHHHSSGLVPRGSHMNRDHFYTLNIAEIAERIGNDDCAYQVLMAFINENGEAQMLNKTA
VAEMIQLSKPTVFATVNSFYCAGYIDETRVGRSKIYTLSDLGVEIVECFKQKAMEMRNL
>3MAJA
GHMDVGERSSDQGTTVLTEAQRIDWMRLIRAENVGPRTFRSLINHFGSARAALERLPELARRGGA
ARAGRIPSEDEARREIEAGRRIGVELVAPGETGYPTRLATIDDAPPLLGVHALPEALAVMARPMI
AIVGSRNASGAGLKFAGQLAADLGAAGFVVISGLARGIDQAAHRASLSSGTVAVLAGGHDKIYPA
EHEDLLLDIIQTRGAAISEMPLGHVPRGKDFPRRNRLISGASVGVAVIEAAYRSGSLITARRAAD
QGREVFAVPGSPLDPRAAGTNDLIKQGATLITSASDIVEAVASILERPIELPGREPEHAPPEGEP
DTGDRTRILALLGPSPVGIDDLIRLSGISPAVVRTILLELELAGRLERHGGSLVSLS
>3MKYB
MGSSHHHHHHSSGLVPRGSHYRPTSAYERGQRYASRLQNEFAGNISALADAENISRKIITRCINT
AKLPKSVVALFSHPGELSARSGDALQKAFTDKEELLKQQASNLHEQKKAGVIFEADEVITLLTSV
LKTSSASRTSLSSRHQFAPGATVLYKGDKMVLNLDRSRVPTECIEKIEAILKELEKPAP
>3MU6D
GRKKIQITRIMDERNRQVTFTKRKFGLMKKAYELSVLCDCEIALIIFNSSNKLFQYASTDMDKVL
LKYTAY
>3MUJB

SMEATPCIKAISPSEGWTTGGATVIIIGDNFFDGLQVVFGTMLVWSELITPHAIRVQTPPRHIPG
VVEVTLSYKSKQFCKGAPGRFVYTALNEPTIDYGFQRLQKVIPRHPGDPERLPKEVLLKRAADLV
EALYGMPH
>3N4PA
MGHHHHHHDYDIPTTENLYFQGGGTNKISQNTVLITDQSREEFDILRYSTLNTNAYDYFGKTLYV
YLDPAFTTNRKASGTGVAAVGAYRHQFLIYGLEHFFLRDLSESSEVAIAECAAHMIISVLSLHPY
LDELRIAVEGNTNQAAAVRIACLIRQSVQSSTLIRVLFYHTPDQNHIEQPFYLMGRDKALAVEQF
ISRFNSGYIKASQELVSYTIKLSHDPIEYLLEQIQNLHRVTLAEGTTARYSAKRQNRISDDLIIA
VIMATYLCDDIHAIRFRVS
>3NCTD
MKTELTLNVLQTMNAQEYEDIRAAGSDERRELTHAVMRELDAPDNWTMNGEYGSEFGGFFPVQVR
FTPAHERFHLALCSPGDVSQVWVLVLVNAGGEPFAVVQVQRRFASEAVSHSLALAASLDTQGYSV
NDIIHILMAEGGQV
>3NFHB
MTDSAIDIVDSVRTASKDLPTRAQLDEITSNDRPTPLANIDATDVEQIYPIESIIPKKELQFIRV
SSILKEADKEKKLELFPYQNNSKYVAKKLDSLTQPSQMTKLQLLYYLSLLLGVYENRRVNNKTKL
LERLNSPPEILVDGILSRFTVIKPGQFGRSKDRSYFIDPQNEDKILCYILAIIMHLDNFIVEITP
LAHELNLKPSKVVSLFRVLGAIVKGATVAQAEAFGIPKSTAASYKIATMKV
>3NO7A
GPEASARSEVKMTVTVGEERRARLRTAYTLTHLQEGHRTFSGFIAAALDAEVQRLEQRYNEGRRF
ENAERGVTRGRPLGS
>3NQUA
MGPRRRSRKPEAPRRRSPSPTPTPGPSRRGPSLGASSHQHSRRRQGWLKEIRKLQKSTHLLIRKL
PFSRLAREICVKFTRGVDFNWQAQALLALQEAAEAFLVHLFEDAYLLTLHAGRVTLFPKDVQLAR
RIRGLEEGLG
>3NR7B
GSHMSEALKILNNIRTLRAQARESTLETLEEMLEKLEVVVNERREEESAAAAEVEERTRKLQQYR
EMLIADGIDPNELLNSMAAAK
>3NXCA
MPPGKCLFSGVFCNMAEKQTAKRNRREEILQSLALMLESSDGSQRITTAKLAASVGVSEAALYRH
FPSKTRMFDSLIEFIEDSLITRINLILKDEKDTTARLRLIVLLLLGFGERNPGLTRILTGHALMF
EQDRLQGRINQLFERIEAQLRQVLREKRMREGEGYATDETLLASQILAFCEGMLSRFVRSEFKYR
PTDDFDARWPLIAAQLQ
>3O27B
MRPGIRKLVVLNPRAYKGGSGHTTFYLLIPKDIAEALDIKPDDTFILNMEQKDGDIVLSYKRVKE
LKI
>3OA6A
MKKHHHHHHMSASEGMKFKFHSGEKVLCFEPDPTKARVLYDAKIVDVIVGKDEKGRKIPEYLIHF
NGWNRSWDRWAAEDHVLRDTDENRRLQRKLARKAVARLRSTGRKK
>3OD8H
MGSSHHHHHHSSGLVPRGSHMAESSDKLYRVEYAKSGRASCKKCSESIPKDSLRMAIMVQSPMFD
GKVPHWYHFSCFWKVGHSIRHPDVEVDGFSELRWDDQQKVKKTAEAGGVTG
>3OKGA
MSHHHHHHSMDIEFMTEGPYKLPPGWRWVRLGEVCLPTERRDPTKNPSTYFVYVDISAIDSTVGK

IVSPKEILGQHAPSRARKVIRSGDVIFATTRPYLKNIALVPPDLDGQICSTGFCVIRANREFAEP
EFLFHLCRSDFITNQLTASKMRGTSYPAVTDNDVYNTLIPLPPLEEQRRIVAKVEALMERVREVR
RLRAEAQKDTELLMQTALAEVFPHPGADLPPGWRWVRLGEVCDIIMGQSPPSSTYNFEGNGLPFF
QGKADFGDLHPTPRIWCSAPQKVARPGDVLISVRAPVGSTNVANLACCIGRGLAALRPRDSLERF
WLLYYLHYLEPELSKMGAGSTFNAITKKDLQNVFIPLPPLEEQRRIVAYLDQIQQQVAALKRAQA
ETEAELKRLEQAILDKAFRGDL

>3OLCX

MSRNDKEPFFVKFLKSSDNSKCFFKALESIKEFQSEEYLQIITEEEALKIKENDRSLYICDPFSG
VVFDHLKKLGCRIVGPQVVIFCMHHQRCVPRAEHPVYNMVMSDVTISCTSLEKEKREEVHKYVQM
MGGRVYRDLNVSVTHLIAGEVGSKKYLVAANLKKPILLPSWIKTLWEKSQEKKITRYTDINMEDF
KCPIFLGCIICVTGLCGLDRKEVQQLTVKHGGQYMGQLKMNECTHLIVQEPKGQKYECAKRWNVH
CVTTQWFFDSIEKGFCQDESIYKTEPRPEALEHHHHHH

>3ON0D

MPKIQTYVNNNVYEQITDLVTIRKQEGIEEASLSNVSSMLLELGLRVYMIQQEKREGGFNQMEYN
KLMLENVSRVRAMCTEILKMSVLNQESIASGNFDYAVIKPAIDKFAREQVSIFFPDDEDDQE

>3P7NB

MRGSHHHHHHGMASMTGGQQMGRDLYDDDDKDHPFTMGQDRPIDGSGAPGADDTRVEVQPPAQWV
LDLIEASPIASVVSDPRLADNPLIAINQAFTDLTGYSEEECVGRNCRFLAGSGTEPWLTDKIRQG
VREHKPVLVEILNYKKDGTPFRNAVLVAPIYDDDDELLYFLGSQVEVDDDQPNMGMARRERAAEM
LKTLSPRQLEVTTLVASGLRNKEVAARLGLSEKTVKMHRGLVMEKLNLKTSADLVRIAVEAGI

>3P83F

GPLGSPEFPGRLMKAGIDEAGKGCVIGPLVVAGVACSDEDRLRKLGVKDSKKLSQGRREELAEEI
RKICRTEVLKVSPENLDERMAAKTINEILKECYAEIILRLKPEIAYVDSPDVIPERLSRELEEIT
GLRVVAEHKADEKYPLVAAASIIAKVEREREIERLKEKFGDFGSGYASDPRTREVLKEWIASGRI
PSCVRMRWKTVSNLRQKTLDDF

>3P83C

MIDVIMTGELLKTVTRAIVALVSEARIHFLEKGLHSRAVDPANVAMVIVDIPKDSFEVYNIDEEK
TIGVDMDRIFDISKSISTKDLVELIVEDESTLKVKFGSVEYKVALIDPSAIRKEPRIPELELPAK
IVMDAGEFKKAIAAADKISDQVIFRSDKEGFRIEAKGDVDSIVFHMTETELIEFNGGEARSMFSV
DYLKEFCKVAGSGDLLTIHLGTNYPVRLVFELVGGRAKVEYILAPRIESE

>3P9AA

MAAPKGNRFWEARSSHGRNPKFESPEALWAACCEYFEWVEANPLWEMKAFSYQGEVIQEPIAKMR
AMTITGLTLFIDVTLETWRTYRLREDLSEVVTRAEQVIYDQKFSGAAADLLNANIIARDLGLKEQ
SQVEDVTPDKGDRDKRRSRIKELFNRGTGRDS

>3PGGB

SYKVNYMSETPANKSQGGSNQKGGNIILPLALIDKCIGNRIYVVMKGDKEFSGVLRGFDEYVNMV
LDDVQEYGFKADEEDISGGNKKLKRVMVNRLETILLSGNNVAMLVPGGDPDSFNFS

>3POVA

GAMEATPTPADLFSEDYLVDTLDGLTVDDQQAVLASLSFSKFLKHAKVRDWCAQAKIQPSMPALR
MAYNYFLFSKVGEFIGSEDVCNFFVDRVFGGVRLLDVASVYAACSQMNAHQRHHICCLVERATSS
QSLNPVWDALRDGIISSSKFHWAVKQQNTSKKIFSPWPITNNHFVAGPLAFGLRCEEVVKTLLAT
LLHPDETNCLDYGFMQSPQNGIFGVSLDFAANVKTDTEGRLQFDPNCKVYEIKCRFKYTFAKMEC
DPIYAAYQRLYEAPGKLALKDFFYSISKPAVEYVGLGKLPSESDYLVAYDQEWEACPRKKRKLTP
LHNLIRECILHNSTTESDVYVLTDPQDTRGQISIKARFKANLFVNVRHSYFYQVLLQSSIVEEYI

GLDSGIPRLGSPKYYIATGFFRKRGYQDPVNCTIGGDALDPHVEIPTLLIVTPVYFPRGAKHRLL
HQAANFWSRSAKDTFPYIKWDFSYLSANVPHSP
>3PVPB
GPHMISAATIMAATAEYFDTTVEELRGPGKTRALAQSRQIAMYLCRELTDLSLPKIGQAFGRDHT
TVMYAQRKILSEMAERREVFDHVKELTTRIRQRSKR
>3Q8DB
MEGWQRAFVLHSRPWSETSLMLDVFTEESGRVRLVAKGARSKRSTLKGALQPFTPLLLRFGGRGE
VKTLRSAEAVSLALPLSGITLYSGLYINELLSRVLEYETRFSELFFDYLHCIQSLAGVTGTPEPA
LRRFELALLGHLGYGVNFTHCAGSGEPVDDTMTYRYREEKGFIASVVIDNKTFTGRQLKALNARE
FPDADTLRAAKRFTRMALKPYLGGKPLKSRELFRQFMPKRTVKTHYE
>3QMBA
MHHHHHHSSRENLYFQGQIKRSARMCGECEACRRTEDCGHCDFCRDMKKFGGPNKIRQKCRLRQC
QLRARESYKYFPSS
>3QO2A
HMGEDVFEVEKILDMKTEGGKVLYKVRWKGYTSDDDTWEPEIHLEDCKEVLLEFRKKIAENKAK
>3QODB
SNAMSNDVDLIKRLGPSAMDQIMLYLAFSAMRTSGHRHGAFLDAAATAAKCAIYMTYLEQGQNLR
MTGHLHHLEPKRVKAIVEEVRQALTEGKLLKMLGSQEPRYLIQFPYVWMEKYPWRPGRSRIPGTS
LTSEEKRQIEQKLPSNLPDAHLITSFEFLELIEFLHKRSQEDLPKEHQMPLSEALAEHIKRRLLY
SGTVTRIDSPWGMPFYALTRPFYAPADDQERTYIMVEDTARFFRMMRDWAEKRPNTMRVLEELDI
LPEKMQQAKDELDEIIRAWADKYHQDDGVPVVLQMVFGKKED
>3QRFN
SSVPLEWPLSSQSGSYELRIEVQPKPHHRAHYETEGSRGAVKAPTGGHPVVQLHGYMENKPLGLQ
IFIGTADERILKPHAFYQVHRITGKTVTTTSYEKIVGNTKVLEIPLEPKNNMRATIDCAGILKLR
NADIELRKGETDIGRKNTRVRLVFRVHIPESSGRIVSLQTASNPIECSQRSAHELPMVERQDTDS
CLVYGGQQMILTGQNFTSESKVVFTEKTTDGQQIWEMEATVAAAAAAPNMLFVEIPEYRNKHIRT
PVKVNFYVINGKRKRSQPQHFTYHPV
>3QU3A
GSHMAEVRGVQRVLFGDWLLGEVSSGQYEGLQWLNEARTVFRVPWKHFGRRDLDEEDAQIFKAWA
VARGRWPPSGVNLPPPEAEAAERRERRGWKTNFRCALHSTGRFILRQDNSGDPVDPHKVYELSRE
LGSTVGP
>3QVGD
MQEPPDLPVPELPDFFEGKHFFLYGEFPGDERRRLIRYVTAFNGELEDRMNERVQFVITAQEWDP
NFEEALMENPSLAFVRPRWIYSCNEKQKLLPHQLYGVVPQA
>3QVGC
SNSADETLCQTKVLLDIFTGVRLYLPPSTPDFSRLRRYFVAFDGDLVQEFDMTSATHVLGSRDKN
PAAQQVSPEWIWACIRKRRLVAPS
>3R0JB
GTHMRKGVDLVTAGTPGENTTPEARVLVVDDEANIVELLSVSLKFQGFEVYTATNGAQALDRARE
TRPDAVILDVMMPGMDGFGVLRRLRADGIDAPALFLTARDSLQDKIAGLTLGGDDYVTKPFSLEE
VVARLRVILRRAGKGNKEPRNVRLTFADIELDEETHEVWKAGQPVSLSPTEFTLLRYFVINAGTV
LSKPKILDHVWRYDFGGDVNVVESYVSYLRRKIDTGEKRLLHTLRGVGYVLREPR
>3R4KA
GMGTVSKALTLLTYFNHGRLEIGLSDLTRLSGMNKATVYRLMSELQEAGFVEQVEGARSYRLGPQ

VLRLAALREASVPILSASRRVLRELSEDTGETTHLSLLQGEQLASLSHAYSSRNATKVMMEDAEV
LTFHGTASGLAVLAYSEPSFVDAVLAAPLTARTPQTQTDPAAIRAEIAEVRRTGLAQSIGGFEAE
VHSHAVPIFGPDRAVLGALAVAAPTSRMTPDQKRTIPPALRAAGLSLTERIGGACPPEFPTDIAA
>3RCOA
ENLYFQGMLEGDLVSKMLRAVLQSHKNGVALPRLQGEYRSLTGDWIPFKQLGFPTLEAYLRSVPA
VVRIETSRSGEITCYAMACTETAR
>3RH2A
GMKTRDKIIQASLELFNEHGERTITTNHIAAHLDISPGNLYYHFRNKEDIIRCIFDQYEQHLLLG
FKPYADQKVDLELLMSYFDAMFYTMWQFRFMYANLADILARDDTLKARYLKVQQAVLEQSIAVLN
QLKKDGILQIEDERIADLADTIKMIIGFWISYKLTQSSIATISKASLYEGLLRVLMIFKAYSTPD
SLANFDRLEQHFRSQSN
>3RLOA
GSVDDSAQSDLKEVMVLNATESFVYEPKEQKKMFHATVATENEVFRVKVFNIDLKEKFTPKKIIA
IANYVCRNGFLEVYPFTLVADVNADRNMEIPKGLIRSASVTPKINQLCSQTKGSFVNGVFEVHKK
NVRGEFTYYEIQDNTGKMEVVVHGRLTTINCEEGDKLKLTCFELAPKSGNTGELRSVIHSHIKVI
KTRKNAAAS
>3S4WB
SHNSHEVEENGSVFVKLLKASGLTLKTGENQNQLGVDQVIFQRKLFQALRKHPAYPKVIEEFVNG
LESYTEDSESLRNCLLSCERLQDEEASMGTFYSKSLIKLLLGIDILQPAIIKMLFEKVPQFLFES
ENRDGINMARLIINQLKWLDRIVDGKDLTAQMMQLISVAPVNLQHDFITSLPEILGDSQHANVGK
ELGELLVQNTSLTVPILDVFSSLRLDPNFLSKIRQLVMGKLSSVRLEDFPVIVKFLLHSVTDTTS
LEVIAELRENLNVQQFILPSRIQASQSKLKSKGLASSSGNQENSDKDCIVLVFDVIKSAIRYEKT
ISEAWFKAIERIESAAEHKSLDVVMLLIIYSTSTQTKKGVEKLLRNKIQSDCIQEQLLDSAFSTH
YLVLKDICPSILLLAQTLFHSQDQRIILFGSLLYKYAFKFFDTYCQQEVVGALVTHVCSGTEAEV
DTALDVLLELIVLNASAMRLNAAFVKGILDYLENMSPQQIRKIFCILSTLAFSQQPGTSNHIQDD
MHLVIRKQLSSTVFKYKLIGIIGAVTMAGIMAEDRSVPSNSSQRSANVSSEQRTQVTSLLQLVHS
CTEHSPWASSLYYDEFANLIQERKLAPKTLEWVGQTIFNDFQDAFVVDFCAAPEGDFPFPVKALY
GLEEYSTQDGIVINLLPLFYQECAKDASRATSQESSQRSMSSLCLASHFRLLRLCVARQHDGNLD
EIDGLLDCPLFLPDLEPGEKLESMSAKDRSLMCSLTFLTFNWFREVVNAFCQQTSPEMKGKVLSR
LKDLVELQGILEKYLAVIPDYVPPFASVDLDTLDMMPRKTFVSLQNYRAFFRELDIEVFSILHSG
LVTKFILDTEMHTEATEVVQLGPAELLFLLEDLSQKLENMLTAPFAKRICCFKNKGRQNIGFSHL
HQRSVQDIVHCVVQLLTPMCNHLENIHNFFQCLGAEHLSADDKARATAQEQHTMACCYQKLLQVL
HALFAWKGFTHQSKHRLLHSALEVLSNRLKQMEQDQPLEELVSQSFSYLQNFHHSVPSFQCGLYL
LRLLMALLEKSAVPNQKKEKLASLAKQLLCRAWPHGEKEKNPTFNDHLHDVLYIYLEHTDNVLKA
IEEITGVGVPELVSAPKDAASSTFPTLTRHTFVIFFRVMMAELEKTVKGLQAGTAADSQQVHEEK
LLYWNMAVRDFSILLNLMKVFDSYPVLHVCLKYGRRFVEAFLKQCMPLLDFSFRKHREDVLSLLQ
TLQLNTRLLHHLCGHSKIRQDTRLTKHVPLLKKSLELLVCRVKAMLVLNNCREAFWLGTLKNRDL
QGEEIISQDPSSSESNAEDSEDG
>3S4WA
MDLKILSLATDKTTDKLQEFLQTLKDDDLASLLQNQAVKGRAVGTLLRAVLKGSPCSEEDGALRR
YKIYSCCIQLVESGDLQQDVASEIIGLLMLEVHHFPGPLLVDLASDFVGAVREDRLVNGKSLELL
PIILTALATKKEVLACGKGDLNGEEYKRQLIDTLCSVRWPQRYMIQLTSVFKDVCLTPEEMNLVV
AKVLTMFSKLNLQEIPPLVYQLLVLSSKGSRRSVLDGIIAFFRELDKQHREEQSSDELSELITAP
ADELYHVEGTVILHIVFAIKLDCELGRELLKHLKAGQQGDPSKCLCPFSIALLLSLTRIQRFEEQ

VFDLLKTSVVKSFKDLQLLQGSKFLQTLVPQRTCVSTMILEVVRNSVHSWDHVTQGLIEFGFILM
DSYGPKKILDGKAVEIGTSLSKMTNQHACKLGANILLETFKIHEMIRQEILEQVLNRVVTRTSSP
INHFLDLFSDIIMYAPLILQNCSKVTETFDYLTFLPLQTVQGLLKAVQPLLKISMSMRDSLILVL
RKAMFASQLDARKSAVAGFLLLLKNFKVLGSLPSSQCTQSIGVTQVRVDVHSRYSAVANETFCLE
IIDSLKRSLGQQADIRLMLYDGFYDVLRRNSQLASSIMQTLFSQLKQFYEPEPDLLPPLKLGACV
LTQGSQIFLQEPLDHLLSCIQHCLAWYKSRVVPLQQGDEGEEEEEELYSELDDMLESITVRMIKS
ELEDFELDKSADFSQNTNVGIKNNICACLIMGVCEVLMEYNFSISNFSKSKFEEILSLFTCYKKF
SDILSEKAGKGKAKMTSKVSDSLLSLKFVSDLLTALFRDSIQSHEESLSVLRSSGEFMHYAVNVT
LQKIQQLIRTGHVSGPDGQNPDKIFQNLCDITRVLLWRYTSIPTSVEESGKKEKGKSISLLCLEG
LQKTFSVVLQFYQPKVQQFLQALDVMGTEEEEAGVTVTQRASFQIRQFQRSLLNLLSSEEDDFNS
KEALLLIAVLSTLSRLLEPTSPQFVQMLSWTSKICKEYSQEDASFCKSLMNLFFSLHVLYKSPVT
LLRDLSQDIHGQLGDIDQDVEIEKTDHFAVVNLRTAAPTVCLLVLSQAEKVLEEVDWLIAKIKGS
ANQETLSDKVTPEDASSQAVPPTLLIEKAIVMQLGTLVTFFHELVQTALPSGSCVDTLLKGLSKI
YSTLTAFVKYYLQVCQSSRGIPNTVEKLVKLSGSHLTPVCYSFISYVQNKSSDAPKCSEKEKAAV
STTMAKVLRETKPIPNLVFAIEQYEKFLIQLSKKSKVNLMQHMKLSTSRDFKIKGSVLDMVLRED
EEHHHHHH

>3S5RB

GMQATMTDKNTRELLLDAATTLFAEQGIAATTMAEIAASVGVNPAMIHYYFKTRDSLLDTIIEER
IGRIIDMIWEPVTGEEDDPLIMVRDLVNRIVNTCETMLWLPSLWIREIVNEGGALREKMLNNIPI
DKMNKFSAKIAEGQKQGVINSGIDSRLLIGSIIGLTMLPLATAKLRDQIPTMKGLSSEDIVCHVT
ALLFTGLTNPSNSDDIKKQRT

>3SIAA

MQPPVANFCLWNLQPIQGSWMGAACIYQMPPSVRNTWWFPLLNTIPLDQYTRIYQWFMGVDRDRS
GTLEINELMMGQFPGGIRLSPQTALRMMRIFDTDFNGHISFYEFMAMYKFMELAYNLFVMNARAR
SGTLEPHEILPALQQLGFYINQRTSLLLHRLFARGMAFCDLNCWIAICAFAAQTRSAYQMIFMNP
YYGPMKPFNPMEFGKFLDVVTSLLE

>3SQIA

MSKLDSLLKELPTRTAHLYRSIWHKYTEWLKTMPDLTGADLKLFLSQKYIVKYIASHDDIAKDPL
PTCDAMIWFSRALDIENNDVLVLQQRLYGLVKLLEFDYSNVIAILQKISINLWNPSTDSLQSKHF
KTCQDKLKLLLDFQWKFNTNVSFEDRTTVSLKDLQCILDDENGKCGLAHSSKPNFVLVPNFQSPF
TCPIFTMAVYYYLRFHGVKKYYKGDGYQILSQLEHIPIIRGKSLDQYPRELTLGNWYPTIFKYCQ
LPYTKKHWFQVNQEWPQFPDFSDSSENTSTLAESDSENTIGIPDFYIEKMNRTKLQPCPQVHVHL
FPTDLPPDIQAVFDLLNSVLVTSLPLLYRVFPTHDIFLDPSLKTPQNIAFLTGTLPLDIESQEHL
LAQLIDKTGTVSELVPNPVKIDQNEHTLTPIGTSLSQTDIPMLDQLKTELQKLIQLQTSTGFSQL
ITVLLEIFQRLDFKKSNKQFVIDLLQSCRKDMRNKLMDPCSLSTNFADELSDDENEKGNKTGAIY
DPETDNGNEESVSD

>3SSCB

MESIQPWIEKFIKQAQQQRSQSTKDYPTSYRNLRVKLSFGYGNFTSIPWFAFLGEGQEASNGIYP
VILYYKDFDELVLAYGISDTNEPHAQWQFSSDIPKTIAEYFQATSGVYPKKYGQSYYACSQKVSQ
GIDYTRFASMLDNIINDYKLIFNSGKSVIPPLEGHHHHHH

>3TB6B

GIDPFTSAKSALHSNKTIGVLTTYISDYIFPSIIRGIESYLSEQGYSMLLTSTNNNPDNERRGLE
NLLSQHIDGLIVEPTKSALQTPNIGYYLNLEKNGIPFAMINASYAELAAPSFTLDDVKGGMMAAE
HLLSLGHTHMMGIFKADDTQGVKRMNGFIQAHRERELFPSPDMIVTFTTEEKESKLLEKVKATLE

KNSKHMPTAILCYNDEIALKVIDMLREMDLKVPEDMSIVGYDDSHFAQISEVKLTSVKHPKSVLG
KAAAKYVIDCLEHKKPKQEDVIFEPELIIRQSARKLNE
>3TEDA
GPDMDSIGESEVRALYKAILKFGNLKEILDELIADGTLPVKSFEKYGETYDEMMEAAKDCVHEEE
KNRKEILEKLEKHATAYRAKLKSGEIKAENQPKDNPLTRLSLKKREKKAVLFNFKGVKSLNAESL
LSRVEDLKYLKNLINSNYKDDPLKFSLGNNTPKPVQNWSSNWTKEEDEKLLIGVFKYGYGSWTQI
RDDPFLGITDKIFLNEVHNPVAKKSASSSDTTPTPSKKGKGITGSSKKVPGAIHLGRRVDYLLSF
LRGGLNTKSPS
>3TEKB
MGEELREEERGEVRSELITKGEKKLVLIRWNTGKTSAGRLFGRYGPGGRPEFFKLLFGAVAGSLR
EQFGPDGENIFNRIRDSEKFRETSRELFDGLKKWFFEEAVPRYNLERGDIFMISTELVLDPDTGE
LLWNRDKTQLIYWIRSDR
>3THOB
MKILHTSDWHLGVTSWTSSRPVDRREELKKALDKVVEEAEKREVDLILLTGDLLHSRNNPSVVAL
HDLLDYLKRMMRTAPVVVLPGNQDWKGLKLFGNFVTSISSDITFVMSFEPVDVEAKRGQKVRILP
FPYPDESEALRKNEGDFRFFLESRLNKLYEEALKKEDFAIFMGHFTVEGLAGYAGIEQGREIIIN
RALIPSVVDYAALGHIHSFREIQKQPLTIYPGSLIRIDFGEEADEKGAVFVELKRGEPPRYERID
ASPLPLKTLYYKKIDTSALKSIRDSCRNFPGYVRVVYEEDSGILPDLMGEIDNLVKIERKSRREI
EEVLRESPEEFKEELDKLDYFELFKEYLKKREENHEKLLKILDELLDEVKKSEA
>3THOA
HHHHHHSSGENLYFQGHMRPERLTVRNFLGLKNVDIEFQSGITVVEGPNGAGKSSLFEAISFALF
GNGIRYPNSYDYVNRNAVDGTARLVFQFERGGKRYEIIREINALQRKHNAKLSEILENGKKAAIA
AKPTSVKQEVEKILGIEHRTFIRTVFLPQGEIDKLLISPPSEITEIISDVFQSKETLEKLEKLLK
EKMKKLENEISSGGAGGAGGSLEKKLKEMSDEYNNLDLLRKYLFDKSNFSRYFTGRVLEAVLKRT
KAYLDILTNGRFDIDFDDEKGGFIIKDWGIERPARGLSGGERALISISLAMSLAEVASGRLDAFF
IDEGFSSLCTENKEKIASVLKELERLNKVIVFITCDREFSEAFDRKLRITGGVVVNE
>3THWD
CCTCTATCTGAAGCCGATCGATGAAGCATCGATCGCACAGCTTCAGATAGAGG
>3TOCB
GSFTMNLNFSLLDEPIPLRGGTILVLEDVCVFSKIVQYCYQYEEDSELKFFDHKMKTIKESEIML
VTDILGFDVNSSTILKLIHADLESQFNEKPEVKSMIDKLVATITELIVFECLENELDLEYDEITI
LELIKSLGVKVETQSDTIFEKCLEILQIFKYLTKKKLLIFVNSGAFLTKDEVASLQEYISLTNLT
VLFLEPRELYDFPQYILDEDYFLITKNMV
>3TRBB
SNAMAANRMRPIHPGEILAEELGFLDKMSANQLAKHLAIPTNRVTAILNGARSITADTALRLAKF
FGTTPEFWLNLQDAYDIKMALKKSGKKIEKEVTPYDQAA
>3TUOD
GPGSGTMLPVFCVVEHYENAIEYDCKEEHAEFVLVRKDMLFNQLIEMALLSLGYSHSSAAQAKGL
IQVGKWNPVPLSYVTDAPDATVADMLQDVYHVVTLKIQLH
>3U50C
QRIYSSIEEIIQQAQASEIGQKKEFYVYGNLVSIQMKNKLYYYRCTCQGKSVLKYHGDSFFCESC
QQFINPQVHLMLRAFVQDSTGTIPVMIFDQQSSQLINQIDPSIHVQEAGQYVKNCIENGQEEIIR
QLFSKLDFARFIFEIQFENKEFNNEQEIAYKVLKIEKENIKE
>3U58A

GSLSDQLSKQTLLISQLQVGKNRFSFKFEGRVVYKSSTFQNQQDSKYFFITAQDANNQEINLSFW
QKVDQSYQTLKVGQYYYFIGGEVKQFKNNLELKFKFGDYQIIPKETLGGSGGSTLLISEVLKTSK
QYLSVLAQVVDIQSSDKNIRLKICDNSCNQELKVVIFPDLCYEWRDKFSINKWYYFNEFVRQIYN
DEVQLKNNIHSSIKESDD
>3U5ZF
MKLSKDTTALLKNFATINSGIMLKSGQFIMTRAVNGTTYAEANISDVIDFDVAIYDLNGFLGILS
LVNDDAEISQSEDGNIKIADARSTIFWPAADPSTVVAPNKPIPFPVASAVTEIKAEDLQQLLRVS
RGLQIDTIAITVKEGKIVINGFNKVEDSALTRVKYSLTLGDYDGENTFNFIINMANMKMQPGNYK
LLLWAKGKQGAAKFEGEHANYVVALEADSTHDF
>3U5ZA
SLFKDDIQLNEHQVAWYSKDWTAVQSAADSFKEKAENEFFEIIGAINNKTKCSIAQKDYSKFMVE
NALSQFPECMPAVYAMNLIGSGLSDEAHFNYLMAAVPRGKRYGKWAKLVEDSTEVLIIKLLAKRY
QVNTNDAINYKSILTKNGKLPLVLKELKGLVTDDFLKEVTKNVKEQKQLKKLALEWGLEHHHHHH
HHHH
>3U5ZM
GPGGSMITVNEKEHILEQKYRPSTIDECILPAFDKETFKSITSKGKIPHIILHSPSPGTGKTTVA
KALCHDVNADMMFVNGSDCKIDFVRGPLTNFASAASFDGRQKVIVIDEFDRSGLAESQRHLRSFM
EAYSSNCSIIITANNIDGIIKPLQSRCRVITFGQPTDEDKIEMMKQMIRRLTEICKHEGIAIADM
KVVAALVKKNFPDFRKTIGELDSYSSKGVLDAGILSLVTNDRGAIDDVLESLKNKDVKQLRALAP
KYAADYSWFVGKLAEEIYSRVTPQSIIRMYEIVGENNQYHGIAANTELHLAYLFIQLACEMQWK
>3ULJA
GSDPQVLRGSGHCKWFNVRMGFGFISMTSREGSPLENPVDVFVHQSKLYMEGFRSLKEGEPVEFT
FKKSSKGFESLRVTGPGGNPCLGNE
>3ULXA
MGMRRERDAEAELNLPPGFRFHPTDDELVEHYLCRKAAGQRLPVPIIAEVDLYKFDPWDLPERAL
FGAREWYFFTPRDRKYPNGSRPNRAAGNGYWKATGADKPVAPRGRTLGIKKALVFYAGKAPRGVK
TDWIMHEYRLADAGRAAAGAKKGSLRLDDWVLCRLYNKKNEWEK
>3UWXB
MGSSHHHHHHSSGLVPRGSHMGPKKVEGRFQLVSPYEPQGDQPQAIAKLVDGLRLGVKHQTLLGA
TGTGKTFTISNVIAQVNKPTLVIAHNKTLAGQLHSELKEFFPHNAVEYFVSYYDYYQPEAYVPQT
DTYIEKDAKINDEIDKLRHSATSALFERRDVIIVASVSCIYGLGSPEEYRELVVSLRVGMEIERN
ALLRRLVDIQYDRNDIDFRRGTFRVRGDVVEIFPASRDEHCIRVEFFGDEIERIREVDALTGEVL
GEREHVAIFPASHFVTREEKMRLAIQNIEQELEERLAELRAQGKLLEAQRLEQRTRYDLEMMREM
GFCSGIENYSRHLALRPPGSTPYTLLDYFPDDFLIIVDESHVTLPQLRGMYNGDRARKQVLVDHG
FRLPSALDNRPLTFEEFEQKINQIIYISATPGPYELEHSPGVVEQIIRPTGLLDPTIDVRPTKGQ
IDDLIGEIHERVERNERTLVTTLTKKMAEDLTDYLKEAGIKVAYLHSEIKTLERIEIIRDLRLGK
YDVLVGINLLREGLDIPEVSLVAILDADKEGFLRSERSLIQTIGRAARNANGHVIMYADTITKSM
EIAIQETKRRRAIQEEYNRKHGIVPRTVKKEIRDVIRATYAAEETEMYEAKPAAAMTKQEREELI
RKLEAEMKEAAKALDFERAAQLRDIIFELKAEG
>3V20B
MTTNLTNSNCVEEYKENGKTKIRIKPFNALIELYHHQTPTGSIKENLDKLENYVKDVVKAKGLAI
PTSGAFSNTRGTWFEVMIAIQSWNYRVKRELNDYLIIKMPNVKTFDFRKIFDNETREKLHQLEKS
LLTHKQQVRLITSNPDLLIIRQKDLIKSEYNLPINKLTHENIDVALTLFKDIEGKCKWDSLVAGV
GLKTSLRPDRRLQLVHEGNILKSLFAHLKMAYWNPKAEFKYYGASSEPVSKADDDALQTAATHTI

VNVNSTPERAVDDIFSLTSFEDIDKMLDQIIKK

>3V4GA

MHHHHHHSSGVDLGTENLYFQSNAMRPSEKQDNLVRAFKALLKEERFGSQGEIVEALKQEGFENI
NQSKVSRMLTKFGAVRTRNAKMEMVYCLPTELGVPTVSSSLRELVLDVDHNQALVVIHTGPGAAQ
LIARMLDSLGKSEGILGVVAGDDTIFITPTLTITTEQLFKSVCELFEYAG

>3V60A

GSHMRPETHINLKVSDGSSEIFFKIKKTTPLRRLMEAFAKRQGKEMDSLRFLYDGIRIQADQTPE
DLDMEDNDIIEAHREQIGG

>3V62C

SHNPDDTTVDNRPIISNAKFLADAAMKKTQKFSKKVKNEPASSQMDIFSQLSRAKKKSKLNNGEI
IVID

>3V68A

MIERILEFTAKHEEWIVGENVEDFTNENIAMFLSRVSNTVSSKIPGYLGEKIDVNGLLSIKIEGS
LEEKLKALISPKVSRQIGRLVMEDDKKLKKLLVEVAKAVLTREILKNELPIEFPGGKIEGLKIQP
RYEEDHINFTARYGSWIVVKRMIIDEKTPLLDIARLLASINETAVNKIKDFADVDDKKIVEYFGG
FKKVKKEEEIKEIVQLFREFKGNEFEVRYAAREMLSKLGLKVDVPSKNLEKYLEKAG

>3V72A

MSKRKAPQETLNGGITDMLVELANFEKNVSQAIHKYNAYRKAASVIAKYPHKIKSGAEAKKLPGV
GTKIAEKIDEFLATGKLRKLEKIRQDDTSSSINFLTRVTGIGPSAARKLVDEGIKTLEDLRKNED
KLNHHQRIGLKYFEDFEKRIPREEMLQMQDIVLNEVKKLDPEYIATVCGSFRRGAESSGDMDVLL
THPNFTSESSKQPKLLHRVVEQLQKVRFITDTLSKGETKFMGVCQLPSENDENEYPHRRIDIRLI
PKDQYYCGVLYFTGSDIFNKNMRAHALEKGFTINKYTIRPLGVTGVAGEPLPVDSEQDIFDYIQW
RYREPKDRSE

>3V9RD

MLSKEALIKILSQNEGGNDMKIADEVVPMIQKYLDIFIDEAVLRSLQSHKDINGERGDKSPLELS
HQDLERIVGLLLMDMLEHHHHHH

>3V9RC

MNDDEDRAQLKARLWIRVEERLQQVLSSEDIKYTPRFINSLLELAYLQLGEMGSDLQAFARHAGR
GVVNKSDLMLYLRKQPDLQERVTQE

>3VEAA

MKYQQLENLESGWKWAYLVKKHREGEAITRHIENSAAQDAVEQLMKLENEPVKVQEWIDAHMNVN
LATRMKQTIRARRKRHFNAEHQHTRKKSIDLEFLVWQRLAVLARRRGNTLSDTVVQLIEDAERKE
KYASQMSSLKQDLKDILDKEV

>3VH5W

GYRRTVPRGTLRKIIKKHKPHLRLAANTDLLVHLSFLLFLHRLAEEARTNAFENKSKIIKPEHTI
AAAKVILKKSRG

>3VH5A

GSEAAGGEQRELLIQRLRAAVHYTTGALAQDVAEDKGVLFSKQTVAAISEITFRQAENFARDLEM
FARHAKRSTITSEDVKLLARRSNSLLKYITQKSDELASSNMEQKEKKKKKSSAAKGRKTEENETP
VTESEDSNMA

>3VIBA

MRKTKTEALKTKEHLMLAALETFYRKGIARTSLNEIAQAAGVTRDALYWHFKNKEDLFDALFQRI
CDDIENCIAQDAADAEGGSWTVFRHTLLHFFERLQSNDIHYKFHNILFLKCEHTEQNAAVIAIAR
KHQAIWREKITAVLTEAVENQDLADDLDKETAVIFIKSTLDGLIWRWFSSGESFDLGKTAPRIIG

IMMDNLENHPCLRRK
>3VK0C
MMGNKLTLPAELPDEQDLRAVLAYNMRLFRVNKGWSQEELARQCGLDRTYVSAVERKRWNIALSN
IEKMAAALGVAAYQLLLPPQERLKLMTNSADTRQMPSESGILEHHHHHH
>3VPRD
VTTTRDRILEEAAKLFTEKGYEATSVQDLAQALGLSKAALYHHFGSKEEILYEISLLALKGLVAA
GEKALEVADPKEALRRFMEAHARYFEENYPFFVTMLQGIKSLSPENRLKTIALRDRHEENLRAIL
RRGVEQGVFREVDVALAGRAVLSMLNWMIRWFRPDGPMRAEEVARAYHDLILRGLERGSA
>3VW4A
MGHHHHHHRNYHLFEKVRKWAYRAIRQGWPVFSQWLDAVIQRVEMYNASLPVPLSPAECRAIGKS
IAKYTHRKFSPEGFSAVQAARGRKGGTKSKRAAVPTSARSLKPWEALGISRATYYRKLKCDPD
>3VZHA
GHMYRSRDFYVRVSGQRALFTNPATKGGSERSSYSVPTRQALNGIVDAIYYKPTFTNIVTEVKVI
NQIQTELQGVRALLHDYSADLSYVSYLSDVVYLIKFHFVWNEDRKDLNSDRLPAKHEAIMERSIR
KGGRRDVFLGTRECLGLVDDISQEEYETTVSYYNGVNIDLGIMFHSFAYPKDKKTPLKSYFTKTV
MKNGVITFKAQSECDIVNTLSSYAFKAPEEIKSVNDECMEYDAMEKGEN
>3W03B
SGMEELEQGLLMQPWAWLQLAENSLLAKVFITKQGYALLVSDLQQVWHEQVDTSVVSQRAKELNK
RLTAPPAAFLCHLDNLLRPLLKDAAHPSEATFSCDCVADALILRVRSELSGLPFYWNFHCMLASP
SLVSQHLIRPLMGMSLALQCQVRELATLLHMKDLEIQDYQESGATLIRDRLKTEPFEENSFLEQF
MIEKLPEACSIGDGKPFVMNLQDLYMAVTTQEVQVGQKHQ
>3W3WB
PRRRTVGMKSSQGNVPTGNKQSVGKSAKISKPLHIKTSAYQKQYKINLETKARPSAGDEDSAHPD
KNKE
>3W3WA
MSALPEEVNRTLLQIVQAFASPDNQIRSVAEKALSEEWITENNIEYLLTFLAEQAAFSQDTTVAA
LSAVLFRKLALKAPITHIRKEVLAQIRSSLLKGFLSERADSIRHKLSDAIAECVQDDLPAWPELL
QALIESLKSGNPNFRESSFRILTTVPYLITAVDINSILPIFQSGFTDASDNVKIAAVTAFVGYFK
QLPKSEWSKLGILLPSLLNSLPRFLDDGKDDALASVFESLIELVELAPKLFKDMFDQIIQFTDMV
IKNKDLEPPARTTALELLTVFSENAPQMCKSNQNYGQTLVMVTLIMMTEVSIDDDDAAEWIESDD
TDDEEEVTYDHARQALDRVALKLGGEYLAAPLFQYLQQMITSTEWRERFAAMMALSSAAEGCADV
LIGEIPKILDMVIPLINDPHPRVQYGCCNVLGQISTDFSPFIQRTAHDRILPALISKLTSECTSR
VQTHAAAALVNFSEFASKDILEPYLDSLLTNLLVLLQSNKLYVQEQALTTIAFIAEAAKNKFIKY
YDTLMPLLLNVLKVNNKDNSVLKGKCMECATLIGFAVGKEKFHEHSQELISILVALQNSDIDEDD
ALRSYLEQSWSRICRILGDDFVPLLPIVIPPLLITAKATQDVGLIEEEEAANFQQYPDWDVVQVQ
GKHIAIHTSVLDDKVSAMELLQSYATLLRGQFAVYVKEVMEEIALPSLDFYLHDGVRAAGATLIP
ILLSCLLAATGTQNEELVLLWHKASSKLIGGLMSEPMPEITQVYHNSLVNGIKVMGDNCLSEDQL
AAFTKGVSANLTDTYERMQDRHGDGDEYNENIDEEEDFTDEDLLDEINKSIAAVLKTTNGHYLKN
LENIWPMINTFLLDNEPILVIFALVVIGDLIQYGGEQTASMKNAFIPKVTECLISPDARIRQAAS
YIIGVCAQYAPSTYADVCIPTLDTLVQIVDFPGSKLEENRSSTENASAAIAKILYAYNSNIPNVD
TYTANWFKTLPTITDKEAASFNYQFLSQLIENNSPIVCAQSNISAVVDSVIQALNERSLTEREGQ
TVISSVKKLLGFLPSSDAMAIFNRYPADIMEKVHKWFA
>3WE2B
GPLGSKTKDYKTRDVTDDVKSIVRFVQEHSSSQGMRNIKHVGPSGRFTMNMLVDIFLGSKSAKIQ

SGIFGKGSAYSRHNAERLFKKLILDKILDEDLYINANDQAIAYVMLGNKAQTVLNGNLKVDFMET
ENSSSVKKQKALVAKVS
>3ZLJD
PNAAATQVDGTQMSLLSVPEETSPAVEALENLDPRSLTPRQALEWIYRLKSLV
>3ZQJA
MGHHHHHHHHHHSSGHIEGRHMADRLIVKGAREHNLRSVDLDLPRDALIVFTGLSGSGKSSLAFD
TIFAEGQRRYVESLSAYARQFLGQMDKPDVDFIEGLSPAVSIDQKSTNRNPRSTVGTITEVYDYL
RLLYARAGTPHCPTCGERVARQTPQQIVDQVLAMPEGTRFLVLAPVVRTRKGEFADLFDKLNAQG
YSRVRVDGVVHPLTDPPKLKKQEKHDIEVVVDRLTVKAAAKRRLTDSVETALNLADGIVVLEFVD
HELGAPHREQRFSEKLACPNGHALAVDDLEPRSFSFNSPYGACPECSGLGIRKEVDPELVVPDPD
RTLAQGAVAPWSNGHTAEYFTRMMAGLGEALGFDVDTPWRKLPAKARKAILEGADEQVHVRYRNR
YGRTRSYYADFEGVLAFLQRKMSQTESEQMKERYEGFMRDVPCPVCAGTRLKPEILAVTLAGESK
GEHGAKSIAEVCELSIADCADFLNALTLGPREQAIAGQVLKEIRSRLGFLLDVGLEYLSLSRAAA
TLSGGEAQRIRLATQIGSGLVGVLYVLDEPSIGLHQRDNRRLIETLTRLRDLGNTLIVVEHDEDT
IEHADWIVDIGPGAGEHGGRIVHSGPYDELLRNKDSITGAYLSGRESIEIPAIRRSVDPRRQLTV
VGAREHNLRGIDVSFPLGVLTSVTGVSGSGKSTLVNDILAAVLANRLNGARQVPGRHTRVTGLDY
LDKLVRVDQSPIGRTPRSNPATYTGVFDKIRTLFAATTEAKVRGYQPGRFSFNVKGGRCEACTGD
GTIKIEMNFLPDVYVPCEVCQGARYNRETLEVHYKGKTVSEVLDMSIEEAAEFFEPIAGVHRYLR
TLVDVGLGYVRLGQPAPTLSGGEAQRVKLASELQKRSTGRTVYILDEPTTGLHFDDIRKLLNVIN
GLVDKGNTVIVIEHNLDVIKTSDWIIDLGPEGGAGGGTVVAQGTPEDVAAVPASYTGKFLAEVVG
GGASAATSRSNRRRNVSA
>4A0AA
MHHHHHHVDENLYFQGGGRMSYNYVVTAQKPTAVNGCVTGHFTSAEDLNLLIAKNTRLEIYVVTA
EGLRPVKEVGMYGKIAVMELFRPKGESKDLLFILTAKYNACILEYKQSGESIDIITRAHGNVQDR
IGRPSETGIIGIIDPECRMIGLRLYDGLFKVIPLDRDNKELKAFNIRLEELHVIDVKFLYGCQAP
TICFVYQDPQGRHVKTYEVSLREKEFNKGPWKQENVEAEASMVIAVPKPFGGAIIIGQESITYHN
GDKYLAIAPPIIKQSTIVCHNRVDPNGSRYLLGDMEGRLFMLLLEKEEQMDGTVTLKDLRVELLG
ETSIAECLTYLDNGVVFVGSRLGDSQLVKLNVDSNEQGSYVVAMETFTNLGPIVDMCVVDLERQG
QGQLVTCSGAFKEGSLRIIRNGIGIHEHASIDLPGIKGLWPLRSDPNRETDDTLVLSFVGQTRVL
MLNGEEVEETELMGFVDDQQTFFCGNVAHQQLIQITSASVRLVSQEPKALVSEWKEPQAKNISVA
SCNSSQVVVAVGRALYYLQIHPQELRQISHTEMEHEVACLDITPLGDSNGLSPLCAIGLWTDISA
RILKLPSFELLHKEMLGGEIIPRSILMTTFESSHYLLCALGDGALFYFGLNIETGLLSDRKKVTL
GTQPTVLRTFRSLSTTNVFACSDRPTVIYSSNHKLVFSNVNLKEVNYMCPLNSDGYPDSLALANN
STLTIGTIDEIQKLHIRTVPLYESPRKICYQEVSQCFGVLSSRIEVQDTSGGTTALRPSASTQAL
SSSVSSSKLFSSSTAPHETSFGEEVEVHNLLIIDQHTFEVLHAHQFLQNEYALSLVSCKLGKDPN
TYFIVGTAMVYPEEAEPKQGRIVVFQYSDGKLQTVAEKEVKGAVYSMVEFNGKLLASINSTVRLY
EWTTEKELRTECNHYNNIMALYLKTKGDFILVGDLMRSVLLLAYKPMEGNFEEIARDFNPNWMSA
VEILDDDNFLGAENAFNLFVCQKDSAATTDEERQHLQEVGLFHLGEFVNVFCHGSLVMQNLGETS
TPTQGSVLFGTVNGMIGLVTSLSESWYNLLLDMQNRLNKVIKSVGKIEHSFWRSFHTERKTEPAT
GFIDGDLIESFLDISRPKMQEVVANLQYDDGSGMKREATADDLIKVVEELTRIH
>4A11B
MLGFLSARQTGLEDPLRLRRAESTRRVLGLELNKDRDVERIHGGGINTLDIEPVEGRYMLSGGSD
GVIVLYDLENSSRQSYYTCKAVCSIGRDHPDVHRYSVETVQWYPHDTGMFTSSSFDKTLKVWDTN
TLQTADVFNFEETVYSHHMSPVSTKHCLVAVGTRGPKVQLCDLKSGSCSHILQGHRQEILAVSWS

PRYDYILATASADSRVKLWDVRRASGCLITLDQHNGKKSQAVESANTAHNGKVNGLCFTSDGLHL
LTVGTDNRMRLWNSSNGENTLVNYGKVCNNSKKGLKFTVSCGCSSEFVFVPYGSTIAVYTVYSGE
QITMLKGHYKTVDCCVFQSNFQELYSGSRDCNILAWVPSLYEPVPDDDETTTKSQLNPAFEDAWS
SSDEEGGTSAWSHPQFEK
>4ACOA
MRSSILFLLKLMKIMDVQQQQEAMSSEDRFQELVDSLKPRTAHQYKTYYTKYIQWCQLNQIIPTP
EDNSVNSVPYKDLPISAELIHWFLLDTLITDDKPGEKREETEDLDEEEENSFKIATLKKIIGSLN
FLSKLCKVHENPNANIDTKYLESVTKLHTHWIDSQKAITTNETNNNTNTQVLCPPLLKVSLNLWNP
ETNHLSEKFFKTCSEKLRFLVDFQLRSYLNLSFEERSKIRFGSLKLGKRDRDAIIYHKVTHSAEK
KDTPGHHQLLALLPQDCPFICPQTTLAAYLYLRFYGIPSVSKGDGFPNLNADENGSLLQDIPILR
GKSLTTYPREETFSNYYTTVFRYCHLPYKRREYFNKCNLVYPTWDEDTFRTFFNEENHGNWLEQP
EAFAFPDKIPFDFKKIMNFKSPYTSYSTNAKKDPFPPPKDLLVQIFPEIDEYKRHDYEGLSQNSR
DFLDLMEVLRERFLSNLPWIYKFFPNHDIFQDPIFGNSDFQSYFNDKTIHSKGSPILSFDILPGF
NKIYKNKTNFYSLLIERPSQLTFASSHNPDTHPTQKQESEGPLQMSQLDTTQLNELLKQQSFEYV
QFQTLSNFQILLSVFNKIFEKLEMKKSSRGYILHQLNLFKITLDERIKKSKIDDADKFIRDNQPI
KKEENIVNEDGPNTSRRTKRPKQIRLLSIADSSDESSTEDSNVFKKDGESIEDGAYGENEDENDS
EMQEQLKSMINELINSKISTFLRDQMDQFELKINALLDKILEEKVTRIIEQKLGSHTGKFSTLKR
PQLYMTEEHNVGFDMEVPKKLRTSGKYAETVKDNDDHQAMSTTASPSPEQDQEAKSYTDEQEFML
DKSIDSIEGIILEWFTPNAKYANQCVHSMNKSGNKSWRANCEALYKERKSIVEFYIYLVNHESLD
RYKAVDICEKLRDQNEGSFSRLAKFLRKWRHDHQNSFDGLLVYLSN
>4AD8A
GIDPFTMTRKARTPKAAPVPEAVAVVEPPPPDAAPTGPRLSRLEIRNLATITQLELELGGGFCAF
TGETGAGKSIIVDALGLLLGGRANHDLIRSGEKELLVTGFWGDGDESEADSASRRLSSAGRGAAR
LSGEVVSVRELQEWAQGRLTIHWQHSAVSLLSPANQRGLLDRRVTKEAQAYAAAHAAWREAVSRL
ERLQASQRERARQIDLLAFQVQEISEVSPDPGEEEGLNTELSRLSNLHESSKHPTSLVPRGSGSA
ADPEALDRVEARLSALSKLKNKYGPTLEDVVEFGAQAAEELAGLEEDERDAGSLQADVDALHAEL
LKVGQALDAAREREAEPLVDSLLAVIRELGMPHARMEFALSALAEPAAYGLSDVLLRFSANPGEE
LGPLSDVASGGELSRVMLAVSTVLGADTPSVVFDEVDAGIGGAAAIAVAEQLSRLADTRQVLVVT
HLAQIAARAHHHYKVEKQVEDGRTVSHVRLLTGDERLEEIARMLSGNTSEAALEHARELLAG
>4ATKB
ANIKRELTACIFPTESEARALAKERQKKDNHNLIERRRRFNINDRIKELGTLIPKSNDPDMRWNK
GTILKASVDYIRKLQREQQRAKDLENRQKKLEHANRHLLLRVQELEMQARAHG
>4BHXB
GHMTDSEFFHQRFRNLIYVEFVGPRKTLIKLRNLCLDWLQPETRTKEEIIELLVLEQYLTIIPEK
LKPWVRAKKPENCEKLVTLLENYKEMYQP
>4BJ1A
GGGRVDHVFYQKFKSMALQELGTNYLSISYVPSLSKFLSKNLRSMKNCIVFFDKVEHIHQYAGID
RAVSETLSLVDINVVIIEMNDYLMKEGIQSSKSKECIESMGQASYSGQLDFEASEKPSNHTSDLM
MMVMRKINNDESIDHIVYFKFEQLDKLSTSTIIEPSKLTEFINVLSVLEKSNNIAFKVLIYSNNV
SISSLLSTSLKKKLNTKYTVFEMPILTCAQEQEYLKKMIKFTFDSGSKLLQSYNSLVTCQLNNKE
SNLAIFFEFLKVFPHPFTYLFNAYTEIIVQSRTFDELLDKIRNRLTIKNYPHSAYNFKK
>4BJIA
GAMGKGQCRVWIITTNMGVESVPTCRHSKLGEPSKTIQEVIEALKPLFEKRPVWTRRALLNHLDP
SYTHYLKFALPYLSYLWTSGPFRDTYTRFGYDPRKDSNAAAYQALFFKLKLNGKHKGTKTHVFDG

KTLFPTNRVYQVCDIVDPTIAPLLKDTQLRSECHRDTGWYRSGRYYKVRDLMREKLFALIEGEMP
SEVAVNMILNAEEVEESDRY
>4BJXA
GSSHHHHHHSSSMNPPPPETSNPNKPKRQTNQLQYLLRVVLKTLWKHQFAWPFQQPVDAVKLNLP
DYYKIIKTPMDMGTIKKRLENNYYWNAQECIQDFNTMFTNCYIYNKPGDDIVLMAEALEKLFLQK
INELPTEEQELVVTIPNSHKKGA
>4DAPA
MEFSPPLQRATLIQRYKRFLADVITPDGRELTLHCPNTGAMTGCATPGDTVWYSTSDNTKRKYPH
TWELTQSQSGAFICVNTLWANRLTKEAILNESISELSGYSSLKSEVKYGAERSRIDFMLQADSRP
DCYIEVKSVTLAENEQGYFPDAVTERGQKHLRELMSVAAEGQRAVIFFAVLHSAITRFSPARHID
EKYAQLLSEAQQRGVEILAYKAEISAEGMALKKSLPVTL
>4DFCC
GPHMASALVMKKGQRLSRDALRTQLDSAGYRHVDQVMEHGEYATRGALLDLFPMGSELPYRLDFF
DDEIDSLRVFDVDSQRTLEEVEAINLLP
>4DG7H
MGSSHHHHHHSSGLVPRGSHMSNFVNLDIFSNYQKYIDNEQEVRENIRIVVREIEHLSKEAQIKL
QIIHSDLSQISAACGLARKQVELCAQKYQKLAELVPAGQYYRYSDHWTFITQRLIFIIALVIYLE
AGFLVTRETVAEMLGLKISQSEGFHLDVEDYLLGILQLASELSRFATNSVTMGDYERPLNISHFI
GDLNTGFRLLNLKNDGLRKRFDALKYDVKKIEEVVYDVSIRGLSSKEKDQQEEPAVPATE

>4DHXF
MVVSKMNKDAQMRAAINQKLIETGERERLKELLRAKLIECGWKDQLKAHCKEVIKEKGLEHVTVD
DLVAEITPKGRALVPDSVKKELLQRIRTFLAQHASL
>4DHXD
GSLVLSELSQGLAVELMERVMMEFVRETCSQELKNAVETDQRVRVARCCEDVCAHLVDLFLVEEI
FQTAKETLQE
>4DKYB
MNKAELIDVLTQKLGSDRRQATAAVENVVDTIVRAVHKGDSVTITGFGVFEQRRRAARVARNPRT
GETVKVKPTSVPAFRPGAQFKAVVSGAQRLPAEGPHHHHHH
>4DRAE
GSHMEGAGAGSGFRKELVSRLLHLHFKDDKTKVSGDALQLMVELLKVFVVEAAVRGVRQAQAEDA
LRVDVDQLEKVLPQLLLDF
>4DRBI
GSIFSYRDGMRQSSLKKDWFLSEEEFKLWNRLYRLRDSDEIKEITLPQVQFSSLQNEENKPAQES
TTGIHQLSLSEWRLWQDHPLPTHQVDHSDRCRHFIGLMQMIEGMRHEEGECSYELEVESYLQMED
VTSTFIAPRNE
>4DWPA
MGSSHHHHHHSSGLVPRGSHMLAAKRKTKTPVLVERIDQFVGQIKEAMKSDDASRNRKIRDLWDA
EVRYHFDNGRTEKTLELYIMKYRNALKAEFGPKSTPLAICNMKKLRERLNTYIARGDYPKTGVAT
SIVEKIERAEFNTAGRKPTVLLRIADFIAAMNGMDAKQDMQALWDAEIAIMNGRAQTTIISYITK
YRNAIREAFGDDHPMLKIATGDAAMYDEARRVKMEKIANKHGALITFENYRQVLKICEDCLKSSD
PLMIGIGLIGMTGRRPYEVFTQAEFSPAPYGKGVSKWSILFNGQAKTKQGEGTKFGITYEIPVLT
RSETVLAAYKRLRESGQGKLWHGMSIDDFSSETRLLLRDTVFNLFEDVWPKEELPKPYGLRHLYA
EVAYHNFAPPHVTKNSYFAAILGHNNNDLETSLSYMTYTLPEDRDNALARLKRTNERTLQQMATI

APVSRKG
>4E1RB
MSYYHHHHHHDYDIPTTENLYFQGAMAKKVTVTLVDDFDGSGAADETVEFGLDGVTYEIDLSTKN
ATKLRGDLKQWVAAGRRVGGR
>4E2IL
KQVSWKLVTEYAMETKCDDVLLLLGMYLEFQYSFEMCLKCIKKEQPSHYKYHEKHYANAAIFADS
KNQKTICQQAVDTVLAKKRVDSLQLTREQMLTNRFNDLLDRMDIMFGSTGSADIEEWMAGVAWLH
CLLPKMDSVVYDFLKCMVYNIPKKRYWLFKGPIDSGKTTLAAALLELCGGKALNVNLPLDRLNFE
LGVAIDQFLVVFEDVKGTGGESRDLPSGQGINNLDNLRDYLDGSVKVNLEKKHLNKRTQIFPPGI
VTMNEYSVPKTLQARFVKQIDFRPKDYLKHCLERSEFLLEKRIIQSGIALLLMLIWYRPVAEFAQ
SIQSRIVEWKERLDKEFSLSVYQKMKFNVAMGIGVLD
>4EOGA
MGMRVLVTTWGNPFQWEPITYEYRGIKVKSRNTLPILVKTLEPERILILVADTMANYYDSGKNKP
EIEEKSFSSYSEVVEDTKERILWHIKEEVIEELREEDPELAKKIENMLKDERITIEVLPGVGVFG
NITVEGEMLDFYYYATYKLAEWLPVQNNLEVYLDLTHGINFMPTFTYRALRNLLGLLAYLYNVKF
EIVNSEPYPLGVSQEIREDTILHIREIGEGVVRPRPQYSPVEGKLYWNAFISSVANGFPLVFASF
YPNIRDVEDYLNKKLEEFLVGIEVGEREDGKPYVKREKALDRSFKNASKLYYALRVFNTKFQNYP
KKEVPIEEIMEISKIFESLPRIGIILERQVEWLRNLVYGRLWYENGEQKIKKGLLEIIKDKKDKR
KEAEALKKGKTISLAEAAKLTRIFSPSGERIETIESPNVVRNFIAHSGFEYNIVYVKYDRLSDRL
YFFYKDKEKAANLAYEALLYRGEKE
>4EQ6B
MGSSHHHHHHSHGSMEVLKNIRIYPLSNFITSTKNYINLPNELRNLISEEQESKLGFLHIIESDF
KPSVALQKLVNCTTGDEKILIIDIVSIWSQQKQRQHGAIYMNSLSCINITGLIVFLELLYDSPMD
ALRRCQVDNFNFQLRGIVIDNLSFLNFESDKNYDVINLSKFEKLFKILRKLREFLGCWIITKSFP
TDFYNGIENTLVDKWSIKRKSGVTLYPTKLPDSYMKGMDLIIYREVVDGRPQYRRIAALEE
>4EQ6A
MMEYEDLELITIWPSPTKNKLCQFIKQNLSKEHVVTQLFFIDATSSFPLSQFQKLVPPTLPENVR
IYENIRINTCLDLEELSAITVKLLQILSMNKINAQRGTEDAVTEPLKIILYINGLEVMFRNSQFK
SSPQRSHELLRDTLLKLRVMGNDENENASIRTLLEFPKEQLLDYYLKKNNNTRTSSVRSKRRRIK
NGDSLAEYIWKYYADSLFE
>4ER8A
MSEYRRYYIKGGTWFFTVNLRNRRSQLLTTQYQMLRHAIIKVKRDRPFEINAWVVLPEHMHCIWT
LPEGDDDFSSRWREIKKQFTHACGLKNIWQPRFWEHAIRNTKDYRHHVDYIYINPVKHGWVKQVS
DWPFSTFHRDVARGLYPIDWAGDVTDFSAGERIIS
>4EXWE
GIDPFTMLHIEFITDLGAKVTVDVESADKLLDVQRQYGRLGWTSGEVPVGGYQFPLENEPDFDWS
LIGARKWTNPEGEEMILHRGHAYRRRELEAVDSRKMKLPAAVKYSRGAKNTDPEHVREKADGEFE
YVTLAIFRGGKRQERYAVPGSNRPQAGAPARSAATRAQGARPGAVAVQDEETPF
>4F6MA
ANKRMKVKHDDHYELIVDGRVYYICIVCKRSYVCLTSLRRHFNIHSWEKKYPCRYCEKVFPLAEY
RTKHEIHHTGERRYQCLACGKSFINYQFMSSHIKSVHSQDPSGDSKLYRLHPCRSLQIRQYAYLS
DRS
>4FB3E
MHHHHHHSDFPSSLTGYLSHAIYSNKTFPAFLVYSTKEKCKQLYDTIGKFRPEFKCLVHYEEGGM

LFFLTMTKHRVSAVKNYCSKLCSVSFLMCKAVTKPMECYQVVTAAPFQLITENKPGLHQFEFTDE
PEEQKAVDGSHHHHHH

>4FCYC

GTTTTCGCATTTATCGTGAAACGCTTTCGCGTTTTTCGTGCGCCGCTTCATCTGATGTGTTGTTG
ACG

>4FCYB

IARPTLEAHDYDREALWSKWDNASDSQRRLAEKWLPAVQAADEMLNQGISTKTAFATVAGHYQVS
ASTLRDKYYQVQKFAKPDWAAALVDGRGASRRNVHKSEFDEDAWQFLIADYLRPEKPAFRKCYER
LELAAREHGWSIPSRATAFRRIQQLDEAMVVACREGEHALMHLIPAQQRTVEHLDAMQWINGDGY
LHNVFVRWFNGDVIRPKTWFWQDVKTRKILGWRCDVSENIDSIRLSFMDVVTRYGIPEDFHITID
NTRGAANKWLTGGAPNRYRFKVKEDDPKGLFLLMGAKMHWTSVVAGKGWGQAKPVERAFGVGGLE
EYVDKHPALAGAYTGPNPQAKPDNYGDRAVDAELFLKTLAEGVAMFNARTGRETEMCGGKLSFDD
VFEREYARTIVRKPTEEQKRMLLLPAEAVNVSRKGEFTLKVGGSLKGAKNVYYNLALMLAGVKKV
VVRFDPQQLHSTVYCYTLDGRFICEAECLAPVAFNDAAAGREYRRRQKQLKSATKAAIKAQKQMD
ALEVAELLP

>4FE7A

MGSSHHHHHHSSGLVPRGSHMFTKRHRITLLFNANKAYDRQVVEGVGEYLQASQSEWDIFIEEDF
RARIDKIKDWLGDGVIADFDDKQIEQALADVDVPIVGVGGSYHLAESYPPVHYIATDNYALVESA
FLHLKEKGVNRFAFYGLPESSGKRWATEREYAFRQLVAEEKYRGVVYQGLETAPENWQHAQNRLA
DWLQTLPPQTGIIAVTDARARHILQVCEHLHIPVPEKLCVIGIDNEELTRYLSRVALSSVAQGAR
QMGYQAAKLLHRLLDKEEMPLQRILVPPVRVIERRSTDYRSLTDPAVIQAMHYIRNHACKGIKVD
QVLDAVGISRSNLEKRFKEEVGETIHAMIHAEKLEKARSLLISTTLSINEISQMCGYPSLQYFYS
VFKKAYDTTPKEYRDVNSEVML

>4FJOC

MTTLTRQDLNFGQVVADVLSEFLEVAVHLILYVREVYPVGIFQKRKKYNVPVQMSCHPELNQYIQ
DTLHCVKPLLEKNDVEKVVVVILDKEHRPVEKFVFEITQPPLLSINSDSLLSHVEQLLAAFILKI
SVCDAVLDHNPPGCTFTVLVHTREAATRNMEKIQVIKDFPWILADEQDVHMHDPRLIPLKTMTSD
ILKMQLYVEERAHKN

>4FJOA

AAPNLAGAVEFSDVKTLLKEWITTISDPMEEDILQVVRYCTDLIEEKDLEKLDLVIKYMKRLMQQ
SVESVWNMAFDFILDNVQVVLQQTYGSTLKVT

>4FW2B

PLREAKDLHTALHIGPRALSKASNISMQQAREVVQTCPHCNSAPALEAGVNPRGLGPLQIWQTDF
TLEPRMAPRSWLAVTVDTASSAIVVTQHGRVTSVAVQHHWATAIAVLGRPKAIKTDNGSCFTSKS
TREWLARWGIAHTTGIPGNSQGQAMVERANRLLKDKIRVLAEGDGFMKRIPTSKQGELLAKAMYA
LNHKERGENTKTPIQKHWRPTVLTEGPPVKIRIETGEWEKGWNVLVWGRGYAAVKNRDTDKVIWV
PSRKVKPDIT

>4G12B

MTASAPDGRPGQPEATNRRSQLKSDRRFQLLAAAERLFAERGFLAVRLEDIGAAAGVSGPAIYRH
FPNKESLLVELLVGVSARLLAGARDVTTRSANLAAALDGLIEFHLDFALGEADLIRIQDRDLAHL
PAVAERQVRKAQRQYVEVWVGVLRELNPGLAEADARLMAHAVFGLLNSTPHSMKAADSKPARTVR
ARAVLRAMTVAALSAADRCL

>4G4KB

MDNSVETIELKRGSNSVYVQYDDIMFFESSTKSHRLIAHLDNRQIEFYGNLKELSQLDDRFFRCH

NSFVVNRHNIESIDSKERIVYFKNKEHCYASVRNVKKI
>4G6DB
MKLKILDKDNATLNVFHRNKEHKTIDNVPTANLVDWYPLSNAYEYKLSRNGEYLELKRLRSTLPS
SYGLDDNNQDIIRDNNHRCKIGYWYNPAVRKDNLKIIEKAKQYGLPIITEEYDANTVEQGFRDIG
VIFQSLKTIVVTRYLEGKTEEELRIFNMKSEESQLNEALKESDFSVDLTYSDLGQIYNMLLLMKK
ISK
>4G6DA
MKEQLEDVLDTLTDREENVLRLRFGLDDGRTRTLEEVGKVFGVTRERIRQIEAKALRKLRHPSRS
KRLKDFMD
>4GNXL
TTTTTTTTTTTTTTTTTTTTTTTTTTTTTTTTTTTTTTTTTTTTTTTTTTTTTTTTTTTTTT
>4GNXK
TTTTTTTTTTTTTTTTTTTTTTTTTTTTTTTTTTTTTTTTTTTTTTTTTTTTTTTTTTTTTT
>4GNXZ
MPIYPIEGLSPYQNRWTIKARVTSKSDIRHWSNQRGEGKLFSVNLLDDSGEIKATGFNDAVDRFY
PLLQENHVYLISKARVNIAKKQFSNLQNEYEITFENSTEIEECTDATDVPEVKYEFVRINELESV
EANQQCDVIGILDSYGELSEIVSKASQRPVQKRELTLVDQGNRSVKLTLWGKTAETFPTNAGVDE
KPVLAFKGVKVGDFGGRSLSMFSSSTMLINPDITESHVLRGWYDNDGAHAQFQPYTNGGVGGGAM
GGGGAGANMAERRTIVQVKDENLGMSEKPDYFNVRATVVYIKQENLYYTACASEGCNKKVNLDHE
NNWRCEKCDRSYATPEYRYILSTNVADATGQMWLSGFNEDATQLIGMSAGELHKLREESESEFSA
ALHRAANRMYMFNCRAKMDTFNDTARVRYTISRAAPVDFAKAGMELVDAIRAYM
>4GS3A
SNAMAGNFLENNTVTLVGKVFTPLEFSHELYGEKFFNFILEVPRLSETKDYLPITISNRLFEGMN
LEVGTRVKIEGQLRSYNRKSPEEGKNKLILTVFARDISVVPE
>4H79A
GSMTTTETPKTISLTWVGTFVDQRVREIQEGYRLDNPRAVATLARLRRGAGKEIGDTPDLWGLIL
DDRFYADAPPLKEKDMEVAENSAHIALTLYAIHQQSRRDDRMHQRGWGLGEAVRRLMPSSEIDEP
LRKRFVQVGHAVTYKALAQRLREIVTLLRRDAIPLDYGLLADQLYQFRTPQGAQRVRTAWGRGFH
AYRPKTTQNPDSTTTTEKDNS
>4H7AB
MSRGHHHHHHGSMSPGERFLDWLKRLQGQKAWTAARAAFRRSLAFPPGAYPRAMPYVEPFLAKGD
WRQEEREAHYLVAALYALKDGDHQVGRTLARALWEKAQGSASVEKRFLALLEADRDQIAFRLRQA
VALVEGGIDFARLLDDLLRWFSPERHVQARWAREYYGA
>4H9SE
GSRRQIQRLEQLLALYVAEIRRLQEKELDLSELDDPDSAYLQEARLKRKLIRLFGRLCELKDCSS
LTGRVIEQRIPYRGTRYPEVNRRIERLINKPGPDTFPDYGDVLRAVEKAAARHSLGLPRQQLQLM
AQDAFRDVGIRLQERRHLDLIYNFGCHLTDDYRPGVDPALSDPVLARRLRENRSLAMSRLDEVIS
KYAMLQDKSEEGERKKRRARL
>4HD0B
HHHHHHMKFAHLADIHLGYEQFHKPQREEEFAEAFKNALEIAVQENVDFILIAGDLFHSSRPSPG
TLKKAIALLQIPKEHSIPVFAIEGNHDRTQRGPSVLNLLEDFGLVYVIGMRKEKVENEYLTSERL
GNGEYLVKGVYKDLEIHGMKYMSSAWFEANKEILKRLFRPTDNAILMLHQGVREVSEARGEDYFE
IGLGDLPEGYLYYARGHIHKRYETSYSGSPVVYPGSLERWDFGDYEVRYEWDGIKFKERYGVNKG
FYIVEDFKPRFVEIKVRPFIDVKIKGSEEEIRKAIKRLIPLIPKNAYVRLNIGWRKPFDLTEIKE

LLNVEYLKIDTWRI
>4HIDA
MSDSFSLLSQITPHQRCSFYAQVIKTWYSDKNFTLYVTDYTENELFFPMSPYTSSSRWRGPFGRF
SIRCILWDEHDFYCRNYIKEGDYVVMKNVRTKIDHLGYLECILHGDSAKRYNMSIEKVDSEEPEL
NEIKSRKRLYVQN
>4HLXD
MHHHHHHSSGVDLGTENLYFQSMGKASIKDWIVCQVNSGKFPGVEWEDEERTRFRIPVTPLADPC
FEWRRDGELGVVYIRERGNMPVDASFKGTRGRRRMLAALRRTRGLQEIGKGISQDGHHFLVFRVR
KP
>4HOBA
GSHMASNPISEEMNLKILAYLGTKQGAKAVHIAQSLGAQRSEVNRHLYRMSEDGRVRKHPQHPVW
YLPA
>4HP1C
MHHHHHHSSGRENLYFQGSNKKRKRCGVCVPCLRKEPCGACYNCVNRSTSHQICKMRKCEQLKKK
RVVPMKG
>4HTOA
MAASQTSQTVASHVPFADLCSTLERIQKSKGRAEKIRHFREFLDSWRKFHDALHKNHKDVTDSFY
PAMRLILPQLERERMAYGIKETMLAKLYIELLNLPRDGKDALKLLNYRTPTGTHGDAGDFAMIAY
FVLKPRCLQKGSLTIQQVNDLLDSIASNNSAKRKDLIKKSLLQLITQSSALEQKWLIRMIIKDLK
LGVSQQTIFSVFHNDAAELHNVTTDLEKVCRQLHDPSVGLSDISI
>4HW0C
MQLERRKRGTMEIMFDILRNCEPKCGITRVIYGAGINYVVAQKYLDQLVKVGALNIKTENDRKIY
EITEKGKLLRTHIEEFIKIRENLYSAKEKVSELLRTDSE
>4I1KB
MRGSHHHHHHGSRSKFYESASARKRTVTAEERERAINAAKTFEPTNPFFRVVLRPSYLYRGCIMY
LPSGFAEKYLSGISGFIKVQLAEKQWPVRCLYKAGRAKFSQGWYEFTLENNLGEGDVCVFELLRT
RDFVLKVTAFRVNEYV
>4I99D
KKVEIDEEIFVIDDFRVDIEKYVEELYKVVKKIYEKTGTPIKFWDLVPDVEPKIIARTFLYLLFL
ENMGRVEIIQEEPFGEILVVPM
>4I99B
MPYIEKLELKGFKSYGNKKVVIPFSKGFTAIVGANGSGKSNIGDAILFVLGGLSAKAMRASRISD
LIFAGSKNEPPAKYAEVAIYFNNEDRGFPIDEDEVVIRRRVYPDGRSSYWLNGRRATRSEILDIL
TAAMISPDGYNIVLQGDITKFIKMSPLERRLLIDDISGIAEYDSKKEKALEEEKEKKNVFMRTFE
AISRNFSEIFAKLSPGGSARLILENPEDPFSGGLEIEAKPAGKDVKRIEAMSGGEKALTALAFVF
AIQKFKPAPFYLFDEIDAHLDDANVKRVADLIKESSKESQFIVITLRDVMMANADKIIGVSMRDG
VSKVVSLSLEKAMKILEEIRKKQGWEHGN
>4ICGD
GSHMSDKPLTKTDYLMRLRRCQTIDTLERVIEKNKYELSDNELAVFYSAADHRLAELTMNKLYDK
IPSSVWKFIR
>4IDUB
MSSQFIFEDVPQRNAATFNPEVGYVAFIGKYGQQLNFGVARVFFLNQKKAKMVLHKTAQPSVDLT
FGGVKFTVVNNHFPQYVSNPVPDNAITLHRMSGYLARWIADTCKASVLKLAEASAQIVMPLAEVK
GCTWADGYTMYLGFAPGAEMFLDAFDFYPLVIEMHRVLKDNMDVNFMKKVLRQRYGTMTAEEWMT

QKITEIKAAFNSVGQLAWAKSGFSPAARTFLQQFGINI
>4IJHA
GSHMVGQLSRGAIAAIMQKGDTNIKPILQVINIRPITTGNSPPRYRLLMSDGLNTLSSFMLATQL
NPLVEEEQLSSNCVCQIHRFIVNTLKDGRRVVILMELEVLKSAEAVGVKIGNPVPYNE
>4IRHA
GAMVPKTEDQRPQLDPYQILGPTSSRLANPGSGQIQLWQFLLELLSDSSNSSCITWEGTNGEFKM
TDPDEVARRWGERKSKPNMNYDKLSRALRYYYDKNIMTKVHGKRYAYKFDFHGIAQALQPHPPE
>4IX7B
DNVMVSIGPNNTCVPASVFENINWSVCSLATRKLLVTIFDRETLATHSVTGKPSPAFKDQDKPLK
RMLDPGKIQDIIFAVTHKCNASEKEVRNAITTKCADENKMMKIQNVKRRS
>4JJNJ
ATCGGATGTATATATCTGACACGTGCCTGGAGACTAGGGAGTAATCCCCTTGGCGGTTAAAACGC
GGGGGACAGCGCGTACGTGCGTTTAAGCGGTGCTAGAGCTGTCTACGACCAATTGAGCGGCCTCG
GCACCGGGATTCTCGAT
>4JJNI
ATCGAGAATCCCGGTGCCGAGGCCGCTCAATTGGTCGTAGACAGCTCTAGCACCGCTTAAACGCA
CGTACGCGCTGTCCCCCGCGTTTTAACCGCCAAGGGGATTACTCCCTAGTCTCCAGGCACGTGTC
AGATATATACATCCGAT
>4JJNL
SAKTLKDLDGWQVIITDDQGRVIDDNNRRRSRKRGGENVFLKRISDGLSFGKGESVIFNDNVTET
YSVYLIHEIRLNTLNNVVEIWVFSYLRWFELKPKLYYEQFRPDLIKEDHPLEFYKDKFFNEVNKS
ELYLTAELSEIWLKDFIAVGQILPESQWNDSSIDKIEDRDFLVRYACEPTAEKFVPIDIFQIIRR
VKEMEPKQSNEYLKRVSVPVSGQKTNRQVMHKMGVERSSKRLAKKPSMKKIKIEPSADDDVNNGN
IPSQRGTSTTHGSISPQEESVSPNISSASPSALTSPTDSSKILQKRSISKELIVSEEIPINSSEQ
ESDYEPNNETSVLSSKPGSKPEKTSTELVDGRENFVYANNPEVSDDGGLEEETDEVS
>4JJNH
SSAAEKKPASKAPAEKKPAAKKTSTSVDGKKRSKVRKETYSSYIYKVLKQTHPDTGISQKSMSIL
NSFVNDIFERIATEASKLAAYNKKSTISAREIQTAVRLILPGELAKHAVSEGTRAVTKYSSSTQA
>4JJNG
SGGKGGKAGSAAKASQSRSAKAGLTFPVGRVHRLLRRGNYAQRIGSGAPVYLTAVLEYLAAEILE
LAGNAARDNKKTRIIPRHLQLAIRNDDELNKLLGNVTIAQGGVLPNIHQNLLPKKSAKTAKASQE
L
>4JLXA
GAMGAWKLQTVLEKVRLSRHEISEAAEVVNWVVEHLLRRLQGGESEFKGVALLRTGSYYERVKIS
APNEFDVMFKLEVPRIQLEEYCNSGAHYFVKFKRNPGGNPLEQFLEKEILSASKMLSKFRKIIKE
EIKNIEDTGVTVERKRRGSPAVTLLISKPKEISVDIILALESKSSWPASTQKGLPISQWLGAKVK
NNLKRQPFYLVPKHAKEGSGFQEETWRLSFSHIEKDILKNHGQSKTCCEIDGVKCCRKECLKLMK
YLLEQLKKKFGNRRELAKFCSYHVKTAFFHVCTQDPHDNQWHLKNLECCFDNCVAYFLQCLKTEQ
LANYFIPGVNLFSRDLIDKPSKEFLSKQIEYERNNGFPVFW
>4JOIC
MLPKPGTYYLPWEVSAGQVPDGSTLRTFGRLCLYDMIQSRVTLMAQHGSDQHQVLVCTKLVEPFH
AQVGSLYIVLGELQHQQDRGSVVKARVLTCVEGMNLPLLEQAIREQRLYKQERGGSQ
>4JOIB
LDPVFLAFAKLYIRDILDMKESRQVPGVFLYNGHPIKQVDVLGTVIGVRERDAFYSYGVDDSTGV

INCICWKKLNTESVSAAPSAARELSLTSQLKKLQETIEQKTKIEIGDTIRVRGSIRTYREEREIH
ATTYYKVDDPVWNIQIARMLELPTIYRKVYDQPFHS
>4JOLD
SEEMIDHRLTDREWAEEWKHLDHLLNCIMDMVEKTRRSLTVLRRCQEADREELNYWIRRYSDAE
>4JQFA
AEALSNPGALDLPSLTSLLSEKAKEFLMENRVQSFYQQELEMVESLLSLANQPVIHSASSDQVNF
KKDTTSKAIHSIFKNAIQLLQEKGLVFQKDDGFDNLYYVTREDKDLHRKIHRIIQQDCQKPNHME
KGCHFLHILACARLSIRPGLSEAVLQQVLELLEDQSDIVSTMEHYYTAF
>4JW3D
MRGSHHHHHHTDPEKVEMYIKNLQDDSYFVRRAAAAALGKIGDERAVEPLIKALKDEDRFVRSSA
AYALGEIGDERAVEPLIKALKDEDWFVRRAAAVALGEIGGERVRAAMEKLAETGTGFARKVAVNY
LETHKSLIS
>4JW3B
AAPTATVTPSSGLSDGTVVKVAGAGLQAGTAYWVAQWARVDTGVWAYNPADNSSVTADANGSAST
SLTVRRSFEGFLFDGTRWGTVDCTTAACQVGLSDAAGNGPEGVAISFAAHHHHHH
>4K2JA
SHPRYQQPPVPYRQIDDCPAKARPQHIFYRRFLGKDGRRDPKCQWKFAVIFWGNDPYGLKKLSQA
FQFGGVKAGPVSCLPHPGPDQSPITYCVYVYCQNKDTSKKVQMARLAWEASHPLAGNLQSSIVKF
KKPLPLTQPG
>4K74B
HHHHHHMKFTVEREHLLKPLQQVSGPLGGRPTLPILGNLLLQVADGTLSLTGTDLEMEMVARVAL
VQPHEPGATTVPARKFFDICRGLPEGAEIAVQLEGERMLVRSGRSRFSLSTLPAADFPNLDDWQS
EVEFTLPQATMKRLIEATQFSMAHQDVRYYLNGMLFETEGEELRTVATDGHRLAVCSMPIGQSLP
SHSVIVPRKGVIELMRMLDGGDNPLRVQIGSNNIRAHVGDFIFTSKLVDGRFPDYRRVLPKNPDK
HLEAGCDLLKQAFARAAILSNEKFRGVRLYVSENQLKITANNPEQEEAEEILDVTYSGAEMEIGF
NVSYVLDVLNALKCENVRMMLTDSVSSVQIEDAASQSAAYVVMPMRL
>4KPYA
MNHLGKTEVFLNRFALRPLNPEELRPWRLEVVLDPPPGREEVYPLLAQVARRAGGVTVRMGDGLA
SWSPPEVLVLEGTLARMGQTYAYRLYPKGRRPLDPKDPGERSVLSALARRLLQERLRRLEGVWVE
GLAVYRREHARGPGWRVLGGAVLDLWVSDSGAFLLEVDPAYRILCEMSLEAWLAQGHPLPKRVRN
AYDRRTWELLRLGEEDPKELPLPGGLSLLDYHASKGRLQGREGGRVAWVADPKDPRKPIPHLTGL
LVPVLTLEDLHEEEGSLALSLPWEERRRRTREIASWIGRRLGLGTPEAVRAQAYRLSIPKLMGRR
AVSKPADALRVGFYRAQETALALLRLDGAQGWPEFLRRALLRAFGASGASLRLHTLHAHPSQGLA
FREALRKAKEEGVQAVLVLTPPMAWEDRNRLKALLLREGLPSQILNVPLREEERHRWENALLGLL
AKAGLQVVALSGAYPAELAVGFDAGGRESFRFGGAACAVGGDGGHLLWTLPEAQAGERIPQEVVW
DLLEETLWAFRRKAGRLPSRVLLLRDGRVPQDEFALALEALAREGIAYDLVSVRKSGGGRVYPVQ
GRLADGLYVPLEDKTFLLLTVHRDFRGTPRPLKLVHEAGDTPLEALAHQIFHLTRLYPASGFAFP
RLPAPLHLADRLVKEVGRLGIRHLKEVDREKLFFV
>4LDUA
MMASLSCVEDKMKTSCLVNGGGTITTTTSQSTLLEEMKLLKDQSGTRKPVINSELWHACAGPLVC
LPQVGSLVYYFSQGHSEQVAVSTRRSATTQVPNYPNLPSQLMCQVHNVTLHADKDSDEIYAQMSL
QPVHSERDVFPVPDFGMLRGSKHPTEFFCKTLTASDTSTHGGFSVPRRAAEKLFPPLDYSAQPPT
QELVVRDLHENTWTFRHIYRGQPKRHLLTTGWSLFVGSKRLRAGDSVLFIRDEKSQLMVGVRRAN
RQQTALPSSVLSADSMHIGVLAAAAHATANRTPFLIFYNPRACPAEFVIPLAKYRKAICGSQLSV

GMRFGMMFETEDSGKRRYMGTIVGISDLDPLRWPGSKWRNLQVEWDEPGCNDKPTRVSPWDIETP
NSYSQSM
>4LG8A
MHHHHHHSSGRENLYFQGTPEIIQKLQDKATVLTTERKERGKTVPEELVKPEELSKYRQVASHVG
LHSASIPGILALDLCPSDTNKILTGGADKNVVVFDKSSEQILATLKGHTKKVTSVVFHPSQDLVF
SASPDATIRIWSVPNASCVQVVRAHESAVTGLSLHATGDYLLSSSDDQYWAFSDIQTGRVLTKVT
DETSGCSLTCAQFHPDGLIFGTGTMDSQIKIWDLKERTNVANFPGHSGPITSIAFSENGYYLATA
ADDSSVKLWDLRKLKNFKTLQLDNNFEVKSLIFDQSGTYLALGGTDVQIYICKQWTEILHFTEHS
GLTTGVAFGHHAKFIASTGMDRSLKFYSL
>4LJKA
MKSHFQYSTLENIPKAFDILKDPPKKLYCVGDTKLLDTPLKVAIIGTRRPTPYSKQHTITLAREL
AKNGAVIVSGGALGVDIIAQENALPKTIMLSPCSLDFIYPTNNHKVIQEIAQNGLILSEYEKDFM
PIKGSFLARNRLVIALSDVVIIPQADLKSGSMSSARLAQKYQKPLFVLPQRLNESDGTNELLEKG
QAQGIFNIQNFINTLLKDYHLKEMPELEHHHHHH
>4LRVL
SMLPNRMALSRQTEDQLKKLKGYTGITPNIAARLAFFRSVESEFRYSPERDSKKLDGTLVLDKIT
WLGETLQATELVLKMLYPQLEQKALIKAWAAHVEDGIAALRNHK
>4M6WB
MGHIVANEKWRGSQLAQEMQGKIKLIFEDGLTPDFYLSNRCCILYVTEADLVAGNGYRKRLVRVR
NSNNLKGIVVVEKTRMSEQYFPALQKFTVLDLGMVLLPVASQMEASCLVIQLVQEQTKEPSKNPL
LGKKRALLLSEPSLLRTVQQIPGVGKVKAPLLLQKFPSIQQLSNASIGELEQVVGQAVAQQIHAF
FTQPRLEHHHHHH
>4M6WA
MGQEGKGTCILVGGHEITSGLEVISSLRAIHGLQVEVCPLNGCDYIVSNRMVVERRSQSEMLNSV
NKNKFIEQIQHLQSMFERICVIVEKDREKTGDTSRMFRRTKSYDSLLTTLIGAGIRILFSSCQEE
TADLLKELSLVEQRKNVGIHVPTVVNSNKSEALQFYLSIPNISYITALNMCHQFSSVKRMANSSL
QEISMYAQVTHQKAEEIYRYIHYVFD
>4MZ9D
MASRGVNKVILVGNLGQDPEVRYMPNGGAVANITLATSESWRDKATGEMKEQTEWHRVVLFGKLA
EVASEYLRKGSQVYIEGQLRTRKWTDQSGQDRYTTEVVVNVGGTMQMLGGRQGGGAPAGGNIGGG
QPQGGWGQPQQPQGGNQFSGGAQSRPQQSAPAAPSNEPPMDFDDDIPF
>4N0UE
GGPSVFLFPPKPKDTLYITREPEVTCVVVDVSHEDPEVKFNWYVDGVEVHNAKTKPREEQYNSTY
RVVSVLTVLHQDWLNGKEYKCKVSNKALPAPIEKTISKAKGQPREPQVYTLPPSRDELTKNQVSL
TCLVKGFYPSDIAVEWESNGQPENNYKTTPPVLDSDGSFFLYSKLTVDKSRWQQGNVFSCSVMHE
ALHNHYTQKSLSLS
>4N0UD
HKSEVAHRFKDLGEENFKALVLIAFAQYLQQCPFEDHVKLVNEVTEFAKTCVADESAENCDKSLH
TLFGDKLCTVATLRETYGEMADCCAKQEPERNECFLQHKDDNPNLPRLVRPEVDVMCTAFHDNEE
TFLKKYLYEIARRHPYFYAPELLFFAKRYKAAFTECCQAADKAACLLPKLDELRDEGKASSAKQR
LKCASLQKFGERAFKAWAVARLSQRFPKAEFAEVSKLVTDLTKVHTECCHGDLLECADDRADLAK
YICENQDSISSKLKECCEKPLLEKSHCIAEVENDEMPADLPSLAADFVESKDVCKNYAEAKDVFL
GMFLYEYARRHPDYSVVLLLRLAKTYETTLEKCCAAADPHECYAKVFDEFKPLVEEPQNLIKQNC
ELFEQLGEYKFQNALLVRYTKKVPQVSTPTLVEVSRNLGKVGSKCCKHPEAKRMPCAEDYLSVVL

NQLCVLHEKTPVSDRVTKCCTESLVNRRPCFSALEVDETYVPKEFNAETFTFHADICTLSEKERQ
IKKQTALVELVKHKPKATKEQLKAVMDDFAAFVEKCCKADDKETCFAEEGKKLVAASQAALGL
>4N0UB
IQRTPKIQVYSRHPAENGKSNFLNCYVSGFHPSDIEVDLLKNGERIEKVEHSDLSFSKDWSFYLL
YYTEFTPTEKDEYACRVNHVTLSQPKIVKWDRDM
>4N0UA
HLSLLYHLTAVSSPAPGTPAFWVSGWLGPQQYLSYNSLRGEAEPCGAWVWENQVSWYWEKETTDL
RIKEKLFLEAFKALGGKGPYTLQGLLGCELGPDNTSVPTAKFALNGEEFMNFDLKQGTWGGDWPE
ALAISQRWQQQDKAANKELTFLLFSCPHRLREHLERGRGNLEWKEPPSMRLKARPSSPGFSVLTC
SAFSFYPPELQLRFLRNGLAAGTGQGDFGPNSDGSFHASSSLTVKSGDEHHYCCIVQHAGLAQPL
RVEL
>4N6QA
MSAANYPDPSLPRPSTSDDFELIVRQNPNRARVAGGKEKERKPVDPPPIVQIRVREEGTYLAQHY
LQSPYFFMSCSLYDAQEDAPASIPPSTALTGTLVSSLHRLKDVDNTDGGFFVWGDLSIKVEGDFR
LKFSLFEMRKTDVVFLKSIVSERFTVSPPKSFPGMAESTFLSRSFADQGVKLRIRKEPRTSAWSH
PQFEK
>4N6RB
MYAVEDRAHSGHHPPPLSMDRIPPPSTMYPSSAGPSAMVSPAGQPEPESLSTVHDGRIWSLQVVQ
QPIRARMCGFGDKDRRPITPPPCIRLIVKDAQTQKEVDINSLDSSFYVVMADLWNADGTHEVNLV
KHSATSPSISTAMSSSYPPPPHPTSSDYPASYQTNPYGQPVGQPVGQPVGYAGVGNYYGGSTQLQ
YQNAYPNPQAQYYQPMYGGMAQPQMPAAQPVTPGPGGMFTRNLIGCLSASAYRLYDTEDKIGVWF
VLQDLSVRTEGIFRLKFSFVNVGKSVSDLPQSDIAEVINKGTAPILASTFSEPFQVFSAKKFPGV
IESTPLSKVFANQGIKIPIRKDGVKGQGSRGRHSDEDDGLDNEYSAHHHHHH
>4NDFA
GSHMGHWSQGLKISMQDPKMQVYKDEQVVVIKDKYPKARYHWLVLPWTSISSLKAVAREHLELLK
HMHTVGEKVIVDFAGSSKLRFRLGYHAIPSMSHVHLHVISQDFDSPCLKNKKHWNSFNTEYFLES
QAVIEMVQEAGRVTVRDGMPELLKLPLRCHECQQLLPSIPQLKEHLRKHWTQ
>4NJXD
MSHHHHHHSMAAAVVLAAGLRAARRAVAATGVRGGQVRGAAGVTDGNEVAKAQQATPGGAAPTIF
SRILDKSLPADILYEDQQCLVFRDVAPQAPVHFLVIPKKPIPRISQAEEEDQQLLGHLLLVAKQT
AKAEGLGDGYRLVINDGKLGAQSVYHLHIHVLGGRQLQWPPG
>4NL4H
HHHHHHSSGLVPRGSHMSVAHVALPVPLPRTFDYLLPEGMAVKAGCRVRVPFGKQERIGIVAAVS
ERSELPLDELKPVAEALDDEPVFSTTVWRLLMWAAEYYHHPIGDVLFHALPVMLRQGKPASATPL
WYWFATEQGQVVDLNGLKRSRKQQQALAALRQGKIWRHQVGELEFNEAALQALRGKGLAELACEA
PALTDWRSAYSVAGERLRLNTEQATAVGAIHSAADRFSAWLLAGITGSGKTEVYLSVLENVLAQG
RQALVMVPEIGLTPQTIARFRQRFNAPVEVLHSGLNDSERLSAWLKAKNGEAAIVIGTRSSLFTP
FKDLGVIVIDEEHDSSYKQQEGWRYHARDLAVWRAHSEQIPIILGSATPALETLHNVRQGKYRQL
TLSKRAGNARPAQQHVLDLKGQPLQAGLSPALISRMRQHLQADNQVILFLNRRGFAPALLCHDCG
WIAECPRCDSYYTLHQAQHHLRCHHCDSQRPIPRQCPSCGSTHLVPVGIGTEQLEQALAPLFPEV
PISRIDRDTTSRKGALEEHLAAVHRGGARILIGTQMLAKGHHFPDVTLVSLLDVDGALFSADFRS
AERFAQLYTQVSGRAGRAGKQGEVILQTHHPEHPLLQTLLYKGYDAFAEQALAERQTMQLPPWTS
HVLIRAEDHNNQQAPLFLQQLRNLLQASPLADEKLWVLGPVPALAPKRGGRWRWQILLQHPSRVR
LQHIVSGTLALINTLPEARKVKWVLDVDPIEG

>4NQWB
MTEHTDFELLELATPYALNAVSDDERADIDRRVAAAPSPVAAAFNDEVRAVRETMAVVSAATTAE
PPAHLRTAILDATKP

(2) 550 non-DNA-binding proteins

>1RQWA
ATFEIVNRCSYTVWAAASKGDAALDAGGRQLNSGESWTINVEPGTKGGKIWARTDCYFDDSGSGI
CKTGDCGGLLRCKRFGRPPTTLAEFSLNQYGKDYIDISNIKGFNVPMDFSPTTRGCRGVRCAADI
VGQCPAKLKAPGGGCNDACTVFQTSEYCCTTGKCGPTEYSRFFKRLCPDAFSYVLDKPTTVTCPG
SSNYRVTFCPTA
>1H2GB
SNMWVIGKSKAQDAKAIMVNGPQFGWYAPAYTYGIGLHGAGYDVTGNTPFAYPGLVFGHNGVISW
GSTAGLGDDVDIFAERLSAEKPGYYLHNGKWVKMLSREETITVKNGQAETFTVWRTVHGNILQTD
QTTQTAYAKSRAWDGKEVASLLAWTHQMKAKNWQEWTQQAAKQALTINWYYADVNGNIGYVHTGA
YPDRQSGHDPRLPVPGTGKWDWKGLLPFEMNPKVYNPQSGYIANWNNSPQKDYPASDLFAFLWGG
ADRVTEIDRLLEQKPRLTADQAWDVIRQTSRQDLNLRLFLPTLQAATSGLTQSDPRRQLVETLTR
WDGINLLNDDGKTWQQPGSAILNVWLTSMLKRTVVAAVPMPFDKWYSASGYETTQDGPTGSLNIS
VGAKILYEAVQGDKSPIPQAVDLFAGKPQQEVVLAALEDTWETLSKRYGNNVSNWKTPAMALTFR
ANNFFGVPQAAAEETRHQAEYQNRGTENDMIVFSPTTSDRPVLAWDVVAPGQSGFIAPDGTVDKH
YEDQLKMYENFGRKSLWLTKQDVEAHKESQEVLHVQR
>1A12A
RRSPPADAIPKSKKVKVSHRSHSTEPGLVLTLGQGDVGQLGLGENVMERKKPALVSIPEDVVQAE
AGGMHTVCLSKSGQVYSFGCNDEGALGRDTSVEGSEMVPGKVELQEKVVQVSAGDSHTAALTDDG
RVFLWGSFRDNNGVIGLLEPMKKSMVPVQVQLDVPVVKVASGNDHLVMLTADGDLYTLGCGEQGQ
LGRVPELFANRGGRQGLERLLVPKCVMLKSRGSRGHVRFQDAFCGAYFTFAISHEGHVYGFGLSN
YHQLGTPGTESCFIPQNLTSFKNSTKSWVGFSGGQHHTVCMDSEGKAYSLGRAEYGRLGLGEGAE
EKSIPTLISRLPAVSSVACGASVGYAVTKDGRVFAWGMGTNYQLGTGQDEDAWSPVEMMGKQLEN
RVVLSVSSGGQHTVLLVKDKEQS
>3INGA
GMKEIRIILMGTGNVGLNVLRIIDASNRRRSAFSIKVVGVSDSRSYASGRNLDISSIISNKEKTG
RISDRAFSGPEDLMGEAADLLVDCTPASRDGVREYSLYRMAFESGMNVVTANKSGLANKWHDIMD
SANQNSKYIRYEATVAGGVPLFSVLDYSILPSKVKRFRGIVSSTINYVIRNMANGRSLRDVVDDA
IKKGIAESNPQDDLNGLDAARKSVILVNHIFGTEYTLNDVEYSGVDERSYNANDRLVTEVYVDDR
RPVAVSRIISLNKDDFLMSIGMDGLGYQIETDSNGTVNVSDIYDGPYETAGAVVNDILLLSKVQK
>2RAUA
GMYEEWKIVKREAPILGNDQLIENIWKMKREDSPYDIISLHKVNLIGGGNDAVLILPGTWSSGEQ
L23VTISWNGVHYTIPDYRKSIVLYLARNGFNVYTIDYRTHYVPPFLKDRQLSFTANWGWSTWIS
DIKEVVSFIKRDSGQERIYLAGESFGGIAALNYSSLYWKNDIKGLILLDGGPTKHGIRPKFYTPE
VNSIEEMEAKGIYVIPSRGGPNNPIWSYALANPDMPSPDPKYKSISDFLMDSLYVTGSANPYDYP
YSKKEDMFPILASFDPYWPYRLSLERDLKFDYEGILVPTIAFVSERFGIQIFDSKILPSNSEIIL
LKGYGHLDVYTGENSEKDVNSVVLKWLSQQR
>1UAIA
AEPCDYPAQQLDLTDWKVTLPIGSSGKPSEIEQPALDTFATAPWFQVNAKCTGVQFRAAVNGVTT
SGSGYPRSELREMTDGGEEKASWSATSGTHTMVFREAFNHLPEVKPHLVGAQIHDGDDDVTVFRL

EGTSLYITKGDDTHHKLVTSDYKLNTVFEGKFVVSGGKIKVYYNGVLQTTISHTSSGNYFKAGAY
TQANCSNSSPCSSSNYGQVSLYKLQVTHS
>3H5QA
SNAMRMIDIIEKKRDGHTLTTEEINFFIGGYVKGDIPDYQASSLAMAIYFQDMNDDERVALTMAM
VNSGDMIDLSDIKGVKVDKHSTGGVGDTTTLVLAPLVAAVDVPVAKMSGRGLGHTGGTIDKLEAI
DGFHVEIDEATFVKLVNENKVAVVGQSGNLTPADKKLYALRDVTGTVNSIPLIASSIMSKKIAAG
ADAIVLDVKTGSGAFMKTLEDAEALAHAMVRIGNNVGRNTMAIISDMNQPLGRAIGNALELQEAI
DTLKGQGPKDLTELVLTLGSQMVVLANKAETLEEARALLIEAINSGAALEKFKTFIKNQGGDETV
IDHPERLPQAQYQIEYKAKKSGYVTELVSNDIGVASMMLGAGRLTKEDDIDLAVGIVLNKKIGDK
VEEGESLLTIHSNRQDVDDVVKKLDSSITIADHVVSPTLIHKIITE
>3N20A
MSMLKREDWYDLTRTTNWTPKYVTENELFPEEMSGARGISMEAWEKYDEPYKITYPEYVSIQREK
DSGAYSIKAALERDGFVDRADPGWVSTMQLHFGAIALEEYAASTAEARMARFAKAPGNRNMATFG
MMDENRHGQIQLYFPYANVKRSRKWDWAHKAIHTNEWAAIAARSFFDDMMMTRDSVAVSIMLTFA
FETGFVNMQFLGLAADAAEAGDHTFASLISSIQTDESRHAQQGGPSLKILVENGKKDEAQQMVDV
AIWRSWKLFSVLTGPIMDYYTPLESRNQSFKEFMLEWIVAQFERQLLDLGLDKPWYWDQFMQDLD
ETHHGMHLGVWYWRPTVWWDPAAGVSPEEREWLEEKYPGWNDTWGQCWDVITDNLVNGKPELTVP
ETLPTICNMCNLPIAHTPGNKWNVKDYQLEYEGRLYHFGSEADRWCFQIDPERYENHTNLVDRFL
KGEIQPADLAGALMYMSLEPGVMGDDAHDYEWVKAYQKKTNAA
>3N2BA
MHHHHHHSSGVDLGTENLYFQSNAMDYFNYQEDGQLWAEQVPLADLANQYGTPLYVYSRATLERH
WHAFDKSVGDYPHLICYAVKANSNLGVLNTLARLGSGFDIVSVGELERVLAAGGDPSKVVFSGVG
KTEAEMKRALQLKIKCFNVESEPELQRLNKVAGELGVKAPISLRINPDVDAKTHPYISTGLRDNK
FGITFDRAAQVYRLAHSLPNLDVHGIDCHIGSQLTALAPFIDATDRLLALIDSLKAEGIHIRHLD
VGGGLGVVYRDELPPQPSEYAKALLDRLERHRDLELIFEPGRAIAANAGVLVTKVEFLKHTEHKN
FAIIDAAMNDLIRPALYQAWQDIIPLRPRQGEAQTYDLVGPVCETSDFLGKDRDLVLQEGDLLAV
RSSGAYGFTMSSNYNTRPRVAEVMVDGNKTYLVRQREELSSLWALESVLPE
>1P0WA
TIKEMPQPKTFGELKNLPLLNTDKPVQALMKIADELGEIFKFEAPGRVTRYLSSQRLIKEACDES
RFDKNLSQALKFVRDFAGDGLFTSWTHEKNWKKAHNILLPSFSQQAMKGYHAMMVDIAVQLVQKW
ERLNADEHIEVPEDMTRLTLDTIGLCGFNYRFNSFYRDQPHPFITSMVRALDEAMNKLQRANPDD
PAYDENKRQFQEDIKVMNDLVDKIIADRKASGEQSDDLLTHMLNGKDPETGEPLDDENIRYQIIT
FLIAGHETTSGLLSFALYFLVKNPHVLQKAAEEAARVLVDPVPSYKQVKQLKYVGMVLNEALRLW
PTAPAFSLYAKEDTVLGGEYPLEKGDELMVLIPQLHRDKTIWGDDVEEFRPERFENPSAIPQHAF
KPWGNGQRACIGQQFALHEATLVLGMMLKHFDFEDHTNYELDIKETLTLKPEGFVVKAKSKKIPL
>1UJ0A
AGHMARRVRALYDFEAVEDNELTFKHGELITVLDDSDANWWQGENHRGTGLFPSNFVTTDLS
>1UJMA
AKIDNAVLPEGSLVLVTGANGFVASHVVEQLLEHGYKVRGTARSASKLANLQKRWDAKYPGRFET
AVVEDMLKQGAYDEVIKGAAGVAHIASVVSFSNKYDEVVTPAIGGTLNALRAAAATPSVKRFVLT
SSTVSALIPKPNVEGIYLDEKSWNLESIDKAKTLPESDPQKSLWVYAASKTEAELAAWKFMDENK
PHFTLNAVLPNYTIGTIFDPETQSGSTSGWMMSLFNGEVSPALALMPPQYYVSAVDIGLLHLGCL
VLPQIERRRVYGTAGTFDWNTVLATFRKLYPSKTFPADFPDQGQDLSKFDTAPSLEILKSLGRPG
WRSIEESIKDLVGSETA

>1PAHA
TVPWFPRTIQELDRFANQILSYGAELDADHPGFKDPVYRARRKQFADIAYNYRHGQPIPRVEYME
EEKKTWGTVFKTLKSLYKTHACYEYNHIFPLLEKYCGFHEDNIPQLEDVSQFLQTCTGFRLRPVA
GLLSSRDFLGGLAFRVFHCTQYIRHGSKPMYTPEPDICHELLGHVPLFSDRSFAQFSQEIGLASL
GAPDEYIEKLATIYWFTVEFGLCKQGDSIKAYGAGLLSSFGELQYCLSEKPKLLPLELEKTAIQN
YTVTEFQPLYYVAESFNDAKEKVRNFAATIPRPFSVRYDPYTQRIEVL
>1XKWA
ESTSATQPPGVTTLGKVPLKPRELPQSASVIDHERLEQQNLFSLDEAMQQATGVTVQPFQLLTTA
YYVRGFKVDSFELDGVPALLGNTASSPQDMAIYERVEILRGSNGLLHGTGNPAATVNLVRKRPQR
EFAASTTLSAGRWDRYRAEVDVGGPLSASGNVRGRAVAAYEDRDYFYDVADQGTRLLYGVTEFDL
SPDTLLTVGAQYQHIDSITNMAGVPMAKDGSNLGLSRDTYLDVDWDRFKWDTYRAFGSLEQQLGG
GWKGKVSAEYQEADSRLRYAGSFGAIDPQTGDGGQLMGAAYKFKSIQRSLDANLNGPVRLFGLTH
ELLGGVTYAQGETRQDTARFLNLPNTPVNVYRWDPHGVPRPQIGQYTSPGTTTTTQKGLYALGRI
KLAEPLTLVVGGRESWWDQDTPATRFKPGRQFTPYGGLIWDFARDWSWYVSYAEVYQPQADRQTW
NSEPLSPVEGKTYETGIKGELADGRLNLSLAAFRIDLENNPQEDPDHPGPPNNPFYISGGKVRSQ
GFELEGTGYLTPYWSLSAGYTYTSTEYLKDSQNDSGTRYSTFTPRHLLRLWSNYDLPWQDRRWSV
GGGLQAQSDYSVDYRGVSMRQGGYALVNMRLGYKIDEHWTAAVNVNNLFDRTYYQSLSNPNWNNR
YGEPRSFNVSLRGAF
>2EX4A
MGSSHHHHHHSSGLVPRGSTSEVIEDEKQFYSKAKTYWKQIPPTVDGMLGGYGHISSIDINSSRK
FLQRFLREGPNKTGTSCALDCGAGIGRITKRLLLPLFREVDMVDITEDFLVQAKTYLGEEGKRVR
NYFCCGLQDFTPEPDSYDVIWIQWVIGHLTDQHLAEFLRRCKGSLRPNGIIVIKDNMAQEGVILD
DVDSSVCRDLDVVRRIICSAGLSLLAEERQENLPDEIYHVYSFALR
>1ZDQA
ATAAEIAALPRQKVELVDPPFVHAHSQVAEGGPKVVEFTMVIEEKKIVIDDAGTEVHAMAFNGTV
PGPLMVVHQDDYLELTLINPETNTLMHNIDFHAATGALGGGGLTEINPGEKTILRFKATKPGVFV
YHCAPPGMVPWHVVSGGNGAIMVLPREGLHDGKGKALTYDKIYYVGEQDFYVPRDENGKYKKYEA
PGDAYEDTVKVMRTLTPTHVVFNGAVGALTGDKAMTAAVGEKVLIVHSQANRDTRPHLIGGHGDY
VWATGKFNTPPDVDQETWFIPGGAAGAAFYTFQQPGIYAYVNHNLIEAFELGAAAHFKVTGEWND
DLMTSVLAPSG
>3ME7A
MSLGTYVPGDITLVDSYGNEFQLKNLKGKPIILSPIYTHCRAACPLITKSLLKVIPKLGTPGKDF
WVITFTFDPKDTLEDIKRFQKEYGIDGKGWKVVKAKTSEDLFKLLDAIDFRFMTAGNDFIHPNVV
VVLSPELQIKDYIYGVNYNYLEFVNALRLARGEGHHHHHH
>2NT3A
GSHMSKKILIVESDTALSATLRSALEGRGFTVDETTDGKGSVEQIRRDRPDLVVLAVDLSAGQNG
YLICGKLKKDDDLKNVPIVIIGNPDGFAQHRKLKAHADEAVAKPVDADQLVERAGALIGFPE
>1RFXA
SSMPLCPIDEAIDKKIKQDFNSLFPNAIKNIGLNCWTVSSRGKLASCPEGTAVLSCSCGSACGSW
DIREEKVCHCQCARIDWTAARCCKLQVAS
>1C7JA
MTHQIVTTQYGKVKGTTENGVHKWKGIPYAKPPVGQWRFKAPEPPEVWEDVLDATAYGPVCPQPS
DLLSLSYTELPRQSEDCLYVNVFAPDTPSQNLPVMVWIHGGAFYLGAGSEPLYDGSKLAAQGEVI
VVTLNYRLGPFGFMHLSSFDEAYSDNLGLLDQAAALKWVRENISAFGGDPDNVTVFGESAGGMSI

AALLAMPAAKGLFQKAIMESGASRTMTKEQAASTAAAFLQVLGINESQLDRLHTVAAEDLLKAAD
QLRIAEKENIFQLFFQPALDPKTLPEEPEKSIAEGAASGIPLLIGTTRDEGYLFFTSDSDVRSQE
TLDAALEYSLGKPLAEKAADLYPRSLESQIHMVTDLLFWRPAVAFASAQSHYAPVWMYRFDWHPE
KPPYNKAFHALELPFVFGNLDGLERMAKAEITDEVKQLSHTIQSAWITFAKTGNPSTEAVNWPAY
HEETRETVILDSEITIENDPESEKRQKLFPSKGE

>1H0HB

SKGFFVDTTRCTACRGCQVACKQWHGNPATPTENTGFHQNPPDFNFHTYKLVRMHEQEIDGRIDW
LFFPDQCRHCIAPPCKATADMEDESAIIHDDATGCVLFTPKTKDLEDYESVISACPYDVPRKVAE
SNQMAKCDMCIDRITNGLRPACVTSCPTGAMNFGDLSEMEAMASARLAEIKAAYSDAKLCDPDDV
RVIFLTAHNPKLYHEYAVA

>1ZE3D

DLYFNPRFLADDPQAVADLSRFENGQELPPGTYRVDIYLNNGYMATRDVTFNTGDSEQGIVPCLT
RAQLASMGLNTASVAGMNLLADDACVPLTTMVQDATAHLDVGQQRLNLTIPQAFMSNRAR

>1UHAA

APECGERASGKRCPNGKCCSQWGYCGTTDNYCGQGCQSQCDYWRCGRDFGGRLCEEDMCCSKYGW
CGYSDDHCEDGCQSQCD

>1J8MF

SKLLDNLRDTVRKFLTGSSSYDKAVEDFIKELQKSLISADVNVKLVFSLTNKIKERLKNEKPPTY
IERREWFIKIVYDELSNLFGGDKEPKVIPDKIPYVIMLVGVQGTGKTTTAGKLAYFYKKKGFKVG
LVGADVYRPAALEQLQQLGQQIGVPVYGEPGEKDVVGIAKRGVEKFLSEKMEIIIVDTAGRHGYG
EEAALLEEMKNIYEAIKPDEVTLVIDASIGQKAYDLASKFNQASKIGTIIITKMDGTAKGGGALS
AVAATGATIKFIGTGEKIDELEVFNPRRFVARLHHHH

>3K8GA

GSGAWKASVDPLGVVGSGADVYLYFPVAGNENLISRIIENHESKADIKKIVDRTTAVYGAFFARS
KEFRLFGSGSYPYAFTNLIFSRSDGWASTKTEHGITYYESEHTDVSIPAPHFSCVIFGSSKRERM
SKMLSRLVNPDRPQLPPRFEKECTSEGTSQTVALYIKNGGHFITKLLNFPQLNLPLGAMELYLTA
RRNEYLYTLSLQLGNAKINFPIQFLISRVLNAHIHVEGDRLIIEDGTISAERLASVISSLYSKKG
SS

>2B1LA

MQFYQADVLTQGKPVLLNVWATWCPTCRAEHQYLNQLSAQGIRVVGMNYKDDRQKAISWLKELGN
PYALSLFDGDGMLGLDLGVYGAPETFLIDGNGIIRYRHAGDLNPRVWEEEIKPLWEKYSKEAAQ

>2ZZ3A

GSHMRSRRVDVMDVMNRLILAMDLMNRDDALRVTGEVREYIDTVKIGYPLVLSEGMDIIAEFRKR
FGCRIIAAFKVADIPETNEKICRATFKAGADAIIVHGFPGADSVRACLNVAEEMGREVFLLTEMS
HPGAEMFIQGAADEIARMGVDLGVKNYVGPSTRPERLSRLREIIGQDSFLISPGVGAQGGDPGET
LRFADAIIVGRSIYLADNPAAAAAGIIESIKDLRIPEDPAANKARKEAELAAATAEQ

>3GY1A

MSLEPTIITDVLCYITKPDRHNLVVVKVETNKGIYGLGCATFQQRPKAVSLVVSEYLKPILIGRD
ANNIEDLWQMMMVNSYWRNGPILNNAISGVDMALWDIKGKLANMPLYQLFGGKSRDAIAAYTHAV
ADNLEDLYTEIDEIRKKGYQHIRCQLGFYGGNSSEFHTTDNPTQGSYFDQDEYMRTTVSMFSSLR
EKYGYKFHILHDVHERLFPNQAVQFAKDVEKYKPYFIEDILPPDQNEWLGQIRSQTSTPLATGEL
FNNPMEWKSLIANRQVDFIRCHVSQIGGITPALKLGSLCAAFGVRIAWHTPSDITPIGVAVNIHL
NINLHNAAIQENIEINDNTRCVFSGIPEAKNGFFYPIESPGIGVDIDENEIIKYPVEYRPHEWTQ
SRIPDGTIVTEGHHHHHH

>1XMZA
MRGSHHHHHHGSASFLKKTMPFKTTIEGTVNGHYFKCTGKGEGNPFEGTQEMKIEVIEGGPLPFA
FHILSTSCXSKTFIKYVSGIPDYFKQSFPEGFTWERTTTYEDGGFLTAHQDTSLDGDCLVYKVKI
LGNNFPADGPVMQNKAGRWEPGTEIVYEVDGVLRGQSLMALKCPGGRHLTCHLHTTYRSKKPASA
LKMPGFHFEDHRIEIMEEVEKGKCYKQYEAAVGRYCDAAPSKLGHN
>2P35A
QGHMAWSAQQYLKFEDERTRPARDLLAQVPLERVLNGYDLGCGPGNSTELLTDRYGVNVITGIDS
DDDMLEKAADRLPNTNFGKADLATWKPAQKADLLYANAVFQWVPDHLAVLSQLMDQLESGGVLAV
QMPDNLQEPTHIAMHETADGGPWKDAFSGGGLRRKPLPPPSDYFNALSPKSSRVDVWHTVYNHPM
KDADSIVEWVKGTGLRPYLAAAGEENREAFLADYTRRIAAAYPPMADGRLLLRFPRLFVVAVKK
>2P3JA
VLDGPYQPTTFNPPVDYWMLLAPTAAGVVVEGTNNTDAWLATILVEPNVTSETRSYTLFGTQEQI
TIANASQTQWKFIDVVKTTQNGSYSQYGPLQSTPKLYAVMKHNGKIYTYNGETPNVTTKYYSTTN
YDSVNMTAFCDFYIIPREEESTCTEYINNGL
>2OFXA
QGHMATNVTYQAHHVSRNKRGQVVGTRGGFRGCTVWLTGLSGAGKTTVSMALEEYLVCHGIPCYT
LDGDNIRQGLNKNLGFSPEDREENVRRIAEVAKLFADAGLVCITSFISPYTQDRNNARQIHEGAS
LPFFEVFVDAPLHVCEQRDVKGLYKKARAGEIKGFTGIDSEYEKPEAPELVLKTDSCDVNDCVQQ
VVELLQERDIVP
>2QSIA
GHMSGSLARAAARPNAPTLVDEATVDDFIAHSGKIVVLFFRGDAVRFPEAADLAVVLPELINAFP
GRLVAAEVAAEAERGLMARFGVAVCPSLAVVQPERTLGVIAKIQDWSSYLAQIGAMLAEVDQPGE
AELQSGS
>2J9FA
SSLDDKPQFPGASAEFIDKLEFIQPNVISGIPIYRVMDRQGQIINPSEDPHLPKEKVLKLYKSMT
LLNTMDRILYESQRQGRISFYMTNYGEEGTHVGSAAALDNTDLVFGQYREAGVLMYRDYPLELFM
AQCYGNISDLGKGRQMPVHYGCKERHFVTISSPLATQIPQAVGAAYAAKRANANRVVICYFGEGA
ASEGDAHAGFNFAATLECPIIFFCRNNGYAISTPTSEQYRGDGIAARGPGYGIMSIRVDGNDVFA
VYNATKEARRRAVAENQPFLIEAMTYRIGHHSTSDDSSAYRPVDEVNYWDKQDHPISRLRHYLLS
QGWWDEEQEKAWRKQSRRKVMEAFEQAERKPKPNPNLLFSDVYQEMPAQLRKQQESLARHLQTYG
EHYPLDHFDK
>3BB0A
MGSVTPIPLPKIDEPEEYNTNYILFWNHVGLELNRVTHTVGGPLTGPPLSARALGMLHLAIHDAY
FSICPPTDFTTFLSPDTENAAYRLPSPNGANDARQAVAGAALKMLSSLYMKPVEQPNPNPGANIS
DNAYAQLGLVLDRSVLEAPGGVDRESASFMFGEDVADVFFALLNDPRGASQEGYHPTPGRYKFDD
EPTHPVVLIPVDPNNPNGPKMPFRQYHAPFYGKTTKRFATQSEHFLADPPGLRSNADETAEYDDA
VRVAIAMGGAQALNSTKRSPWQTAQGLYWAYDGSNLIGTPPRFYNQIVRRIAVTYKKEEDLANSE
VNNADFARLFALVDVACTDAGIFSWKEKWEFEFWRPLSGVRDDGRPDHGDPFWLTLGAPATNTND
IPFKPPFPAYPSGHATFGGAVFQMVRRYYNGRVGTWKDDEPDNIAIDMMISEELNGVNRDLRQPY
DPTAPIEDQPGIVRTRIVRHFDSAWELMFENAISRIFLGVHWRFDAAAARDILIPTTTKDVYAVD
NNGATVFQNVEDIRYTTRGTREDREGLFPIGGVPLGIEIADEIFNNGLKPTPPEIQPMPQETPVQ
KPVGQQPVKGMWEEEQAPVVKEAP
>3H9CA
TQVAKKILVTCALPYANGSIHLGHMLEHIQADVWVRYQRMRGHEVNFICADDAHGTPIMLKAQQL

GITPEQMIGEMSQEHQTDFAGFNISYDNYHSTHSEENRQLSELIYSRLKENGFIKNRTISQLYDP
EKGMFLPDRFVKGTCPKCKSPDQYGDNCEVCGATYSPTELIEPKSVVSGATPVMRDSEHFFFDLP
SFSEMLQAWTRSGALQEQVANKMQEWFESGLQQWDISRDAPYFGFEIPNAPGKYFYVWLDAPIGY
MGSFKNLCDKRGDSVSFDEYWKKDSTAELYHFIGKDIVYFHSLFWPAMLEGSNFRKPSNLFVHGY
VTVNGAKMSKSRGTFIKASTWLNHFDADSLRYYYTAKLSSRIDDIDLNLEDFVQRVNADIVNKVV
NLASRNAGFINKRFDGVLASELADPQLYKTFTDAAEVIGEAWESREFGKAVREIMALADLANRYV
DEQAPWVVAKQEGRDADLQAICSMGINLFRVLMTYLKPVLPKLTERAEAFLNTELTWDGIQQPLL
GHKVNPFKALYNRIDMRQVEALVEASK
>2FHLA
ARKCSLTGKWTNNLGSIMTIRAVNSRGEFTGTYLTAVADNPGNITLSPLLGIQHKRASQPTFGFT
VHWNFSESTTVFTGQCFIDRNGKEVLKTMWLLRSSVNDISYDWKATRVGYNNFTRLS
>2PRVA
GMIYSKVENFINENKQNAIFTEGASHENIGRIEENLQCDLPNSYKWFLEKYGAGGLFGVLVLGYN
FDHASVVNRTNEYKEHYGLTDGLVVIEDVDYFAYCLDTNKMKDGECPVVEWDRVIGYQDTVADSF
IEFFYNKIQEAKDDWDEDEDWDD
>1JLJA
MATEGMILTNHDHQIRVGVLTVSDSCFRNLAEDRSGINLKDLVQDPSLLGGTISAYKIVPDEIEE
IKETLIDWCDEKELNLILTTGGTGFAPRDVTPEATKEVIEREAPGMALAMLMGSLNVTPLGMLSR
PVCGIRGKTLIINLPGSKKGSQECFQFILPALPHAIDLLRDAIVKVKEVHDRSHHHHHH
>1XFIA
SESDSEMVPFPQLPMPIENNYRACTIPYRFPSDDPKKATPNEISWINVFANSIPSFKKRAESDIT
VPDAPARAEKFAERYAGILEDLKKDPESHGGPPDGILLCRLREQVLRELGFRDIFKKVKDEENAK
AISLFPQVVSLSDAIEDDGKRLENLVRGIFAGNIFDLGSAQLAEVFSRDGMSFLASCQNLVPRPW
VIDDLENFQAKWINKSWKKAVIFVDNSGADIILGILPFARELLRRGAQVVLAANELPSINDITCT
ELTEILSQLKDENGQLLGVDTSKLLIANSGNDLPVIDLSRVSQELAYLSSDADLVIVEGMGRGIE
TNLYAQFKCDSLKIGMVKHLEVAEFLGGRLYDCVFKFNEVQS
>1KZ1A
MFSGIKGPNPSDLKGPELRILIVHARGNLQAIEPLVKGAVETMIEKHDVKLENIDIESVPGSWEL
PQGIRASIARNTYDAVIGIGVLIKGSTMHFEYISEAVVHGLMRVGLDSGVPVILGLLTVLNEEQA
LYRAGLNGGHNHGNDWGSAAVEMGLKALY
>2WL5A
STPSIVIASAARTAVGSFNGAFANTPAHELGATVISAVLERAGVAAGEVNEVILGQVLPAGEGQN
PARQAAMKAGVPQEATAWGMNQLCGSGLRAVALGMQQIATGDASIIVAGGMESMSMAPHCAHLRG
GVKMGDFKMIDTMIKDGLTDAFYGYHMGTTAENVAKQWQLSRDEQDAFAVASQNKAEAAQKDGRF
KDEIVPFIVKGRKGDITVDADEYIRHGATLDSMAKLRPAFDKEGTVTAGNASGLNDGAAAALLMS
EAEASRRGIQPLGRIVSWATVGVDPKVMGTGPIPASRKALERAGWKIGDLDLVEANEAFAAQACA
VNKDLGWDPSIVNVNGGAIAIGNPIGASGARILNTLLFEMKRRGARKGLATLCIGGGMGVAMCIE
SL
>1E2XA
FADRMVIKAQSPAGFAEEYIIESIWNNRFPPGTILPAERELSELIGVTRTTLREVLQRLARDGWL
TIQHGKPTKVNNFWETSGLNILETLARLDHESVPQLIDNLLSVRTNISTIFIRTAFRQHPDKAQE
VLATANEVADHADAFAELDYNIFRGLAFASGNPIYGLILNGMKGLYTRIGRHYFANPEARSLALG
FYHKLSALCSEGAHDQVYETVRRYGHESGEIWHRMQKNLPGDLAIQGR
>2O7IA

MQVSLPREDTVYIGGALWGPATTWNLYAPQSTWGTDQFMYLPAFQYDLGRDAWIPVIAERYEFVD
DKTLRIYIRPEARWSDGVPITADDFVYALELTKELGIGPGGGWDTYIEYVKAVDTKVVEFKAKEE
NLNYFQFLSYSLGAQPMPKHVYERIRAQMNIKDWINDKPEEQVVSGPYKLYYYDPNIVVYQRVDD
WWGKDIFGLPRPKYLAHVIYKDNPSASLAFERGDIDWNGLFIPSVWELWEKKGLPVGTWYKKEPY
FIPDGVGFVYVNNTKPGLSDPAVRKAIAYAIPYNEMLKKAYFGYGSQAHPSMVIDLFEPYKQYID
YELAKKTFGTEDGRIPFDLDMANKILDEAGYKKGPDGVRVGPDGTKLGPYTISVPYGWTDWMMMC
EMIAKNLRSIGIDVKTEFPDFSVWADRMTKGTFDLIISWSVGPSFDHPFNIYRFVLDKRLSKPVG
EVTWAGDWERYDNDEVVELLDKAVSTLDPEVRKQAYFRIQQIIYRDMPSIPAFYTAHWYEYSTKY
WINWPSEDNPAWFRPSPWHADAWPTLFIISKKSDPQPVPSWLGTVDEGGIEIPTAKIFEDLQKAT
MHHHHHH
>1YU0A
MSTAVQFRGGTTAQHATFTGAAREITVDTDKNTVVVHDGATAGGFPLARHDLVKTAFIKADKSAV
AFTRTGNATASIKAGTIVEVNGKLVQFTADTAITMPALTAGTDYAIYVCDDGTVRADSNFSAPTG
YTSTTARKVGGFHYAPGSNAAAQAGGNTTAQINEYSLWDIKFRPAALDPRGMTLVAGAFWADIYL
LGVNHLTDGTSKYNVTIADGSASPKKSTKFGGDGSAAYSDGAWYNFAEVMTHHGKRLPNYNEFQA
LAFGTTEATSSGGTDVPTTGVNGTGATSAWNIFTSKWGVVQASGCLWTWGNEFGGVNGASEYTAN
TGGRGSVYAQPAAALFGGAWNGTSLSGSRAALWYSGPSFSFAFFGARGVCDHLILE
>1YUKA
QECTKFKVSSCRECIESGPGCTWCQKLNFTGPGDPDSIRCDTRPQLLMRGCAADDIMDPTSLAET
QEDHNGGQKQLSPQKVTLYLRPGQAAAFNVTFRRAKGY
>2QNDA
ASRFHEQFIVREDLMGLAIGTHGANIQQARKVPGVTAIDLDEDTCTFHIYGEDQDAVKKARSFLE
FAEDVIQVPRNLVGKVIGKNGKLIQEIVDKSGVVRVRIEAENEKNVPQEEGMVPFVFVGTKDSIA
NATVLLDYHLNYLK
>3EOFA
GMMDTVKNRRTIRKYQQKDITPDLLNDLLETSFRASTMGGMQLYSVVVTRDAEKKEILSPAHFNQ
PMVKEAPVVLTFCADFRRFCKYCQERNAVPGYGNLMSFLNAAMDTLLVAQTFCTLAEEAGLGICY
LGTTTYNPQMIIDALHLPELVFPITTVTVGYPAESPKQVDRLPIEGIIHEESYHDYTAEDINRLY
AYKESLPENKLFIEENQKETLPQVFTDVRYTKKDNEFMSENLLKVLRRQGFMD
>2GRRA
GSHMSGIALSRLAQERKAWRKDHPFGFVAVPTKNPDGTMNLMNWECAIPGKKGTPWEGGLFKLRM
LFKDDYPSSPPKCKFEPPLFHPNVYPSGTVCLSILEEDKDWRPAITIKQILLGIQELLNEPNIQS
PAQAEAYTIYCQNRVEYEKRVRAQAKKFAPS
>3EJVA
MGSDKIHHHHHHENLYFQGMTMADETIIILNVLGQYTRAHDRRDPDAMAALFAPEATIEIVDAVGG
ASRSISRLEGRDAIRVAVRQMMAPHGYRAWSQNVVNAPIIVIEGDHAVLDAQFMVFSILAAEVPD
GGWPTGTFGAQGRIVPIEAGQYRLTLRTVADGWVISAMRIEHRLPMAFG
>3GWBA
ELDGKAPSHRNLNVQTWSTAEGAKVLFVEARELPMFDLRLIFAAGSSQDGNAPGVALLTNAMLNE
GVAGKDVGAIAQGFEGLGADFGNGAYKDMAVASLRSLSAVDKREPALKLFAEVVGKPTFPADSLA
RIKNQMLAGFEYQKQNPGKLASLELMKRLYGTHPYAHASDGDAKSIPPITLAQLKAFHAKAYAAG
NVVIALVGDLSRSDAEAIAAQVSAALPKGPALAKIEQPAEPKASIGHIEFPSSQTSLMLAQLGID
RDDPDYAAVSLGNQILGGGGFGTRLMSEVREKRGLTYGVYSGFTPMQARGPFMINLQTRAEMSEG
TLKLVQDVFAEYLKNGPTQKELDDAKRELAGSFPLSTASNADIVGQLGAMGFYNLPLSYLEDFMR

QSQELTVEQVKAAMNKHLNVDKMVIVSAGPTVAQKPLEHHHHHH
>1GWTA
MQLTPTFYDNSCPNVSNIVRDTIVNELRSDPRIAASILRLHFHDCFVNGCDASILLDNTTSFRTE
KDAFGNANSARGFPVIDRMKAAVESACPRTVSCADLLTIAAQQSVTLAGGPSWRVPLGRRDSLQA
FLDLANANLPAPFFTLPQLKDSFRNVGLNRSSDLVALSGGHTFGKNQCRFIMDRLYNFSNTGLPD
PTLNTTYLQTLRGLCPLNGNLSALVDMDLRTPTIFDNKYYVNLEEQKGLIQSDQELFSSPNATDT
IPLVRSFANSTQTFFNAFVEAMDRMGNITPLTGTQGQIRLNCRVVNSNS
>1E39A
ADNLAEFHVQNQECDSCHTPDGELSNDSLTYENTQCVSCHGTLAEVAETTKHEHYNAHASHFPGE
VACTSCHSAHEKSMVYCDSCHSFDFNMPYAKKWLRDEPTIAELAKDKSERQAALASAPHDTVDVV
VVGSGGAGFSAAISATDSGAKVILIEKEPVIGGNAKLAAGGMNAAWTDQQKAKKITDSPELMFED
TMKGGQNINDPALVKVLSSHSKDSVDWMTAMGADLTDVGMMGGASVNRAHRPTGGAGVGAHVVQV
LYDNAVKRNIDLRMNTRGIEVLKDDKGTVKGILVKGMYKGYYWVKADAVILATGGFAKNNERVAK
LDPSLKGFISTNQPGAVGDGLDVAENAGGALKDMQYIQAAPTLSVKGGVMVTEAVRGNGAILVNR
EGKRFVNEITTRDKASAAILAQTGKSAYLIFDDSVRKSLSKIDKYIGLGVAPTADSLVKLGKMEG
IDGKALTETVARYNSLVSSGKDTDFERPNLPRALNEGNYYAIEVTPGVHHTMGGVMIDTKAEVMN
AKKQVIPGLYGAGEVTGGVHGANRLGGNAISDIITFGRLAGEEAAKYSKKN
>3E3UA
MAVVPIRIVGDPVLHTATTPVTVAADGSLPADLAQLIATMYDTMDAANGVGLAANQIGCSLRLFV
YDCAADRAMTARRRGVVINPVLETSEIPETMPDPDTDDEGCLSVPGESFPTGRAKWARVTGLDAD
GSPVSIEGTGLFARMLQHETGHLDGFLYLDRLIGRYARNAKRAVKSHGWGVPGLSWLPGEDPDPF
GH
>1E30A
GTLDTTWKEATLPQVKAMLEKDTGKVSGDTVTYSGKTVHVVAAAVLPGFPFPSFEVHDKKNPTLE
IPAGATVDVTFINTNKGFGHSFDITKKGPPYAVMPVIDPIVAGTGFSPVPKDGKFGYTNFTWHPT
AGTYYYVCQIPGHAATGQFGKIVVK
>3K2CA
MAHHHHHHMGTLEAQTQGPGSMAKEASGNVYFDVYANEESLGRIVMKLEDDIVPKTAKNFRTLCE
RPKGEGYKGSTFHRIIPGFMVQGGDYTAHNGTGGRSIYGEKFPDENFELKHTKEGILSMANCGAH
TNGSQFFITLGKTQWLDEKHVVFGEVVEGMDVVHKIAKYGSESGQVKKGYRIEIRDCGVLGSN
>2R1BA
GSSLRGGHAGTTYIFSKGGGQITYKWPPNDRPSTRADRLAIGFSTVQKEAVLVRVDSSSGLGDYL
ELHIHQGKIGVKFNVGTDDIAIEESNAIINDGKYHVVRFTRSGGNATLQVDSWPVIERYPAGNND
NERLAIARQRIPYRLGRVVDEWLLDKGRQLTIFNSQATIIIGGKEQGQPFQGQLSGLYYNGLKVL
NMAAENDANIAIVGNVRLVGEVPSS
>1I9YA
YDPIHEYVNHELRKRENEFSEHKNVKIFVASYNLNGCSATTKLENWLFPENTPLADIYVVGFQEI
VQLTPQQVISADPAKRREWESCVKRLLNGKCTSGPGYVQLRSGQLVGTALMIFCKESCLPSIKNV
EGTVKKTGLGGVSGNKGAVAIRFDYEDTGLCFITSHLAAGYTNYDERDHDYRTIASGLRFRRGRS
IFNHDYVVWFGDFNYRISLTYEEVVPCIAQGKLSYLFEYDQLNKQMLTGKVFPFFSELPITFPPT
YKFDIGTDIYDTSDKHRVPAWTDRILYRGELVPHSYQSVPLYYSDHRPIYATYEANIVKVDREKK
KILFEELYNQRKQEVRDASQTS
>1OAIA
PTLSPEQQEMLQAFSTQSGMNLEWSQKCLQDNNWDYTRSAQAFTHLKAKGEIPEVAFMK

>3C1JA

APAVADKADNAFMMICTALVLFMTIPGIALFYGGLIRGKNVLSMLTQVTVTFALVCILWVVYGYS
LAFGEGNNFFGNINWLMLKNIELTAVMGSIYQYIHVAFQGSAACITVGLIVGALAERIRFSAVLI
FVVVWLTLSYIPIAHMVWGGGLLASHGALDFAGGTVVHINAAIAGLVGAYLIGKRVGFGKEAFKP
HNLPMVFTGTAILYIGWFGANAGSAGTANEIAALAFVNTVVATAAAILGWIFGEWALRGKPSLLG
ACSGAIAGLVGVTPACGYIGVGGALIIGVVAGLAGLWGVTMLKRLLRVDDPCDVFGVHGVCGIVG
CIMTGIFAASSLGGVGFAEGVTMGHQLLVQLESIAITIVWSGVVAFIGYKLADLTVGLRVPEEQE
REGLDVNSHGENAYNADQAQQPAQADLEHHHHHH

>2C15A

MSFTPANRAYPYTRLRRNRRDDFSRRLVRENVLTVDDLILPVFVLDGVNQRESIPSMPGVERLSI
DQLLIEAEEWVALGIPALALFPVTPVEKKSLDAAEAYNPEGIAQRATRALRERFPELGIITDVAL
DPFTTHGQDGILDDDGYVLNDVSIDVLVRQALSHAEAGAQVVAPSDMMDGRIGAIREALESAGHT
NVRVMAYSAKYASAYYGPFRDAVGSASNLGKGNKATYQMDPANSDEALHEVAADLAEGADMVMVX
PGMPYLDIVRRVKDEFRAPTFVYQVSGEYAMHMGAIQNGWLAESVILESLTAFKRAGADGILTYF
AKQAAEQLRRGR

>1QL3A

ADPAAGEKVFGKCKACHKLDGNDGVGPHLNGVVGRTVAGVDGFNYSDPMKAHGGDWTPEALQEFL
TNPKAVVKGTKMAFAGLPKIEDRANLIAYLEGQQ

>1CHMA

QMPKTLRIRNGDKVRSTFSAQEYANRQARLRAHLAAENIDAAIFTSYHNINYYSDFLYCSFGRPY
ALVVTEDDVISISANIDGGQPWRRTVGTDNIVYTDWQRDNYFAAIQQALPKARRIGIEHDHLNLQ
NRDKLAARYPDAELVDVAAACMRMRMIKSAEEHVMIRHGARIADIGGAAVVEALGDQVPEYEVAL
HATQAMVRAIADTFEDVELMDTWTWFQSGINTDGAHNPVTTRKVNKGDILSLNCFPMIAGYYTAL
ERTLFLDHCSDDHLRLWQVNVEVHEAGLKLIKPGARCSDIARELNEIFLKHDVLQYRTFGYGHSF
GTLSHYYGREAGLELREDIDTVLEPGMVVSMEPMIMLPEGLPGAGGYREHDILIVNENGAENITK
FPYGPEKNIIR

>3CZVA

GSMSRLSWGYREHNGPIHWKEFFPIADGDQQSPIEIKTKEVKYDSSLRPLSIKYDPSSAKIISNS
GHSFNVDFDDTENKSVLRGGPLTGSYRLRQVHLHWGSADDHGSEHIVDGVSYAAELHVVHWNSDK
YPSFVEAAHEPDGLAVLGVFLQIGEPNSQLQKITDTLDSIKEKGKQTRFTNFDLLSLLPPSWDYW
TYPGSLTVPPLLESVTWIVLKQPINISSQQLAKFRSLLCTAEGEAAAFLVSNHRPPQPLKGRKVR
ASFH

>1CZYA

AMADLEQKVLEMEASTYDGVFIWKISDFPRKRQEAVAGRIPAIFSPAFYTSRYGYKMCLRIYLNG
DGTGRGTHLSLFFVVMKGPNDALLRWPFNQKVTLMLLDQNNREHVIDAFRPDVTSSSFQRPVNDM
NIASGCPLFCPVSKMEAKNSYVRDDAIFIKAIVDLTGL

>2V5IA

MVSVGDAAFRQEANKKFKYSVKLSDYSTLQDAVTDAVDGLLIDINYNFTDGESVDFXGKILTINC
KAKFIGDGALIFNNMGPGSVINQPFMESKTTPWVIFPWDADGKWITDAALVAATLKQSKIEGYQP
GVNDWVKFPGLEALLPQNVKDQHIAATLDIRSASRVEIRNAGGLMAAYLFRSCHHCKVIDSDSII
GGKDGIITFENLSGDWGLGNYVIGGRVHYGSGSGVQFLRNNGGESHNGGVIGVTSWRAGESGFKT
YQGSVGGGTARNYNLQFRDSVALSPVWDGFDLGSDPGMAPEPDRPGDLPVSEYPFHQLPNNHLVD
NILVMNSLGVGLGMDGSGGYVSNVTVQDCAGAGMLAHTYNRVFSNITVIDCNYLNFDSDQIIIIG
DCIVNGIRAAGIKPQPSNGLVISAPNSTISGLVGNVPPDKILVGNLLDPVLGQSRVIGFNSDTAE

LALRINKLSATLDSGALRSHLNGYAGSGSAWTELTALSGSTPNAVSLKVNRGDYKTTEIPISGTV
LPDEGVLDINTMSLYLDAGALWALIRLPDGSKTRMKLSV
>1YCLA
PSVESFELDHNAVVAPYVRHCGVHKVGTDGVVNKFDIRFCQPNKQAMKPDTIHTLEHLLAFTIRS
HAEKYDHFDIIDISPMGAQTGYYLVVSGEPTSAEIVDLLEDTMKEAVEITEIPAANEKQCGQAKL
HDLEGAKRLMRFWLSQDKEELLKVFG
>2G8FA
GSHMAKEEIIWESLSVDVGSQGNPGIVEYKGVDTKTGEVLFEREPIPIGTNNMGEFLAIVHGLRY
LKERNSRKPIYSDSQTAIKWVKDKKAKSTLVRNEETALIWKLVDEAEEWLNTHTYETPILKWQTD
KWGAIKADYGRK
>1G8KB
RTTLAYPATAVSVAKNLAANEPVSFTYPDTSSPCVAVKLGAPVPGGVGPDDDIVAYSVLCTHMGC
PTSYDSSSKTFSCPCHFTEFDAEKAGQMICGEATADLPRVLLRYDAASDALTAVGVDGLIYGRQA
NVI
>2NS9A
SLRLHAGVWGLKVRYEGSFEVSKTPEEVFEFLTDPKRFSRAFPGFKSVEVEDGSFTIELRLSLGP
LRGDARVRASFEDLEKPSKATVKGSGRGAGSTLDFTLRFAVEPSGGGSRVSWVFEGNVGGLAASM
GGRVLDSLARRMINDVISGVKRELGEA
>2V9MA
MQNITQSWFVQGMIKATTDAWLKGWDERNGGNLTLRLDDADIAPYHDNFHQQPRYIPLSQPMPLL
ANTPFIVTGSGKFFRNVQLDPMANLGIVKVDSDGAGYHILWGLFNEAVPTSELPAHFLSHCERIK
ATNGKDRVIMHCHATNLIALTYVLENDTAVFTRQLWEGSTECLVVFPDGVGILPWMVPGTDAIGQ
ATAQEMQKHSLVLWPFHGVFGSGPTLDETFGLIDTAEKSAQVLVKVYSMGGMKQTISREELIALG
KRFGVTPLASALAL
>2QIQA
AGFRKMAFPSGKVEGCMVQVTCGTTTLNGLWLDDTVYCPRHVICTAEDMLNPNYEDLLIRKSNHS
FLVQAGNVQLRVIGHSMQNCLLRLKVDTSNPKTPKYKFVRIQPGQTFSVLACYNGSPSGVYQCAM
RPNHTIKGSFLNGSCGSVGFNIDYDCVSFCYMHHMELPTGVHAGTDLEGKFYGPFVDRQTAQAAG
TDTTITLNVLAWLYAAVINGDRWFLNRFTTTLNDFNLVAMKYNYEPLTQDHVDILGPLSAQTGIA
VLDMCAALKELLQNGMNGRTILGSTILEDEFTPFDVVRQCS
>2Z25A
TAPSQVLKIRRPDDWHLHLRDGDMLKTVVPYTSEIYGRAIVMPNLAPPVTTVEAAVAYRQRILDA
VPAGHDFTPLMTCYLTDSLDPNELERGFNEGVFTAAKLYPANATVNSSHGVTSVDAIMPVLERME
KIGMPLLVHGEVTHADIDIFDREARFIESVMEPLRQRLTALKVVFEHITTKDAADYVRDGNERLA
ATITPQHLMFNRNHMLVGGVRPHLYCLPILKRNIHQQALRELVASGFNRVFLGTDSAPHARHRKE
SSCGCAGCFNAPTALGSYATVFEEMNALQHFEAFCSVNGPQFYGLPVNDTFIELVREEQQVAESI
ALTDDTLVPFLAGETVRWSVKQ
>3NZNA
SNAVNLFGQKDRGNHVSGVDRGKVIMYGLSTCVWCKKTKKLLTDLGVDFDYVYVDRLEGKEEEEA
VEEVRRFNPSVSFPTTIINDEKAIVGFKEKEIRESLGF
>1YPQA
RVANCSAPCPQDWIWHGENCYLFSSGSFNWEKSQEKCLSLDAKLLKINSTADLDFIQQAISYSSF
PFWMGLSRRNPSYPWLWEDGSPLMPHLFRVRGAVSQTYPSGTCAYIQRGAVYAENCILAAFSICQ
KKANL

>3ENUA

TIEVPVLTFVPVQVSAELENRGCWVKFFDKKNFQGDSLFLSGPATLPRLIGPFGYDWENKVRSVK
VGPRANLTIFDNHNYRDEDKFLDAGANVANLSKEMGFFDNFRSMVLNCI

>3APAA

GSARSSSYSGEYGSGGGKRFSHSGNQLDGPITALRVRVNTYYIVGLQVRYGKVWSDYVGGRNGDL
EEIFLHPGESVIQVSGKYKWYLKKLVFVTDKGRYLSFGKDSGTSFNAVPLHPNTVLRFISGRSGS
LIDAIGLHWDV

>2Q5XA

GIILTKVGYYTIPSMDDLAKITNEKGECIVSDFTIGRKGYGSIYFEGDVNLTNLNLDDIVHIRRK
EVVVYLDDNQKPPVGEGLNRKAEVTLDGVWPTDKTSRCLIKSPDRLADINYEGRLEAVSRKQGAQ
FKEYRPETGSWVFKVSHFAKYGLQD

>2Q66A

KVFGITGPVSTVGATAAENKLNDSLIQELKKEGSFETEQETANRVQVLKILQELAQRFVYEVSKK
KNMSDGMARDAGGKIFTYGSYRLGVHGPGSDIDTLVVVPKHVTREDFFTVFDSLLRERKELDEIA
PVPDAFVPIIKIKFSGISIALICARLDQPQVPLSLTLSDKNLLRNLDEKDLRALNGTRVTDEILE
LVPKPNVFRIALRAIKLWAQRRAVYANIFGFPGGVAWAMLVARICQLYPNACSAVILNRFFIILS
EWNWPQPVILKPIEDGPLQVRVWNPKIYAQDRSHRMPVITPAYPSMCATHNITESTKKVILQEFV
RGVQITNDIFSNKKSWANLFEKNDFFFRYKFYLEITAYTRGSDEQHLKWSGLVESKVRLLVMKLE
VLAGIKIAHPFTKPFESSYCCPTEDDYEMIQDKYGSHKTETALNALKLVTDENKEEESIKDAPKA
YLSTMYIGLDFNIENKKEKVDIHIPCTEFVNLCRSFNEDYGDHKVFNLALRFVKGYDLPDEVFDE
NEKRP

>1DQEA

SQEVMKNLSLNFGKALDECKKEMTLTDAINEDFYNFWKEGYEIKNRETGCAIMCLSTKLNMLDPE
GNLHHGNAMEFAKKHGADETMAQQLIDIVHGCEKSTPANDDKCIWTLGVATCFKAEIHKLNWAPS
MDVAVGE

>1PC5A

MAFVVTDNCIKCKYTDCVEVCPVDCFYEGPNFLVIHPDECIDCALCEPECGAQAIFSEDEVPEDM
QEFIQLNAELAEVWPNITEKKDPLPDAEDWDGVKGKLQHLER

>2FW6A

MMSETAPLPSASSALEDKAASAPVVGIIMGSQSDWETMRHADALLTELEIPHETLIVSANRTPDR
LADYARTAAERGLNVIIAGAGGAAHLPGMCAAWTRLPVLGVPVESRALKGMDSLLSIVQMPGGVP
VGTLAIGASGAKNAALLAASILALYNPALAARLETWRALQTASVPNSPITEDK

>2QQRA

GHMQSITAGQKVISKHKNGRFYQCEVVRLTTETFYEVNFDDGSFSDNLYPEDIVSQDCLQFGPPA
EGEVVQVRWTDGQVYGAKFVASHPIQMYQVEFEDGSQLVVKRDDVYTLDEELP

>1BF6A

SFDPTGYTLAHEHLHIDLSGFKNNVDCRLDQYAFICQEMNDLMTRGVRNVIEMTNRYMGRNAQFM
LDVMRETGINVVACTGYYQDAFFPEHVATRSVQELAQEMVDEIEQGIDGTELKAGIIAEIGTSEG
KITPLEEKVFIAAALAHNQTGRPISTHTSFSTMGLEQLALLQAHGVDLSRVTVGHCDLKDNLDNI
LKMIDLGAYVQFDTIGKNSYYPDEKRIAMLHALRDRGLLNRVMLSMDITRRSHLKANGGYGYDYL
LTTFIPQLRQSGFSQADVDVMLRENPSQFFQ

>3AHYA

MHHHHHHMLPKDFQWGFATAAYQIEGAVDQDGRGPSIWDTFCAQPGKIADGSSGVTACDSYNRTA
EDIALLKSLGAKSYRFSISWSRIIPEGGRGDAVNQAGIDHYVKFVDDLLDAGITPFITLFHWDLP

EGLHQRYGGLLNRTEFPLDFENYARVMFRALPKVRNWITFNEPLCSAIPGYGSGTFAPGRQSTSE
PWTVGHNILVAHGRAVKAYRDDFKPASGDGQIGIVLNGDFTYPWDAADPADKEAAERRLEFFTAW
FADPIYLGDYPASMRKQLGDRLPTFTPEERALVHGSNDFYGMNHYTSNYIRHRSSPASADDTVGN
VDVLFTNKQGNCIGPETQSPWLRPCAAGFRDFLVWISKRYGYPPIYVTENGTSIKGESDLPKEKI
LEDDFRVKYYNEYIRAMVTAVELDGVNVKGYFAWSLMDNFEWADGYVTRFGVTYVDYENGQKRFP
KKSAKSLKPLFDELIAAA
>2AHEA
MALSMPLNGLKEEDKEPLIELFVKAGSDGESIGNCPFSQRLFMILWLKGVVFSVTTVDLKRKPAD
LQNLAPGTHPPFITFNSEVKTDVNKIEEFLEEVLCPPKYLKLSPKHPESNTAGMDIFAKFSAYIK
NSRPEANEALERGLLKTLQKLDEYLNSPLPDEIDENSMEDIKFSTRKFLDGNEMTLADCNLLPKL
HIVKVVAKKYRNFDIPKEMTGIWRYLTNAYSRDEFTNTCPSDKEVEIAYSDVAKRLPSKVPKGEF
QHTGGRY
>1OX3A
ADIVLNDLPFVDGPPAEGQSRISWIKNGEEILGADTQYGSEGSMNRPTVSVLRNVEVLDKNIGIL
KTSLETANSDIKTIQEAGYIPEAPRDGQAYVRKDGEWVLLSTFL
>1QZ0A
MRERPHTSGHHGAGEARATAPSTVSPYGPEARAELSSRLTTLRNTLAPATNDPRYLQACGGEKLN
RFRDIQCRRQTAVRADLNANYIQVGNTRTIACQYPLQSQLESHFRMLAENRTPVLAVLASSSEIA
NQRFGMPDYFRQSGTYGSITVESKMTQQVGLGDGIMADMYTLTIREAGQKTISVPVVHVGNWPDQ
TAVSSEVTKALASLVDQTAETKRNMYESKGSSAVADDSKLRPVIHCRAGVGRTAQLIGAMCMNDS
RNSQLSVEDMVSQMRVQRNGIMVQKDEQLDVLIKLAEGQGRPLLNS
>1LT4A
NGDRLYRADSRPPDEIKRSGGLMPRGHNEYFDRGTQMNINLYDHARGTQTGFVRYDDGYVSTKLS
LRSAHLAGQSILSGYSTYYIYVIATAPNMFNVNDVLGVYSPHPYEQEVSALGGIPYSQIYGWYRV
NFGVIDERLHRNREYRDRYYRNLNIAPAEDGYRLAGFPPDHQAWREEPWIHHAPQGCGNSSNSSR
TITRTITGDTCNEETQNLSTIYLREYQSKVKRQIFSDYQSEVDIYNRIRDEL
>1OCBA
YNGNPFEGVQLWANNYYRSEVHTLAIPQITDPALRAAASAVAEVPSFQWLDRNVTVDTLLVQTLS
EIREANQAGANPQYAAQIVVYDLPDRDCAAAASNGEWAIANNGVNNYKAYINRIREILISFSDVR
TILVIEPDSLANMVTNMNVPKCSGAASTYRELTIYALKQLDLPHVAMYMDAGHAGWLGWPANIQP
AAELFAKIYEDAGKPRAVRGLATNVANYNAWSVSSPPPYTSPNPNYDEKHYIEAFRPLLEARGFP
AQFIVDQGRSGKQPTGQKEWGHWCNAIGTGFGMRPTANTGHQYVDAFVWVKPGGECDGTSDTTAA
RYDYHCGLEDALKPAPEAGQWFNEYFIQLLRNANPPF
>3OCCA
MATPHINAEMGDFADVVLMPGDPLRAKFIAETFLQDVREVNNVRGMLGFTGTYKGRKISVMGHGM
GIPSCSIYAKELITDFGVKKIIRVGSCGAVRTDVKLRDVVIGMGACTDSKVNRMRFKDHDYAAIA
DFEMTRNAVDAAKAKGVNVRVGNLFSADLFYTPDPQMFDVMEKYGILGVEMEAAGIYGVAAEFGA
KALTICTVSDHIRTGEQTTAAERQTTFNDMIEIALESVLLGDNA
>1ZCJA
ASGQAKALQYAFFAEKSANKWSTPSGASWKTASAQPVSSVGVLGLGTMGRGIAISFARVGISVVA
VESDPKQLDAAKKIITFTLEKEASRAHQNGQASAKPKLRFSSSTKELSTVDLVVEAVFEDMNLKK
KVFAELSALCKPGAFLCTNTSALNVDDIASSTDRPQLVIGTHFFSPAHVMRLLEVIPSRYSSPTT
IATVMSLSKKIGKIGVVVGNCYGFVGNRMLAPYYNQGFFLLEEGSKPEDVDGVLEEFGFKMGPFR
VSDLAGLDVGWKIRKGQGLTGPSLPPGTPVRKRGNSRYSPLGDMLCEAGRFGQKTGKGWYQYDKP

LGRIHKPDPWLSTFLSQYREVHHIEQRTISKEEILERCLYSLINEAFRILEEGMAARPEHIDVIY
LHGYGWPRHKGGPMFYAASVGLPTVLEKLQKYYRQNPDIPQLEPSDYLRRLVAQGSPPLKEWQSL
AGPHGSKL
>1LVMA
GHHHHHHHGESLFKGPRDYNPISSTICHLTNESDGHTTSLYGIGFGPFIITNKHLFRRNNGTLLV
QSLHGVFKVKNTTTLQQHLIDGRDMIIIRMPKDFPPFPQKLKFREPQREERICLVTTNFQTKSMS
SMVSDTSCTFPSSDGIFWKHWIQTKDGQCGSPLVSTRDGFIVGIHSASNFTNTNNYFTSVPKNFM
ELLTNQEAQQWVSGWRLNADSVLWGGHKVFMDKP
>1E0WA
AESTLGAAAAQSGRYFGTAIASGRLSDSTYTSIAGREFNMVTAENEMKIDATEPQRGQFNFSSAD
RVYNWAVQNGKQVRGHTLAWHSQQPGWMQSLSGSALRQAMIDHINGVMAHYKGKIVQWDVVNEAF
ADGSSGARRDSNLQRSGNDWIEVAFRTARAADPSAKLCYNDYNVENWTWAKTQAMYNMVRDFKQR
GVPIDCVGFQSHFNSGSPYNSNFRTTLQNFAALGVDVAITELDIQGAPASTYANVTNDCLAVSRC
LGITVWGVRDSDSWRSEQTPLLFNNDGSKKAAYTAVLDALNGGDSSEPPADGG
>3EMHA
GPLGSPEFQSKPTPVKPNYALKFTLAGHTKAVSSVKFSPNGEWLASSSADKLIKIWGAYDGKFEK
TISGHKLGISDVAWSSDSNLLVSASDDKTLKIWDVSSGKCLKTLKGHSNYVFCCNFNPQSNLIVS
GSFDESVRIWDVKTGKCLKTLPAHSDPVSAVHFNRDGSLIVSSSYDGLCRIWDTASGQCLKTLID
DDNPPVSFVKFSPNGKYILAATLDNTLKLWDYSKGKCLKTYTGHKNEKYCIFANFSVTGGKWIVS
GSEDNLVYIWNLQTKEIVQKLQGHTDVVISTACHPTENIIASAALENDKTIKLWKSDC
>3G5JA
SNAMSVIKIEKALKLDKVIFVDVRTEGEYEEDHILNAINMPLFKNNEHNEVGTIYKMQGKHEAIQ
KGFDYVSYKLKDIYLQAAELALNYDNIVIYCARGGMRSGSIVNLLSSLGVNVYQLEGGYKAYRNF
VLEY
>3B7AA
MTMEQFLTSLDMIRSGCAPKFKLKTEDLDRLRVGDFNFPPSQDLMCYTKCVALMAGTVNKKGEFN
APKALAQLPHLVPPEMMEMSRKSVEACRDTHKQFKESCERVYQTAKCFSENADGQFMWP
>3B7CA
GMPTDDIVQLLKGQEEAWNRGDLDAYMQGYWQNEQLMLISNGKFRNGWDETLAAYKKNYPDKESL
GELKFTIKEIKMLSNYAAMVVGRWDLKRLKDTPTGVFTLLVEKIDDRWVITMDHSSD
>2AD6A
DADLDKQVNTAGAWPIATGGYYSQHNSPLAQINKSNVKNVKAAWSFSTGVLNGHEGAPLVIGDMM
YVHSAFPNNTYALNLNDPGKIVWQHKPKQDASTKAVMCCDVVDRGLAYGAGQIVKKQANGHLLAL
DAKTGKINWEVEVCDPKVGSTLTQAPFVAKDTVLMGCSGAELGVRGAVNAFDLKTGELKWRAFAT
GSDDSVRLAKDFNSANPHYGQFGLGTKTWEGDAWKIGGGTNWGWYAYDPKLNLFYYGSGNPAPWN
ETMRPGDNKWTMTIWGRDLDTGMAKWGYQKTPHDEWDFAGVNQMVLTDQPVNGKMTPLLSHIDRN
GILYTLNRENGNLIVAEKVDPAVNVFKKVDLKTGTPVRDPEFATRMDHKGTNICPSAMGFHNQGV
DSYDPESRTLYAGLNHICMDWEPFMLPYRAGQFFVGATLAMYPGPNGPTKKEMGQIRAFDLTTGK
AKWTKWEKFAAWGGTLYTKGGLVWYATLDGYLKALDNKDGKELWNFKMPSGGIGSPMTYSFKGKQ
YIGSMYGVGGWPGVGLVFDLTDPSAGLGAVGAFRELQNHTQMGGGLMVFSL
>1WSRA
AQEVLRRTPLYDFHLAHGGKMVAFAGWSLPVQYRDSHTDSHLHTRQHCSLFDVSHMLQTKILGSD
RVKLMESLVVGDIAELRPNQGTLSLFTNEAGGILDDLIVTNTSEGHLYVVSNAGCWEKDLALMQD
KVRELQNQGRDVGLEVLDNALLALQGPTAAQVLQAGVADDLRKLPFMTSAVMEVFGVSGCRVTRC

GYTGEDGVEISVPVAGAVHLATAILKNPEVKLAGLAARDSLRLEAGLCLYGNDIDEHTTPVEGSL
SWTLGKRRRAAMDFPGAKVIVPQLKGRVQRRRVGLMCEGAPMRAHSPILNMEGTKIGTVTSGCPS
PSLKKNVAMGYVPCEYSRPGTMLLVEVRRKQQMAVVSKMPFVPTNYYTLK
>1BCH1
AIEVKLANMEAEINTLKSKLELTNKLHAFSMGKKSGKKFFVTNHERMPFSKVKALCSELRGTVAI
PRNAEENKAIQEVAKTSAFLGITDEVTEGQFMYVTGGRLTYSNWKKDQPDDWYGHGLGGGEDCVH
IVDNGLWNDISCQASHTAVCEFPA
>1DXKA
SQKVEKTVIKNETGTISISQLNKNVWVHTELGSFNGEAVPSNGLVLNTSKGLVLVDSSWDDKLTK
ELIEMVEKKFQKRVTDVIITHAHADRIGGIKTLKERGIKAHSTALTAELAKKNGYEEPLGDLQTV
TNLKFGNMKVETFYPGKGHTEDNIVVWLPQYNILVGGSLVKSTSAKDLGNVADAYVNEWSTSIEN
VLKRYRNINAVVPGHGEVGDKGLLLHTLDLLK
>3H8TA
DEPNQPSTPEAVTKTVTIDASKYETWQYFSFSKGEVVNVTDYKNDLNWDMALHRYDVRLNCGESG
KGKGGAVFSGKTEMDQATTVPTDGYTVDVLGRITVKYEMGPDGHQMEYEEQGFSEVITGKKNAQG
FASGGWLEFSHGPAGPTYKLSKRVFFVRGADGNIAKVQFTDYQDAELKKGVITFTYTYPVK
>3M7OA
MNGVAAALLVWILTSPSSSDHGSENGWPKHTACNSGGLEVVYQSCDPLQDFGLSIDQCSKQIQSN
LNIRFGIILRQDIRKLFLDITLMAKGSSILNYSYPLCEEDQPKFSFCGRRKGEQIYYAGPVNNPG
LDVPQGEYQLLLELYNENRATVACANATVTSS
>3OYYA
MASMKTAQEFRAGQVANINGAPWVIQKAEFNKSGRNAAVVKMKLKNLLTGAGTETVFKADDKLEP
IILDRKEVTYSYFADPLYVFMDSEFNQYEIEKDDLEGVLTFIEDGMTDICEAVFYNDKVISVELP
TTIVRQIAYTEPAVRGDTSGKVMKTARLNNGAELQVSAFCEIGDSIEIDTRTGEYKSRVKA
>1FAZA
APADKPQVLASFTQTSASSQNAWLAANRNQSAWAAYEFDWSTDLCTQAPDNPFGFPFNTACARHD
FGYRNYKAAGSFDANKSRIDSAFYEDMKRVCTGYTGEKNTACNSTAWTYYQAVKIFG
>1ODZA
MRADVKPVTVKLVDSQATMETRSLFAFMQEQRRHSIMFGHQHETTQGLTITRTDGTQSDTFNAVG
DFAAVYGWDTLSIVAPKAEGDIVAQVKKAYARGGIITVSSHFDNPKTDTQKGVWPVGTSWDQTPA
VVDSLPGGAYNPVLNGYLDQVAEWANNLKDEQGRLIPVIFRLYHENTGSWFWWGDKQSTPEQYKQ
LFRYSVEYLRDVKGVRNFLYAYSPNNFWDVTEANYLERYPGDEWVDVLGFDTYGPVADNADWFRN
VVANAALVARMAEARGKIPVISGIGIRAPDIEAGLYDNQWYRKLISGLKADPDAREIAFLLVWRN
APQGVPGPNGTQVPHYWVPANRPENINNGTLEDFQAFYADEFTAFNRDIEQVYQRPTLIVK
>1PF3A
AERPTLPIPDLLTTDARNRIQLTIGAGQSTFGGKTATTWGYNGNLLGPAVKLQRGKAVTVDIYNQ
LTEETTLHWHGLEVPGEVDGGPQGIIPPGGKRSVTLNVDQPAATCWFHPHQHGKTGRQVAMGLAG
LVVIEDDEILKLMLPKQWGIDDVPVIVQDKKFSADGQIDYQLDVMTAAVGWFGDTLLTNGAIYPQ
HAAPRGWLRLRLLNGCNARSLNFATSDNRPLYVIASDGGLLPEPVKVSELPVLMGERFEVLVEVN
DNKPFDLVTLPVSQMGMAIAPFDKPHPVMRIQPIAISASGALPDTLSSLPALPSLEGLTVRKLQL
SMDPMLDMMGMQMLMEKYGDQAMAGMDHSQMMGHMGHGNMNHMNHGGKFDFHHANKINGQAFDMN
KPMFAAAKGQYERWVISGVGDMLLHPFHIHGTQFRILSENGKPPAAHRAGWKDTVKVEGNVSEVL
VKFNHDAPKEHAYMAHCHLLEHEDTGMMLGFTVSAWSHPNFEK
>1UWLA

MTDNNNYRDVEIRAPRGNKLTAKSWLTEAPLRMLMNNLDPQVAENPKELVVYGGIGRAARNWECY
DKIVETLTRLEDDETLLVQSGKPVGVFKTHSNAPRVLIANSNLVPHWANWEHFNELDAKGLAMYG
QMTAGSWIYIGSQGIVQGTYETFVEAGRQHYGGSLKGKWVLTAGLGGMGGAQPLAATLAGACSLN
IESQQSRIDFRLETRYVDEQATDLDDALVRIAKYTAEGKAISIALHGNAAEILPELVKRGVRPDM
VTDQTSAHDPLNGYLPAGWTWEQYRDRAQTEPAAVVKAAKQSMAVHVQAMLDFQKQGVPTFDYGN
NIRQMAKEEGVANAFDFPGFVPAYIRPLFCRGVGPFRWAALSGEAEDIYKTDAKVKELIPDDAHL
HRWLDMARERISFQGLPARICWVGLGLRAKLGLAFNEMVRSGELSAPVVIGRDHLDSGSVSSPNR
ETEAMRDGSDAVSDWPLLNALLNTAGGATWVSLHHGGGVGMGFSQHSGMVIVCDGTDEAAERIAR
VLTNDPGTGVMRHADAGYDIAIDCAKEQGLDLPMITG

>3MCXA

GDWLDLNTTSSVETGQAIVTLDDAQIALNGIYRLASGHSYYGDNYWYYGDCRAADVQARITKGDG
KRVSPYYEYNVLASDNLNIVLPWNTVYKVIRQTNNLIQKIESGSIQSSDTKELNRIKSEALVMRG
LSLFNLTRLFGMPYTNDKGASLGVPIETSPSDPTHKPSRSTVAQCYEQVVSDMSNALSGLRQETS
NGYINYWAAQALLSRVYLNMGEYQKAYDAATDVIKNNGGRYQLYSYEEYPNVWGQDFQSESLFEL
YITLSEPSGGTGGEGAPMVYANEATVDWNNLILSEDFLNLLNEDPKDVRHCLTKESVIENNTGLP
AAAMHEKVYLAKFPGKTGDDPKTNNICIIRLSEVYLNAAEAGLKKGTDIEEAQGYLNDIISRRTT
DTSQQVSTETFTLDRILKERRKELVGEGEVFYDYLRNGLAIERKGSWHLETLKASNAQKIEATDL
RIALPIPQSEIDANPNIQQNPR

>1Z82A

MGSDKIHHHHHHMEMRFFVLGAGSWGTVFAQMLHENGEEVILWARRKEIVDLINVSHTSPYVEES
KITVRATNDLEEIKKEDILVIAIPVQYIREHLLRLPVKPSMVLNLSKGIEIKTGKRVSEIVEEIL
GCPYAVLSGPSHAEEVAKKLPTAVTLAGENSKELQKRISTEYFRVYTCEDVVGVEIAGALKNVIA
IAAGILDGFGGWDNAKAALETRGIYEIARFGMFFGADQKTFMGLAGIGDLMVTCNSRYSRNRRFG
ELIARGFNPLKLLESSNQVVEGAFTVKAVMKIAKENKIDMPISEEVYRVVYEGKPPLQSMRDLMR
RSLKDEFWAS

>3GZRA

GGEGTDAIQALIQAYFTAWNTNAPERFAEIFWPDGSWVNVVGMHWRGRDQIVFAHTAFLKTIFKD
CKQELVTIEARTIAPGSALAVVTLIQDAYVTPDGRQMPRAHDRLTLLAVEREGVWRFIHGHNTIV
NPDAANNDPVLRMKPA

>2A33A

MEIKGESMQKSKFRRICVFCGSSQGKKSSYQDAAVDLGNELVSRNIDLVYGGGSIGLMGLVSQAV
HDGGRHVIGIIPKTLMPRELTGETVGEVRAVADMHQRKAEMAKHSDAFIALPGGYGTLEELLEVI
TWAQLGIHDKPVGLLNVDGYYNSLLSFIDKAVEEGFISPTAREIIVSAPTAKELVKKLEEYAPCH
ERVATKLCWEMERIGYSSEE

>1DHIA

MISLIAALAVDRVIGMENAMPWNLPASLAWFKRNTLDKPVIMGRHTWESIGRPLPGRKNIILSSQ
PGTDDRVTWVKSVDEAIAACGDVPEIMVIGGGRVYEQFLPKAQKLYLTHIDAEVEGDTHFPDYEP
DDWESVFSEFHDADAQNSHSYCFEILERR

>3A8GA

MSVTIDHTTENAAPAQAPVSDRAWALFRALDGKGLVPDGYVEGWKKTFEEDFSPRRGAELVARAW
TDPEFRQLLLTDGTAAVAQYGYLGPQGEYIVAVEDTPTLKNVIVCSLCACTAWPILGLPPTWYKS
FEYRARVVREPRKVLSEMGTEIASDIEIRVYDTTAETRYMVLPQRPAGTEGWSQEQLQEIVTKDC
LIGVAIPQVPTV

>1VDWA

MSVKTWRKIAIDIIRDFDHNIMPLFGNPKASETISISPSGDETKVVDKVAENIIISKFKDLGVNV
VSEEIGRIDQGSDYTVVVDPLDGSYNFINGIPFFAVSVAIFHEKDPIYAFIYEPIVERLYEGIPG
KGSYLNGEKIKVRELAEKPSISFYTKGKGTKIIDKVKRTRTLGAIALELAYLARGALDAVVDIRN
YLRPTDIAAGVVIAREAGAIVKDLDGKDVEITFSATEKVNIIAANNEELLETILRSIEK
>2QKFA
MDIKINDITLGNNSPFVLFGGINVLESLDSTLQTCAHYVEVTRKLGIPYIFKASFDKANRSSIHS
YRGVGLEEGLKIFEKVKAEFGIPVITDVHEPHQCQPVAEVCDVIQLPAFLARQTDLVVAMAKTGN
VVNIKKPQFLSPSQMKNIVEKFHEAGNGKLILCERGSSFGYDNLVVDMLGFGVMKQTCGNLPVIF
DVTHSLQTRDAGSAASGGRRAQALDLALAGMATRLAGLFLESHPDPKLAKCDGPSALPLHLLEDF
LIRIKALDDLIKSQPILTIE
>2ZU1A
GPAFEFAVAMMKRNSSTVKTEYGEFTMLGIYDRWAVLPRHAKPGPTILMNDQEVGVLDAKELVDK
DGTNLELTLLKLNRNEKFRDIRGFLAKEEVEVNEAVLAINTSKFPNMYIPVGQVTEYGFLNLGGT
PTKRMLMYNFPTRAGQAGGVLMSTGKVLGIHVGGNGHQGFSAALLKHYFNDEQ
>2GJPA
HHMGTNGTMMQYFEWHLPNDGQHWNRLRDDASNLRNRGITAIWIPPAWKGTSQNDVGYGAYDLYD
LGEFNQKGTVRTKYGTRSQLESAIHALKNNGVQVYGDVVMNHKGGADATENVLAVEVNPNNRNQE
ISGDYTIEAWTKFDFPGRGNTYSDFKWRWYHFDGVDWDQSRQFQNRIYKFRGDGKAWDWEVDSEN
GNYDYLMYADVDMDHPEVVNELRRWGEWYTNTLNLDGFRIDAVKHIKYSFTRDWLTHVRNATGKE
MFAVAEFWKNDLGALENYLNKTNWNHSVFDVPLHYNLYNASNSGGNYDMAKLLNGTVVQKHPMHA
VTFVDNHDSQPGESLESFVQEWFKPLAYALILTREQGYPSVFYGDYYGIPTHSVPAMKAKIDPIL
EARQNFAYGTQHDYFDHHNIIGWTREGNTTHPNSGLATIMSDGPGGEKWMYVGQNKAGQVWHDIT
GNKPGTVTINADGWANFSVNGGSVSIWVKR
>3B5NC
GSIKFTKQSSVASTRNTLKMAQDAERAGMNTLGMLGHQSEQLNNVEGNLDLMKVQNKVADEKVAE
LKKLQ
>1HQGA
MSSKPKPIEIIGAPFSKGQPRGGVEKGPAALRKAGLVEKLKETEYNVRDHGDLAFVDVPNDSPFQ
IVKNPRSVGKANEQLAAVVAETQKNGTISVVLGGDHSMAIGSISGHARVHPDLCVIWVDAHTDIN
TPLTTSSGNLCGQPVAFLLKELKGKFPDVPGFSWVTPCISAKDIVYIGLRDVDPGEHYIIKTLGI
KYFSMTEVDKLGIGKVMEETFSYLLGRKKRPIHLSFDVDGLDPVFTPATGTPVVGGLSYREGLYI
TEEIYKTGLLSGLDIMEVNPTLGKTPEEVTRTVNTAVALTLSCFGTKREGNHKPETDYLKPPK
>2OQYA
MKITDLELHAVGIPRHTGFVNKHVIVKIHTDEGLTGIGEMSDFSHLPLYSVDLHDLKQGLLSILL
GQNPFDLMKINKELTDNFPETMYYYEKGSFIRNGIDNALHDLCAKYLDISVSDFLGGRVKEKIKV
CYPIFRHRFSEEVESNLDVVRQKLEQGFDVFRLYVGKNLDADEEFLSRVKEEFGSRVRIKSYDFS
HLLNWKDAHRAIKRLTKYDLGLEMIESPAPRNDFDGLYQLRLKTDYPISEHVWSFKQQQEMIKKD
AIDIFNISPVFIGGLTSAKKAAYAAEVASKDVVLGTTQELSVGTAAMAHLGCSLTNINHTSDPTG
PELYVGDVVKNRVTYKDGYLYAPDRSVKGLGIELDESLLAKYQVPDLSWDNVTVHQLQDRTADTK
S
>2OQAA
DVSFSLSGSSSTSYSKFIGALRKALPSNGTVYNITLLLSSASGASRYTLMKLSNYDGKAITVAID
VTNVYIMGYLVNSTSYFFNESDAKLASQYVFAGSTIVTLPYSGNYEKLQTAAGKIREKIPLGFPA
LDSAITTLFHYDSTAAAAAFLVIIQTTAESSRFKYIEGQIIMRISKNGVPSLATISLENEWSALS

KQIQLAQTNNGTFKTPVVIMDAGGQRVEIGNVGSKVVTKNIQLLLN
>3QD5A
GPGSMAATPLPPLRLAIACDDAGVSYKEALKAHLSDNPLVSSITDVGVTSTTDKTAYPHVAIQAA
QLIKDGKVDRALMICGTGLGVAISANKVPGIRAVTAHDTFSVERAILSNDAQVLCFGQRVIGIEL
AKRLAGEWLTYRFDQKSASAQKVQAISDYEKKFVEVN
>3D1RA
GMRRELAIEFSRVTESAALAGYKWLGRGDKNTADGAAVNAMRIMLNQVNIDGTIVIGEGEIAEAP
MLYIGEKVGTGRGDAVDIAVDPIEGTRMTAMGQANALAVLAVGDKGCFLNAPDMYMEKLIVGPGA
KGTIDLNLPLADNLRNVAAALGKPLSELTVTILAKPRHDAVIAEMQQLGVRVFAIPDGDVAASIL
TCMPDSEVDVLYGIGGAPEGVVSAAVIRALDGDMNGRLLARHDVKGDNEENRRIGEQELARCKAM
GIEAGKVLRLGDMARSDNVIFSATGITKGDLLEGISRKGNIATTETLLIRGKSRTIRRIQSIHYL
DRKDPEMQVHIL
>1RWIB
RPSWSPTQASGQTVLPFTGIDFRLSPSGVAVDSAGNVYVTSEGMYGRVVKLATGSTGTTVLPFNG
LYQPQGLAVDGAGTVYVTDFNNRVVTLAAGSNNQTVLPFDGLNYPEGLAVDTQGAVYVADRGNNR
VVKLAAGSKTQTVLPFTGLNDPDGVAVDNSGNVYVTDTDNNRVVKLEAESNNQVVLPFTDITAPW
GIAVDEAGTVYVTEHNTNQVVKLLAGSTTSTVLPFTGLNTPLAVAVDSDRTVYVADRGNDRVVKL
TSLEHHHHHH
>3NXDC
GSHMGGVEVLEVKTGVDSITEVECFLTPEMGDPDEHLRGFSKSISISDTFESDSPNRDMLPCYSV
ARIPLPNLNEDLTCGNILMWEAVTLKTEVIGVTSLMNVHSNGQATHDNGAGKPVQGTSFHFFSVG
GEALELQGVLFNYRTKYPDGTIFPKNATVQSQVMNTEHKAYLDKNKAYPVECWVPDPTRNENTRY
FGTLTGGENVPPVLHITNTATTVLLDEFGVGPLCKGDNLYLSAVDVCGMFTNRSGSQQWRGLSRY
FKVQLRKRRVKN
>1TEJB
NSVNPCCDPQTCKPIEGKHCISGPCCENCYFLRSGTICQRARGDGNNDYCTGITPDCPRNRYNV
>3EWDA
MNILQEPIDFLKKEELKNIDLSQMSKKERYKIWKRIPKCELHCHLDLCFSADFFVSCIRKYNLQP
NLSDEEVLDYYLFAKGGKSLGEFVEKAIKVADIFHDYEVIEDLAKHAVFNKYKEGVVLMEFRYSP
TFVAFKYNLDIELIHQAIVKGIKEVVELLDHKIHVALMCIGTGHEAANIKASADFCLKHKADFVG
FDHGGHEVDLKEYKEIFDYVRESGVPLSVHAGEDVTLPNLNTLYSAIQVLKVERIGHGIRVAESQ
ELIDMVKEKNILLEVCPISNVLLKNAKSMDTHPIRQLYDAGVKVSVNSDDPGMFLTNINDDYEEL
YTHLNFTLEDFMKMNEWALEKSFMDSNIKDKIKNLYFKGEFEAYV
>1EW4A
MNDSEFHRLADQLWLTIEERLDDWDGDSDIDCEINGGVLTITFENGSKIIINRQEPLHQVWLATK
QGGYHFDLKGDEWICDRSGETFWDLLEQAATQQAGETVSFR
>1H6WA
LSYPNATESVYGLTRYSTNDEAIAGVNNESSITPAKFTVALNNVFETRVSTESSNGVIKISSLPQ
ALAGADDTTAMTPLKTQQLAVKLIAQIAPSKNAATESEQGVIQLATVAQARQGTLREGYAISPYT
FMNSTATEEYKGVIKLGTQSEVNSNNASVAVTGATLNGRGSTTSMRGVVKLTTTAGSQSGGDASS
ALAWNADVIHQRGGQTINGTLRINNTLTIASGGANITGTVNMTGGYIQGKRVVTQNEIDRTIPVG
AIMMWAADSLPSDAWRFCHGGTVSASDCPLYASRIGTRYGGSSSNPGLPDMR
>2CB9A
MARSQLSAAGEQHVIQLNQQGGKNLFCFPPISGFGIYFKDLALQLNHKAAVYGFHFIEEDSRIEQ

YVSRITEIQPEGPYVLLGYSAGGNLAFEVVQAMEQKGLEVSDFIIVDAYKKDQSITADTENDDSA
AYLPEAVRETVMQKKRCYQEYWAQLINEGRIKSNIHFIEAGIQTETSGAMVLQKWQDAAEEGYAE
YTGYGAHKDMLEGEFAEKNANIILNILDKINSDQKVLPNKHGSHHHHHH
>1GXJA
EKEMIERDMREYRGFSRAVRAVFEEKERFPGLVDVVSNLIEVDEKYSLAVSVLLGGTAQNIVVRN
VDTAKAIVEFLKQNEAGRVTILPLDLIDGSFNRISGLENERGFVGYAVDLVKFPSDLEVLGGFLF
GNSVVVETLDDAIRMKKKYRLNTRIATLDGELISGRGAITGGREERSSNVFERRIK
>1VLCA
MGSDKIHHHHHHMKIAVLPGDGIGPEVVREALKVLEVVEKKTGKTFEKVFGHIGGDAIDRFGEPL
PEETKKICLEADAIFLGSVGGPKWDDLPPEKRPEIGGLLALRKMLNLYANIRPIKVYRSLVHVSP
LKEKVIGSGVDLVTVRELSYGVYYGQPRGLDEEKGFDTMIYDRKTVERIARTAFEIAKNRRKKVT
SVDKANVLYSSMLWRKVVNEVAREYPDVELTHIYVDNAAMQLILKPSQFDVILTTNMFGDILSDE
SAALPGSLGLLPSASFGDKNLYEPAGGSAPDIAGKNIANPIAQILSLAMMLEHSFGMVEEARKIE
RAVELVIEEGYRTRDIAEDPEKAVSTSQMGDLICKKLEEIW
>2VLBA
GQMQQASTPTIGMIVPPAAGLVPADGARLYPDLPFIASGLGLGSVTPEGYDAVIESVVDHARRLQ
KQGAAVVSLMGTSLSFYRGAAFNAALTVAMREATGLPCTTMSTAVLNGLRALGVRRVALATAYID
DVNERLAAFLAEESLVPTGCRSLGITGVEAMARVDTATLVDLCVRAFEAAPDSDGILLSCGGLLT
LDAIPEVERRLGVPVVSSSPAGFWDAVRLAGGGAKARPGYGRLFDESGGSHHHHHH
>2VLPB
MESKRNKPGKATGKGKPVGDKWLDDAGKDSGAPIPDRIADKLRDKEFKSFDDFAKAVWEEVSKDP
ELSKNLNPSNKSSVSKGYSPFTPKNQQVGGRKVYELHHDKPISQGGEVYDMDNIRVTTPKRHIDI
HRGK
>2Y88A
MPLILLPAVNVVEGRAVRLVQGKAGSQTEYGSAVDAALGWQRDGAEWIHLVDLDAAFGRGSNHEL
LAEVVGKLDVQVELSGGIRDDESLAAALATGCARVNVGTAALENPQWCARVIGEHGDQVAVGLDV
QIIDGEHRLRGRGWETDGGDLWDVLERLDSEGCSRFVVTDITKDGTLGGPNLDLLAGVADRTDAP
VIASGGVSSLDDLRAIATLTHRGVEGAIVGKALYARRFTLPQALAAVRD
>3DV9A
SNAMFKEAINNYLHTHGYESIDLKAVLFDMDGVLFDSMPNHAESWHKIMKRFGFGLSREEAYMHE
GRTGASTINIVSRRERGHDATEEEIKAIYQAKTEEFNKCPKAERMPGALEVLTKIKSEGLTPMVV
TGSGQTSLLDRLNHNFPGIFQANLMVTAFDVKYGKPNPEPYLMALKKGGFKPNEALVIENAPLGV
QAGVAAGIFTIAVNTGPLHDNVLLNEGANLLFHSMPDFNKNWETLQSALKQD
>2QRJA
MGHHHHHHHHHHSSGHIEGRHMAAVTLHLRAETKPLEARAALTPTTVKKLIAKGFKIYVEDSPQS
TFNINEYRQAGAIIVPAGSWKTAPRDRIIIGLKEMPETDTFPLVHEHIQFAHCYKDQAGWQNVLM
RFIKGHGTLYDLEFLENDQGRRVAAFGFYAGFAGAALGVRDWAFKQTHSDDEDLPAVSPYPNEKA
LVKDVTKDYKEALATGARKPTVLIIGALGRCGSGAIDLLHKVGIPDANILKWDIKETSRGGPFDE
IPQADIFINCIYLSKPIAPFTNMEKLNNPNRRLRTVVDVSADTTNPHNPIPIYTVATVFNKPTVL
VPTTAGPKLSVISIDHLPSLLPREASEFFSHDLLPSLELLPQRKTAPVWVRAKKLFDRHCARVKR
SSRL
>2O6XA
NDDLWHQWKRMYNKEYNGADDQHRRNIWEKNVKHIQEHNLRHDLGLVTYTLGLNQFTDMTFEEFK
AKYLTEMSRASDILSHGVPYEANNRAVPDKIDWRESGYVTEVKDQGNCGSGWAFSTTGTMEGQYM

KNERTSISFSEQQLVDCSRPWGNNGCGGGLMENAYQYLKQFGLETESSYPYTAVEGQCRYNKQLG
VAKVTGFYTVHSGSEVELKNLVGAEGPAAVAVDVESDFMMYRSGIYQSQTCSPLRVNHAVLAVGY
GTQGGTDYWIVKNSWGLSWGERGYIRMVRNRGNMCGIASLASLPMVARFP
>1GQGA
DTSSLIVEDAPDHVRPYVIRHYSHARAVTVDTQLYRFYVTGPSSGYAFTLMGTNAPHSDALGVLP
HIHQKHYENFYCNKGSFQLWAQSGNETQQTRVLSSGDYGSVPRNVTHTFQIQDPDTEMTGVIVPG
GFEDLFYYLGTNATDTTHTPYIPSSSDSSSTTGPDSSTISTLQSFDVYAELSFTPRTDTVNGTAP
ANTVWHTGANALASTAGDPYFIANGWGPKYLNSQYGYQIVAPFVTATQAQDTNYTLSTISMSTTP
STVTVPTWSFPGACAFQVQEGRVVVQIGDYAATELGSGDVAFIPGGVEFKYYSEAYFSKVLFVSS
GSDGLDQNLVNGGEEWSSVSFPADW
>3MSUA
SNAMEVMLMSKYATLKYADKNIEIELPVYSPSLGNDCIDVSSLVKHGIFTYDPGFMSTAACESKI
TYIDGGKGVLLHRGYPIEEWTQKSNYRTLCYALIYGELPTDEQVKSFRQEIINKMPVCEHVKAAI
AAMPQHTHPMSSLIAGVNVLAAEHIHNGQKESQDEVAKNIVAKIATIAAMAYRHNHGKKFLEPKM
EYGYAENFLYMMFADDESYKPDELHIKAMDTIFMLHADHEQNASTSTVRLSGSTGNSPYAAIIAG
ITALWGPAHGGANEAVLKMLSEIGSTENIDKYIAKAKDKDDPFRLMGFGHRVYKNTDPRATAMKK
NCEEILAKLGHSDNPLLTVAKKLEEIALQDEFFIERKLFSNVDFYSGIILKAMGIPEDMFTAIFA
LARTSGWISQWIEMVNDPAQKIGRPRQLYTGATNRNF
>1KTBA
LENGLARTPPMGWLAWERFRCNVNCREDPRQCISEMLFMEMADRIAEDGWRELGYKYINIDDCWA
AKQRDAEGRLVPDPERFPRGIKALADYVHARGLKLGIYGDLGRLTCGGYPGTTLDRVEQDAQTFA
EWGVDMLKLDGCYSSGKEQAQGYPQMARALNATGRPIVYSCSWPAYQGGLPPKVNYTLLGEICNL
WRNYDDIQDSWDSVLSIVDWFFTNQDVLQPFAGPGHWNDPDMLIIGNFGLSYEQSRSQMALWTIM
AAPLLMSTDLRTISPSAKKILQNRLMIQINQDPLGIQGRRIIKEGSHIEVFLRPLSQAASALVFF
SRRTDMPFRYTTSLAKLGFPMGAAYEVQDVYSGKIISGLKTGDNFTVIINPSGVVMWYLCPKALL
IQQQAPGGPSRLPLL
>2WGVA
MGSITENTSWNKEFSAEAVNGVFVLCKSSSKSCATNDLARASKEYLPASTFKIPNAIIGLETGVI
KNEHQVFKWDGKPRAMKQWERDLTLRGAIQVSATPVFQQIAREVGEVRMQKYLKKFSYGNQNISG
GIDKFWLEGQLRISAVNQVEFLESLYLNKLSASKENQLIVKEALVTEAAPEYLVHSKTGFSGVGT
ESNPGVAWWVGWVEKETEVYFFAFNMDIDNESKLPLRKSIPTKIMESEGIIGG
>3GBXA
SNAMLKREMNIADYDAELWQAMEQEKVRQEEHIELIASENYTSPRVMQAQGSQLTNKYAEGYPGK
RYYGGCEYVDVVEQLAIDRAKELFGADYANVQPHSGSQANFAVYTALLQPGDTVLGMNLAQGGHL
THGSPVNFSGKLYNIVPYGIDESGKIDYDEMAKLAKEHKPKMIIGGFSAYSGVVDWAKMREIADS
IGAYLFVDMAHVAGLIAAGVYPNPVPHAHVVTTTTHKTLAGPRGGLILAKGGDEELYKKLNSAVF
PSAQGGPLMHVIAGKAVALKEAMEPEFKVYQQQVAKNAKAMVEVFLNRGYKVVSGGTENHLFLLD
LVDKNLTGKEADAALGRANITVNKNSVPNDPKSPFVTSGIRIGSPAVTRRGFKEAEVKELAGWMC
DVLDNINDEATIERVKAKVLDICARFPVYA
>1E6WA
AAAVRSVKGLVAVITGGASGLGLSTAKRLVGQGATAVLLDVPNSEGETEAKKLGGNCIFAPANVT
SEKEVQAALTLAKEKFGRIDVAVNCAGIAVAIKTYHEKKNQVHTLEDFQRVINVNLIGTFNVIRL
VAGVMGQNEPDQGGQRGVIINTASVAAFEGQVGQAAYSASKGGIVGMTLPIARDLAPIGIRVVTI
APGLFATPLLTTLPDKVRNFLASQVPFPSRLGDPAEYAHLVQMVIENPFLNGEVIRLDGAIRMQP

>1E6FA
MKSNEHDDCQVTNPSTGHLFDLSSLSGRAGFTAAYSEKGLVYMSICGENENCPPGVGACFGQTRI
SVGKANKRLRYVDQVLQLVYKDGSPCPSKSGLSYKSVISFVCRPEAGPTNRPMLISLDKQTCTLF
FSWHTPLACEQAT
>3A75B
TTHFTVADRWGNVVSYTTTIEQLFGTGIMVPDYGVILNNELTDFDAIPGGANEVQPNKRPLSSMT
PTILFKDDKPVLTVGSPGGATIISSVLQTILYHIEYGMELKAAVEEPRIYTNSMSSYRYEDGVPK
DVLSKLNGMGHKFGTSPVDIGNVQSISIDHENGTFKGVADSSRNGAAIGINLKRK
>3FIRA
MKKIITLFGACALAFSMANADVNLYGPGGPHTALKDIANKYSEKTGVKVNVNFGPQATWFEKAKK
DADILFGASDQSALAIASDFGKDFNVSKIKPLYFREAIILTQKGNPLKIKGLKDLANKKVRIVVP
EGAGESNTSGTGVWEDMIGRTQDIKTIQNFRNNIVAFVPNSGSARKLFAQDQADAWITWIDWSKS
NPDIGTAVAIEKDLVVYRTFNVIAKEGASKETQDFIAYLSSKEAKEIFKKYGWREH
>2IAPA
IPVIEPLFTKVTEDIPGAQGPVFDKNGDFYIVAPEVEVNGKPAGEILRIDLKTGKKTVICKPEVN
GYGGIPAGCQCDRDANQLFVADMRLGLLVVQTDGTFEEIAKKDSEGRRMQGCNDCAFDYEGNLWI
TAPAGEVAPADYTRSMQEKFGSIYCFTTDGQMIQVDTAFQFPNGIAVRHMNDGRPYQLIVAETPT
KKLWSYDIKGPAKIENKKVWGHIPGTHEGGADGMDFDEDNNLLVANWGSSHIEVFGPDGGQPKMR
IRCPFEKPSNLHFKPQTKTIFVTEHENNAVWKFEWQRNGKKQYCETLKFGIF
>1Q74A
MSETPRLLFVHAHPDDESLSNGATIAHYTSRGAQVHVVTCTLGEEGEVIGDRWAQLTADHADQLG
GYRIGELTAALRALGVSAPIYLGGAGRWRDSGMAGTDQRSQRRFVDADPRQTVGALVAIIRELRP
HVVVTYDPNGGYGHPDHVHTHTVTTAAVAAAGVGSGTADHPGDPWTVPKFYWTVLGLSALISGAR
ALVPDDLRPEWVLPRADEIAFGYSDDGIDAVVEADEQARAAKVAALAAHATQVVVGPTGRAAALS
NNLALPILADEHYVLAGGSAGARDERGWETDLLAGLGFTASGT
>2CWLA
MFLRIDRLQIELPMPKEQDPNAAAAVQALLGGRFGEMSTLMNYMYQSFNFRGKKALKPYYDLIAN
IATEELGHIELVAATINSLLAKNPGKDLEEGVDPESAPLGFAKDVRNAAHFIAGGANSLVMGAMG
EHWNGEYVFTSGNLILDLLHNFFLEVAARTHKLRVYEMTDNPVAREMIGYLLVRGGVHAAAYGKA
LESLTGVEMTKMLPIPKIDNSKIPEAKKYMDLGFHRNLYRFSPEDYRDLGLIWKGASPEDGTEVV
VVDGPPTGGPVFDAGHDAAEFAPEFHPGELYEIAKKLYEKAK
>3NK6A
SEFMTEPAIITNASDPAVQRIIDVTKHSRASIKTTLIEDTEPLMECIRAGVQFIEVYGSSGTPLD
PALLDLCRQREIPVRLIDVSIVNQLFKAERKAKVFGIARVPRPARLADIAERGGDVVVLDGVKIV
GNIGAIVRTSLALGAAGIVLVDSDLATIADRRLLRASRGYVFSLPVVLADREEAVSFLRDNDIAL
MVLDTDGDLGVKDLGDRADRMALVFGSEKGGPSGLFQEASAGTVSIPMLSSTESLNVSVSVGIAL
HERSARNFAVRRAAAQA
>1V3IA
ATSDSNMLLNYVPVYVMLPLGVVNVDNVFEDPDGLKEQLLQLRAAGVDGVMVDVWWGIIELKGPK
QYDWRAYRSLLQLVQECGLTLQAIMSFHQCGGNVGDIVNIPIPQWVLDIGESNHDIFYTNRSGTR
NKEYLTVGVDNEPIFHGRTAIEIYSDYMKSFRENMSDFLESGLIIDIEVGLGPAGELRYPSYPQS
QGWEFPGIGEFQCYDKYLKADFKAAVARAGHPEWELPDDAGKYNDVPESTGFFKSNGTYVTEKGK
FFLTWYSNKLLNHGDQILDEANKAFLGCKVKLAIKVSGIHWWYKVENHAAELTAGYYNLNDRDGY
RPIARMLSRHHAILNFTCLEMRDSEQPSDAKSGPQELVQQVLSGGWREDIRVAGQNALPRYDATA

YNQIILNARPQGVNNNGPPKLSMFGVTYLRLSDDLLQKSNFNIFKKFVLKMHADQDYCANPQKYN
HAITPLKPSAPKIPIEVLLEATKPTLPFPWLPETDMKVDG
>2W87A
ALLLQEAQAGFCRVDGTIDNNHTGFTGSGFANTNNAQGAAVVWAIDATSSGRRTLTIRYANGGTA
NRNGSLVINGGSNGNYTVSLPTTGAWTTWQTATIDVDLVQGNNIVQLSATTAEGLPNIDSLSVVG
GTVRAGNCG
>2W8TA
TEAAAQPHALPADAPDIAPERDLLSKFDGLIAERQKLLDSGVTDPFAIVMEQVKSPTEAVIRGKD
TILLGTYNYMGMTFDPDVIAAGKEALEKFGSGTCGSRMLNGTFHDHMEVEQALRDFYGTTGAIVF
STGYMANLGIISTLAGKGEYVILDADSHASIYDGCQQGNAEIVRFRHNSVEDLDKRLGRLPKEPA
KLVVLEGVYSMLGDIAPLKEMVAVAKKHGAMVLVDEAHSMGFFGPNGRGVYEAQGLEGQIDFVVG
TFSKSVGTVGGFVVSNHPKFEAVRLACRPYIFTASLPPSVVATATTSIRKLMTAHEKRERLWSNA
RALHGGLKAMGFRLGTETCDSAIVAVMLEDQEQAAMMWQALLDGGLYVNMARPPATPAGTFLLRC
SICAEHTPAQIQTVLGMFQAAGRAVGVIGLEHHHHHH
>1Y0BA
SNAMEALKRKIEEEGVVLSDQVLKVDSFLNHQIDPLLMQRIGDEFASRFAKDGITKIVTIESSGI
APAVMTGLKLGVPVVFARKHKSLTLTDNLLTASVYSFTKQTESQIAVSGTHLSDQDHVLIIDDFL
ANGQAAHGLVSIVKQAGASIAGIGIVIEKSFQPGRDELVKLGYRVESLARIQSLEEGKVSFVQEV
HS
>3K3KA
MGSSHHHHHHSSGLVPRGSHMPSELTPEERSELKNSIAEFHTYQLDPGSCSSLHAQRIHAPPELV
WSIVRRFDKPQTYKHFIKSCSVEQNFEMRVGCTRDVIVISGLPANTSTERLDILDDERRVTGFSI
IGGEHRLTNYKSVTTVHRFEKENRIWTVVLESYVVDMPEGNSEDDTRMFADTVVKLNLQKLATVA
EAMARNSGDGSGSQVT
>3LIDA
MEKYQALLANNVENTAKEALHQLAYTGREYNNIQDQIETISDLLGHSQSLYDYLREPSKANLTIL
ENMWSSVARNQKLYKQIRFLDTSGTEKVRIKYDFKTSIAGPSLILRDKSAREYFKYAQSLDNEQI
SAWGIELERDKGELVYPLSPSLRILMPISVNDVRQGYLVLNVDIEYLSSLLNYSPVRDFHIELVK
HKGFYIASPDESRLYGDIIPERSQFNFSNMYPDIWPRVVSEQAGYSYSGEHLIAFSSIKFVSNEP
LHLIIDLSNEQLSKRATRDINDLIQESLEHHHHHH
>1PGTA
MPPYTVVYFPVRGRCAALRMLLADQGQSWKEEVVTVETWQEGSLKASCLYGQLPKFQDGDLTLYQ
SNTILRHLGRTLGLYGKDQQEAALVDMVNDGVEDLRCKYVSLIYTNYEAGKDDYVKALPGQLKPF
ETLLSQNQGGKTFIVGDQISFADYNLLDLLLIHEVLAPGCLDAFPLLSAYVGRLSARPKLKAFLA
SPEYVNLPINGNGKQ
>1ZLHB
NECVSKGFGCLPQSDCPQEARLSYGGCSTVCCDLSKLTGCKGKGGECNPLDRQCKELQAESASCG
KGQKCCVWLH
>3CIFA
MGSSHHHHHHSSGLVPRGSHMTATLGINGFGRIGRLVLRACMERNDITVVAINDPFMDVEYMAYL
LKYDSVHGNFNGTVEVSGKDLCINGKVVKVFQAKDPAEIPWGASGAQIVCESTGVFTTEEKASLH
LKGGAKKVIISAPPKDNVPMYVMGVNNTEYDPSKFNVISNASSTTNCLAPLAKIINDKFGIVEGL
MTTVHSLTANQLTVDGPSKGGKDWRAGRCAGNNIIPASTGAAKAVGKVIPALNGKLTGMAIRVPT
PDVSVVDLTCKLAKPASIEEIYQAVKEASNGPMKGIMGYTSDDVVSTDFIGCKYSSILDKNACIA

LNDSFVKLISWYDNESGYSNRLVDLAVYVASRGL
>2JINA
SMRVDYLVTEEEINLTRGPSGLGFNIVGGTDQQYVSNDSGIYVSRIKENGAAALDGRLQEGDKIL
SVNGQDLKNLLHQDAVDLFRNAGYAVSLRVQHRESSI
>2RK6A
GSHMASKRALVILAKGAEEMETVIPVDVMRRAGIKVTVAGLAGKDPVQCSRDVVICPDASLEDAK
KEGPYDVVVLPGGNLGAQNLSESAAVKEILKEQENRKGLIAAICAGPTALLAHEIGFGSKVTTHP
LAKDKMMNGGHYTYSENRVEKDGLILTSRGPGTSFKFALAIVEALNGKEVAAQVKAPLVLKD
>2GAIA
MAKKVKKYIVVESPAKAKTIKSILGNEYEVFASMGHIIDLPKSKFGVDLEKDFEPEFAVIKGKEK
VVEKLKDLAKKGELLIASDMDREGEAIAWHIARVTNTLGRKNRIVFSEITPRVIREAVKNPREID
MKKVRAQLARRILDRIVGYSLSPVLWRNFKSNLSAGRVQSATLKLVCDREREILRFVPKKYHRIT
VNFDGLTAEIDVKEKKFFDAETLKEIQSIDELVVEEKKVSVKKFAPPEPFKTSTLQQEAYSKLGF
SVSKTMMIAQQLYEGVETKDGHIAFITYMRTDSTRVSDYAKEEARNLITEVFGEEYVGSKRERRK
SNAKIQDAHEAIRPTNVFMTPEEAGKYLNSDQKKLYELIWKRFLASQMKPSQYEETRFVLRTKDG
KYRFKGTVLKKIFDGYEKVWKTERNTGEFPFEEGESVKPVVVKIEEQETKPKPRYTEGSLVKEME
RLGIGRPSTYASTIKLLLNRGYIKKIRGYLYPTIVGSVVMDYLEKKYSDVVSVSFTAEMEKDLDE
VEQGKKTDKIVLREFYESFSSVFDRNDRIVVDFPTNQKCSCGKEMRLSFGKYGFYLKCECGKTRS
VKNDEIAVIDDGKIFLGRKDSESGSPDGRSVEGKGNLSEKRRKGKKGS
>2GAKA
PEFFSVRHLELAGDDPYSNVNCTKILQGDPEEIQKVKLEILTVQFKKRPRWTPHDYINMTRDCAS
FIRTRKYIVEPLTKEEVGFPIAYSIVVHHKIEMLDRLLRAIYMPQNFYCIHVDRKAEESFLAAVQ
GIASCFDNVFVASQLESVVYASWTRVKADLNCMKDLYRMNANWKYLINLCGMDFPIKTNLEIVRK
LKCSTGENNLETEKMPPNKEERWKKRYAVVDGKLTNTGIVKAPPPLKTPLFSGSAYFVVTREYVG
YVLENENIQKLMEWAQDTYSPDEFLWATIQRIPEVPGSFPSSNKYDLSDMNAIARFVKWQYFEGD
VSNGAPYPPCSGVHVRSVCVFGAGDLSWMLRQHHLFANKFDMDVDPFAIQCLDEHLRRKALENLE
H
>2RLCA
CTGLALETKDGLHLFGRNMDIEYSFNQSIIFIPRNFKCVNKSNKKELTTKYAVLGMGTIFDDYPT
FADGMNEKGLGCAGLNFPVYVSYSKEDIEGKTNIPVYNFLLWVLANFSSVEEVKEALKNANIVDI
PISENIPNTTLHWMISDITGKSIVVEQTKEKLNVFDNNIGVLTNSPTFDWHVANLNQYVGLRYNQ
VPEFKLGDQSLTALGQGTGLVGLPGDFTPASRFIRVAFLRDAMIKNDKDSIDLIEFFHILNNVAM
VRGSTRTVEEKSDLTQYTSCMCLEKGIYYYNTYENNQINAIDMNKENLDGNEIKTYKYNKTLSIN
HVN
>3DR3A
SNAMLNTLIVGASGYAGAELVTYVNRHPHMNITALTVSAQSNDAGKLISDLHPQLKGIVELPLQP
MSDISEFSPGVDVVFLATAHEVSHDLAPQFLEAGCVVFDLSGAFRVNDATFYEKYYGFTHQYPEL
LEQAAYGLAEWCGNKLKEANLIAVPGCYPTAAQLALKPLIDADLLDLNQWPVINATSGVSGAGRK
AAISNSFCEVSLQPYGVFTHRHQPEIATHLGADVIFTPHLGNFPRGILETITCRLKSGVTQAQVA
QALQQAYAHKPLVRLYDKGVPALKNVVGLPFCDIGFAVQGEHLIIVATEDNLLKGAAAQAVQCAN
IRFGYAETQSLI
>2CN3A
ISSQAVTSVPYKWDNVVIGGGGGFMPGIVFNETEKDLIYARAAIGGAYRWDPSTETWIPLLDHFQ
MDEYSYYGVESIATDPVDPNRVYIVAGMYTNDWLPNMGAILRSTDRGETWEKTILPFKMGGNMPG

RSMGERLAIDPNDNRILYLGTRCGNGLWRSTDYGVTWSKVESFPNPGTYIYDPNFDYTKDIIGVV
WVVFDKSSSTPGNPTKTIYVGVADKNESIYRSTDGGVTWKAVPGQPKGLLPHHGVLASNGMLYIT
YGDTCGPYDGNGKGQVWKFNTRTGEWIDITPIPYSSSDNRFCFAGLAVDRQNPDIIMVTSMNAWW
PDEYIFRSTDGGATWKNIWEWGMYPERILHYEIDISAAPWLDWGTEKQLPEINPKLGWMIGDIEI
DPFNSDRMMYVTGATIYGCDNLTDWDRGGKVKIEVKATGIEECAVLDLVSPPEGAPLVSAVGDLV
GFVHDDLKVGPKKMHVPSYSSGTGIDYAELVPNFMALVAKADLYDVKKISFSYDGGRNWFQPPNE
APNSVGGGSVAVAADAKSVIWTPENASPAVTTDNGNSWKVCTNLGMGAVVASDRVNGKKFYAFYN
GKFYISTDGGLTFTDTKAPQLPKSVNKIKAVPGKEGHVWLAAREGGLWRSTDGGYTFEKLSNVDT
AHVVGFGKAAPGQDYMAIYITGKIDNVLGFFRSDDAGKTWVRINDDEHGYGAVDTAITGDPRVYG
RVYIATNGRGIVYGEPASDEPV

>1XA3A

MHHHHHHGSTSLYKKAGSETLYIQGDHLPMPKFGPLAGLRVVFSGIEIAGPFAGQMFAEWGAEVI
WIENVAWADTIRVQPNYPQLSRRNLHALSLNIFKDEGREAFLKLMETTDIFIEASKGPAFARRGI
TDEVLWQHNPKLVIAHLSGFGQYGTEEYTNLPAYNTIAQAFSGYLIQNGDVDQPMPAFPYTADYF
SGLTATTAALAALHKVRETGKGESIDIAMYEVMLRMGQYFMMDYFNGGEMCPRMSKGKDPYYAGC
GLYKCADGYIVMELVGITQIEECFKDIGLAHLLGTPEIPEGTQLIHRIECPYGPLVEEKLDAWLA
THTIAEVKERFAELNIACAKVLTVPELESNPQYVARESITQWQTMDGRTCKGPNIMPKFKNNPGQ
IWRGMPSHGMDTAAILKNIGYSENDIQELVSKGLAKVEDSTHHHHHH

>1GPIA

QQAGTNTAENHPQLQSQQCTTSGGCKPLSTKVVLDSNWRWVHSTSGYTNCYTGNEWDTSLCPDGK
TCAANCALDGADYSGTYGITSTGTALTLKFVTGSNVGSRVYLMADDTHYQLLKLLNQEFTFDVDM
SNLPCGLNGALYLSAMDADGGMSKYPGNKAGAKYGTGYCDSQCPKDIKFINGEANVGNWTETGSN
TGTGSYGTCCSEMDIWEANNDAAAFTPHPCTTTGQTRCSGDDCARNTGLCDGDGCDFNSFRMGDK
TFLGKGMTVDTSKPFTVVTQFLTNDNTSTGTLSEIRRIYIQNGKVIQNSVANIPGVDPVNSITDN
FCAQQKTAFGDTNWFAQKGGLKQMGEALGNGMVLALSIWDDHAANMLWLDSDYPTDKDPSAPGVA
RGTCATTSGVPSDVESQVPNSQVVFSNIKFGDIGSTFSGTS

>1CMNA

ASGAPSFPFSRASGPEPPAEFAKLRATNPVSQVKLFDGSLAWLVTKHKDVCFVATSEKLSKVRTR
QGFPELSASGKQAAKAKPTFVDMDPPEHMHQRSMVEPTFTPEAVKNLQPYIQRTVDDLLEQMKQK
GCANGPVDLVKEFALPVPSYIIYTLLGVPFNDLEYLTQQNAIRTNGSSTAREASAANQELLDYLA
ILVEQRLVEPKDDIISKLCTEQVKPGNIDKSDAVQIAFLLLVAGNATMVNMIALGVATLAQHPDQ
LAQLKANPSLAPQFVEELCRYHTAVALAIKRTAKEDVMIGDKLVRANEGIIASNQSANRDEEVFE
NPDEFNMNRKWPPQDPLGFGFGDHRCIAEHLAKAELTTVFSTLYQKFPDLKVAVPLGKINYTPLN
RDVGIVDLPVIF

>1LRIA

TACTASQQTAAYKTLVSILSDASFNQCSTDSGYSMLTAKALPTTAQYKLMCASTACNTMIKKIVT
LNPPNCDLTVPTSGLVLNVYSYANGFSNKCSSL

>2QECA

GMSPTVLPATQADFPKIVDVLVEAFANDPTFLRWIPQPDPGSAKLRALFELQIEKQYAVAGNIDV
ARDSEGEIVGVALWDRPDGNHSAKDQAAMLPRLVSIFGIKAAQVAWTDLSSARFHPKFPHWYLYT
VATSSSARGTGVGSALLNHGIARAGDEAIYLEATSTRAAQLYNRLGFVPLGYIPSDDDGTPELAM
WKPPAMPTV

>1VAJA

MVFKIKDEWGEFLVRLARRAIEEYLKTGKEIEPPKDTPPELWEKMGVFVTLNRYNVPPQTALRGC

IGFPTPIYPLVEATIKAAIYSAVDDPRFPPVKLEEMDNLVVEVSVLTPPELIEGPPEERPRKIKV
GRDGLIVEKGIYSGLLLPQVPVEWGWDEEEFLAETCWKAGLPPDCWLDEDTKVYKFTAEIFEEEY
PRGPIKRKPLVLEHHHHHH
>1D7EA
VAFGSEDIENTLAKMDDGQLDGLAFGAIQLDGDGNILQYNAAEGDITGRDPKQVIGKNFFKDVAP
CTDSPEFYGKFKEGVASGNLNTMFEYTFDYQMTPTKVKVHMKKALSGDSYWVFVKRV
>1YNVX
DVSGTVCLSALPPEATDTLNLIASDGPFPYSQDGVVFQNRESVLPTQSYGYYHEYTVITPGARTR
GTRRIITGEATQEDYYTGDHYATFSLIDKTC
>1XH9A
GNAAAAKKGSEQESVKEFLAKAKEDFLKKWENPAQNTAHLDQFERIKTLGTGSFGRVMLVKHMET
GNHYAMKILDKQKVVKLKEIEHTLNEKRILQAVNFPFLVKLEFSFKDNSNLYMVMEYAPGGEMFS
HLRRIGRFSEPHARFYAAQIVLTFEYLHSLDLIYRDLKPENLMIDQQGYIKVTDFGLAKRVKGRT
WTLCGTPEYLAPEIILSKGYNKAVDWWALGVLIYEMAAGYPPFFADQPIQIYEKIVSGKVRFPSH
FSSDLKDLLRNLLQVDLTKRFGNLKNGVNDIKNHKWFATTDWIAIYQRKVEAPFIPKFKGPGDTS
NFDDYEEEEIRVSINEKCGKEFSEF
>3OIRA
SNADGLEGMDDPDATSKKVVPLGVEIYEINGPFFFGVADRLKGVLDVIEETPKVFILRMRRVPVI
DATGMHALWEFQESCEKRGTILLLSGVSDRLYGALNRFGFIEALGEERVFDHIDKALAYAKLLVE
TAEER
>2V4CA
ADYDLKFGMNAGTSSNEYKAAEMFAKEVKEKSQGKIEISLYPSSQLGDDRAMLKQLKDGSLDFTF
AESARFQLFYPEAAVFALPYVISNYNVAQKALFDTEFGKDLIKKMDKDLGVTLLSQAYNGTRQTT
SNRAINSIADMKGLKLRVPNAATNLAYAKYVGASPTPMAFSEVYLALQTNAVDGQENPLAAVQAQ
KFYEVQKFLAMTNHILNDQLYLVSNETYKELPEDLQKVVKDAAENAAKYHTKLFVDGEKDLVTFF
EKQGVKITHPDLVPFKESMKPYYAEFVKQTGQKGESALKQIEAINPHHH
>1JNRA
MVYYPKKYELYKADEVPTEVVETDILIIGGGFSGCGAAYEAAYWAKLGGLKVTLVEKAAVERSGA
VAQGLSAINTYIDLTGRSERQNTLEDYVRYVTLDMMGLAREDLVADYARHVDGTVHLFEKWGLPI
WKTPDGKYVREGQWQIMIHGESYKPIIAEAAKMAVGEENIYERVFIFELLKDNNDPNAVAGAVGF
SVREPKFYVFKAKAVILATGGATLLFRPRSTGEAAGRTWYAIFDTGSGYYMGLKAGAMLTQFEHR
FIPFRFKDGYGPVGAWFLFFKCKAKNAYGEEYIKTRAAELEKYKPYGAAQPIPTPLRNHQVMLEI
MDGNQPIYMHTEEALAELAGGDKKKLKHIYEEAFEDFLDMTVSQALLWACQNIDPQEQPSEAAPA
EPYIMGSHSGEAGFWVCGPEDLMPEEYAKLFPLKYNRMTTVKGLFAIGDCAGANPHKFSSGSFTE
GRIAAKAAVRFILEQKPNPEIDDAVVEELKKKAYAPMERFMQYKDLSTADDVNPEYILPWQGLVR
LQKIMDEYAAGIATIYKTNEKMLQRALELLAFLKEDLEKLAARDLHELMRAWELVHRVWTAEAHV
RHMLFRKETRWPGYYYRTDYPELNDEEWKCFVCSKYDAEKDEWTFEKVPYVQVIEWSF
>2QM0A
SNAMNTTVEKQQIITSNTEQWKMYSKLEGKEYQIHISKPKQPAPDSGYPVIYVLDGNAFFQTFHE
AVKIQSVRAEKTGVSPAIIVGVGYPIEGAFSGEERCYDFTPSVISKDAPLKPDGKPWPKTGGAHN
FFTFIEEELKPQIEKNFEIDKGKQTLFGHXLGGLFALHILFTNLNAFQNYFISSPSIWWNNKSVL
EKEENLIIELNNAKFETGVFLTVGSLEREHMVVGANELSERLLQVNHDKLKFKFYEAEGENHASV
VPTSLSKGLRFISYV
>2QM6A

MGSSHHHHHHSSGLVPRGSHMASAASYPPIKNTKVGLALSSHPLASEIGQKVLEEGGNAIDAAVA
IGFALAVVHPAAGNIGGGGFAVIHLANGENVALDFREKAPLKATKNMFLDKQGNVVPKLSEDGYL
AAGVPGTVAGMEAMLKKYGTKKLSQLIDPAIKLAENGYAISQRQAETLKEARERFLKYSSSKKYF
FKKGHLDYQEGDLFVQKDLAKTLNQIKTLGAKGFYQGQVAELIEKDMKKNGGIITKEDLASYNVK
WRKPVVGSYRGYKIISMSPPSSGGTHLIQILNVMENADLSALGYGASKNIHIAAEAMRQAYADRS
VYMGDADFVSVPVDKLINKAYAKKIFDTIQPDTVTPSSQIKPGMGQLHEGSN

>3GHJA

MGSSHHHHHHSSGRENLYFQGVPMNIKGLFEVAVKVKNLEKSSQFYTEILGFEAGLLDSARRWNF
LWVSGRAGMVVLQEEKENWQQQHFSFRVEKSEIEPLKKALESKGVSVHGPVNQEWMQAVSLYFAD
PNGHALEFTAL

>2Z58B

NTIRVIVSVDKAKFNPHEVLGIGGHIVYQFKLIPAVVVDVPANAVGKLKKMPWVEKVEFDHQAVL
L

>1CUHA

LPTSNPAQELEARQLGRTTRDDLINGNSASCADVIFIYARGSTETGNLGTLGPSIASNLESAFGK
DGVWIQGVGGAYRATLGDNALPRGTSSAAIREMLGLFQQANTKCPDATLIAGGYSQGAALAAASI
EDLDSAIRDKIAGTVLFGYTKNLQNRGRIPNYPADRTKVFCNTGDLVCTGSLIVAAPHLAYGPDA
EGPAPEFLIEKVRAVRGSA

>1OJQA

AETKNFTDLVEATKWGNSLIKSAKYSSKDKMAIYNYTKNSSPINTPLRSANGDVNKLSENIQEQV
RQLDSTISKSVTPDSVYVYRLLNLDYLSSITGFTREDLHMLQQTNNGQYNEALVSKLNNLMNSRI
YRENGYSSTQLVSGAALAGRPIELKLELPKGTKAAYIDSKELTAYPGQQEVLLPRGTEYAVGSVK
LSDNKRKIIITAVVFKK

>1NIJA

MNPIAVTLLTGFLGAGKTTLLRHILNEQHGYKIAVIENEFGEVSVDDQLIGDRATQIKTLTNGCI
CCSRSNELEDALLDLLDNLDKGNIQFDRLVIECTGMADPGPIIQTFFSHEVLCQRYLLDGVIALV
DAVHADEQMNQFTIAQSQVGYADRILLTKTDVAGEAEKLHERLARINARAPVYTVTHGDIDLGLL
FNTNGFMLEENVVSTKPRFHFIADKQNDISSIVVELDYPVDISEVSRVMENLLLESADKLLRYKG
MLWIDGEPNRLLFQGVQRLYSADWDRPWGDEKPHSTMVFIGIQLPEEEIRAAFAGLRK

>2O2GA

GMDRTLTHQPQEYAVSVSVGEVKLKGNLVIPNGATGIVLFAHGSGSSRYSPRNRYVAEVLQQAGL
ATLLIDLLTQEEEEIDLRTRHLRFDIGLLASRLVGATDWLTHNPDTQHLKVGYFGASTGGGAALV
AAAERPETVQAVVSRGGRPDLAPSALPHVKAPTLLIVGGYDLPVIAMNEDALEQLQTSKRLVIIP
RASHLFEEPGALTAVAQLASEWFMHYLR

>1O2DA

MGSDKIHHHHHHVWEFYMPTDVFFGEKILEKRGNIIDLLGKRALVVTGKSSSKKNGSLDDLKKLL
DETEISYEIFDEVEENPSFDNVMKAVERYRNDSFDFVVGLGGGSPMDFAKAVAVLLKEKDLSVED
LYDREKVKHWLPVVEIPTTAGTGSEVTPYSILTDPEGNKRGCTLMFPVYAFLDPRYTYSMSDELT
LSTGVDALSHAVEGYLSRKSTPPSDALAIEAMKIIHRNLPKAIEGNREARKKMFVASCLAGMVIA
QTGTTLAHALGYPLTTEKGIKHGKATGMVLPFVMEVMKEEIPEKVDTVNHIFGGSLLKFLKELGL
YEKVAVSSEELEKWVEKGSRAKHLKNTPGTFTPEKIRNIYREALGV

>3MR1A

GPGSMIKIHTEKDFIKMRAAGKLAAETLDFITDHVKPNVTTNSLNDLCHNFITSHNAIPAPLNYK
GFPKSICTSINHVVCHGIPNDKPLKNGDIVNIDVTVILDGWYGDTSRMYYVGDVAIKPKRLIQVT

YDAMMKGIEVVRPGAKLGDIGYAIQSYAEKHNYSVVRDYTGHGIGRVFHDKPSILNYGRNGTGLT
LKEGMFFTVEPMINAGNYDTILSKLDGWTVTTRDKSLSAQFEHTIGVTKDGFEIFTL
>3NJCA
MGHHHHHHSHMKSKFEASIDNLKEIEMNAYAYELIREIVLPDMLGQDYSSMMYWAGKHLARKFPL
ESWEEFPAFFEEAGWGTLTNVSAKKQELEFELEGPIISNRLKHQKEPCFQLEAGFIAEQIQLMND
QIAESYEQVKKRADKVVLTVKWDMKDPV
>3R0NA
MQDVRVQVLPEVRGQLGGTVELPCHLLPPVPGLYISLVTWQRPDAPANHQNVAAFHPKMGPSFPS
PKPGSERLSFVSAKQSTGQDTEAELQDATLALHGLTVEDEGNYTCEFATFPKGSVRGMTWLRV
>1FPZA
MKPPSSIQTSEFDSSDEEPIEDEQTPIHISWLSLSRVNCSQFLGLCALPGCKFKDVRRNVQKDTE
ELKSCGIQDIFVFCTRGELSKYRVPNLLDLYQQCGIITHHHPIADGGTPDIASCCEIMEELTTCL
KNYRKTLIHSYGGLGRSCLVAACLLLYLSDTISPEQAIDSLRDLRGSGAIQTIKQYNYLHEFRDK
LAAHLSSRDSQSRSVSR
>3LX4A
MGSSHHHHHHSQDPNSAAPAAEAPLSHVQQALAELAKPKDDPTRKHVCVQVAPAVRVAIAETLGL
APGATTPKQLAEGLRRLGFDEVFDTLFGADLTIMEEGSELLHRLTEHLEAHPHSDEPLPMFTSCC
PGWIAMLEKSYPDLIPYVSSCKSPQMMLAAMVKSYLAEKKGIAPKDMVMVSIMPCTRKQSEADRD
WFCVDADPTLRQLDHVITTVELGNIFKERGINLAELPEGEWDNPMGVGSGAGVLFGTTGGVMEAA
LRTAYELFTGTPLPRLSLSEVRGMDGIKETNITMVPAPGSKFEELLKHRAAARAEAAAHGTPGPL
AWDGGAGFTSEDGRGGITLRVAVANGLGNAKKLITKMQAGEAKYDFVEIMACPAGCVGGGGQPRS
TDKAITQKRQAALYNLDEKSTLRRSHENPSIRELYDTYLGEPLGHKAHELLHTHYVAGGVEEKDE
KK
>1SCTB
SKVAELANAVVSNADQKDLLRMSWGVLSVDMEGTGLMLMANLFKTSPSAKGKFARLGDVSAGKDN
SKLRGHSITLMYALQNFVDALDDVERLKCVVEKFAVNHINRQISADEFGEIVGPLRQTLKARMGN
YFDEDTVAAWASLVAVVQASL
>3M1MA
SSNFSSERIRYAKWFLEHGFNIIPIDPESKKPVLKEWQKYSHEMPSDEEKQRFLKMIEEGYNYAI
PGGQKGLVILDFESKEKLKAWIGESALEELCRKTLCTNTVHGGIHIYVLSNDIPPHKINPLFEEN
GKGIIDLQSYNSYVLGLGSCVNHLHCTTDKCPWKEQNYTTCYTLYNELKEISKVDLKSLLRFLAE
KGKRLGITLSKTAKEWLEGKKEEEDTVVEFEELRKELVKRDSGKPVEKIKEEICTKSPPKLIKEI
ICENKTYADVNIDRSRGDWHVILYLMKHGVTDPDKILELLPRDSKAKENEKWNTQKYFVITLSKA
WSVVKKYLEA
>3M12A
STHFDVIVVGAGSMGMAAGYQLAKQGVKTLLVDAFDPPHTNGSHHGDTRIIRHAYGEGREYVPLA
LRSQELWYELEKETHHKIFTKTGVLVFGPKGESAFVAETMEAAKEHSLTVDLLEGDEINKRWPGI
TVPENYNAIFEPNSGVLFSENCIRAYRELAEARGAKVLTHTRVEDFDISPDSVKIETANGSYTAD
KLIVSMGAWNSKLLSKLNLDIPLQPYRQVVGFFESDESKYSNDIDFPGFMVEVPNGIYYGFPSFG
GCGLRLGYHTFGQKIDPDTINREFGVYPEDESNLRAFLEEYMPGANGELKRGAVCMYTKTLDEHF
IIDLHPEHSNVVIAAGFSGHGFKFSSGVGEVLSQLALTGKTEHDISIFSINRPALKESLQKTTI
>3BDZA
TSLFTTADHYHTPLGPDGTPHAFFEALRDEAETTPIGWSEAYGGHWVVAGYKEIQAVIQNTKAFS
NKGVTFPRYETGEFELMMAGQDDPVHKKYRQLVAKPFSPEATDLFTEQLRQSTNDLIDARIELGE

GDAATWLANEIPARLTAILLGLPPEDGDTYRRWVWAITHVENPEEGAEIFAELVAHARTLIAERR
TNPGNDIMSRVIMSKIDGESLSEDDLIGFFTILLLGGIDATARFLSSVFWRLAWDIELRRRLIAH
PELIPNAVDELLRFYGPAMVGRLVTQEVTVGDITMKPGQTAMLWFPIASRDRSAFDSPDNIVIER
TPNRHLSLGHGIHRCLGAHLIRVEARVAITEFLKRIPEFSLDPNKECEWLMGQVAGMLHVPIIFP
KGKRLSE
>1FY3A
IVGGRKARPRQFPFLASIQNQGRHFCGGALIHARFVMTAASCFQSQNPGVSTVVLGAYDLRRRER
QSRQTFSISSMSENGYDPQQNLNDLMLLQLDREANLTSSVTILPLPLQNATVEAGTRCQVAGWGS
QRSGGRLSRFPRFVNVTVTPEDQCRPNNVCTGVLTRRGGICNGDQGTPLVCEGLAHGVASFSLGP
CGRGPDFFTRVALFRDWIDGVLNNPGPGPA
>2VR3A
MRGSHHHHHHGSGTDITNQLTNVTVGIDSGTTVYPHQAGYVKLNYGFSVPNSAVKGDTFKITVPK
ELNLNGVTSTAKVPPIMAGDQVLANGVIDSDGNVIYTFTDYVNTKCDVKATLTMPAYIDPENVKK
TGNVTLATGIGSTTANKTVLVDYEKYGKFYNLSIKGTIDQIDKTNNTYRQTIYVNPSGDNVIAPV
LTGNLKPNTDSNALIDQQNTSIKVYKVDNAADLSESYFVNPENFEDVTNSVNITFPNPNQYKVEF
NTPDDQITTPYIVVVNGHIDPNSKGDLALRSTLYGYNSNIIWRSMSWDNEVAFNNGSGSGDGIDC
PVVP
>1R8ME
LEANEGSKTLQRNRKMAMGRKKFNMDPKKGIQFLVENELLQNTPEEIARFLYKGEGLNKTAIGDY
LGEREELNLAVLHAFVDLHEFTDLNLVQALRQFLWSFRLPGEAQKIDRMMEAFAQRYCLCNPGVF
QSTDTCYVLSYSVIMLNTDLHNPNVRDKMGLERFVAMNRGINEGGDLPEELLRNLYDSIRNEPFK
IPEDDGND
>1BYPA
AEVLLGSSDGGLAFVPSDLSIASGEKITFKNNAGFPHNDLFDKKEVPAGVDVTKISMPEEDLLNA
PGEEYSVTLTEKGTYKFYCAPHAGAGMVGKVTVN
>3D0KA
SNAMKPADLTNADRIALELGHAGRNAIPYLDDDRNADRPFTLNTYRPYGYTPDRPVVVVQHGVLR
NGADYRDFWIPAADRHKLLIVAPTFSDEIWPGVESYNNGRAFTAAGNPRHVDGWTYALVARVLAN
IRAAEIADCEQVYLFGHSAGGQFVHRLMSSQPHAPFHAVTAANPGWYTLPTFEHRFPEGLDGVGL
TEDHLARLLAYPMTILAGDQDIATDDPNLPSEPAALRQGPHRYARARHYYEAGQRAAAQRGLPFG
WQLQVVPGIGHDGQAMSQVCASLWFDGRMPDAAELARLAGSQSA
>1ZHVA
APRIKLKILNGSYGIARLSASEAIPAWADGGGFVSITRTDDELSIVCLIDRIPQDVRVDPGWSCF
KFQGPFAFDETGIVLSVISPLSTNGIGIFVVSTFDGDHLLVRSNDLEKTADLLANAGHSLLLEHH
HHHH
>3H3JA
MNKFKGNKVVLIGNGAVGSSYAFSLVNQSIVDELVIIDLDTEKVRGDVMDLKHATPYSPTTVRVK
AGEYSDCHDADLVVICAGARQKPGETRLDLVSKNLKIFKSIVGEVMASKFDGIFLVATNPVDILA
YATWKFSGLPKERVIGSGTILDSARFRLLLSEAFDVAPRSVDAQIIGEHGDTELPVWSHANIAGQ
PLKTLLEQRPEGKAQIEQIFVQTRDAAYDIIQAKGATYYGVAMGLARITEAIFRNEDAVLTVSAL
LEGEYEEEDVYIGVPAVINRNGIRNVVEIPLNDEEQSKFAHSAKTLKDIMAEAEELK
>3H36A
SNAVELLQVDADLQAEIVGKYNADLQKAVQIEEKKASEIATEAVKEHVTAEYEERYAEHEEHDRI
MRDVAEILEQMEHAEVRRLITEDKVRPD

>1Z3EB
MEKEKVLEMTIEELDLSVRSYNCLKRAGINTVQELANKTEEDMMKVRNLGRKSLEEVKAKLEELG
LGLRKDDG
>3KFFA
EEATSKGQNLNVEKINGEWFSILLASDKREKIEEHGSMRVFVEHIHVLENSLAFKFHTVIDGECS
EIFLVADKTEKAGEYSVMYDGFNTFTILKTDYDNYIMFHLINEKDGKTFQLMELYGRKADLNSDI
KEKFVKLCEEHGIIKENIIDLTKTNRCLKARE
>2RB8A
MRLDAPSQIEVKDVTDTTALITWMPPSQPVDGFELTYGIKDVPGDRTTIDLTEDENQYSIGNLKP
DTEYEVSLISRRGDMSSNPAKETFTTGLAAALEHHHHHH
>2RBDA
GMGILSGNPQDEPLHYGEVFSTWTYLSTNNGLINGYRSFINHTGDEDLKNLIDEAIQAMQDENHQ
LEELLRSNGVGLPPAPPDRPAARLDDIPVGARFNDPEISATISMDVAKGLVTCSQIIGQSIREDV
ALMFSQFHMAKVQFGGKMLKLNKNKGWLIPPPLHSDRPIKE
>2W1VA
MSTFRLALIQLQVSSIKSDNLTRACSLVREAAKQGANIVSLPECFNSPYGTTYFPDYAEKIPGES
TQKLSEVAKESSIYLIGGSIPEEDAGKLYNTCSVFGPDGSLLVKHRKIHLFDIDVPGKITFQESK
TLSPGDSFSTFDTPYCKVGLGICYDMRFAELAQIYAQRGCQLLVYPGAFNLTTGPAHWELLQRAR
AVDNQVYVATASPARDDKASYVAWGHSTVVDPWGQVLTKAGTEETILYSDIDLKKLAEIRQQIPI
LKQKRADLYTVESKKP
>3OBIA
GMPHHQYVLTLSCPDRAGIVSAVSTFLFENGQNILDAQQYNDTESGHFFMRVVFNAAAKVIPLAS
LRTGFGVIAAKFTMGWHMRDRETRRKVMLLVSQSDHCLADILYRWRVGDLHMIPTAIVSNHPRET
FSGFDFGDIPFYHFPVNKDTRRQQEAAITALIAQTHTDLVVLARYMQILSDEMSARLAGRCINIH
HSFLPGFKGAKPYHQAFDRGVKLIGATAHYVTSALDEGPIIDQDVERISHRDTPADLVRKGRDIE
RRVLSRALHYHLDDRVILNGRKTVVFTD
>2BZUA
RIAISNSNRTRSVPSLTTIWSISPTPNCSIYETQDANLFLCLTKNGAHVLGTITIKGLKGALREM
HDNALSLKLPFDNQGNLLNCALESSTWRYQETNAVASNALTFMPNSTVYPRNKTAHPGNMLIQIS
PNITFSVVYNEINSGYAFTFKWSAEPGKPFHPPTAVFCYITEQGSHHHHHH
>1Q16A
MSKFLDRFRYFKQKGETFADGHGQLLNTNRDWEDGYRQRWQHDKIVRSTHGVNCTGSCSWKIYVK
NGLVTWETQQTDYPRTRPDLPNHEPRGCPRGASYSWYLYSANRLKYPMMRKRLMKMWREAKALHS
DPVEAWASIIEDADKAKSFKQARGRGGFVRSSWQEVNELIAASNVYTIKNYGPDRVAGFSPIPAM
SMVSYASGARYLSLIGGTCLSFYDWYCDLPPASPQTWGEQTDVPESADWYNSSYIIAWGSNVPQT
RTPDAHFFTEVRYKGTKTVAVTPDYAEIAKLCDLWLAPKQGTDAAMALAMGHVMLREFHLDNPSQ
YFTDYVRRYTDMPMLVMLEERDGYYAAGRMLRAADLVDALGQENNPEWKTVAFNTNGEMVAPNGS
IGFRWGEKGKWNLEQRDGKTGEETELQLSLLGSQDEIAEVGFPYFGGDGTEHFNKVELENVLLHK
LPVKRLQLADGSTALVTTVYDLTLANYGLERGLNDVNCATSYDDVKAYTPAWAEQITGVSRSQII
RIAREFADNADKTHGRSMIIVGAGLNHWYHLDMNYRGLINMLIFCGCVGQSGGGWAHYVGQEKLR
PQTGWQPLAFALDWQRPARHMNSTSYFYNHSSQWRYETVTAEELLSPMADKSRYTGHLIDFNVRA
ERMGWLPSAPQLGTNPLTIAGEAEKAGMNPVDYTVKSLKEGSIRFAAEQPENGKNHPRNLFIWRS
NLLGSSGKGHEFMLKYLLGTEHGIQGKDLGQQGGVKPEEVDWQDNGLEGKLDLVVTLDFRLSSTC
LYSDIILPTATWYEKDDMNTSDMHPFIHPLSAAVDPAWEAKSDWEIYKAIAKKFSEVCVGHLGKE

TDIVTLPIQHDSAAELAQPLDVKDWKKGECDLIPGKTAPHIMVVERDYPATYERFTSIGPLMEKI
GNGGKGIAWNTQSEMDLLRKLNYTKAEGPAKGQPMLNTAIDAAEMILTLAPETNGQVAVKAWAAL
SEFTGRDHTHLALNKEDEKIRFRDIQAQPRKIISSPTWSGLEDEHVSYNAGYTNVHELIPWRTLS
GRQQLYQDHQWMRDFGESLLVYRPPIDTRSVKEVIGQKSNGNQEKALNFLTPHQKWGIHSTYSDN
LLMLTLGRGGPVVWLSEADAKDLGIADNDWIEVFNSNGALTARAVVSQRVPAGMTMMYHAQERIV
NLPGSEITQQRGGIHNSVTRITPKPTHMIGGYAHLAYGFNYYGTVGSNRDEFVVVRKMKNIDWLD
GEGNDQVQESVK
>3CX5C
MAFRKSNVYLSLVNSYIIDSPQPSSINYWWNMGSLLGLCLVIQIVTGIFMAMHYSSNIELAFSSV
EHIMRDVHNGYILRYLHANGASFFFMVMFMHMAKGLYYGSYRSPRVTLWNVGVIIFILTIATAFL
GYCCVYGQMSHWGATVITNLFSAIPFVGNDIVSWLWGGFSVSNPTIQRFFALHYLVPFIIAAMVI
MHLMALHIHGSSNPLGITGNLDRIPMHSYFIFKDLVTVFLFMLILALFVFYSPNTLGHPDNYIPG
NPLVTPASIVPEWYLLPFYAILRSIPDKLLGVITMFAAILVLLVLPFTDRSVVRGNTFKVLSKFF
FFIFVFNFVLLGQIGACHVEVPYVLMGQIATFIYFAYFLIIVPVISTIENVLFYIGRVNK
>3I7QA
MFTGSIVAIVTPMDEKGNVCRASLKKLIDYHVASGTSAIVSVGTTGESATLNHDEHADVVMMTLD
LADGRIPVIAGTGANATAEAISLTQRFNDSGIVGCLTVTPYYNRPSQEGLYQHFKAIAEHTDLPQ
ILYNVPSRTGCDLLPETVGRLAKVKNIIGIREATGNLTRVNQIKELVSDDFVLLSGDDASALDFM
QLGGHGVISVTANVAARDMAQMCKLAAEGHFAEARVINQRLMPLHNKLFVEPNPIPVKWACKELG
LVATDTLRLPMTPITDSGRETVRAALKHAGLL
>3P1GA
GSILAETHGTRPDLTDQPIPDADYTWYTDGSSFLQEGQRRAGAAVTTETEVIWARALPAGTSAQR
AELIALTQALKMAEGKKLNVYTDSRYAFATAHVHSEGREIKNKNEILALLKALFLPKRLSIIHCP
GHQKGNSAEARGNRMADQAAREAAMKAVLETSTLL
>3EA6A
QGDIGIDNLRNFYTKKDFVDLKDVKDNDTPIANQLQFSNESYDLISESKDFNKFSNFKGKKLDVF
GISYNGQCNTKYIYGGVTATNEYLDKSRNIPINIWINGNHKTISTNKVSTNKKFVTAQEIDVKLR
KYLQEEYNIYGHNGTKKGEEYGHKSKFYSGFNIGKVTFHLNNNDTFSYDLFYTGDDGLPKSFLKI
YEDNKTVESEKFHLDVDISYKETI
>1EZ6A
ATSTKKLHKEPATLIKAIDGDTVKLMYKGQPMVFRLLLVDIPETKHPKKGVEKYGPEAAAFTKKM
VENAKKIEVEFDKGQRTDKYGRGLAYIYADGKMVNEALVRQGLAKVAYVYKGNNTHEQLLRKAEA
QAKKEKLNIWSEDNADSGQ
>3GXBA
MVLDVAFVLEGSDKIGEADFNRSKEFMEEVIQRMDVGQDSIHVTVLQYSYMVTVEYPFSEAQSKG
DILQRVREIRYQGGNRTNTGLALRYLSDHSFLVSQGDREQAPNLVYMVTGNPASDEIKRLPGDIQ
VVPIGVGPNANVQELERIGWPNAPILIQDFETLPREAPDLVLQRCCSPHHHHHH
>1LG7A
VDEMDTHDPHQLRYEKFFFTVKMTVRSNRPFRTYSDVAAAVSHWDHMYIGMAGKRPFYKILAFLG
SSNLKATPAVLADQGQPEYHAHCEGRAYLPHRMGKTPPMLNVPEHFRRPFNIGLYKGTVELTMTI
YDDESLEAAPMIWDHFNSSKFSDFREKALMFGLIVEKKASGAWVLDSVSHFK
>2Y0GA
MAHHHHHHGHHHQLVSKGEELFTGVVPILVELDGDVNGHKFSVSGEGEGDATYGKLTLKFICTTG
KLPVPWPTLVTTLXVQCFSRYPDHMKQHDFFKSAMPEGYVQERTIFFKDDGNYKTRAEVKFEGDT

LVNRIELKGIDFKEDGNILGHKLEYNYNSHNVYIMADKQKNGIKVNFKIRHNIEDGSVQLADHYQ
QNTPIGDGPVLLPDNHYLSTQSALSKDPNEKRDHMVLLEFVTAAGITLGMDELYK
>1K3UB
TTLLNPYFGEFGGMYVPQILMPALNQLEEAFVSAQKDPEFQAQFADLLKNYAGRPTALTKCQNIT
AGTRTTLYLKREDLLHGGAHKTNQVLGQALLAKRMGKSEIIAETGAGQHGVASALASALLGLKCR
IYMGAKDVERQSPNVFRMRLMGAEVIPVHSGSATLKDACNEALRDWSGSYETAHYMLGTAAGPHP
YPTIVREFQRMIGEETKAQILDKEGRLPDAVIACVGGGSNAIGMFADFINDTSVGLIGVEPGGHG
IETGEHGAPLKHGRVGIYFGMKAPMMQTADGQIEESYSISAGLDFPSVGPQHAYLNSIGRADYVS
ITDDEALEAFKTLCRHEGIIPALESSHALAHALKMMREQPEKEQLLVVNLSGRGDKDIFTVHDIL
KARGEI
>3NYSA
MIEFIDLKNQQARIKDKIDAGIQRVLRHGQYILGPEVTELEDRLADFVGAKYCISCANGTDALQI
VQMALGVGPGDEVITPGFTYVATAETVALLGAKPVYVDIDPRTYNLDPQLLEAAITPRTKAIIPV
SLYGQCADFDAINAIASKYGIPVIEDAAQSFGASYKGKRSCNLSTVACTSFFPSAPLGCYGDGGA
IFTNDDELATAIRQIARHGQDRRYHHIRVGVNSRLDTLQAAILLPKLEIFEEEIALRQKVAAEYD
LSLKQVGIGTPFIEVNNISVYAQYTVRMDNRESVQASLKAAGVPTAVHYPIPLNKQPAVADEKAK
LPVGDKAATQVMSLPMHPYLDTASIKIICAALTNLEHHHHHH
>2A6ZA
GSDASKLSSDYSLPDLINTRKVPNNWQTGEQASLEEGRIVLTSNQNSKGSLWLKQGFDLKDSFTM
EWTFRSVGYSGQTDGGISFWFVQDSNIPRDKQLYNGPVNYDGLQLLVDNNGPLGPTLRGQLNDGQ
KPVDKTKIYDQSFASCLMGYQDSSVPSTIRVTYDLEDDNLLKVQVDNKVCFQTRKVRFPSGSYRI
GVTAQNGAVNNNAESFEIFKMQFFNGV
>1G72B
YDGQNCKEPGNCWENKPGYPEKIAGSKYDPKHDPVELNKQEESIKAMDARNAKRIANAKSSGNFV
FDVK
>1A8P
SNLNVERVLSVHHWNDTLFSFKTTRNPSLRFENGQFVMIGLEVDGRPLMRAYSIASPNYEEHLEF
FSIKVQNGPLTSRLQHLKEGDELMVSRKPTGTLVTSDLLPGKHLYMLSTGTGLAPFMSLIQDPEV
YERFEKVVLIHGVRQVNELAYQQFITEHLPQSEYFGEAVKEKLIYYPTVTRESFHNQGRLTDLMR
SGKLFEDIGLPPINPQDDRAMICGSPSMLDESCEVLDGFGLKISPRMGEPGDYLIERAFVEK
>1A8Y
GLDFPEYDGVDRVINVNAKNYKNVFKKYEVLALLYHEPPEDDKASQRQFEMEELILELAAQVLED
KGVGFGLVDSEKDAAVAKKLGLTEEDSIYVFKEDEVIEYDGEFSADTLVEFLLDVLEDPVELIEG
ERELQAFENIEDEIKLIGYFKNKDSEHYKAFKEAAEEFHPYIPFFATFDSKVAKKLTLKLNEIDF
YEAFMEEPVTIPDKPNSEEEIVNFVEEHRRSTLRKLKPESMYETWEDDMDGIHIVAFAEEADPDG
YEFLEILKSVAQDNTDNPDLSIIWIDPDDFPLLVPYWEKTFDIDLSAPQIGVVNVTDADSVWMEP
SAEELEDWLEDVL
>1ABE
NLKLGFLVKQPEEPWFQTEWKFADKAGKDLGFEVIKIAVPDGEKTLNAIDSLAASGAKGFVICTP
DPKLGSAIVAKARGYDMKVIAVDDQFVNAKGKPMDTVPLVMMAATKIGERQGQELYKEMQKRGWD
VKESAVMAITANELDTARRRTTGSMDALKAAGFPEKQIYQVPTKSNDIPGAFDAANSMLVQHPEV
KHWLIVGMNDSTVLGGVRATEGQGFKAADIIGIGINGVDAVSELSKAQATGFYGSLLPSPDVHGY
KSSEMLYNWVAKDVEPPKFTEVTDVVLITRDNFKEELEKKGLGGK
>1AIR

ATDTGGYAATAGGNVTGAVSKTATSMQDIVNIIDAARLDANGKKVKGGAYPLVITYTGNEDSLIN
AAAANICGQWSKDPRGVEIKEFTKGITIIGANGSSANFGIWIKKSSDVVVQNMRIGYLPGGAKDG
DMIRVDDSPNVWVDHNELFAANHECDGTPDNDTTFESAVDIKGASNTVTVSYNYIHGVKKVGLDG
SSSSDTGRNITYHHNYYNDVNARLPLQRGGLVHAYNNLYTNITGSGLNVRQNGQALIENNWFEKA
INPVTSRYDGKNFGTWVLKGNNITKPADFSTYSITWTADTKPYVNADSWTSTGTFPTVAYNYSPV
SAQCVKDKLPGYAGVGKNLATLTSTAC
>1AL3
TWPDKGSLYVATTHTQARYALPGVIKGFIERYPRVSLHMHQGSPTQIAEAVSKGNADFAIATEAL
HLYDDLVMLPCYHWNRSIVVTPEHPLATKGSVSIEELAQYPLVTYTFGFTGRSELDTAFNRAGLT
PRIVFTATDADVIKTYVRLGLGVGVIASMAVDPVSDPDLVKLDANGIFSHSTTKIGFRRSTFLRS
YMYDFIQRFAPHLTRDVVDTAVALRSNEDIEAMFKDIKLPEK
>1ALHA
MPVLENRAAQGDITAPGGARRLTGDQTAALRDSLSDKPAKNIILLIGDGMGDSEITAARNYAEGA
GGFFKGIDALPLTGQYTHYALNKKTGKPDYVTDSAASATAWSTGVKTYNGALGVDIHEKDHPTIL
EMAKAAGLATGNVSTAELQDATPAALVAHVTSRKCYGPSATSEKCPGNALEKGGKGSITEQLLNA
RADVTLGGGAKTFAETATAGEWQGKTLREQAEARGYQLVSDAASLNSVTEANQQKPLLGLFADGN
MPVRWLGPKATYHGNIDKPAVTCTPNPQRNDSVPTLAQMTDKAIELLSKNEKGFFLQVEGASIDK
QDHAANPCGQIGETVDLDEAVQRALEFAKKEGNTLVIVTANHAHASQIVAPDTKAPGLTQALNTK
DGAVMVMSYGNSEEDSQEHTGSQLRIAAYGPHAANVVGLTDQTDLFYTMKAALGLK
>1AMF
GKITVFAAASLTNAMQDIATQFKKEKGVDVVSSFASSSTLARQIEAGAPADLFISADQKWMDYAV
DKKAIDTATRQTLLGNSLVVVAPKASVQKDFTIDSKTNWTSLLNGGRLAVGDPEHVPAGIYAKEA
LQKLGAWDTLSPKLAPAEDVRGALALVERNEAPLGIVYGSDAVASKGVKVVATFPEDSHKKVEYP
VAVVEGHNNATVKAFYDYLKGPQAAEIFKRYGFTIK
>1AMK
SAKPQPIAAANWKCNGTTASIEKLVQVFNEHTISHDVQCVVAPTFVHIPLVQAKLRNPKYVISAE
NAIAKSGAFTGEVSMPILKDIGVHWVILGHSERRTYYGETDEIVAQKVSEACKQGFMVIACIGET
LQQREANQTAKVVLSQTSAIAAKLTKDAWNQVVLAYEPVWAIGTGKVATPEQAQEVHLLLRKWVS
ENIGTDVAAKLRILYGGSVNAANAATLYAKPDINGFLVGGASLKPEFRDIIDATR
>1AMX
TSSVFYYKTGDMLPEDTTHVRWFLNINNEKSYVSKDITIKDQIQGGQQLDLSTLNINVTGTHSNY
YSGQSAITDFEKAFPGSKITVDNTKNTIDVTIPQGYGSYNSFSINYKTKITNEQQKEFVNNSQAW
YQEHGKEEVNGKSFNHTVHN
>1ARB
GVSGSCNIDVVCPEGDGRRDIIRAVGAYSKSGTLACTGSLVNNTANDRKMYFLTAHHCGMGTAST
AASIVVYWNYQNSTCRAPNTPASGANGDGSMSQTQSGSTVKATYATSDFTLLELNNAANPAFNLF
WAGWDRRDQNYPGAIAIHHPNVAEKRISNSTSPTSFVAWGGGAGTTHLNVQWQPSGGVTEPGSSG
SPIYSPEKRVLGQLHGGPSSCSATGTNRSDQYGRVFTSWTGGGAAASRLSDWLDPASTGAQFIDG
LDS
>1ARU
SVTCPGGQSTSNSQCCVWFDVLDDLQTNFYQGSKCESPVRKILRIVFHDAIGFSPALTAAGQFGG
GGADGSIIAHSNIELAFPANGGLTDTIEALRAVGINHGVSFGDLIQFATAVGMSNCPGSPRLEFL
TGRSNSSQPSPPSLIPGPGNTVTAILDRMGDAGFSPDEVVDLLAAHSLASQEGLNSAIFRSPLDS
TPQVFDTQFYIETLLKGTTQPGPSLGFAEELSPFPGEFRMRSDALLARDSRTACRWQSMTSSNEV

MGQRYRAAMAKMSVLGFDRNALTDCSDVIPSAVSNNAAPVIPGGLTVDDIEVSCPSEPFPEIATA
SGPLPSLAPAP
>1AT0
CFTPESTALLESGVRKPLGELSIGDRVLSTANGQAVYSEVILFDRNLEQQNFVQLHTDGGAVLTV
TPAHLVSVWQPESQKLTFVFADRIEEKNQVLVRDVETGELRPQRVVKVGSVRSKGVVAPLTREGT
IVVNSVAASCYA
>1AV4
ASPFRLASAGEISEVQGILRTAGLLGPEKRIAYLGVLDPARGAGSEAEDRRFRVFIHDVSGARPQ
EVTVSVTNGTVISAVELDTAATGELPVLEEEFEVVEQLLATDERWLKALAARNLDVSKVRVAPLS
AGVFEYAEERGRRILRGLAFVQDFPEDSAWAHPVDGLVAYVDVVSKEVTRVIDTGVFPVPAEHGN
YTDPELTGPLRTTQKPISITQPEGPSFTVTGGNHIEWEKWSLDVGFDVREGVVLHNIAFRDGDRL
RPIINRASIAEMVVPYGDPSPIRSWQNYFDTGEYLVGQYANSLELGCDCLGDITYLSPVISDAFG
NPREIRNGICMHEEDWGILAKHSDLWSGINYTRRNRRMVISFFTTIGNDYGFYWYLYLDGTIEFE
AKATGVVFTSAFPEGGSDNISQLAPGLGAPFHQHIFSARLDMAIDGFTNRVEEEDVVRQTMGPGN
ERGNAFSRKRTVLTRESEAVREADARTGRTWIISNPESKNRLNEPVGYKLHAHNQPTLLADPGSS
IARRAAFATKDLWVTRYADDERYPTGDFVNQHSGGAGLPSYIAQDRDIDGQDIVVWHTFGLTHFP
RVEDWPIMPVDTVGFKLRPEGFFDRSPVLDVPAN
>1AYL
MRVNNGLTPQELEAYGISDVHDIVYNPSYDLLYQEELDPSLTGYERGVLTNLGAVAVDTGIFTGR
SPKDKYIVRDDTTRDTFWWADKGKGKNDNKPLSPETWQHLKGLVTRQLSGKRLFVVDAFCGANPD
TRLSVRFITEVAWQAHFVKNMFIRPSDEELAGFKPDFIVMNGAKCTNPQWKEQGLNSENFVAFNL
TERMQLIGGTWYGGEMKKGMFSMMNYLLPLKGIASMHCSANVGEKGDVAVFFGLSGTGKTTLSTD
PKRRLIGDDEHGWDDDGVFNFEGGCYAKTIKLSKEAEPEIYNAIRRDALLENVTVREDGTIDFDD
GSKTENTRVSYPIYHIDNIVKPVSKAGHATKVIFLTADAFGVLPPVSRLTADQTQYHFLSGFTAK
LAPTPTFSACFGAAFLSLHPTQYAEVLVKRMQAAGAQAYLVNTGWNGTGKRISIKDTRAIIDAIL
NGSLDNAETFTLPMFNLAIPTELPGVDTKILDPRNTYASPEQWQEKAETLAKLFIDNFDKYTDTP
AGAALVAAGPKL
>1B51A
ADVPAGVQLADKQTLVRNNGSEVQSLDPHKIEGVPESNVSRDLFEGLLISDVEGHPSPGVAEKWE
NKDFKVWTFHLRENAKWSDGTPVTAHDFVYSWQRLADPNTASPYASYLQYGHIANIDDIIAGKKP
ATDLGVKALDDHTFEVTLSEPVPYFYKLLVHPSVSPVPKSAVEKFGDKWTQPANIVTNGAYKLKN
WVVNERIVLERNPQYWDNAKTVINQVTYLPISSEVTDVNRYRSGEIDMTYNNMPIELFQKLKKEI
PNEVRVDPYLCTYYYEINNQKAPFNDVRVRTALKLALDRDIIVNKVKNQGDLPAYSYTPPYTDGA
KLVEPEWFKWSQQKRNEEAKKLLAEAGFTADKPLTFDLLYNTSDLHKKLAIAVASIWKKNLGVNV
NLENQEWKTFLDTRHQGTFDVARAGWCADYNEPTSFLNTMLSDSSNNTAHYKSPAFDKLIADTLK
VADDTQRSELYAKAEQQLDKDSAIVPVYYYVNARLVKPWVGGYTGKDPLDNIYVKNLYIIKH
>1B6A
KVQTDPPSVPICDLYPNGVFPKGQECEYPEEKKALDQASEEIWNDFREAAEAHRQVRKYVMSWIK
PGMTMIEICEKLEDCSRKLIKENGLNAGLAFPTGCSLNNCAAHYTPNAGDTTVLQYDDICKIDFG
THISGRIIDCAFTVTFNPKYDTLLKAVKDATNTGIKCAGIDVRLCDVGEAIQEVMESYEVEIDGK
TYQVKPIRNLNGHSIGQYRIHAGKTVPIVKGGEATRMEEGEVYAIETFGSTGKGVVHDDMECSHY
MKNFDVGHVPIRLPRTKHLLNVINENFGTLAFCRRWLDRLGESKYLMALKNLCDLGIVDPYPPLC
DIKGSYTAQFEHTILLRPTCKEVVSRGDDY
>1BB9

TTGRLDLPPGFMFKVQAQHDYTATDTDELQLKAGDVVLVIPFQNPEEQDEGWLMGVKESDWNQHK
ELEKCRGVFPENFTERVQ
>1BDB
MKLKGEAVLITGGASGLGRALVDRFVAEGAKVAVLDKSAERLAELETDHGDNVLGIVGDVRSLED
QKQAASRCVARFGKIDTLIPNAGIWDYSTALVDLPEESLDAAFDEVFHINVKGYIHAVKACLPAL
VASRGNVIFTISNAGFYPNGGGPLYTAAKHAIVGLVRELAFELAPYVRVNGVGSGGINSDLRGPS
SLGPLADMLKSVLPIGRMPEVEEYTGAYVFFATRGDAAPATGALLNYDGGLGVRGFFSGAGGNDL
LEQLNIH
>1BFD
ASVHGTTYELLRRQGIDTVFGNPGSNELPFLKDFPEDFRYILALQEACVVGIADGYAQASRKPAF
INLHSAAGTGNAMGALSNAWNSHSPLIVTAGQQTRAMIGVEALLTNVDAANLPRPLVKWSYEPAS
AAEVPHAMSRAIHMASMAPQGPVYLSVPYDDWDKDADPQSHHLFDRHVSSSVRLNDQDLDILVKA
LNSASNPAIVLGPDVDAANANADCVMLAERLKAPVWVAPSAPRCPFPTRHPCFRGLMPAGIAAIS
QLLEGHDVVLVIGAPVFRYHQYDPGQYLKPGTRLISVTCDPLEAARAPMGDAIVADIGAMASALA
NLVEESSRQLPTAAPEPAKVDQDAGRLHPETVFDTLNDMAPENAIYLNESTSTTAQMWQRLNMRN
PGSYYFCAAGGLGFALPAAIGVQLAEPERQVIAVIGDGSANYSISALWTAAQYNIPTIFVIMNNG
TYGALRWFAGVLEAENVPGLDVPGIDFRALAKGYGVQALKADNLEQLKGSLQEALSAKGPVLIEV
STV
>1BG2
DLAECNIKVMCRFRPLNESEVNRGDKYIAKFQGEDTVVIASKPYAFDRVFQSSTSQEQVYNDCAK
KIVKDVLEGYNGTIFAYGQTSSGKTHTMEGKLHDPEGMGIIPRIVQDIFNYIYSMDENLEFHIKV
SYFEIYLDKIRDLLDVSKTNLSVHEDKNRVPYVKGCTERFVCSPDEVMDTIDEGKSNRHVAVTNM
NEHSSRSHSIFLINVKQENTQTEQKLSGKLYLVDLAGSEKVSKTGAEGAVLDEAKNINKSLSALG
NVISALAEGSTYVPYRDSKMTRILQDSLGGNCRTTIVICCSPSSYNESETKSTLLFGQRAKTI
>1BG6
SKTYAVLGLGNGGHAFAAYLALKGQSVLAWDIDAQRIKEIQDRGAIIAEGPGLAGTAHPDLLTSD
IGLAVKDADVILIVVPAIHHASIAANIASYISEGQLIIILNPGATGGALEFRKILRENGAPEVTIG
ETSSMLFTCRSERPGQVTVNAIKGAMDFACLPAAKAGWALEQIGSVLPQYVAVENVLHTSLTNVN
AVMHPLPTLLNAARCESGTPFQYYLEGITPSVGSLAEKVDAERIAIAKAFDLNVPSVCEWYPATI
YEAVQGNPAYRGIAGPINLNTRYFFEDVSTGLVPLSELGRAVNVPTPLIDAVLDLISSLIDTDFR
KEGRTLEKLGLSGLTAAGIRSAVE
>1CHD
LLSSEKLIAIGASTGGTEAIRHVLQPLPLSSPAVIITQHMPPGFTRSFAERLNKLCQISVKEAED
GERVLPGHAYIAPGDKHMELARSGANYQIKIHDGPPVNRHRPSVDVLFHSVAKHAGRNAVGVILT
GMGNDGAAGMLAMYQAGAWTIAQNEASCVVFGMPREAINMGGVSEVVDLSQVSQQMLAKISAGQA
IRI
>1CIY
YTPIDISLSLTQFLLSEFVPGAGFVLGLVDIIWGIFGPSQWDAFLVQIEQLINQRIEEFARNQAI
SRLEGLSNLYQIYAESFREWEADPTNPALREEMRIQFNDMNSALTTAIPLLAVQNYQVPLLSVYV
QAANLHLSVLRDVSVFGQRWGFDAATINSRYNDLTRLIGNYTDYAVRWYNTGLERVWGPDSRDWV
RYNQFRRELTLTVLDIVALFSNYDSRRYPIRTVSQLTREIYTNPVLENFDGSFRGMAQRIEQNIR
QPHLMDILNSITIYTDVHRGFNYWSGHQITASPVGFSGPEFAFPLFGNAGNAAPPVLVSLTGLGI
FRTLSSPLYRRIILGSGPNNQELFVLDGTEFSFASLTTNLPSTIYRQRGTVDSLDVIPPQDNSVP
PRAGFSHRLSHVTMLSQAAGAVYTLRAPTFSWQHRSAEFNNIIPSSQITQIPLTKSTNLGSGTSV

VKGPGFTGGDILRRTSPGQISTLRVNITAPLSQRYRVRIRYASTTNLQFHTSIDGRPINQGNFSA
TMSSGSNLQSGSFRTVGFTTPFNFSNGSSVFTLSAHVFNSGNEVYIDRIEFVPAEVT
>1CLC
IETKVSAAKITENYQFDSRIRLNSIGFIPNHSKKATIAANCSTFYVVKEDGTIVYTGTATSMFDN
DTKETVYIADFSSVNEEGTYYLAVPGVGKSVNFKIAMNVYEDAFKTAMLGMYLLRCGTSVSATYN
GIHYSHGPCHTNDAYLDYINGQHTKKDSTKGWHDAGDYNKYVVNAGITVGSMFLAWEHFKDQLEP
VALEIPEKNNSIPDFLDELKYEIDWILTMQYPDGSGRVAHKVSTRNFGGFIMPENEHDERFFVPW
SSAATADFVAMTAMAARIFRPYDPQYAEKCINAAKVSYEFLKNNPANVFANQSGFSTGEYATVSD
ADDRLWAAAEMWETLGDEEYLRDFENRAAQFSKKIEADFDWDNVANLGMFTYLLSERPGKNPALV
QSIKDSLLSTADSIVRTSQNHGYGRTLGTTYYWGCNGTVVRQTMILQVANKISPNNDYVNAALDA
ISHVFGRNYYNRSYVTGLGINPPMNPHDRRSGADGIWEPWPGYLVGGGWPGPKDWVDIQDSYQTN
EIAINWNAALIYALAGFVNYN
>1CNV
DISSTEIAVYWGQREDGLLRDTCKTNNYKIVFISFLDKFGCEIRKPELELEGVCGPSVGNPCSFL
ESQIKECQRMGVKVFLALGGPKGTYSACSADYAKDLAEYLHTYFLSERREGPLGKVALDGIHFDI
QKPVDELNWDNLLEELYQIKDVYQSTFLLSAAPGCLSPDEYLDNAIQTRHFDYIFVRFYNDRSCQ
YSTGNIQRIRNAWLSWTKSVYPRDKNLFLELPASQATAPGGGYIPPSALIGQVLPYLPDLQTRYA
GIALWNRQADKETGYSTNIIRYL
>1COT
DGDAAKGEKEFNKCKACHMIQAPDGTDIIKGGKTGPNLYGVVGRKIASEEGFKYGEGILEVAEKN
PDLTWTEADLIEYVTDPKPWLVKMTDDKGAKTKMTFKMGKNQADVVAFLAQNSPDA
>1CPO
EPGSGIGYPYDNNTLPYVAPGPTDSRAPCPALNALANHGYIPHDGRAISRETLQNAFLNHMGIAN
SVIELALTNAFVVCEYVTGSDCGDSLVNLTLLAEPHAFEHDHSFSRKDYKQGVANSNDFIDNRNF
DAETFQTSLDVVAGKTHFDYADMNEIRLQRESLSNELDFPGWFTESKPIQNVESGFIFALVSDFN
LPDNDENPLVRIDWWKYWFTNESFPYHLGWHPPSPAREIEFVTSASSAVLAASVTSTPSSLPSGA
IGPGAEAVPLSFASTMTPFLLATNAPYYAQDPTLGPND
>1CV8
NEQYVNKLENFKIRETQGNNGWCAGYTMSALLNATYNTNKYHAEAVMRFLHPNLQGQQFQFTGLT
PREMIYFGQTQGRSPQLLNRMTTYNEVDNLTKNNKGIAILGSRVESRNGMHAGHAMAVVGNAKLN
NGQEVIIIWNPWDNGFMTQDAKNNVIPVSNGDHYQWYSSIYGY
>1CVL
ADTYAATRYPVILVHGLAGTDKFANVVDYWYGIQSDLQSHGAKVYVANLSGFQSDDGPNGRGEQL
LAYVKQVLAATGATKVNLIGHSQGGLTSRYVAAVAPQLVASVTTIGTPHRGSEFADFVQDVLKTD
PTGLSSTVIAAFVNVFGTLVSSSHNTDQDALAALRTLTTAQTATYNRNFPSAGLGAPGSCQTGAA
TETVGGSQHLLYSWGGTAIQPTSTVTGATDTSTGTLDVANVTDPSTLALLATGAVMINRASGQND
GLVSRCSSLFGQVISTSYHWNHLDEINQLLGVRGANAEDPVAVIRTHVNRLKLQGV
>1CYO
SKAVKYYTLEEIQKHNNSKSTWLILHYKVYDLTKFLEEHPGGEEVLREQAGGDATENFEDVGHST
DARELSKTFIIGELHPDDRSKIT
>4FHZA
MHHHHHHSSGLVPRGSGMKETAAAKFERQHMDSPDLGTDDDDKAMADIMTRKLTFGRRGAAPGEA
TSLVVFLHGYGADGADLLGLAEPLAPHLPGTAFVAPDAPEPCRANGFGFQWFPIPWLDGSSETAA
AEGMAAAARDLDAFLDERLAEEGLPPEALALVGFSQGTMMALHVAPRRAEEIAGIVGFSGRLLAP

ERLAEEARSKPPVLLVHGDADPVVPFADMSLAGEALAEAGFTTYGHVMKGTGHGIAPDGLSVALA
FLKERLPDACGRTRAPPPPPLRSGC
>4FHRA
GTKFSKEQLRTFQMIHENFGRALSTYLSGRLRTFVDVEISIDQLTYEEFIRSVMIPSFIVIFTGD
VFEGSAIFEMRLDLFYTMLDIIMGGPGENPPNRPPTEIETSIMRKEVTNMLTLLAQAWSDFQYFI
PSIENVETNPQFVQIVPPNEIVLLVTASVSWGEFTSFINVCWPFSLLEPLLEKLSDR
>4FGZA
MTLIENLNSDKTFLENNQYTDEGVKVYEFIFGENYISSGGLEATKKILSDIELNENSKVLDIGSG
LGGGCMYINEKYGAHTHGIDICSNIVNMANERVSGNNKIIFEANDILTKEFPENNFDLIYSRDAI
LHLSLENKNKLFQKCYKWLKPTGTLLITDYCATEKENWDDEFKEYVKQRKYTLITVEEYADILTA
CNFKNVVSKDLSDYWNQLLEVEHKYLHENKEEFLKLFSEKKFISLDDGWSRKIKDSKRKMQRWGY
FKATKN
>4FFXA
GSHMAAGGDHGSPDSYRSPLASRYASPEMCFVFSDRYKFRTWRQLWLWLAEAEQTLGLPITDEQI
REMKSNLENIDFKMAAEEEKRLRHDVMAHVHTFGHCCPKAAGIIHLGATSCYVGDNTDLIIILRNA
LDLLLPKLARVISRLADFAKERASLPTLGFTHFQPAQLTTVGKRCCLWIQDLCMDLQNLKRVRDD
LRFRGVKGTTGTQASFLQLFEGDDHKVEQLDKMVTEKAGFKRAFIITGQTYTRKVDIEVLSVLAS
LGASVHKICTDIRLLANLKEMEEPFEKQQIGSSAMPYKRNPMRSERCCSLARHLMTLVMDPLQTA
SVQWFERTLDDSANRRICLAEAFLTADTILNTLQNISEGLVVYPKVIERRIRQELPFMATENIIM
AMVKAGGSRQDCHEKIRVLSQQAASVVKQEGGDNDLIERIQVDAYFSPIHSQLDHLLDPSSFTGR
ASQQVQRFLEEEVYPLLKPYESVMKVKAELCL
>4AVXA
SMGRGSGTFERLLDKATSQLLLETDWESILQICDLIRQGDTQAKYAVNSIKKKVNDKNPHVALYA
LEVMESVVKNCGQTVHDEVANKQTMEELKDLLKRQVEVNVRNKILYLIQAWAHAFRNEPKYKVVQ
DTYQIMKVEGHVFPEFKESDAMFAAERAPDWVDAEECHRCRVQFGVMTRKHHCRACGQIFCGKCS
SKYSTIPKFGIEKEVRVCEPCYEQLNRKAEG
>4FEIA
QGGPWTPAADWRDAGTHLDLLLDVPGVDAGTLALAEDGGQLTVSGERPGTEHLLRSERPSGRFVR
ELAFPEPVRPASGVASLAGGVLTVRFEKLRPTIDVTA
>4FF1A
MGGSHHHHHHRSESTVTEELKEGIDAVYPSLVGTADSKAEGIKNYFKLSFTLPEEQKSRTVGSEA
PLKDVAQALSSRARYELFTEKETANPAFNGEVIKRYKELMEHGEGIADILRSRLAKFLNTKDVGK
RFAQGTEANRWVGGKLLNIVEQDGDTFKYNEQLLQTAVLAGLQWRLTATSNTAIKDAKDVAAITG
IDQALLPEGLVEQFDTGMTLTEAVSSLAQKIESYWGLSRNPNAPLGYTKGIPTAMAAEILAAFVE
STDVVENIVDMSEIDPDNKKTIGLYTITELDSFDPINSFPTAIEEAVLVNPTEKMFFGDDIPPVA
NTQLRNPAVRNTPEQKAALKAEQATEFYVHTPMVQFYETLGKDRILELMGAGTLNKELLNDNHAK
SLEGKNRSVEDSYNQLFSVIEQVRAQSEDISTVPIHYAYNMTRVGRMQMLGKYNPQSAKLVREAI
LPTKATLDLSNQNNEDFSAFQLGLAQALDIKVHTMTREVMSDELTKLLEGNLKPAIDMMVEFNTT
GSLPENAVDVLNTALGDRKSFVALMALMEYSRYLVAEDKSAFVTPLYVEADGVTNGPINAMMLMT
GGLFTPDWIRNIAKGGLFIGSPNKTMNEHRSTADNNDLYQASTNALMESLGKLRSNYASNMPIQS
QIDSLLSLMDLFLPDINLGENGALELKRGIAKNPLTITIYGSGARGIAGKLVSSVTDAIYERMSD
VLKARAKDPNISAAMAMFGKQAASEAHAEELLARFLKDMETLTSTVPVKRKGVLELQSTGTGAKG
KINPKTYTIKGEQLKALQENMLHFFVEPLRNGITQTVGESLVYSTEQLQKATQIQSVVLEDMFKQ
RVQEKLAEKAKDPTWKKGDFLTQKELNDIQASLNNLAPMIETGSQTFYIAGSENAEVANQVLATN

LDDRMRVPMSIYAPAQAGVAGIPFMTIGTGDGMMMQTLSTMKGAPKNTLKIFDGMNIGLNDITDA
SRKANEAVYTSWQGNPIKNVYESYAKFMKNVDFSKLSPEALEAIGKSALEYDQRENATVDDIANA
ASLIERNLRNIALGVDIRHKVLDKVNLSIDQMAAVGAPYQNNGKIDLSNMTPEQQADELNKLFRE
ELEARKQKVAKAR
>4FE1A
MTISPPEREPKVRVVVDNDPVPTSFEKWAKPGHFDRTLARGPQTTTWIWNLHALAHDFDTHTSDL
EDISRKIFSAHFGHLAVVFIWLSGMYFHGAKFSNYEAWLADPTGIKPSAQVVWPIVGQGILNGDV
GGGFHGIQITSGLFQLWRASGITNEFQLYCTAIGGLVMAGLMLFAGWFHYHKRAPKLEWFQNVES
MLNHHLAGLLGLGSLAWAGHQIHVSLPINKLLDAGVAAKDIPLPHEFILNPSLMAELYPKVDWGF
FSGVIPFFTFNWAAYSDFLTFNGGLNPVTGGLWLSDTAHHHLAIAVLFIIAGHMYRTNWGIGHSL
KEILEAHKGPFTGAGHKGLYEVLTTSWHAQLAINLAMMGSLSIIVAQHMYAMPPYPYLATDYPTQ
LSLFTHHMWIGGFLVVGGAAHGAIFMVRDYDPAMNQNNVLDRVLRHRDAIISHLNWVCIFLGFHS
FGLYVHNDTMRAFGRPQDMFSDTGIQLQPVFAQWVQNLHTLAPGGTAPNAAATASVAFGGDVVAV
GGKVAMMPIVLGTADFMVHHIHAFTIHVTVLILLKGVLFARSSRLIPDKANLGFRFPCDGPGRGG
TCQVSGWDHVFLGLFWMYNCISVVIFHFSWKMQSDVWGTVAPDGTVSHITGGNFAQSAITINGWL
RDFLWAQASQVIGSYGSALSAYGLLFLGAHFIWAFSLMFLFSGRGYWQELIESIVWAHNKLKVAP
AIQPRALSIIQGRAVGVAHYLLGGIATTWAFFLARIISVG
>4AVPA
SMGPTSQRRGSLQLWQFLVALLDDPSNSHFIAWTGRGMEFKLIEPEEVARRWGIQKNRPAMNYDK
LSRSLRYYYEKGIMQKVAGERYVYKFVCDPEALFSMAFSDN
>4FD5A
MLDSKLNNIRFETISSKYYDDVIEHLRQTFFADEPLNKAVNLTRPGQGHPLLEQHSLSTLKDNVS
IMAISNDGDIAGVALNGILYGNTDIEKSREKLNEIQDESFKKIFKLLYEQNLKINLFKQFDVDKI
FEIRILSVDSRFRGKGLAKKLIEKSEELALDRGFQVMKTDATGAFSQRVVSSLGFITKCEINYTD
YLDENGEQIFVVDPPHEKLKIMCKVIN
>4FCIA
MSAKSRTIGIIGAPFSKGQPRGGVEEGPTVLRKAGLLEKLKEQECDVKDYGDLPFADIPNDSPFQ
IVKNPRSVGKASEQLAGKVAEVKKNGRISLVLGGDHSLAIGSISGHARVHPDLGVIWVDAHTDIN
TPLTTTSGNLHGQPVSFLLKELKGKIPDVPGFSWVTPCISAKDIVYIGLRDVDPGEHYILKTLGI
KYFSMTEVDRLGIGKVMEETLSYLLGRKKRPIHLSFDVDGLDPSFTPATGTPVVGGLTYREGLYI
TEEIYKTGLLSGLDIMEVNPSLGKTPEEVTRTVNTAVAITLACFGLAREGNHKPIDYLNPPK
>4F9KA
MSGLNDIFEAQKIEWHEHHHHHHENLYFQSHMEDESLKGCELYVQLHGIQQVLKDCIVHLCISKP
ERPMKFLREHFEKLEKEENRQILARQKSNS
>4F99B
GPGTRTGRLKKPFVKVEDMSQLYRPFYLQLTNMPFINYSIQKPCSPFDVDKPSSMQKQTQVKLRI
QTDGDKYGGTSIQLQLKEKKKKGYCECCLQKYEDLETHLLSEQHRNFAQSNQYQVVDDIVSKLVF
DFVEYEKDTPKKKR
>4F8CA
MAHHHHHHSSGLEVLFQGPPVSHSINNPSIQHVQDFATLSARSLRANVLLNSDDHSVPIHAKNPS
ELLEAIDNNISQTAQDWGVSIQEVEVILGSSKRIIEPVAGVTANTIMKLFLDNDIFSYSFEKGQS
LSLSQLQERLASLPAHKNFILRVNDGGLGHAYVIDFPATTNPSRDAFLYQSDLGEGVTREVRFED
WMTQKASHPISLDDINTHFIGIAQDQIDLAHIAKLFDVDGNVKMLRADHLISHKTSEFNFQLFEY
DLKNLENNMSIIKTH

>4F84A
MGSSHHHHHHSSGLVPRGSHMAAASAPVPGPGGASSTARGRIPAPATPYQEDIARYWNNEARPVN
LRLGDVDGLYHHHYGIGAVDHAALGDPGDGGYEARLIAELHRLESAQAEFLLDHLGPVGPGDTLV
DAGCGRGGSMVMAHQRFGCKVEGVTLSAAQAEFGNRRARELGIDDHVRSRVCNMLDTPFEKGTVA
ASWNNESSMYVDLHDVFAEHSRFLRVGGRYVTVTGCWNPRYGQPSKWVSQINAHFECNIHSRREY
LRAMADNRLVPQTVVDLTPETLPYWELRATSSLVTGIEEAFIESYRDGSFQYVLIAADRV
>4F6OA
EQAKAQLSNGYNNPNVNASNMYGPPQNMSLPPPQTQTIQGTDQPYQYSQCTGRRKALIIGINYIG
SKNQLRGCINDAHNIFNFLTNGYGYSSDDIVILTDDQNDLVRVPTRANMIRAMQWLVKDAQPNDS
LFLHYSGHGGQTEDLDGDEEDGMDDVIYPVDFETQGPIIDDEMHDIMVKPLQQGVRLTALFDSCH
SGTVLDLPYTYSTKGIIKEPNIWKDVGQDGLQAAISYATGNRAALIGSLGSIFKTVKGGMGNNVD
RERVRQIKFSAADVVMLSGSKDNQTSADAVEDGQNTGAMSHAFIKVMTLQPQQSYLSLLQNMRKE
LAGKYSQKPQLSSSHPIDVNLQFIM
>2LT5A
RPCKYKLKKSTNKFCVTCENQAPVHFVGVGSCGSGGSGIFLETSLSAGSDWLTFQKKHITNTRDV
DCDNIMSTNLFHCKDKNTFIYSRPEPVKAICKGIIASKNVLTTSEFYLSDCNVTS
>4F52B
GSMDVDTPSGTNSGAGKKRFEVKKWNAVALWAWDIVVDNCAICRNHIMDLCIECQANQASATSEE
CTVAWGVCNHAFHFHCISRWLKTRQVCPLDNREWEFQKYGH
>4F52E
GSMAVEELQSIIKRCQILEEQDFKEEDFGLFQLAGQRCIEEGHTDQLLEIIQNEKNKVIIKNMGW
NLVGPVVRCLLCKDKEDSKRKVYFLIFDLLVKLCNPKELLLGLLELIEEPSGKQISQSILLLLQP
LQTVIQKLHNKAYSIGLALSTLWNQLSLLPVPYSKEQIQMDDYGLCQCCKALIEFTKPFVEEVID
NKENSLENEKLKDELLKFCFKSLKCPLLTAQFFEQSEEGGNDPFRYFASEIIGFLSAIGHPFPKM
IFNHGRKKRTWNYLEFEEEENKQLADSMASLAYLVFVQGIHIDQLPMVLSPLYLLQFNMGHIEVF
LQRTEESVISKGLELLENSLLRIEDNSLLYQYLEIKSFLTVPQGLVKVMTLCPIETLRKKSLAML
QLYINKLDSQGKYTLFRCLLNTSNHSGVEAFIIQNIKNQIDMSLKRTRNNKWFTGPQLISLLDLV
LFLPEGAETDLLQNSDRIMASLNLLRYLVIKDNENDNQTGLWTELGNIENNFLKPLHIGLNMSKA
HYEAEIKNSQEAQKSKDLCSITVSGEEIPNMPPEMQLKVLHSALFTFDLIESVLARVEELIEIKT
KSTSEENIGIK
>4F3WA
GPGSMPDIDWKQLRDKATQVAAGAYAPYSRFPVGAAALVDDGRVVTGCNVENVSYGLALCAECGV
VCALHATGGGRLVALACVDGRGAPLMPCGRCRQLLFEHGGPELLVDHLAGPRRLGDLLPEPFHAD
LTGEP
>4F2ME
YPYDVPDYAGAQPARSPGLVPRGSRTANLNNGFYPVSSSEVGLVNKSVVLLPSFYTHTIVNITID
LGMKRSGYGQPIASTLSNITLPMQDNNTDVYCIRSDQFSVYVHSTCKSSLWDNIFKRNCTDVLDA
TAVIKTGTCPFSFDKLNNYLTFNKFCLSLSPVGANCKFDVAARTRTNEQVVRSLYVIYEEGDNIV
LVPRGSDYKDDDDK
>4F2ZA
MSSQVEHPAGGYKKLFETVEELSSPLTAHVTGRIPLWLTGSLLRCGPGLFEVGSEPFYHLFDGQA
LLHKFDFKEGHVTYHRRFIRTDAYVRAMTEKRIVITEFGTCAFPDPCKNIFSRFFSYFRGVEVTD
NALVNIYPVGEDYYACTETNFITKVNPETLETIKQVDLCNYVSVNGATAHPHIENDGTVYNIGNC
FGKNFSIAYNIVKIPPLQADKEDPISKSEIVVQFPCSDRFKPSYVHSFGLTPNYIVFVETPVKIN

LFKFLSSWSLWGANYMDCFESNETMGVWLHIADKKRKKYINNKYRTSPFNLFHHINTYEDHEFLI
VDLCCWKGFEFVYNYLYLANLRENWEEVKKNARKAPQPEVRRYVLPLNIDKADTGKNLVTLPNTT
ATAILCSDETIWLEPEVLFSGPRQAFEFPQINYQKYGGKPYTYAYGLGLNHFVPDRLCKLNVKTK
ETWVWQEPDSYPSEPIFVSHPDALEEDDGVVLSVVVSPGAGQKPAYLLILNAKDLSEVARAEVEI
NIPVTFHGLFKKS

>4F0VA

MGSSHHHHHHSSGENLYFEGSHMASMTGGQQMGRMDSLDQCIVNACKNSWDKSYLAGTPNKDNCS
GFVQSVAAELGVPMPRGNANAMVDGLEQSWTKLASGAEAAQKAAQGFLVIAGLKGRTYGHVAVVI
SGPLYRQKYPMCWCGSIAGAVGQSQGLKSVGQVWNRTDRDRLNYYVYSLASCSLPRAS

>4F02A

GPLGSMNPSAPSYPMASLYVGDLHPDVTEAMLYEKFSPAGPILSIRVCRDMITRRSLGYAYVNFQ
QPADAERALDTMNFDVIKGKPVRIMWSQRDPSLRKSGVGNIFIKNLDKSIDNKALYDTFSAFGNI
LSCKVVCDENGSKGYGFVHFETQEAAERAIEKMNGMLLNDRKVFVGRFKSRKEREAELGARAKEF
YPYDVPDYAGSSGRIVTD

>4ASUH

AEAAAAQAPAAGPGQMSFTFASPTQVFFNSANVRQVDVPTQTGAFGILAAHVPTLQVLRPGLVVV
HAEDGTTSKYFVSSGSVTVNADSSVQLLAEEAVTLDMLDLGAAKANLEKAQSELLGAADEATRAE
IQIRIEANEALVKALE

>4EY0A

HSNEKWFHGKLGAGRDGRHIAERLLTEYCIETGAPDGSFLVRESETFVGDYTLSFWRNGKVQHCR
IHSRQDAGTPKFFLTDNLVFDSLYDLITHYQQVPLRCNEFEMRLSEPVPQTNAHESKEWYHASLT
RAQAEHMLMRVPRDGAFLVRKRNEPNSYAISFRAEGKIKHCRVQQEGQTVMLGNSEFDSLVDLIS
YYEKHPLYRKMKLRYPINEEALEKIGTAEPDFGALFEGRNPGFYVEANPMP

>4ARZB

MSLEATDSKAMVLLMGVRRCGKSSICKVVFHNMQPLDTLYLESTSNPSLEHFSTLIDLAVMELPG
QLNYFEPSYDSERLFKSVGALVYVIDSQDEYINAITNLAMIIEYAYKVNPSINIEVLIHKVDGLS
EDFKVDAQRDIMQRTGEELLELGLDGVQVSFYLTSIFDHSIYEAFSRIVQKLIPELSFLENMLDN
LIQHSKIEKAFLFDVNSKIYVSTDSNPVDIQMYEVCSEFIDVTIDLFDLYKAPVLRNSQKSSDKD
NVINPRNELQNVSQLANGVIIYLRQMIRGLALVAIIRPNGTDMESCLTVADYNIDIFKKGLEDIW
ANARASQAKNSIEDDV

>4EVWA

MIVIPMAGMSSRFFKAGYTQPKYMLEAHGQTLFEHSVNSFAAYFASTPFLFIVRNVYDTAVFVRE
KATQLGIKQFYIAELHTETRGQAETVTLGLEELAKQGVDYQGSITVFNIDTFRPNFVFPDISQHS
DGYLEVFQGGGDNWSFAKPEHAGSTKVIQTAEKNPISDLCSTGLYHFNRKEDYLEAYREYVARPS
QEWERGELYIAPLYNELIQKGLNIHYHLIARHEVIFCGVPDEYTDFLRQPQPLEHHHHHH

>4EVFA

MPKVTDIANELKQAIDAKDEVQIAFIASEYSAESREKIAKAYVASYGKELPDDIKKALKGGSEES
LLMDLFSDRHEVRAQHIRDALSGRNDHMAFFDTVILCTPEDWHETVAAYTRMFKKPLVEDFMKDV
GRKEDWCLLMEKWMAHERVSRPGSPEDEAQRLDQAFDQKNTAYLIDFFGTVPSAEYRPIAEAFKA
QNGKSIEQAIATIYTKTDYYTFYCAHFALLGMHRLAAYLINCACNDKGDEKRMRRITGMMVDKCL
GAKHAYKIYGDMGTDIERCFDKRMAPILRTLWRVK

>4EUWA

MAHHHHHHVDDDDKMSENLYFQSSKNKPHVKRPMNAFMVWAQAARRKLADQYPHLHNAELSKTLG

KLWRLLNESEKRPFVEEAERLRVQHKKDHPDYKYQPRRRKS
>4ETYA
SMQEGSLPDITIFPNSSLMISQGTFVTVVCSYSDKHDLYNMVRLEKDGSTFMEKSTEPYKTEDEF
EIGPVNETITGHYSCIYSKGITWSERSKTLELKVIKENVIQTPAPGPTSEHLG
>4ETPB
GASEIAALEKEIAALEKEIAALEKEISKQEKFYNDTYNTVCKELLRSRRLENSIIEQKGTMRVYA
YVMEQNLPENLLFDYENGVITQGLSEHVYKFNRVIPHLKVSEDCFFTQEYSVYHDMALNQKKNFN
LISLSTTPHGSLRESLIKFLAEKDTIYQKQYVITLQFVFLSDDEFSQDMLLDYSHNDKDSIKLKF
EKHSISLDSKLVIIENGLEDLPLNFSADEHPNLPHSGMGIIKVQFFPRDSKSDGNNDPVPVDFYF
IELNNLKSIEQFDKSIFKKESAETPIALVLKKLISDTKSFFLLNLNDSKNVNKLLTISEEVQTQL
AKRKKKLT
>4ESVA
MSELFSERIPPQSIEAEQAVLGAVFLDPAALVPASEILIPEDFYRAAHQKIFHAMLRVADRGEPV
DLVTVTAELAASEQLEEIGGVSYLSELADAVPTAANVEYYARIVEEKSVLRRLIRTATSIAQDGY
TREDEIDVLLDEADRKIMEVSQRKHSGAFKNIKDILVQTYDNIEMLHNRDGEITGIPTGFTELDR
MTSGFQRSDLIIVAARPSVGKTAFALNIAQNVATKTNENVAIFSLEMSAQQLVMRMLCAEGNINA
QNLRTGKLTPEDWGKLTMAMGSLSNAGIYIDDTPSIRVSDIRAKCRRLKQESGLGMIVIDYLQLI
QGSGRSKENRQQEVSEISRSLKALARELEVPVIALSQLSRSVEQRQDKRPMMSDIRESGSIEQDA
DIVAFLYRDDYYNKDSENKNIIEIIIAKQRNGPVGTVQLAFIKEYNKFVNLERRFDEAQIPPGA
>4ESEA
SNAMSKVLVLKSSILATSSQSNQLADFFVEQWQAAHAGDQITVRDLAAQPIPVLDGELVGALRPS
GTALTPRQQEALALSDELIAELQANDVIVIAAPMYNFNIPTQLKNYFDMIARAGVTFRYTEKGPE
GLVTGKRAIILTSRGGIHKDTPTDLVVPYLRLFLGFIGITDVEFVFAEGIAYGPEVATKAQADAK
TLLAQVVAA
>2LS8A
MVCPIDWRAFQSNCYFPLTDNKTWAESERNCSGMGAHLMTISTEAEQNFIIQFLDRRLSYFLGLR
DENAKGQWRWVDQTPFNPRRVFWHKNEPDNSQGENCVVLVYNQDKWAWNDVPCNFEASRICKIPG
TTLNAENLYFQSHHHHHHWSHPQFEK
>4ERRA
MGQIFTVQELKERAKVFAKPIGASYQGILDQLDLVHQAKGRDQIAASFELNKKINDYIAEHPTSG
RNQALTQLKEQVTSALGLEHHHHHH
>4EPAA
GAMGQTSQQDESTLVVTASKQSSRSASANNVSSTVVSAPELSDAGVTASDKLPRVLPGLNIENSG
NMLFSTISLRGVSSAQDFYNPAVTLYVDGVPQLSTNTIQALTDVQSVELLRGPQGTLYGKSAQGG
IINIVTQQPDSTPRGYIEGGVSSRDSYRSKFNLSGPIQDGLLYGSVTLLRQVDDGDMINPATGSD
DLGGTRASIGNVKLRLAPDDQPWEMGFAASRECTRATQDAYVGWNDIKGRKLSISDGSPDPYMRR
CTDSQTLSGKYTTDDWVFNLISAWQQQHYSRTFPSGSLIVNMPQRWNQDVQELRAATLGDARTVD
MVFGLYRQNTREKLNSAYDMPTMPYLSSTGYTTAETLAAYSDLTWHLTDRFDIGGGVRFSHDKSS
TQYHGSMLGNPFGDQGKSNDDQVLGQLSAGYMLTDDWRVYTRVAQGYKPSGYNIVPTAGLDAKPF
VAEKSINYELGTRYETADVTLQAATFYTHTKDMQLYSGPVRMQTLSNAGKADATGVELEAKWRFA
PGWSWDINGNVIRSEFTNDSELYHGNRVPFVPRYGAGSSVNGVIDTRYGALMPRLAVNLVGPHYF
DGDNQLRQGTYATLDSSLGWQATERMNISVYVDNLFDRRYRTYGYMNGSSAVAQVNMGRTVGINT
RIDFF
>4EPCA

GSTTSTKPSQPSKPSGGTNNKLTVSANRGVAQIKPTNNGLYTTVYDSKGHKTDQVQKTLSVTKTA
TLGNNKFYLVEDYNSGKKYGWVKQGDVVYNTAKAPVKVNQTYNVKAGSTLYTVPWGTPKQVASKV
SGTGNQTFKATKQQQIDKATYLYGTVNGKSGWISKYYLTTASKPSNPTKPSTNNQLTVTNNSGVA
QINAKNSGLYTTVYDTKGKTTNQIQRTLSVTKAATLGDKKFYLVGDYNTGTNYGWVKQDEVIYNT
AKSPVKINQTYNVKPGVKLHTVPWGTYNQVAGTVSGKGDQTFKATKQQQIDKATYLYGTVNGKSG
WISKYYLTA
>4EOZA
GSNMVKVPECRLADELGGLWENSRFTDCCLCVAGQEFQAHKAILAARSPVFSAMFEHEMEESKKN
RVEINDVEPEVFKEMMCFIYTGKAPNLDKMADDLLAAADKYALERLKVMCEDALCSNLSVENAAE
ILILADLHSADQLKT
>4EODA
MAVTDLSLTNSSLMPTLNPMIQQLALAIAASWQSLPLKPYQLPEDLGYVEGRLEGEKLVIENRCY
QTPQFRKMELELAKVGKGLDILHCVMFPEPLYGLPLFGCDIVAGPGGVSAAIADLSPTQSDRQLP
AAYQKSLAELGQPEFEQQRELPPWGEIFSEYCLFIRPSNVTEEERFVQRVVDFLQIHCHQSIVAE
PLSEAQTLEHRQGQIHYCQQQQKNDKTRRVLEKAFGEAWAERYMSQVLFDVIQ
>4EO1A
PSEQTPEEICEAKPPIDGVFNNVFKGDEGGFYINYNGCEYEATGVTVCQNDGTVCSSSAWKPTGY
VPESG
>4EN6B
QTILPYPNGLYVINKGDGYMRTNDKDLIGTLLIESSTSGSIIQPRLRNTTRPLFNTSNPTIFSQE
YTEARLNDAFNIQLFNTSTTLFKFVEEAPTNKNISMKVYNTYEKYELINYQNGNIDDKAEYYLPS
LGKCEVSDAPSPQAPVVETPVDQDGFIQTGPNENIIVGVINPSENIEEISTPIPDDYTYNIPTSI
QNNACYVLFKVNTTGVYKITTKNNLPPLIIYEAIGSSNRNMNSNNLSNDNIKAIKYITGLNRSDA
KSYLIVSLFKDKNYYIRIPQISSSTTSQLIFKRELGNISDLADSTVNILDNLNTSGTHYYTRQSP
DVGNYISYQLTIPGDFNNIASSIFSFRTRNNQGIGTLYRLTESINGYNLITINNYSDLLNNVEPI
SLLNGATYIFRVKVTELNNYNIIFDAYRNS
>4EMOA
GSMAPPAGGAAAAASDLGSAAVLMAVHAAVRPLGAGPDAEAQLRRLQLSADPERPGRFRLELLGA
GPGAVNLEWPLESVSYTIRGPTQHELQPPPGGPGTLSMHFLNPQEAQRWAVLVRGATVEGQNGS
>4AQ1A
MASFTDVAPQYKDAIDFLVSTGATKGKTETKFGVYDEITRLDAAVILARVLKLDVGNAKDAGFTD
VPKDRAKYVNALVEAGVLNGKAPGKFGAYDPLTRVEMAKIIANAHKLKADDVKLPFTDVNDTWAP
YVKALYKYEVTKGKTPTSFGAYQNITRGDFAQFVYRAVNINAVPEIVEVTAVNSTTVKVTFNTQI
ADVDFTNFAIDNGLTVTKATLSRDKKSVEVVVNKPFTRNQEYTITATGIKNLKGETAKELTGKFV
WSVQDAVTVALNNSSLKVGEESGLTVKDQDGKDVVGAKVELTSSNTNIVVVSSGEVSVSAAKVTA
VKPGTADVTAKVTLPDGVVLTNTFKVTVTEVPVQVQNQGFTLVDNLSNAPQNTVAFNKAEKVTSM
FAGETKTVAMYDTKNGDPETKPVDFKDATVRSLNPIIATAAINGSELLVTANAGQSGKASFEVTF
KDNTKRTFTVDVKKEPVLQDIKVDATSVKLSDEAVGGGEVEGVNQKTIKVSAVDQYGKEIKFGTK
GKVTVTTNTEGLVIKNVNSDNTIDFDSGNSATDQFVVVATKDKIVNGKVEVKYFKNASDTTPTST
KTITVNVVNVKADATPVGLDIVAPSEIDVNAPNTASTADVDFINFESVEIYTLDSNGNRLKKVTP
TATTLVGTNDYVEVNGNVLQFKGNDELTLLTSSSTVNVDVTADGITKRIPVKYINSASVPASATV
ATSPVTVKLNSSDNDLTFEELIFGVIDPTQLVKDEDINEFIAVSKAAKNDGYLYNKPLVTVKDAS
GEVIPTGANVYGLNHDATNGNIWFDEEQAGLAKKFSDVHFDVDFSLANVVKTGSGTVSSSPSLSD
AIQLTNSGDAVSFTLVIKSIYVKGADKDDNNLLAAPVSVNVTVTKGS

>4EMZB

MGGKWSKSSVIGWPAVRERMRRAEPAADGVGAVSRDLEKHGAITSSNTAANNAACAWLEAQEEEE
VGFPVTPQVPLRPMTYKAAVDLSHFLKEKGGLEGLIHSQRRQDILDLWIYHTQGYFPDWQNYTPG
PGVRYPLTFGWCYKLVPVEPDKVEEANKGENTSLLHPVSLHGMDDPEREVLEWRFDSRLAFHHVA
RELHPEYFKNC

>4EMKB

MDSSPNEFLNKVIGKKVLIRLSSGVDYKGILSCLDGYMNLALERTEEYVNGKKTNVYGDAFIRGN
NVLYVSALDD

>4EMCC

QKKRFLPQSVLIKREDEIAFDDFHLDARKVLNDLSATSENPFSSSPNTKKIKSKGKTLEVVPKKK
NKKII

>4ELLA

GEFNTIQQLMMILNSASDQPSENLISYFNNCTVNPKESILKRVKDIGYIFKEKFAKAVGQGCVEI
GSQRYKLGVRLYYRVMESMLKSEEERLSIQNFSKLLNDNIFHMSLLACALEVVMATYSRSTSQNL
DSGTDLSFPWILNVLNLKAFDFYKVIESFIKAEGNLTREMIKHLERCEHRIMESFAWLSDSPLFD
LIKQSKDREGPTDHLESACPLNLPLQNNHTAADMYLEPVRAPKKKGSTTRVNSTANAETQATSAF
QTQKPLKSTSLSLFYKKVYRLAYLRLNTLCERLLSEHPELEHIIWTLFQHTLQNEYELMRDRHLD
QIMMCSMYGICKVKNIDLKFKIIVTAYKDLPHAVQETFKRVLIKEEEYDSIIVFYNSVFMQRLKT
NILQYASTRPPTLAPIPHIPR

>3VRCA

ADLSPEEQIETRQAGYAFMAWNMGKIKANLEGEYNADQVRAAANVVAAIANSGMGALYGPGTDKN
VGAVKTRAKPELFQNLEDVGKLARDLGTAANALAAAAATGEANAVKSAFADVGAACKACHQKYRA
D

>3VR8A

MLRAVRALICRIGARRTLSVSSSRLDVSTSNIAQYKVIDHAYDVVIIGAGGAGLRAAMGLGEAGF
KTAVVTKMFPTRSHTTAAQGGINAALGSMNPDDWKWHFYDTAKGSDWLGDQNAMHYLTRNAVEAV
TELENFGMPFSRTPEGKIYQRSFGGQSNNYGKGGVAKRTCCVADRTGHSMLHTLYGNSLRCHCTF
FIEYFALDLLMDKGRCVGVIALCLEDGTIHRFRSKRTIVATGGYGRAYFSCTTAHMNTGDGTALA
TRAGIALEDLEFIQFHPTGIYGVGCLITEGSRGEGGFLVNSEGERFMERYAPKAKDLASRDVVSR
AETIEIMEGRGVGPEKDHIYLQLHHLPAEQLHQRLPGISETAKIFAGVDVTKEPIPVIPTVHYNM
GGIPTNYKAQVIKYTKEGGDKIVPGLYACGECACHSVHGANRLGANSLLDAVVFGRACSINIKEE
LKPDEKIPELPEGAGEESIANLDAVRYANGDVPTAELRLTMQKTMQKHAGVFRRGDILAEGVKKM
MDLSKELKRLKTTDRSLIWNSDLTESLELQNLMLNATQTIVAAENRKESRGAHARDDFPKREDEY
DYSKPIEGQTKRPFEKHWRKHTLTKQDPRTGHITLDYRPVIDKTLDPAEVDWIPPIIRSY

>4EJOA

SNAMAYDDIVSSMVLELRRGTLVMLVLSQLREPAYGYALVKSLADHGIPIEANTLYPLMRRLESQ
GLLASEWDNGGSKPRKYYRTTDEGLRVLREVEAQWHVLCDGVGKLLETNGEDREHAER

>4EJ7A

MGSSHHHHHHSSGRENLYFQGMSHIQRETSCSRPRLNSNLDADLYGYRWARDNVGQSGATIYRLY
GKPNAPELFLKHGKGSVANDVTDEMVRLNWLTAFMPLPTIKHFIRTPDDAWLLTTAIPGKTAFQV
LEEYPDSGENIVDALAVFLRRLHSIPVCNCPFNSDRVFRLAQAQSRMNNGLVDASDFDDERNGWP
VEQVWKEMHKLLPFSPDSVVTHGDFSLDNLIFDEGKLIGCIDVGRVGIADRYQDLAILWNCLGEF
SPSLQKRLFQKYGIDNPDMNKLQFHLMLDEFF

>4EIYA

MKTIIALSYIFCLVFADYKDDDDGAPPIMGSSVYITVELAIAVLAILGNVLVCWAVWLNSNLQNV
TNYFVVSLAAADIAVGVLAIPFAITISTGFCAACHGCLFIACFVLVLTQSSIFSLLAIAIDRYIA
IRIPLRYNGLVTGTRAKGIIAICWVLSFAIGLTPMLGWNNCGQPKEGKNHSQGCGEGQVACLFED
VVPMNYMVYFNFFACVLVPLLLMLGVYLRIFLAARRQLADLEDNWETLNDNLKVIEKADNAAQVK
DALTKMRAAALDAQKATPPKLEDKSPDSPEMKDFRHGFDILVGQIDDALKLANEGKVKEAQAAAE
QLKTTRNAYIQKYLERARSTLQKEVHAAKSLAIIVGLFALCWLPLHIINCFTFFCPDCSHAPLWL
MYLAIVLSHTNSVVNPFIYAYRIREFRQTFRKIIRSHVLRQQEPFKAHHHHHHHHHH
>2LRKA
AEELEEVVMGLIINSGQARSLAYAALKQAKQGDFAAAKAMMDQSRMALNEAHLVQTKLIEGDAGE
GKMKVSLVLVEAQLHLMTSMLARELITELIELHEKLKA
>2LRKD
MFQQEVTITAPNGLHTRPAAQFVKEAKGFTSEITVTSNGKSASAKSLFKLQTLGLTQGTVVTISA
EGEDEQKAVEHLVKLMAELE
>4EIVA
MHHHHHHENLYFQGGTIYKQFTSRTLLNFFEVAALTDGETNESVAAVCKIAAKDPAIVGVSVRPA
FVRFIRQELVKSAPEVAGIKVCAAVNFPEGTGTPDTVSLEAVGALKDGADEIECLIDWRRMNENV
ADGESRIRLLVSEVKKVVGPKTLKVVLSGGELQGGDIISRAAVAALEGGADFLQTSSGLGATHAT
MFTVHLISIALREYMVRENERIRVEGINREGAAVRCIGIKIEVGDVHMAETADFLMQMIFENGPR
SIVRDKFRVGGGFNLLKELRDCYESWDSVGVSPDTSP
>4APMA
GSAMGSTPKDIWGRYMAKFDLAKSHGSGIYVDLGGTERVGATQHRMPTGKCPVMGKVINLGNNAD
FLNRISAENPQDRGLAFPDTAVAVTRNSNARNRAAAEKTEIILSPVSAADLVRWGYDGNDVANCA
EYAGNIIPASDTATKYRYPFVYDAKEEMCHILFTPMQYNRGSRYCDNDGSQDEGTSSLLCMEPMK
SGIDAHLYYGSSRVDKKWEENCPMYPVKDAIFGRGANGSCVAIESAFEEFTRDAEECSALMFENA
AADLEIDEEADNFDELKTLSDGLRNIKASKIAQALFSPIAKAGTSAKNSKGVGMNWANYDSNTGL
CRVIEETPNCLIIDAGSFAMTAVGSPLEQDAVPFPCDIVTNGYIEPRPRSRHRNTTPIFEVTTAL
SREALKCSKYVHEKYSESCGTYYYCSEEKPSSWAFWRNLDAAALVPR
>2LRJA
GSSISHSGNLYTAGQCTWYVYDKVGGEIGSTWGNANNWAAAAQGAGFTVNHTPSKGAILQSSEGP
FGHVAYVESVNSDGSVTISEMNYSGGPFSVSSRTISASEAGNYNYIHI
>4EFOA
GPLGSTSDILHRMVIHVFSLQQMTAHKIYIHSYNTATIFHELVYKQTKIISSNQELIYEGRRLVL
EPGRLAQHFPKTTEENPIFVVSLERPHRD
>4EFZA
GPGSMTVEGFFDPATCTISYLLFDSGSGECALIDSVLDYDPKSGRTRTASADQLIARVAALGARV
RWLLETHVHADHLSAAPYLKTRVGGEIAIGRHVTRVQDVFGKLFNAGPAFAHDGSQFDRLLDDGD
TLALGALSIRAMHTPGHTPACMTYVVTEAHAAHDARDAAAFVGDTLFMPDYGTARCDFPGGDARS
LYRSIRKVLSLPPATRLYMCHDYQPNGRAIQYASTVADELRENVHIREGVTEDDFVAMRTARDAT
LDMPVLMLPSVQVNMRAGRLPEPEDNGVRYLKIPLDAI
>4AOWA
MHHHHHHSSGVDLGTENLYFQSMMTEQMTLRGTLKGHNGWVTQIATTPQFPDMILSASRDKTIIM
WKLTRDETNYGIPQRALRGHSHFVSDVVISSDGQFALSGSWDGTLRLWDLTTGTTTRRFVGHTKD
VLSVAFSSDNRQIVSGSRDKTIKLWNTLGVCKYTVQDESHSEWVSCVRFSPNSSNPIIVSCGWDK
LVKVWNLANCKLKTNHIGHTGYLNTVTVSPDGSLCASGGKDGQAMLWDLNEGKHLYTLDGGDIIN

ALCFSPNRYWLCAATGPSIKIWDLEGKIIVDELKQEVISTSSKAEPPQCTSLAWSADGQTLFAGY
TDNLVRVWQVTIGTR
>4EFIA
MSSPDFSAGRELRTQGARIAGVVSCVPSKQVDNDYFVERFDASAVRDVVKMIGVNRRRWADAQTS
AGDLCRKAGEKLLAGLGWQADSIDALIFVSQTPNYRLPATAFVLQAELDLPASCLALDINLGCSG
YPQALWLGMNLIQTGAAKRVLLAVGDTISKMIDPTDRSTSLLFGDAGTMTALETSNGDAAAHFII
GADGKGARNLIVPSGGFKPYDAAADERMAGKSPECLFMDGGEIFNFTLNAVPKLVSRTLDIAGRD
KDSYDAFLFHQANLFMLKHLAKKAGLPAERVPVNIGEYGNTSCASIPLLITTELKDRLKEETLQL
GMFGFGVGYSWASAALAVGPLNIVDTIET
>4EEEA
MECVKTRSVNIHVPVKETSKVVLECRGDSYFRHFSYVYWIIGKNKTVDQLPPNSGYRERIYLFKK
PHRCENRPRADLILTNITDEMRNEKLTCVLIDPKDPLKESVILSKIWNCVYKI
>2LRDA
AMGKCSVLKKVACAAAIAGAVAACGGIDLPCVLAALKAAEGCASCFCEDHCHGVCKDLHLC
>4EEIA
MIKRYDVAEISKIWADENKYAKMLEVELAILEALEDRMVPKGTAAEIRARAQIRPERVDEIEKVT
KHDIIAFCTSIAEQFTAETGKFFHFGVTSSDIIDSALSLQIRDSMSYVIKDLEALCDSLLTKAEE
TKEIITMGRSHGMFAEPMSFGQKFLGAYVEFKRRLKDLKDFQKDGLTVQFSGAVGNYCILTTEDE
KKAADILGLPVEEVSTQVIPRDRIAKLISIHGLIASAIERLAVEIRHLHRSDVFEVYEGFSKGQK
GSSTMPHKKNPISTENLTGMARMLRSHVSIALENCVLWHERDISHSSAERFYLPDNFGIMVYALR
RMKNTIDNLVVQRDIIEDRVRSTSAYLSSFYLHFLVANTPFMREDCYKIVQQVAFDLKQGESFSK
KLQKVMHDEHNIILDIPEMDFEGIKKTYLKEIDHVFDRSVKARGENLY
>4EDFA
MFEIKKICCIGAGYVGGPTCSVIAHMCPEIRVTVVDVNESRINAWNSPTLPIYEPGLKEVVESCR
GKNLFFSTNIDDAIKEADLVFISVNTPTETYGMGKGRAADLKYIEACARRIVQNSNGYKIVTEKS
TVPVRAAESIRRIFDANTKPNLNLQVLSNPEFLAEGTAIKDLKNPDRVLIGGDETPEGQRAVQAL
CAVYEHWVPREKILTTNTWSSELSKLAANAFLAQRISSINSISALCEATGADVEEVATAIGMDQR
IGNKFLKASVGFGGSCFQKDVLNLVYLCEALNLPEVARYWQQVIDMNDYQRRRFASRIIDSLFNT
VTDKKIAILGFAFKKDTGDTRESSSIYISKYLMDEGAHLHIYDPKVPREQIVVDLSHPGVSEDDQ
VSRLVTISKDPYEACDGAHAVVICTEWDMFKELDYERIHKKMLKPAFIFDGRRVLDGLHNELQTI
GFQIETIGKKVSSKRIPYAPSGEIPKFSLQDPPNKKPKV
>3VQKA
MYYLGKELQKRSEELSRGFYELVYPPVDMYEEGGYLVVVADLAGFNKEKIKARVSGQNELIIEAE
REITEPGVKYLTQRPKYVRKVIRLPYNVAKDAEISGKYENGVLTIRIPIAGTSVIKIE
>4EBBA
PDPGFQERFFQQRLDHFNFERFGNKTFPQRFLVSDRFWVRGEGPIFFYTGNEGDVWAFANNSAFV
AELAAERGALLVFAEHRYYGKSLPFGAQSTQRGHTELLTVEQALADFAELLRALRRDLGAQDAPA
IAFGGSYGGMLSAYLRMKYPHLVAGALAASAPVLAVAGLGDSNQFFRDVTADFEGQSPKCTQGVR
EAFRQIKDLFLQGAYDTVRWEFGTCQPLSDEKDLTQLFMFARNAFTVLAMMDYPYPTDFLGPLPA
NPVKVGCDRLLSEAQRITGLRALAGLVYNASGSEHCYDIYRLYHSCADPTGCGTGPDARAWDYQA
CTEINLTFASNNVTDMFPDLPFTDELRQRYCLDTWGVWPRPDWLLTSFWGGDLRAASNIIFSNGN
LDPWAGGGIRRNLSASVIAVTIQGGAHHLDLRASHPEDPASVVEARKLEATIIGEWVKAARREQQ
PALRGGPRLSLENLYFQ
>4EAZA

DPNSMRSIASSKLWMLEFSAFLERQQDPDTYNKHLFVHISQSSPSYSDPYLETVDIRQIYDKFPE
KKGGLKELFERGPSNAFFLVKFWADLNTNIDDEGSAFYGVSSQYESPENMIITCSTKVCSFGKQV
VEKVETEYARYENGHYLYRIHRSPLCEYMINFIHKLKHLPEKYMMNSVLENFTILQVVTNRDTQE
TLLCIAYVFEVSASEHGAQHHIYRLVKE
>4EAGC
MESVAAESAPAPENEHSQETPESNSSVYTTFMKSHRCYDLIPTSSKLVVFDTSLQVKKAFFALVT
NGVRAAPLWDSKKQSFVGMLTITDFINILHRYYKSALVQIYELEEHKIETWREVYLQDSFKPLVC
ISPNASLFDAVSSLIRNKIHRLPVIDPESGNTLYILTHKRILKFLKLFITEFPKPEFMSKSLEEL
QIGTYANIAMVRTTTPVYVALGIFVQHRVSALPVVDEKGRVVDIYSKFDVINLAAEKTYNNLDVS
VTKALQHRSHYFEGVLKCYLHETLEAIINRLVEAEVHRLVVVDEHDVVKGIVSLSDILQALVLTG
GEKKP
>4E8JA
MKNNNVTEKELFYILDLFEHMKVTYWLDGGWGVDVLTGKQQREHRDIDIDFDAQHTQKVIQKLED
IGYKIEVHWMPSRMELKHEEYGYLDIHPINLNDDGSITQANPEGGNYVFQNDWFSETNYKDRKIP
CISKEAQLLFHSGYDLTETDHFDIKNLKSIT
>4E88A
MGGRALRVLVDMDGVLADVEGGLLRKFRARFPDQPFIALEDRRGYKACEQYGRLRPGLSEKARSI
AESKNFFFELEPLPGAVEAVKEMASLQNTDVFICTSPHKMFKYCPYEKYAWVEKYFGPDFLEQIV
LTRDKTVVSADLLIDDRPDITGAEPTPSWEHVLFTACHNQHLQLQPPRRRLHSWADDWKAILDSK
RPCGSLEHHHHHH
>4AN6A
DYTVHDTDGKPVLNNAGQYYILPAKQGKGGGLGLSNDDDGNCPLTVSQTPIDLPIGLPVRFSSRA
RISHITTALSLNIEFTIAPACAPKPARWRIFNEQSSEKGYTPVKISDDFSSAAPFQIKKFEEDYK
LVYCSKSESGERKCVDLGIKIDDEKNRRLVLKEGDPFKVKFKKVDEESSEEWSIV
>4E4TA
MAHHHHHHMGTLEAQTQGPGSMTATPDSVSPILPGAWLGMVGGGQLGRMFCFAAQSMGYRVAVLD
PDPASPAGAVADRHLRAAYDDEAALAELAGLCEAVSTEFENVPAASLDFLARTTFVAPAGRCVAV
AQDRIAEKRFIEASGVPVAPHVVIESAAALAALDDAALDAVLPGILKTARLGYDGKGQVRVSTAR
EARDAHAALGGVPCVLEKRLPLKYEVSALIARGADGRSAAFPLAQNVHHNGILALTIVPAPAADT
ARVEEAQQAAVRIADTLGYVGVLCVEFFVLEDGSFVANEMAPRPHNSGHYTVDACATSQFEQQVR
AMTRMPLGNPRQHSPAAMLNILGDVWFPNGAAAGAVTPPWDTVAAMPAAHLHLYGKEEARVGRKM
GHVNFTAEMRDDAVAAATACAQLLRVPLD
>4E51A
MAHHHHHHMGTLEAQTQGPGSMTEQKRKLEKLTGVKGMNDILPQDAGLWEFFEATVKSLLRAYGY
QNIRTPIVEHTPLFTRGIGEVTDIVEKEMYSFVDALNGENLTLRPENTAAVVRAAIEHNMLYDGP
KRLWYIGPMFRHERPQRGRYRQFHQVGVEALGFAGPDADAEIVMMCQRLWEDLGLTGIKLEINSL
GLAEERAAHRVELIKYLEQHADKLDDDAQRRLYTNPLRVLDTKNPALQEIVRNAPKLIDFLGDVS
RAHFEGLQRLLKANNVPFTINPRLVRGLDYYNLTVFEWVTDKLGAQGTVAAGGRYDPLIEQLGGK
PTAACGWAMGIERILELLKEEHLVPEQEGVDVYVVHQGDAAREQAFIVAERLRDTGLDVILHCSA
DGAGASFKSQMKRADASGAAFAVIFGEDEVTNGTASVKPLRGTGDDGEKSVQQSVPVESLTEFLI
NAMVATAEDGDD
>2LQOA
MVTAALTIYTTSWCGYCLRLKTALTANRIAYDEVDIEHNRAAAEFVGSVNGGNRTVPTVKFADGS
TLTNPSADEVKAKLVKIAGLEHHHHHH

>4E1YA
GSHPFDQAVVKDPTASYVDVKARRTFLQSGQLDDRLKAALPKEYDCTTEATPNPQQGEMVIPRRY
LSGNHGPVNPDYEPVVTLYRDFEKISATLGNLYVATGKPVYATCLLNMLDKWAKADALLNYDPKS
QSWYQVEWSAATAAFALSTMMAEPNVDTAQRERVVKWLNRVARHQTSFPGGDTSCCNNASYWRGQ
EATIIGVISKDDELFRWGLGRYVQAMGLINEDGSFVHEMTRHEQSLHYQNYAMLPLTMIAETASR
QGIDLYAYKENGRDIHSARKFVFAAVKNPDLIKKYASEPQDTRAFKPGRGDLNWIEYQRARFGFA
DELGFMTVPIFDPRTGGSGTLLAYKPQG
>4E1JA
MHHHHHHSSGVDLGTENLYFQSMMGGYILAIDQGTTSTRAIVFDGNQKIAGVGQKEFKQHFPKSG
WVEHDPEEIWQTVVSTVKEAIEKSGITANDIAAIGITNQRETVVVWDRETGKPIHNAIVWQDRRT
AAFCDKLKKKGLEKTFVKKTGLLLDPYFSGTKLNWLLSNVKGAQVRAAKGELCFGTIDTFLIWRL
TGGECFCTDATNASRTLLYNIAENAWDDELTEVLRVPKEMLPEVKDCAADFGVTDPSLFGAAIPI
LGVAGDQQAATIGQACFKPGMLKSTYGTGCFALLNTGKDMVRSKNRLLTTIAYRLDGETTYALEG
SIFVAGAAVQWLRDGLKVIKAAPDTGSLAESADPSQEVYLVPAFTGLGAPHWDPDARGAIFGMTR
NTGPAEFARAALEAVCYQTRDLLEAMHKDWRRNGNDTVLRVDGGMVASDWTMQRLSDLLDAPVDR
PVILETTALGVAWLAGSRAGVWPNQEAFAKSWARDRRFEPHMDEATRKVKLKGWRSAVKRTLIAA
>4E0VA
MNVFFMFSKPGKLADDRNPLEECFRETDYEEFLEIAKNGLSTTSNPKRVVIVGAGMSGLSAAYVL
ANAGHQVTVLEASERAGGQVKTYRNEKEGWYANLGPMRLPEKHRIVREYIRKFGLQLNEFSQENE
NAWYFIKNIRKRVGEVNKDPGVLDYPVKPSEVGKSAGQLYEESLQKAVEELRRTNCSYMLNKYDT
YSTKEYLLKEGNLSPGAVDMIGDLLNEDSGYYVSFIESLKHDDIFAYEKRFDEIVGGMDKLPTSM
YQAIQEKVHLNARVIKIQQDVKEVTVTYQTSEKETLSVTADYVIVCTTSRAARRIKFEPPLPPKK
AHALRSVHYRSGTKIFLTCTKKFWEDDGIHGGKSTTDLPSRFIYYPNHNFPNGVGVIIAYGIGDD
ANYFEALDFEDCGDIVINDLSLIHQLPKEEIQAICRPSMIQRWSLDKYAMGGITTFTPYQFQHFS
EALTAPVDRIYFAGEYTAQAHGWIASTIKSGPEGLDVNRASE
>4E0IA
MKAIDKMTDNPPQEGLSGRKIIYDEDGKPSRSCNTLLDFQYVTGKISNGLKNLSSNGKLAGTGAL
TGEASELMPGSRTYRKVDPPDVEQLGRSSWTLLHSVAASYPAQPTDQQKGEMKQFLNIFSHIYPC
NWSAKDFEKYIRENAPQVESREELGRWMCEAHNKVNKKLRKPKFDCNFWEKRWKDGWDE
>4DYLA
SMGFSSELCSPQGHGVLQQMQEAELRLLEGMRKWMAQRVKSDREYAGLLHHMSLQDSGGQSRAIS
PDSPISQSWAEITSQTEGLSRLLRQHAEDLNSGPLSKLSLLIRERQQLRKTYSEQWQQLQQELTK
THSQDIEKLKSQYRALARDSAQAKRKYQEASKDKDRDKAKDKYVRSLWKLFAHHNRYVLGVRAAQ
LHHQHHHQLLLPGLLRSLQDLHEEMACILKEILQEYLEISSLVQDEVVAIHREMAAAAARIQPEA
EYQGFLRQYGSAPDVPPCVTFDESLLEEGEPLEPGELQLNELTVESVQHTLTSVTDELAVATEMV
FRRQEMVTQLQQELRNEEENTHPRERVQLLGKRQVLQEALQGLQVALCSQAKLQAQQELLQTKLE
HLGPGEPPPVLLLQDD
>4DXRA
GPGGSGGVTEEQVHHIVKQALQRYSEDRIGLADYALESGGASVISTRCSETYETKTALLSLFGIP
LWYHSQSPRVILQPDVHPGNCWAFQGPQGFAVVRLSARIRPTAVTLEHVPKALSPNSTISSAPKD
FAIFGFDEDLQQEGTLLGKFTYDQDGEPIQTFHFQAPTMATYQVVELRILTNWGHPEYTCIYRFR
VHGEPAH
>3VP7A
INIFNATFKISHSGPFATINGLRLGSIPESVVPWKEINAALGQLILLLATINKNLKINLVDYELQ

PMGSFSKIKKRMVNSVEYNNSTTNAPGDWLILPVYYDENFNLGRIFRKETKFDKSLETTLEIISE
ITRQLSTIASSYSSQTLTTSQDESSMNNANDVENSTSILELPYIMNKDKINGLSVKLHGSSPNLE
WTTAMKFLLTNVKWLLAFSSNLLSK
>4DXDA
MHHHHHHLEFEQGFNHLATLKVIGVGGGGNNAVNRMIDHGMNNVEFIAINTDGQALNLSKAESKI
QIGEKLTRGLGAGANPEIGKKAAEESREQIEDAIQGADMVFVTSGMGGGTGTGAAPVVAKIAKEM
GALTVGVVTRPFSFEGRKRQTQAAAGVEAMKAAVDTLIVIPNDRLLDIVDKSTPMMEAFKEADNV
LRQGVQGISDLIAVSGEVNLDFADVKTIMSNQGSALMGIGVSSGENRAVEAAKKAISSPLLETSI
VGAQGVLMNITGGESLSLFEAQEAADIVQDAADEDVNMIFGTVINPELQDEIVVTVIATGFDDKP
TSHGRKSGSTGFGTSVNTSSNATSKDESFTSNSSNAQATDSVSERTHTTKEDDIPSFIRNREERR
SRRTRR
>4DX1A
MFNRPILFDIVSRGSTADLDGLLPFLLTHKKRLTDEEFREPSTGKTCLPKALLNLSNGRNDTIPV
LLDIAERTGNMREFINSPFRDIYYRGQTALHIAIERRCKHYVELLVAQGADVHAQARGRFFQPKD
EGGYFYFGELPLSLAACTNQPHIVNYLTENPHKKADMRRQDSRGNTVLHALVAIADNTRENTKFV
TKMYDLLLLKCARLFPDSNLEAVLNNDGLSPLMMAAKTGKIGIFQHIIRREVTDEAAAHHHHHH
>4AKKA
MRGSHHHHHHTDPHASSVPGRGSIEGRMNNMAGNTPEVVDWFARARRLQKQQLHQLAQQGTLAGQ
ISALVHMLQCERGASNIWLCSGGRLYAAECRAGAALVDEQLTRFYAALEPARDAASSALCWRIAC
AVWYLPQLAALRKRVRDREIAAEEATGQFSRIIRHLLNIVPQLNDSIDDPQIAGRMVALYSFMQG
KELAGQERALGALGFARGQFSDELRQQLVDRIDGQQPCFDSFQALAQPPQTALFAEQCQASLEIE
QLRRVACTRQPPADEGETALRWFCAQTQRLEQLRGVEELLIVDLLNAADALLEGEEPEAQLPPAD
WQEDSIALRLDKQLLPLVRQQAHELQQLSGQLASLKDALEERKLIEKAKSVLMTYQGMQEEQAWQ
ALRKMAMDKNQRMVEIARALLTVKALWRVTPKE
>4DW0A
GSSKKVGTLNRFTQALVIAYVIGYVFVYNKGYQDTDTVLSSVTTKVKGIALTKTSELGERIWDVA
DYIIPPQEDGSFFVLTNMIITTNQTQSKCAENPTPASTCTSHRDCKRGFNDARGDGVRTGRCVSY
SASVKTCEVLSWCPLEKIVDPPNPPLLADAERFTVLIKNNIRYPKFNFNKRNILPNINSSYLTHC
VFSRKTDPDCPIFRLGDIVGEAEEDFQIMAVRGGVMGVQIRWDCDLDMPQSWCVPRYTFRRLDNK
DPDNNVAPGYNFRFAKYYKNSDGTETRTLIKGYGIRFDVMVFGQAGKFNIIPTLLNIGAGLALLG
LVNVICDWIVLTFMK
>4DT4A
MGSSHHHHHHSSGLVPRGSHMSESVQSNSAVLVHFTLKLDDGTTAESTRNNGKPALFRLGDASLS
EGLEQHLLGLKVGDKTTFSLEPDAAFGVPSPDLIQYFSRREFMDAGEPEIGAIMLFTAMDGSEMP
GVIREINGDSITVDFNHPLAGQTVHFDIEVLEIDPALEA
>2LPIA
GSGNSHTTPWTNPGLAENFMNSFMQGLSSMPGFTASQLDDMSTIAQSMVQSIQSLAAQGRTSPNK
LQALNMRFASSMAEIAASEEGGGSLSTKTSSIASAMSNAFLQTTGVVNQPFINEITQLVSMFAQA
GMNDVSA
>4DPPA
MHHHHHHGLPIPNPLLGLDSTENLYFQGIDPFTAAVVPNFHLPMRSLEVKNRTNTDDIKALRVIT
AIKTPYLPDGRFDLEAYDDLVNIQIQNGAEGVIVGGTTGEGQLMSWDEHIMLIGHTVNCFGGSIK
VIGNTGSNSTREAIHATEQGFAVGMHAALHINPYYGKTSIEGLIAHFQSVLHMGPTIIYNVPGRT
GQDIPPRAIFKLSQNPNLAGVKECVGNKRVEEYTENGVVVWSGNDDECHDSRWDYGATGVISVTS

NLVPGLMRKLMFEGRNSSLNSKLLPLMAWLFHEPNPIGINTALAQLGVSRPVFRLPYVPLPLSKR
LEFVKLVKEIGREHFVGEKDVQALDDDDFILIGRY
>4DOTA
MRAPIPEPKPGDLIEIFRPFYRHWAIYVGDGYVVHLAPPSEVAGAGAASVMSALTDKAIVKKELL
YDVAGSDKYQVNNKHDDKYSPLPCSKIIQRAEELVGQEVLYKLTSENCEHFVNELRYGVARSDQV
RDLEHHHHHH
>4DOJA
LENPTNLEGKLADAEEEIILEGEDTQASLNWSVIVPALVIVLATVVWGIGFKDSFTNFASSALSA
VVDNLGWAFILFGTVFVFFIVVIAASKFGTIRLGRIDEAPEFRTVSWISMMFAAGMGIDLMFYGT
TEPLTFYRNGVPGHDEHNVGVAMSTTMFHWTLHPWAIYAIVGLAIAYSTFRVGRKQLLSSAFVPL
IGEKGAEGWLGKLIDILAIIATVFGTACSLGLGALQIGAGLSAANIIEDPSDWTIVGIVSVLTLA
FIFSAISGVGKGIQYLSNANMVLAALLAIFVFVVGPTVSILNLLPGSIGNYLSNFFQMAGRTAMS
ADGTAGEWLGSWTIFYWAWWISWSPFVGMFLARISRGRSIREFILGVLLVPAGVSTVWFSIFGGT
AIVFEQNGESIWGDGAAEEQLFGLLHALPGGQIMGIIAMILLGTFFITSADSASTVMGTMSQHGQ
LEANKWVTAAWGVATAAIGLTLLLSGGDNALSNLQNVTIVAATPFLFVVIGLMFALVKDLSNDVI
YLEYREQQRFNARLARERRVHNEHRKRELAAKRRRERKASGAGKRR
>4AIEA
MASASWWKNAVVYQVYPKSFQDSNGDGIGDLQGIISRLDYLEKLGIDAIWLSPVYQSPGVDNGYD
ISDYEAIDPQYGTMADMDELISKAKEHHIKIVMDLVVNHTSDQHKWFVEAKKGKDNQYRDYYIWR
DPVDEHEPNDLKSAFSGSAWKYDERSGQYYLHFFADQQPDLNWQNTELRQKIYNMMNFWLDKGIG
GFRMDVIELIGKDPDKNIRENGPMLHPYLQEMNKATFGKRDVMTVGETWNATPKIAEEYSDPDRH
ELSMVFQFENQSLDQQPGKEKWDLKPLDLGELKKVLVKWQTKIDFDHAWNSLFWENHDIPRVISR
WGNDQEYRVQCAKMFAIILHMMHGTPYIFNGEEIGMTNCPVKNIDEVEDIESINMYNERLAEGYD
EEELIHAINVKGRDNARRPMQWNDEKNAGFSEVDPWLSVNPNYKDINVENALADPNSIFYTYQKL
IKLRHENPIVVDGDFSLVSNTQDAVLAYYRILNDKKWLVVANLSNEEQNFVSNDQIETILSNYPE
RNNVQNITLKPYEAFISKVIELEHHHHHH
>4DMUB
APDCSQPLDVILLLDGSSSFPASYFDEMKSFAKAFISKANIGPRLTQVSVLQYGSITTIDVPWNV
VPEKAHLLSLVDVMQREGGPSQIGDALGFAVRYLTSEMHGARPGASKAVVILVTDVSVDSVDAAA
DAARSNRVTVFPIGIGDRYDAAQLRILAGPAGDSNVVKLQRIEDLPTMVTLGNSFLHKLCSG
>4DM3A
MSGADRSPNAGAAPDSAPGQAAVASAYQRFEPRAYLRNNYAPPRGDLCNPNGVGPWKLRCLAQTF
ATGEVSGRTLIDIGSGPTVYQLLSACSHFEDITMTDFLEVNRQELGRWLQEEPGAFNWSMYSQHA
CLIEGKGECWQDKERQLRARVKRVLPIDVHQPQPLGAGSPAPLPADALVSAFCLEAVSPDLASFQ
RALDHITTLLRPGGHLLLIGALEESWYLAGEARLTVVPVSEEEVREALVRSGYKVRDLRTYIMPA
HLQTGVDDVKGVFFAWAQKVGLEHHHHHH
>4DLFA
MGALRIDSHQHFWRYRAADYPWIGAGMGVLARDYLPDALHPLMHAQALGASIAVQARAGRDETAF
LLELACDEARIAAVVGWEDLRAPQLAERVAEWRGTKLRGFRHQLQDEADVRAFVDDADFARGVAW
LQANDYVYDVLVFERQLPDVQAFCARHDAHWLVLDHAGKPALAEFDRDDTALARWRAALRELAAL
PHVVCKLSGLVTEADWRRGLRASDLRHIEQCLDAALDAFGPQRLMFGSDWPVCLLAASYDEVASL
VERWAESRLSAAERSALWGGTAARCYALPEPADARLAENLYFQ
>4DJSA
LATRAIPELTKLLNDEDQVVVNKAAVMVHQLSKKEASRHAIMRSPQMVSAIVRTMQNTNDVETAR

CTAGTLHNLSHHREGLLAIFKSGGIPALVKMLGSPVDSVLFYAITTLHNLLLHQEGAKMAVRLAG
GLQKMVALLNKTNVKFLAITTDCLQILAYGNQESKLIILASGGPQALVNIMRTYTYEKLLWTTSR
VLKVLSVCSSNKPAIVEAGGMQALGLHLTDPSQRLVQNCLWTLRNLSDAATKQEGMEGLLGTLVQ
LLGSDDINVVTCAAGILSNLTCNNYKNKMMVCQVGGIEALVRTVLRAGDREDITEPAICALRHLT
SRHQEAEMAQNAVRLHYGLPVVVKLLHPPSHWPLIKATVGLIRNLALCPANHAPLREQGAIPRLV
QLLVRAHQDTQRRTSMGGTQQQFVEGVRMEEIVEGCTGALHILARDVHNRIVIRGLNTIPLFVQL
LYSPIENIQRVAAGVLCELAQDKEAAEAIEAEGATAPLTELLHSRNEGVATYAAAVLFRMSED
>4DJTA
GPGSMERRELTYKICLIGDGGVGKTTYINRVLDGRFEKNYNATVGAVNHPVTFLDDQGNVIKFNV
WDTAGQEKKAVLKDVYYIGASGAILFFDVTSRITCQNLARWVKEFQAVVGNEAPIVVCANKIDIK
NRQKISKKLVMEVLKGKNYEYFEISAKTAHNFGLPFLHLARIFTGRPDLIFVSNVNLEPTEVNYD
YHSPEESKYIDYMEQASKMAPEE
>4DJBA
MGSSHHHHHHSQDPMIRCLRLKVEGALEQIFTMAGLNIRDLLRDILRRWRDENYLGMVEGAGMFI
EEIHPEGFSLYVHLDVRAVSLLEAIVQHLTEAIISSLAVEFDHATGGERVHLIDLHFEVLDNLLE
>4DIXA
GPSSSKSEENISLVYEIDGTEALGSCLRVRPCSNDAPDLSKCTIQWYRSSSDGSKKELISGATKS
VYAPEPFDVGRVLHADIIYDGHSLSLSTVGKIDPAAGLGSYVEALVRKHDVDFNVVVTQMSGEDH
TSESIHLFHVGKMRIKLCKGKTVIAKEYYSSAMQLCGVRGGGNAAAQALYWQAKKGVSFVIAFES
ERERNAAIMLARRFACDCNVTLAGPEDRTETGQSP
>4DIPA
YFQSMGALIPEPEVKIEVLQKPFICHRKTKGGDLMLVHYEGYLEKDGSLFHSTHKHNNGQPIWFT
LGILEALKGWDQGLKGMCVGEKRKLIIPPALGYGKEGKGKIPPESTLIFNIDLLEIRNGP
>4DIQA
LRRRYTMASGPQVDNTGGEPAWDSPLRRVLAELNRIPSSRRRAARLFEWLIAPMPPDHFYRRLWE
REAVLVRRQDHTYYQGLFSTADLDSMLRNEEVQFGQHLDAARYINGRRETLNPPGRALPAAAWSL
YQAGCSLRLLCPQAFSTTVWQFLAVLQEQFGSMAGSNVYLTPPNSQGFAPHYDDIEAFVLQLEGR
KLWRVYRPRAPTEELALTSSPNFSQDDLGEPVLQTVLEPGDLLYFPRGFIHQAECQDGVHSLHLT
LSTYQRNTWGDFLEAILPLAVQAAMEENVEFRRGLPRDFMDYMGAQHSDSKDPRRTAFMEKVRVL
VARLGHFAPVDAVADQRAKDFIHDSLPPVLTDRERALSVYGLPIRWEAGEPVNVGAQLTTETEVH
MLQDGIARLVGEGGHLFLYYTVENSRVYHLEEPKCLEIYPQQADAMELLLGSYPEFVRVGDLPCD
SVEDQLSLATTLYDKGLLLTKMPLALNAENLYFQ
>2LOYA
MLIYKDIFTDDELSSDSFPMKLVDDLVYEFKGKHVVRKEGEIVLAGSNPSAEEGAEDDGSDEHVE
RGIDIVLNHKLVEMNCYEDASMFKAYIKKFMKNVIDHMEKNNRDKADVDAFKKKIQGWVVSLLAK
DRFKNLAFFIGERAAEGAENGQVAIIEYRDVDGTEVPTLMLVKEAIIEEKCLEHHHHHH
>2LORA
MVNLGLSRVDDAVAAKHPGLGEYAACQSHAFMKGVFTFVTGTGMAFGLQMFIQRKFPYPLQWSLL
VAVVAGSVVSYGVTRVESEKCNNLWLFLETGQLPKDRSTDQRS
>2LONA
MSANRRWWVPPDDEDCVSEKLLRKTRESPLVPIGLGGCLVVAAYRIYRLRSRGSTKMSIHLIHTR
VAAQACAVGAIMLGAVYTMYSDYVKRMAQDAGEK
>4DG8A
GHMDSFFRKKAIVRMSQNSLLDLYAHPTVVARFSEMAALHPHREAIRDRFGSVDYRQLLDSAEQL

SDYLLEHYPQPGVCLGVYGEYSRESITCLLAILLSGHHYLYIDLKQPAAWNAELCRQVDCRLILD
CSTTPTPANGLPCVPVRHLPAAPASVARPCFAADQIAYINFSSGTTGRPKAIACTHAGITRLCLG
QSFLAFAPQMRFLVNSPLSFDAATLEIWGALLNGGCCVLNDLGPLDPGVLRQLIGERGADSAWLT
ASLFNTLVDLDPDCLGGLRQLLTGGDILSVPHVRRALLRHPRLHLVNGYGPTENTTFTCCHVVTD
DDLEEDDIPIGKAIAGTAVLLLDEHGQEIAEPDRAGEIVAFGAGLAQGYRNDAARTRASFVELPY
RGRLLRAYRTGDRARYDEQGRLRFIGRGDGQVKLNGYRLDLPALEQRFRRQPGILDCALLVRERN
GVKQLLCAWTGKADASPQALLRQLPTWQRPHACVRVEALPLTAHGKLDRAALLRRLEEPLERCAS
ALDPDQRGCAQLWSELLGCEVGAADQDFFLCGGNSLLALQLVALCQSAGAGANLGLADLQANSRL
DQFSRLLRSHGLAPERLLERAATPEQPLVLSRSAA
>2LOEA
EKVKGCDFTTSESTIFSKGYSINEISNKSSNNQQDIVCTVKAHANDLIGFKCPSNYSVEPHDCFV
SAFNLSGKNENLENKLKLTNIIMDHYNNTFYSRLPSLISDNWKFFCVCSKDNEKKLVFTVEA
>4DEPB
EPLEADKCKEREEKIILVSSANEIDVRPCPLNPNEHKGTITWYKDDSKTPVSTEQASRIHQHKEK
LWFVPAKVEDSGHYYCVVRNSSYCLRIKISAKFVENEPNLCYNAQAIFKQKLPVAGDGGLVCPYM
EFFKNENNELPKLQWYKDCKPLLLDNIHFSGVKDRLIVMNVAEKHRGNYTCHASYTYLGKQYPIT
RVIEFITLEENKPTRPVIVSPANETMEVDLGSQIQLICNVTGQLSDIAYWKWNGSVIDEDDPVLG
EDYYSVENPANKRRSTLITVLNISEIESRFYKHPFTCFAKNTHGIDAAYIQLIYPVTNFQK
>2LOBA
HHHHHHHHHHSSGHIEGRHMENLYFQGIRKVLLLKEDHEGLGISITGGKEHGVPILISEIHPGQP
ADRCGGLHVGDAILAVNGVNLRDTKHKEAVTILSQQRGEIEFEVVYV
>4AFIA
GSPFYIKSSPSPQKRYQDTPGVEHIPVVQIDLSVPLKVPGLPMSDQYVKLEEAMAILFAVVARGT
TILAKHAWCGGNFLEVTEQILAKIPSENNKLTYSHGNYLFHYICQDRIVYLCITDDDFERSRAFS
FLNEVKKRFQTTYGSRAQTALPYAMNSEFSSVLAAQLKHHSEN
>4DCNC
GSRTVDLELELQIELLRETKRKYESVLQLGRALTAHLYSLLQTQHALGDAFADLSQKSPELQEEF
GYNAETQKLLCKNGETLLGAVNFFVSSINTLVTKTMEDTLMTVKQYEAARLEYDAYRTDLEELSL
GPRDAGTRGRLESAQATFQAHRDKYEKLRGDVAIKLKFLEENKIKVMHKQLLLFHNAVSAYFAGN
QKQ
>3VO1A
MVSTTETAEAEPVKKLEKVSKKQEEGLVTNKYKPKEPYVGRCLLNTRITGDQAPGETWHMVFSTE
GEVPYREGQSIGVIADGEDKNGKPHKLRLYSIASSALGDFGDSKTVSLCVKRLVYTNDQGEVVKG
VCSNFLCDLKPGAEVKITGPVGKEMLMPKDPNATIIMLATGTGIAPFRSFLWKMFFEEHEDYKYT
GLAWLFLGVPTSDTLLYKEELEKMKEMAPDNFRLDFAVSREQTNAAGEKMYIQTRMAEYKEELWE
LLKKDNTYVYMCGLKGMEKGIDDIMLDLAAKDGINWLDYKKQLKKSEQWNVEVY
>4DCXA
AAPDEITTAWPVNVGPLNPHLYTPNQMFAQSMVYEPLVKYQADGSVIPWLAKSWTHSEDGKTWTF
TLRDDVKFSNGEPFDAEAAAENFRAVLDNRQRHAWLELANQIVDVKALSKTELQITLKSAYYPFL
QELALPRPFRFIAPSQFKNHETMNGIKAPIGTGPWILQESKLNQYDVFVRNENYWGEKPAIKKIT
FNVIPDPTTRAVAFETGDIDLLYGNEGLLPLDTFARFSQNPAYHTQLSQPIETVMLALNTAKAPT
NELAVREALNYAVNKKSLIDNALYGTQQVADTLFAPSVPYANLGLKPSQYDPQKAKALLEKAGWT
LPAGKDIREKNGQPLRIELSFIGTDALSKSMAEIIQADMRQIGADVSLIGEEESSIYARQRDGRF
GMIFHRTWGAPYDPHAFLSSMRVPSHADFQAQQGLADKPLIDKEIGEVLATHDETQRQALYRDIL

TRLHDEAVYLPISYISMMVVSKPELGNIPYAPIATEIPFEQIKPVKP
>4DCKC
MALLRKSYSEPQLKGIVTKLYSRQGYHLQLQADGTIDGTKDEDSTYTLFNLIPVGLRVVAIQGVQ
TKLYLAMNSEGYLYTSELFTPECKFKESVFENYYVTYSSMIYRQQQSGRGWYLGLNKEGEIMKGN
HVKKNKPAAHFLPKPLKVAMYKEPSLHDLTEFSRSGSGTPTKSRSVSGVLNGGKSMSHNEST
>4DBLC
MSIVMQLQDVAESTRLGPLSGEVRAGEILHLVGPNGAGKSTLLARMAGMTSGKGSIQFAGQPLEA
WSATKLALHRAYLSQQQTPPFATPVWHYLTLHQHDKTRTELLNDVAGALALDDKLGRSTNQLSGG
EWQRVRLAAVVLQITPQANPAGQLLLLDQPMNSLDVAQQSALDKILSALSQQGLAIVMSSHDLNH
TLRHAHRAWLLKGGKMLASGRREEVLTPPNLAQAYGMNFRRLDIEGHRMLISTI
>4D97A
MRGSHHHHHHGMASMPLHHLTRFPRLEFIGAPTPLEYLPRLSDYLGREIYIKRDDVTPIAMGGNK
LRKLEFLVADALREGADTLITAGAIQSNHVRQTAAVAAKLGLHCVALLENPIGTTAENYLTNGNR
LLLDLFNTQIEMCDALTDPDAQLQTLATRIEAQGFRPYVIPVGGSSALGAMGYVESALEIAQQCE
EVVGLSSVVVASGSAGTHAGLAVGLEHLMPDVELIGVTVSRSVAEQKPKVIALQQAIAGQLALTA
TADIHLWDDYFAPGYGVPNDAGMEAVKLLASLEGVLLDPVYTGKAMAGLIDGISQKRFNDDGPIL
FIHTGGAPALFAYHPHVTYPE
>4D8QH
MSLRLPQNPNAGLFKQGYNSYSNADGQIIKSIAAIRELHQMCLTSMGPCGRNKIIVNHLGKIIIT
NDAATMLRELDIVHPAVKVLVMATEQQKIDMGDGTNLVMILAGELLNVSEKLISMGLSAVEIIQG
YNMARKFTLKELDEMVVGEITDKNDKNELLKMIKPVISSKKYGSEDILSELVSEAVSHVLPVAQQ
AGEIPYFNVDSIRVVKIMGGSLSNSTVIKGMVFNREPEGHVKSLSEDKKHKVAVFTCPLDIANTE
TKGTVLLHNAQEMLDFSKGEEKQIDAMMKEIADMGVECIVAGAGVGELALHYLNRYGILVLKVPS
KFELRRLCRVCGATPLPRLGAPTPEELGLVETVKTMEIGGDRVTVFKQEQGEISRTSTIILRGAT
QNNLDDIERAIDDGVAAVKGLMKPSGGKLLPGAGATEIELISRITKYGERTPGLLQLAIKQFAVA
FEVVPRTLAETAGLDVNEVLPNLYAAHNVTEPGAVKTDHLYKGVDIDGESDEGVKDIREENIYDM
LATKKFAINVATEAATTVLSIDQIIMAKKAGGPRAPQGPRPGNWDQED
>4D87A
MSNKYRVRKNVLHLTDTEKRDFVRTVLILKEKGIYDRYIAWHGAAGKFHTPPGSDRNAAHMSSAF
LPWHREYLLRFERDLQSINPEVTLPYWEWETDAQMQDPSQSQIWSADFMGGNGNPIKDFIVDTGP
FAAGRWTTIDEQGNPSGGLKRNFGATKEAPTLPTRDDVLNALKITQYDTPPWDMTSQNSFRNQLE
GFINGPQLHNRVHRWVGGQMGVVPTAPNDPVFFLHHANVDRIWAVWQIIHRNQNYQPMKNGPFGQ
NFRDPMYPWNTTPEDVMNHRKLGYVYDIELRKSKRSSHHHHHH
>4D8KA
GGSNPPASPLQDNLVIALHSYEPSHDGDLGFEKGEQLRILEQSGEWWKAQSLTTGQEGFIPFNFV
AKANSLEPEPWFFKNLSRKDAERQLLAPGNTHGSFLIRESESTAGSFSLSVRDFDQNQGEVVKHY
KIRNLDNGGFYISPRITFPGLHELVRHYTNASDGLCTRLSRPCQT
>3VF0B
HMLDPEEIRKRLEHTERQFRNRRKILIRGLPGDVTNQEVHDLLSDYELKYCFVDKYKGTAFVTLL
NGEQAEAAINAFHQSRLRERELSVQLQPTDALLCVANLPPSLTQQQFEELVRPFGSLERCFLVYS
ERTGQSKGYGFAEYMKKDSAARAKSDLLGKPLGPRTLYVHWTDAGQLTPALLHSRCLCVDRLPPG
FNDVDALCRALSAVHSPTFCQLACGQDGQLKGFAVLEYETAEMAEEAQQQADGLSLGGSHLRVSF
CAPGPPGRSMLAALIAAQATALNRG
>3VFDA

ESGAVPKRKDPLTHTSNSLPRSKTVMKTGSAGLSGHHRAPSYSGLSMVSGVKQGSGPAPTTHKGT
PKTNRTNKPSTPTTATRKKKDLKNFRNVDSNLANLIMNEIVDNGTAVKFDDIAGQDLAKQALQEI
VILPSLRPELFTGLRAPARGLLLFGPPGNGKTMLAKAVAAESNATFFNISAASLTSKYVGEGEKL
VRALFAVARELQPSIIFIDQVDSLLCERREGEHDASRRLKTEFLIEFDGVQSAGDDRVLVMGATN
RPQELDEAVLRRFIKRVYVSLPNEETRLLLLKNLLCKQGSPLTQKELAQLARMTDGYSGSDLTAL
AKDAALGPIRELKPEQVKNMSASEMRNIRLSDFTESLKKIKRSVSPQTLEAYIRWNKDFGDTTV
>2LO0A
SVDVAVSAGAGERASAEQKESYEPPKPAVGPSGESVVATEAFWDDLQGFLEQRLKDYDEANKLRV
LFKEAWRSSF
>3VE0I
YPYDVPDYAIEGRGARSMPLGVVTNSTLEVTEIDQLVCKDHLASTDQLKSVGLNLEGSGVSTDIP
SATKRWGFRSGVPPKVVSYEAGEWAENCYNLEIKKPDGSECLPPPPDGVRGFPRCRYVHKAQGTG
PCPGDYAFHKDGAFFLYDRLASTVIYRGVNFAEGVIAFLILAKPKETFLQSPPIREAVNYTENTS
SYYATSYLEYEIENFGAQHSTTLFKINNNTFVLLDRPHTPQFLFQLNDTIHLHQQLSNTTGKLIW
TLDANINADIGEWAFWENKKNLSEQLRGEELSFETLSL
>4AE2A
ETGHHHHHHSADEPMDFKINTDEIMTSLKSVNGQIESLISPDGSRKNPARNCRDLKFCHPELKSG
EYWVDPNQGCKLDAIKVFCNMETGETCISANPLNVPRKHWWTDSSAEKKHVWFGESMDGGFQFSY
GNPELPEDVLDVQLAFLRLLSSRASQQITYHCKNSIAYMDQASGNVKKALKLMGSNEGEFKAEGN
SKFTYTVLEDGCTKHTGEWSKTVFEYRTRKAVRLPIVDIAPYDIGGPDQEFGVDVGPVCFL
>3VDIA
MALFGTKDTTTAHSDYEIILEGGSSSWGQIKGRAKVNVPAALPLLPADCNIKIEAKPLDAQKGVV
RFTSQIESIVDSTKNKLVVEVDIANETKDRRIAVGEGEVSVGDFSHKFSFEGSVVNMYYYRSDAV
RRNVPNPVYMQGRQFHDIMMKVPLDNKDLIETWEGFQQSISGGGVNFGDWIREFWFIGPAYTAIN
EGGQRISPIQVNNFGVESGEKGPVGVSRWKFSHAGSGIVDSISRWAELFPVEQLNKPASIEGGFR
SDSQGIEVKVDGNLPGVSRDAGGGLRRILNHPLIPLVHHGMVGKFNDFTVDTQLKVVLPKGYKIR
YAAPQFRSQNLEEYRWSGGAYARWVEHVCKGGTGQFEVLYAQ
>3VDJA
YVEFEPSDKHIKEYLNKIQNSLSTEWSPCSVTCGNGIQVRIKPGSANKPKDELDYANDIEKKICK
MEKCPHHHHHHA
>4ADZA
GSHMTTTEAGASAPSPAVDGAVNQTARQAEADGTDIVTDHDRGVHGYHKQKAEHLKRLRRIEGQI
RGLQRMVDEDVYCIDILTQVSASTKALQSFALQLLEEHLRHCVADAALKGGTEIDAKVEEATKAI
GRLLRT
>3VBAA
MRSIIKGRVWKFGNNVDTDAILPARYLVYTKPEELAQFVMTGADPDFPKKVKPGDIIVGGKNFGC
GSSREHAPLGLKGAGISCVIAESFARIFYRNAINVGLPLIECKGISEKVNEGDELEVNLETGEIK
NLTTGEVLKGQKLPEFMMEILEAGGLMPYLKKKMAESQLEHHHHHH
>3V9OA
GPGSMTGTTMFAALLHPRLADCRRLYLRNHEVYMNIGAFEHEKRGEQRVVINVDLFVPLALTTPV
EDKLREVVDYDLMKQSVAQCVARGHIHLQETLCDAIAASLLAHDAVRAVRVSTEKPDAYPDCDAV
GVEVFRIKDEERA
>3V9BA
IPRFGVKTEQEDVLAKELEDVNKWGLHVFRIAELSGNRPLTVIMHTIFQERDLLKTFKIPVDTLI

TYLMTLEDHYHADVAYHNNIHAADVVQSTHVLLSTPALEAVFTDLEILAAIFASAIHDVDHPGVS
NQFLINTNSELALMYNDSSVLENHHLAVGFKLLQEENCDIFQNLTKKQRQSLRKMVIDIVLATDM
SKHMNLLADLKTMVETKKVTSSGVLLLDNYSDRIQVLQNMVHCADLSNPTKPLQLYRQWTDRIME
EFFRQGDRERERGMEISPMCDKHNASVEKSQVGFIDYIVHPLWETWADLVHPDAQDILDTLEDNR
EWYQSTIPQSPSPAPDDPEEGRQGQTEKFQFELTL
>2LNAA
MGHHHHHHSHMKRSGREITWKDFVNNYLSKGVVDRLEVVNKRFVRVTFTPGKTPVDGQYVWFNIG
SVDTFERNLETLQQELGIEGENRVPVVYIAESDG
>3V65B
TGEENCNVNNGGCAQKCQMIRGAVQCTCHTGYRLTEDGRTCQDVNECAEEGYCSQGCTNSEGAFQ
CWCEAGYELRPDRRSCKALGPEPVLLFANRIDIRQVLPHRSEYTLLLNNLENAIALDFHHRRELV
FWSDVTLDRILRANLNGSNVEEVVSTGLESPGGLAVDWVHDKLYWTDSGTSRIEVANLDGAHRKV
LLWQSLEKPRAIALHPMEGTIYWTDWGNTPRIEASSMDGSGRRIIADTHLFWPNGLTIDYAGRRM
YWVDAKHHVIERANLDGSHRKAVISQGLPHPFAITVFEDSLYWTDWHTKSINSANKFTGKNQEII
RNKLHFPMDIHTLHPQRQPAGKNRCGDNNGGCTHLCLPSGQNYTCACPTGFRKINSHACAQ
>3V53A
MGHHHHHHMKRKHIKSLIEKIPTAKPELFAYPLDWSIVDSILMERRIRPWINKKIIEYIGEEEAT
LVDFVCSKVMAHSSPQSILDDVAMVLDEEAEVFIVKMWRLLIYETEAKKIGLVK
>3V57B
MLDAFSRVVVNSDAKAAYVGGSDLQALKSFIADGNKRLDAVNSIVSNASCMVSDAVSGMICENPG
LISPGGNCYTNRRMAACLRDGEIILRYVSYALLAGDASVLEDRCLNGLKETYIALGVPTNSSIRA
VSIMKAQAVAFITNTATERKMSFAAGDCTSLASEVASYFDRVGAAIS
>3V43A
HMEPIPICSFCLGTKEQNREKKPEELISCADCGNSGHPSCLKFSPELTVRVKALRWQCIECKTCS
SCRDQGKNADNMLFCDSCDRGFHMECCDPPLTRMPKGMWICQICRPR
>4AC5C
CFEPPPATTTQTGFRGLSMGEVLHPATVKAKKERDAQYPPALAAVKAEGPPVSQVYKNVKVLGNL
TEAEFLRTMTAITEWVSPQEGCTYCHDENNLASEAKYPYVVARRMLEMTRAINTNWTQHVAQTGV
TCYTCHRGTPLPPYVRYLEPTLPLNNRETPTHVERVETRSGYVVRLAKYTAYSALNYDPFTMFLA
NDKRQVRVVPQTALPLVGVSRGKERRPLSDAYATFALMMSISDSLGTNCTFCHNAQTFESWGKKS
TPQRAIAWWGIRMVRDLNMNYLAPLNASLPASRLGRQGEAPQADCRTCHQGVTKPLFGASRLKDY
PELGPIKAAAK
>3V48A
GHMKLSLSPPPYADAPVVVLISGLGGSGSYWLPQLAVLEQEYQVVCYDQRGTGNNPDTLAEDYSI
AQMAAELHQALVAAGIEHYAVVGHALGALVGMQLALDYPASVTVLISVNGWLRINAHTRRCFQVR
ERLLYSGGAQAWVEAQPLFLYPADWMAARAPRLEAEDALALAHFQGKNNLLRRLNALKRADFSHH
ADRIRCPVQIICASDDLLVPTACSSELHAALPDSQKMVMPYGGHACNVTDPETFNALLLNGLASL
LHHREAAL
>3V2AR
MQSKVLLAVALWLCVETRAASVGLPSVSLDLPRLSIQKDILTIKANTTLQITCRGQRDLDWLWPN
NQSGSEQRVEVTECSDGLFCKTLTIPKVIGNDTGAYKCFYRETDLASVIYVYVQDYRSPFIASVS
DQHGVVYITENKNKTVVIPCLGSISNLNVSLCARYPEKRFVPDGNRISWDSKKGFTIPSYMISYA
GMVFCEAKINDESYQSIMYIVVVVGYRIYDVVLSPSHGIELSVGEKLVLNCTARTELNVGIDFNW
EYPSSKHQHKKLVNRDLKTQSGSEMKKFLSTLTIDGVTRSDQGLYTCAASSGLMTKKNSTFVRVH

EKPFVAFGSGMESLVEATVGERVRIPAKYLGYPPPEIKWYKNGIPLESNHTIKAGHVLTIMEVSE
RDTGNYTVILTNPISKEKQSHVVSLVVYVPPQIGEKSLISPVDSYQYGTTQTLTCTVYAIPPPHH
IHWYWQLEEECANEPSQAVSVTNPYPCEEWRSVEDFQGGNKIEVNKNQFALIEGKNKTVSTLVIQ
AANVSALYKCEAVNKVGRGERVISFHVTRGPEITLQPDMQPTEQESVSLWCTADRSTFENLTWYK
LGPQPLPIHVGELPTPVCKNLDTLWKLNATMFSNSTNDILIMELKNASLQDQGDYVCLAQDRKTK
KRHCVVRQLTVLERVAPTITGNLENQTTSIGESIEVSCTASGNPPPQIMWFKDNETLVEDSGIVL
KDGNRNLTIRRVRKEDEGLYTCQACSVLGCAKVEAFFIIEGAQEKTNLERTHHHHHH

>3J16C

MKLNISYPVNGSQKTFEIDDEHRIRVFFDKRIGQEVDGEAVGDEFKGYVFKISGGNDKQGFPMKQ
GVLLPTRIKLLLTKNVSCYRPRRDGERKRKSVRGAIVGPDLAVLALVIVKKGEQELEGLTDTTVP
KRLGPKRANNIRKFFGLSKEDDVRDFVIRREVTKGEKTYTKAPKIQRLVTPQRLQRKRHQRALKV
RNAQAQREAAAEYAQLLAKRLSERKAEKAEIRKRRASSLKA

>3VMGA

MANVDEAILKRVKGWAPYVDAKLGFRNHWYPVMFSKEINEGEPKTLKLLGENLLVNRIDGKLYCL
KDRCLHRGVQLSVKVECKTKSTITCWYHAWTYRWEDGVLCDILTNPTSAQIGRQKLKTYPVQEAK
GCVFIYLGDGDPPPLARDTPPNFLDDDMEILGKNQIIKSNWRLAVENGFDPSHIYIHKDSILVKD
NDLALPLGFAPGGDRKQQTRVVDDDVVGRKGVYDLIGEHGVPVFEGTIGGEVVREGAYGEKIVAN
DISIWLPGVLKVNPFPNPDMMQFEWYVPIDENTHYYFQTLGKPCANDEERKKYEQEFESKWKPMA
LEGFNNDDIWAREAMVDFYADDKGWVNEILFESDEAIVAWRKLASEHNQGIQTQAHVSGLEHHHH
HH

>3V33A

GGGTPKAPNLEPPLPEEEKEGSDLRPVVIDGSNVAMSHGNKEVFSCRGILLAVNWFLERGHTDIT
VFVPSWRKEQPRPDVPITDQHILRELEKKKILVFTPSRRVGGKRVVCYDDRFIVKLAYESDGIVV
SNDTYRDLQGERQEWKRFIEERLLMYSFVNDKFMPPDDPLGRHGPSLDNFLRKKPLTLEHRKQPC
PYGRKCTYGIKCRFFHPERPSCPQRSVA

>3V22V

MKRQKRDRLERAHQRGYQAGIAGRSKEMCPYQTLNQRSQWLGGWREAMADRVVMAHHHHHH

>4ABRL

MVALPTINQLVRKGREKVRKKSKVPALKGAPFRRGVCTVVRTVTPKKPNSALRKVAKVRLTSGYE
VTAYIPGEGHNLQEHSVVLIRGGRVKDLPGVRYHIVRGVYDAAGVKDRKKSRSKYGTKKPKEAAK
TAAKK

>3VM6A

MNHKVHHHHHHIEGRHMAVVKEVLEIAEKIKNMEIRGAGKIARSAAYALQLQAEKSKATNVDEFW
KEMKQAAKILFETRPTAVSLPNALRYVMHRGKIAYSSGADLEQLRFVIINAAKEFIHNSEKALER
IGEFGAKRIEDGDVIMTHSHSKAAISVMKTAWEQGKDIKVIVTETRPKWQGKITAKELASYGIPV
IYVVDSAARHYMKMTDKVVMGADSITVNGAVINKIGTALIALTAKEHRVWTMIAAETYKFHPETM
LGQLVEIEMRDPTEVIPEDELKTWPKNIEVWNPAFDVTPPEYVDVIITERGIIPPYAAIDILREE
FGWALKYTEPWED

>3J0TL

MKTFTAKPETVKRDWYVVDATGKTLGRLATELARRLRGKHKAEYTPHVDTGDYIIVLNADKVAVT
GNKRTDKVYYHHTGHIGGIKQATFEEMIARRPERVIEIAVKGMLPKGPLGRAMFRKLKVYAGNEH
NHAAQQPQVLDI

>3UXQD

MAVKKFKPYTPSRRFMTVADFSEITKTEPEKSLVKPLKKTGGRNNQGRITVRFRGGGHKRLYRII

DFKRWDKVGIPAKVAAIEYDPNRSARIALLHYVDGEKRYIIAPDGLQVGQQVVAGPDAPIQVGNA
LPLRFIPVGTVVHAVELEPKKGAKLARAAGTSAQIQGREGDYVILRLPSGELRKVHGECYATVGA
VGNADHKNIVLGKAGRSRWLGRRPHVRGAAMNPVDHPHGGGEGRAPRGRPPASPWGWQTKGLKTR
KRRKPSSRFIIARRKK
>3UX2A
SNARIMEEKALEVYDLIRTIRDPEKPNTLEELEVVSESCVEVQEINEEEYLVIIRFTPTVPHCSL
ATLIGLCLRVKLQRCLPFKHKLEIYISEGTHSTEEDINKQINDKERVAAAMENPNLREIVEQCVL
>3UW8A
MAETPNSDMSGATGGRSKRPKSNQDWWPSKLNLEILDQNARDVGPVEDDFDYAEEFQKLDLEAVK
SDLEELMTSSQDWWPADYGHYGPLFIRMAWHSAGTYRTADGRGGAAGGRQRFAPINSWPDNANLD
KARRLLLPIKQKYGQKISWADLMILAGNVAIESMGFKTFGYAGGREDAFEEDKAVNWGPEDEFET
QERFDEPGEIQEGLGASVMGLIYVNPEGPDGNPDPEASAKNIRQTFDRMAMNDKETAALIAGGHT
FGKVHGADDPEENLGPEPEAAPIEQQGLGWQNKNGNSKGGEMITTGIEGPWTQSPTEWDMGYINN
LLDYEWEPEKGPGGAWQWAPKSEELKNSVPDAHDPDEKQTPMMLTTDIALKRDPDYREVMETFQE
NPMEFGMNFAKAWYKLTHRDMGPPERFLGPEVPDEEMIWQDPLPDADYDLIGDEEIAELKEEILD
SDLSVSQLVKTAWASASTYRDSDKRGGANGARLRLEPQKNWEVNEPEQLETVLGTLENIQTEFND
SRSDGTQVSLADLIVLGGNAAVEQAAANAGYDVEIPFEPGRVDAGPEHTDAPSFDALKPKVDGVR
NYIQDDITRPAEEVLVDNADLLNLTASELTALIGGMRSIGANYQDTDLGVFTDEPETLTNDFFVN
LLDMGTEWEPAADSEHRYKGLDRDTGEVKWEATRIDLIFGSNDRLRAISEVYGSADAEKKLVHDF
VDTWSKVMKLDRFDLEHHHHHH
>3UW2A
MAHHHHHHMGTLEAQTQGPGSMISQSIFKAYDIRGVIGKTLDADVARSIGRAFGSEVRAQGGDAV
VVARDGRLSGPELVGALADGLRAAGVDVVDVGMVPTPVGYFAASVPLALSGGERRVDSCIVVTGS
HNPPDYNGFKMVLRGAAIYGDQIQGLYKRIVDARFETGSGSYEQYDVADQYVERIVGDIKLTRPL
KLVVDAGNGVAGPLATRLFKALGCELVELFTDIDGNFPNHHPDPAHPENLQDVIAKLKATDAEIG
FAFDGDGDRLGVVTKDGQIIYPDRQLMLFAEEVLSRNPGAQIIYDVKCTRNLARWVREKGGEPLM
WKTGHSLVKAKLRETGAPLAGEMSGHVFFKDRWYGFDDGLYTGARLLEILARVADPSALLNGLPN
AVSTPELQLKLEEGENVKLIDKLRADAKFDGADEVVTIDGLRVEYPDGFGLARSSNTTPVVVLRF
EATSDAALARIQDDFRRALKAAKPGANLPF
>3UV1A
DPIHYDKITEEINKAIDDAIAAIEQSETIDPMKVPDHADKFERHVGILDFKGELAMRNIEARGLK
QMKRQGDANVKGEEGIVKAHLLIGVHDDIVSMEYDLAYKLGDLHPTTHVISDIQDFVVALSLEIP
DEGNITMTSFEVRQFANVVNHIGGLSILDPIFGVLSDVLTAIFQDTVRKEMTKVLAPAFKRELEK
N
>4A9WA
MDSVDVVVIGGGQSGLSAGYFLRRSGLSYVILDAEASPGGAWQHAWHSLHLFSPAGWSSIPGWPM
PASQGPYPARAEVLAYLAQYEQKYALPVLRPIRVQRVSHFGERLRVVARDGRQWLARAVISATGT
WGEAYTPEYQGLESFAGIQLHSAHYSTPAPFAGMRVAIIGGGNSGAQILAEVSTVAETTWITQHE
PAFLADDVDGRVLFERATERWKAQQEGREPDLPPGGFGDIVMVPPVLDARARGVLAAVPPPARFS
PTGMQWADGTERAFDAVIWCTGFRPALSHLKGLDLVTPQGQVEVDGSGLRALAVPSVWLLGYGDW
NGMASATLIGVTRYAREAVRQVTAYCADHQDR
>3UV2A
SMQCQSTEDAMTVLTPLTEKDYEGLKRVLRSLQAHKMAWPFLEPVDPNDAPDYYGVIKEPMDLAT
MEERVQRRYYEKLTEFVADMTKIFDNCRYYNPSDSPFYQCAEVLESFFVQKLKGFKASRSH

>3UUMA
DMDLDSYQIALEEVLTWLLSAEDTFQEQDDISDDVEDVKEQFATHETFMMELSAHQSSVGSVLQA
GNQLMTQGTLSDEEEFEIQEQMTLLNARWEALRVESMERQSRLHDALMELQKKQLQQL
>3UTNX
MGSSHHHHHHSSGLVPRGSHMASMPLFDLISPKAFVKLVASEKVHRIVPVDATWYLPSWKLDNKV
DFLTKPRIPNSIFFDIDAISDKKSPYPHMFPTKKVFDDAMSNLGVQKDDILVVYDRVGNFSSPRC
AWTLGVMGHPKVYLLNNFNQYREFKYPLDSSKVAAFSPYPKSHYESSESFQDKEIVDYEEMFQLV
KSGELAKKFNAFDARSLGRFEGTEPEPRSDIPSGHIPGTQPLPYGSLLDPETKTYPEAGEAIHAT
LEKALKDFHCTLDPSKPTICSCGTGVSGVIIKTALELAGVPNVRLYDGSWTEWVLKSGPEWIAEN
RD
>4A9AA
MHHHHHHMSTTVEKIKAIEDEMARTQKNKATSFHLGQLKAKLAKLRRELLTSASSGSGGGAGIGF
DVARTGVASVGFVGFPSVGKSTLLSKLTGTESEAAEYEFTTLVTVPGVIRYKGAKIQMLDLPGII
DGAKDGRGRGKQVIAVARTCNLLFIILDVNKPLHHKQIIEKELEGVGIRLNKTPPDILIKKKEKG
GISITNTVPLTHLGNDEIRAVMSEYRINSAEIAFRCDATVDDLIDVLEASSRRYMPAIYVLNKID
SLSIEELELLYRIPNAVPISSGQDWNLDELLQVMWDRLNLVRIYTKPKGQIPDFTDPVVLRSDRC
SVKDFCNQIHKSLVDDFRNALVYGSSVKHQPQYVGLSHILEDEDVVTILKK
>4A91A
MTDTQYIGRFAPSPSGELHFGSLIAALGSYLQARARQGRWLVRIEDIDPPREVPGAAETILRQLE
HYGLHWDGDVLWQSQRHDAYREALAWLHEQGLSYYCTCTRARIQSIGGIYDGHCRVLHHGPDNAA
VRIRQQHPVTQFTDQLRGIIHADEKLAREDFIIHRRDGLFAYNLAVVVDDHFQGVTEIVRGADLI
EPTVRQISLYQLFGWKVPDYIHLPLALNPQGAKLSKQNHAPALPKGDPRPVLIAALQFLGQQAEA
HWQDFSVEQILQSAVKNWRLTAVPESAIVNSTFSNASC
>3UR1C
SQIGETLENIRSIEKLIQNIMRIARETNILALNATIEAARAGEAGKGFMIVANEVQNLSNETNEV
TKQIVEKAREILESSQRSLE
>4A8JC
MHHHHHHMGSVQRQDLVLFSDQSVLPAHFFQDSNSHNLFFITHQSCTQPLWMINALVETHVLGSP
SSLNESSSSMLPSSTRSHAVLASFIHEQNYFTNSLNKLKIPSNNYNVLDFLSDFIVNNIHNKPRD
KILSDVLAKFSAAIQNNPTDTIVIIEQPELLLSLVSGLTCSELNNKFITPLLRQCKVLIIVSNSD
IFNIDEYDASVHSSNLQNFYKSSFIKSMINLNLNPLKTGFAKDVTGSLHVCRGGAPIATSNTSLH
VVENEYLYLNEKESTKLFYR
>3UR1B
KEFEVLSFEIDEQALAFDVDNIEMVIEKSDITPVPKSRHFVEGVINLRGRIIPVVNLAKILGISF
DEQKMKSIIVARTKDVEVGFLVDRVLGVLRITENQLDLTNVSDKFGKKSKGLVKTDGRLIIYLDI
DKIIEEITV
>4A8JB
MASSSHNPVILLKRILSLTESSPFILCLDSIAQTSYKLIQEFVHQSKSKGNEYPIVYISFETVNK
PSYCTQFIDATQMDFVHLVKQIISYLPAATATQAKKHMVIIDSLNYISTEYITRFLSEIASPHCT
MVATYHKDIKDENRTVIPDWNNNYPDKLTLLQFMATTIVDIDVVLTGTLDTEEVSELLNEFRIPR
GLNNDIFQLRLVNKRKSGRSLEYDFIVNSNTHEYELLSTTKQEEESSSNGLETPEMLQGLTTFNL
GTSNKQKLAK
>3UPIA
SMSYTWTGALITPCAAEESKLPINPLSNSLLRHHNMVYATTSRSASLRQKKVTFDRLQVLDDHYR

DVLKEMKAKASTVKAKLLSIEEACKLTPPHSAKSKFGYGAKDVRNLSSRAVNHIRSVWEDLLEDT
ETPIDTTIMAKSEVFCVQPEKGGRKPARLIVFPDLGVRVCEKMALYDVVSTLPQAVMGSSYGFQY
SPKQRVEFLVNTWKSKKCPMGFSYDTRCFDSTVTESDIRVEESIYQCCDLAPEARQAIRSLTERL
YIGGPLTNSKGQNCGYRRCRASGVLTTSCGNTLTCYLKATAACRAAKLQDCTMLVNGDDLVVICE
SAGTQEDAAALRAFTEAMTRYSAPPGDPPQPEYDLELITSCSSNVSVAHDASGKRVYYLTRDPTT
PLARAAWETARHTPINSWLGNIIMYAPTLWARMILMTHFFSILLAQEQLGKALDCQIYGACYSIE
PLDLPQIIERLHGLSAFTLHSYSPGEINRVASCLRKLGVPPLRTWRHRARSVRAKLLSQGGRAAI
CGRYLFNWAVRTKLKLTPIPAASQLDLSGWFVAGYSGGDIYHSLSRARPRENLYFQGLEHHHHHH
>3UOQG
MPRRRVIGQRKILPDPKFGSELLAKFVNILMVDGKKSTAESIVYSALETLAQRSGKSELEAFEVA
LENVRPTVEVKSRRVGGSTYQVPVEVRPVRRNALAMRWIVEAARKRGDKSMALRLANELSDAAEN
KGTAVKKREDVHRMAEANKAFAHYRWLSLRSFSHQAGASSKQPALGYLN
>3VKFA
SQKLDDVDPLVTTNFGKIRGIKKELNNEILGPVIQFLGVPYAAPPTGEHRFQPPEPPSPWSDIRN
ATQFAPVCPQNIIDGRLPEVMLPVWFTNNLDVVSSYVQDQSEDCLYLNIYVPTEDVKRISKECAR
KPGKKICRKGDIRDSGGPKPVMVYIHGGSYMEGTGNLYDGSVLASYGNVIVITVNYRLGVLGFLS
TGDQAAKGNYGLLDLIQALRWTSENIGFFGGDPLRITVFGSGAGGSCVNLLTLSHYSEGLFQRAI
AQSGTALSSWAVSFQPAKYARILATKVGCNVSDTVELVECLQKKPYKELVDQDVQPARYHIAFGP
VIDGDVIPDDPQILMEQGEFLNYDIMLGVNQGEGLKFVENIVDSDDGVSASDFDFAVSNFVDNLY
GYPEGKDVLRETIKFMYTDWADRHNPETRRKTLLALFTDHQWVAPAVATADLHSNFGSPTYFYAF
YHHCQTDQVPAWADAAHGDEVPYVLGIPMIGPTELFPCNFSKNDVMLSAVVMTYWTNFAKTGDPN
QPVPQDTKFIHTKPNRFEEVAWTRYSQKDQLYLHIGLKPRVKEHYRANKVNLWLELVPHLHNLND
>4A7FB
ELDRAQERLATALQKLEEAEKAADESERGMKVIESRAQKDEEKMEIQEIQLKEAKHIAEDADRKY
EEVARKLVIIESDLERAEERAELSEGKCAELEEELKTVTNNLKSLEAQAEKYSQKEDKYEEEIKV
LSDKLK
>4A7KA
MYSKVFLKPHCEPEQPAALPLFQPQLVQGGRPDGYWVEAFPFRSDSSKCPNIIGYGLGTYDMKSD
IQMLVNPYATTNNQSSSWTPVPLAKLDFPVAMHYADITKNGFNDVIITDQYGSSMDDIWAYGGRV
SWLENPGELRDNWTMRTIGHSPGMHRLKAGHFTRTDRVQVVAVPIVVASSDLTTPADVIIFTAPD
DPRSEQLWQRDVVGTRHLVHEVAIVPAAETDGEMRFDQIILAGRDGVDCLWYDGARWQRHLVGTG
LPEERGDPYWGAGSAAVGRVGDDYAGYICSAEAFHGNTVSVYTKPAGSPTGIVRAEWTRHVLDVF
GPLNGKHTGSIHQVVCADIDGDGEDEFLVAMMGADPPDFQRTGVWCYKLVDRTNMKFSKTKVSSV
SAGRIATANFHSQGSEVDIATISYSVPGYFESPNPSINVFLSTGILAERLDEEVMLRVVRAGSTR
FKTEMEFLDVAGKKLTLVVLPPFARLDVERNVSGVKVMAGTVCWADENGKHERVPATRPFGCESM
IVSADYLESGEEGAILVLYKPSSTSGRPPFRSMDELVAHNLFPAYVPDSVRAMKFPWVRCADRPW
AHGRFKDLDFFNLIGFHVNFADDSAAVLAHVQLWTAGIGVSAGFHNHVEASFCEIHACIANGTGR
GGMRWATVPDANFNPDSPNLEDTELIVVPDMHEHGPLWRTRPDGHPLLRMNDTIDYPWHAWLAGA
GNPSPQAFDVWVAFEFPGFETFSTPPPPRVLEPGRYAIRFGDPHQTASLALQKNDATDGTPVLAL
LDLDGGPSPQAWNISHVPGTDMYEIAHAKTGSLVCARWPPVKNQRVAGTHSPAAMGLTSRWAVTK
NTKGQITFRLPEAPDHGPLFLSVSAIRHQQEADAIPVIVQGDSIELSAWSLVPAN
>3UMFA
MGSSHHHHHHSSGLVPRGSHMTDQKLAKAKVIFVLGGPGSGKGTQCEKLVQKFHFNHLSSGDLLR
AEVQSGSPKGKELKAMMERGELVPLEVVLALLKEAMIKLVDKNCHFLIDGYPRELDQGIKFEKEV

CPCLCVINFDVSEEVMRKRLLKRAETSNRVDDNEETIVKRFRTFNELTKPVIEHYKQQNKVITID
ASGTVDAIFDKVNHELQKFGVK
>3ULRB
GPLGSSDLGITAIALYDYQAAGDDEISFDPDDIITNIEMIDDGWWRGVCKGRYGLFPANYVELRQ
>2LLIA
KEAAPKCNNCSQRGHLKKDCPHIICSYCGATDDHYSRHCPKAIQCSKCDEVGHYRSQCPHKWKKV
QCTLCKSKKHSKERCPSIWRAYILVDDNEKAKPKVLPFHTIYCYNCGGKGHFGDDCKEK
>4A6SA
AWKGEVLANNEAGQVTSIIYNPGDVITIVAAGWASYGPTQKWGPQGDREHPDQGLICHDAFCGAL
VMKIGNSGTIPVNTGLFRWVAPNNVQGAITLIYNDVPGTYGNNSGSFSVNIGKDQS
>2LLFA
PRLFECSNKTGRFLATEIVDFTQDDLDENDVYLLDTWDQIFFWIGKGANESEKEAAAETAQEYLR
SHPGSRDLDTPIIVVKQGFEPPTFTGWFMAWDPLCWSDRKSY
>3UILA
EDPPACGSIVPRREWRALASECRERLTRPVRYVVVSHTAGSHCDTPASCAQQAQNVQSYHVRNLG
WCDVGYNFLIGEDGLVYEGRGWNIKGAHAGPTWNPISIGISFMGNYMNRVPPPRALRAAQNLLAC
GVALGALRSNYEVKGHRDVQPTLSPGDRLYEIIQTWSHYRA
>3UGJA
GLVPRGSHMMEILRGSPALSAFRINKLLARFQAANLQVHNIYAEYVHFADLNAPLNDSEQAQLTR
LLQYGPALSSHTPAGKLLLVTPRPGTISPWSSKATDIAHNCGLQQVDRLERGVAYYIEASTLTAE
QWRQVAAELHDRMMETVFSSLTDAEKLFIHHQPAPVSSVDLLGEGRQALIDANLRLGLALAEDEI
DYLQEAFTKLGRNPNDIELYMFAQANSEHCRHKIFNADWIIDGKPQPKSLFKMIKNTFETTPDYV
LSAYKDNAAVMEGSAVGRYFADHNTGRYDFHQEPAHILMKVETHNHPTAISPWPGAATGSGGEIR
DEGATGRGAKPKAGLVGFSVSNLRIPGFEQPWEEDFGKPERIVTALDIMTEGPLGGAAFNNEFGR
PALTGYFRTYEEKVNSHNGEELRGYHKPIMLAGGIGNIRADHVQKGEIVVGAKLIVLGGPAMNIG
LGGGAASSMASGQSDADLDFASVQRDNPEMERRCQEVIDRCWQLGDANPILFIHDVGAGGLSNAM
PELVSDGGRGGKFELRDILSDEPGMSPLEIWCNESQERYVLAVAADQLPLFDELCKRERAPYAVI
GDATEEQHLSLHDNHFDNQPIDLPLDVLLGKTPKMTRDVQTLKAKGDALNRADITIADAVKRVLH
LPTVAEKTFLVTIGDRTVTGMVARDQMVGPWQVPVADCAVTTASLDSYYGEAMSIGERAPVALLD
FAASARLAVGEALTNIAATQIGDIKRIKLSANWMAAAGHPGEDAGLYDAVKAVGEELCPQLGLTI
PVGKDSMSMKTRWQEGNEQREMTSPLSLVISAFARVEDVRHTLTPQLSTEDNALLLIDLGKGHNA
LGATALAQVYRQLGDKPADVRDVAQLKGFYDAMQALVAARKLLAWHDRSDGGLLVTLAEMAFAGH
CGVQVDIAALGDDHLAALFNEELGGVIQVRAEDRDAVEALLAQYGLADCVHYLGQALAGDRFVIT
ANDQTVFSESRTTLRVWWAETTWQMQRLRDNPQCADQEHEAKANDTDPGLNVKLSFDINEDIAAP
YIATGARPKVAVLREQGVNSHVEMAAAFHRAGFDAIDVHMSDLLGGRIGLGNFHALVACGGFSYG
DVLGAGEGWAKSILFNHRVRDEFETFFHRPQTLALGVCNGCQMMSNLRELIPGSELWPRFVRNHS
DRFEARFSLVEVTQSPSLLLQGMVGSQMPIAVSHGEGRVEVRDDAHLAALESKGLVALRYVDNFG
KVTETYPANPNGSPNGITAVTTENGRVTIMMPHPERVFRTVANSWHPENWGEDSPWMRIFRNARK
QLG
>3UGQA
MGSSHHHHHHSSGLVPRGSHMASATKNASSATPATMTSMVSQRQDLFMTDPLSPGSMFFLPNGAK
IFNKLIEFMKLQQKFKFGFNEVVTPLIYKKTLWEKSGHWENYADDMFKVETTDEEKEEYGLKPMN
CPGHCLIFGKKDRSYNELPLRFSDFSPLHRNEASGALSGLTRLRKFHQDDGHIFCTPSQVKSEIF
NSLKLIDIVYNKIFPFVKGGSGAESNYFINFSTRPDHFIGDLKVWNHAEQVLKEILEESGKPWKL

NPGDGAFYGPKLDIMVTDHLRKTHQVATIQLDFQLPERFDLKFKDQDNSYKRPIMIHRATFGSIE
RFMALLIDSNEGRWPFWLNPYQAVIIPVNTKNVQQLDMCTALQKKLRNELEADDMEPVPLNDWHF
NVDLDIRNEPVGYRIKSAILKNYSYLIIVGDEEVQLQKYNIRERDNRKSFEKLTMSQIWEKFIEL
EKNYK
>4A69C
GAMRQLAVIPPMLYDADQQRIKFINMNGLMADPMKVYKDRQVMNMWSEQEKETFREKFMQHPKNF
GLIASFLERKTVAECVLYYYLTKKNENYK
>4A6DA
MGSSEDQAYRLLNDYANGFMVSQVLFAACELGVFDLLAEAPGPLDVAAVAAGVRASAHGTELLLD
ICVSLKLLKVETRGGKAFYRNTELSSDYLTTVSPTSQCSMLKYMGRTSYRCWGHLADAVREGRNQ
YLETFGVPAEELFTAIYRSEGERLQFMQALQEVWSVNGRSVLTAFDLSVFPLMCDLGGGAGALAK
ECMSLYPGCKITVFDIPEVVWTAKQHFSFQEEEQIDFQEGDFFKDPLPEADLYILARVLHDWADG
KCSHLLERIYHTCKPGGGILVIESLLDEDRRGPLLTQLYSLNMLVQTEGQERTPTHYHMLLSSAG
FRDFQFKKTGAIYDAILARKGTHHHHHH
>3UEZE
GSHMEEVSEYCSHMIGSGHLQSLQRLIDSQMETSCQITFEFVDQEQLKDPVCYLKKAFLLVQDIM
EDTMRFRDNTPNAIAIVQLQELSLRLKSCFTKDYEEHDKACVRTFYETPLQLLEKVKNVFNETKN
LLDKDWNIFSKNCNNSFAECSSQ
>4A5VA
SSEPAKLDLSCVHSDNKGSRAPTIGEPVPDVSLEQCAAQCKAVDGCTHFTYNDDSKMCHVKEGKP
DLYDLTGGKTASRSCDRSCFEQHVSYEGAPDVMTAMVTSQSADCQAACAADPSCEIFTYNEHDQK
CTFKGRGFSAFKERGVLGVTSGPKQFCDEGG
>3UBBA
ERAGPVTWVMMIACVVVFIAMQILGDQEVMLWLAWPFDPTLKFEFWRYFTHALMHFSLMHILFNL
LWWWYLGGAVEKRLGSGKLIVITLISALLSGYVQQKFSGPWFGGLSGVVYALMGYVWLRGERDPQ
SGIYLQRGLIIFALIWIVAGWFDLFGMSMANGAHIAGLAVGLAMAFVDSLNA
>3UBRA
SDKTEPRNEVYKDKFKNQYNSWHDTAKSEELVDALEQDPNMVILWAGYAFAKDYKAPRGHMYAVT
DVRNTLRTGAPKNAEDGPLPMACWSCKSPDVPRLIEEQGEDGYFKGKWAKGGPEVTNTIGCSDCH
EKGSPKLRISRPYVDRALDAIGTPFSKASKQDKESMVCAQCHVEYYFEKKEDKKGFVKFPWDMGV
TVDQMEVYYDGIEFSDWTHALSKTPMLKAQHPEYETWKMGIHGKNNVSCVDCHMPKVTSPEGKKF
TDHKVGNPFDRFEETCATCHSQTKEFLVGVTNERKAKVKEMKLKAEEQLVKAHFEAAKAWELGAT
EAEMKPILTDIRHAQWRWDLAIASHGVAAHAPEEALRVLGTSVNKAADARVKLAQLLAKKGLTDP
VAIPDISTKAKAQAVLGMDMEKMNAEKEAFKKDMLPKWDAEAKKREATY
>4A53A
GAMGMSVADFYGSNVEVLLNNDSKARGVITNFDSSNSILQLRLANDSTKSIVTKDIKDLRILPKN
EIMPKNGTKSPSTNSTKLKSAETYSSKNKWSMDCDEEFDFAANLEKFDKKQVFAEFREKD
>3UA0A
MGHHHHHHMRVKTFVILCCALQYVAYTNANINDFDEDYFGSDVTVQSSNTTDEIIRDASGAVIEE
QITTKKMQRKNKNHGILGKNEKMIKTFVITTDSDGNESIVEEDVLMKTLSDGTVAQSYVAADAGA
YSQS
>3U9GA
GPLGMADPGVCCFITKILCAHGGRMTLEELLGEIRLPEAQLYELLETAGPDRFVLLETGGQAGIT
RSVVATTRARVCRRKYCQRPCDSLHLCKLNLLGRCHYAQSQRNLCKYSHDVLSEQNFQILKNHEL

SGLNQEELACLLVQSDPFFLPEICKSYKGEGRKQTCGQPQPCERLHICEHFTRGNCSYLNCLRSH
NLMDRKVLTIMREHGLSPDVVQNIQDICNNKHAR
>3U88C
SMDSRLQRIHAEIKNSLKIDNLDVNRCIEALDELASLQVTMQQAQKHTEMITTLKKIRRFKVSQV
IMEKSTMLYNKFKNMFLVGEGDSV
>3U6WA
GAMTTSESPDAYTESFGAHTIVKPAGPPRVGQPSWNPQRASSMPVNRYRPFAEEVEPIRLRNRTW
PDRVIDRAPLWCAVDLRDGNQALIDPMSPARKRRMFDLLVRMGYKEIEVGFPSASQTDFDFVREI
IEQGAIPDDVTIQVLTQCRPELIERTFQACSGAPRAIVHFYNSTSILQRRVVFRANRAEVQAIAT
DGARKCVEQAAKYPGTQWRFEYSPESYTGTELEYAKQVCDAVGEVIAPTPERPIIFNLPATVEMT
TPNVYADSIEWMSRNLANRESVILSLHPHNDRGTAVAAAELGFAAGADRIEGCLFGNGERTGNVC
LVTLGLNLFSRGVDPQIDFSNIDEIRRTVEYCNQLPVHERHPYGGDLVYTAFSGSHQDAINKGLD
AMKLDADAADCDVDDMLWQVPYLPIDPRDVGRTYEAV
>3U43A
MELKHSISDYTEAEFLEFVKKICRAEGATEEDDNKLVREFERLTEHPDGSDLIYYPRDDREDSPE
GIVKEIKEWRAANGKSGFKQGLEHHHHHH
>3VIQB
MEKSQLESRVHLLEQQKEQLESSLQDALAKLKNRDAKQTVQKHIDLLHTYNEIRDIALGMIGKVA
EHEKCTSVELFDRFGVNGSE
>3U3EA
MVLSEGEWQLVLHVWAKVEADVAGHGQDILIRLFKSHPETLEKFDRFKHLKTEAEMKASEDLKKH
GVTVLTALGAILKKKGHHEAELKPLAQSHATKHKIPIKYLEFISEAIIHVLHSRHPGDFGADAQG
AMNKALELFRKDIAAKYKELGYQG
>3U2GA
MGTYEIRGQVASGFGDQSWDASSFAGFYYDIDDNVSTETLTVSDLDGNVIPEGGLVYTTTIADVD
FEYYNPDAGWDQYPVMGFFAEEYIPINPDKADKIAKLVLDSDDKYTIRTGEMLDLGEGYAIEAKQ
VDVDGEKVWLEFTKDGEFVDDEIISVSTADDEANTWDVELDDIEDEDDVVVLKVHVNQVFQGAVD
SIAQIEGLWLIDYANAMTIESDDEFGNLDDVSIDGDTLKISNEDTFTLTRDSEEEIGEGMYFMIA
DTSSSDLRYYPYVEKTIGLEHHHHHH
>3U22A
GACDGILEGIYDSPAASDSNELGFIRTDPSTHSGTIYIDATDYRRWTFIDFHTQKVDSVNVTDSE
QKEPEEWDIAVHRYDVKTNAGAVLETGFTGFSALRNADAMPEGAYVEDVWTTAKIAIDMSGMMDG
NIVYMESYYNEELSKWLNVDKSNMPPTYTLSNKVYMVKLKDGTYAAVRLTNYMNASGVKGFMTID
YIYPFEL
>3U0CA
GSQAANDAANKLFSLTIADLTANQNINTTNAHSTSNILIPELKAPKSLNASSQLTLLIGNLIQIL
GEKSLTALTNKITAWKSQQQARQQKNLEFSDKINTLLSETEGLTRDYEKQINKLKNADSKIKDLE
NKINQIQTRLSELDPESPEKKKLSREEIQLTIKKDAAVKDRTLIEQKTLSIHSKLTDKSMQLEKE
IDSFSA
>3U0OA
MSENSIRLTQYSHGAGCGCKISPKVLETILHSEQAKFVDPNLLVGNETRDDAAVYDLGNGTSVIS
TTDFFMPIVDNPFDFGRIAATNAISDIFAMGGKPIMAIAILGWPINKLSPEIAREVTEGGRYACR
QAGIALAGGHSIDAPEPIFGLAVTGIVPTERVKKNSTAQAGCKLFLTKPLGIGVLTTAEKKSLLK
PEHQGLATEVMCRMNIAGASFANIEGVKAMTDVTGFGLLGHLSEMCQGAGVQARVDYEAIPKLPG

VEEYIKLGAVPGGTERNFASYGHLMGEMPREVRDLLCDPQTSGGLLLAVMPEAENEVKATAAEFG
IELTAIGELVPARGGRAMVEIR
>4A2NB
MNENLWKICFIVMFIIWVFVRKVYGTRAMKNKSKKKVRPNFEKSLVFLNFIGMVFLPLTAVFSSY
LDSFNINLPDSIRLFALIVTFLNIGLFTKIHKDLGNNWSAILEIKDGHKLVKEGIYKNIRHPMYA
HLWLWVITQGIILSNWVVLIFGIVAWAILYFIRVPKEEELLIEEFGDEYIEYMGKTGRLFPKVV
>3VIAA
GSMAFVKSGWLLRQSTILKRWKKNWFDLWSDGHLIYYDDQTRQNIEDKVHMPMDCINIRTGQECR
DTQPPDGKSKDCMLQIVCRDGKTISLCAESTDDCLAWKFTLQDSRTN
>4A2AA
MIDLSKTVFYTSIDIGSRYIKGLVLGKRDQEWEALAFSSVKSRGLDEGEIKDAIAFKESVNTLLK
ELEEQLQKSLRSDFVISFSSVSFEREDTVIERDFGEEKRSITLDILSEMQSEALEKLKENGKTPL
HIFSKRYLLDDERIVFNPLDMKASKIAIEYTSIVVPLKVYEMFYNFLQDTVKSPFQLKSSLVSTA
EGVLTTPEKDRGVVVVNLGYNFTGLIAYKNGVPIKISYVPVGMKHVIKDVSAVLDTSFEESERLI
ITHGNAVYNDLKEEEIQYRGLDGNTIKTTTAKKLSVIIHARLREIMSKSKKFFREVEAKIVEEGE
IGIPGGVVLTGGGAKIPRINELATEVFKSPVRTGCYANSDRPSIINADEVANDPSFAAAFGNVFA
VSENPYEETPVKSENPLKKIFRLFKELME
>4A25A
TTIHDVQTTGLTQDAVTGFDASSRLNAGLQEVLVDLTALHLQGKQAHWNIVGENWRDLHLQLDTL
VEAARGFSDDVAERMRAVGGVPDARPQTVAASRIGDVGPDEIDTRACVEAIVALVRHTVDTIRRV
HDPIDAEDPASADLLHAITLELEKQAWMIGSENRSPRRR
>3TWLA
MPELPEVEAARRAIEENCLGKKIKRVIIADDNKVIHGISPSDFQTSILGKTIISARRKGKNLWLE
LDSPPFPSFQFGMAGAIYIKGVAVTKYKRSAVKDSEEWPSKYSKFFVELDDGLELSFTDKRRFAK
VRLLANPTSVSPISELGPDALLEPMTVDEFAESLAKKKITIKPLLLDQGYISGIGNWIADEVLYQ
ARIHPLQTASSLSKEQCEALHTSIKEVIEKAVEVDADSSQFPSNWIFHNREKKPGKAFVDGKKID
FITAGGRTTAYVPELQKLYGKDAEKAAKVRPAKRGVKPKEDDGDHHHHHH
>3TVLA
MAQGLIEVERKFLPGPGTEERLQELGGTLEYRVTFRDTYYDTPELSLMQADHWLRRREDSGWELK
CPGAAGVLGPHTEYKELTAEPTIVAQLCKVLRADGLGAGDVAAVLGPLGLQEVASFVTKRSAWKL
VLLGADEEEPQLRVDLDTADFGYAVGEVEALVHEEAEVPTALEKIHRLSSMLGVPAQETAPAKLI
VYLQRFRPQDYQRLLEVNSSRERPQETEDPDHCLG
>3TV0A
GSSSMAEKTQKSVKIAPGAVVCVESEIRGDVTIGPRTVIHPKARIIAEAGPIVIGEGNLIEEQAL
IINAYPDNITPDTEDPEPKPMIIGTNNVFEVGCYSQAMKMGDNNVIESKAYVGRNVILTSGCIIG
ACCNLNTFEVIPENTVIYGADCLRRVQTERPQPQTLQLDFLMKILPNYHHLKKTMKGSSTPVKN
>3TURA
RRYTLNATALGLGGAATRQLTFQTSSPAHLTMPYVMPGDGEVVGVGEPVAIRFDENIADRGAAEK
AIKITTNPPVEGAFYWLNNREVRWRPEHFWKPGTAVDVAVNTYGVDLGEGMFGEDNVQTHFTIGD
EVIATADDNTKILTVRVNGEVVKSMPTSMGKDSTPTANGIYIVGSRYKHIIMDSSTYGVPVNSPN
GYRTDVDWATQISYSGVFVHSAPWSVGAQGHTNTSHGCLNVSPSNAQWFYDHVKRGDIVEVVNTV
GGTLPGIDGLGDWNIPWDQWRAGNAKA
>3TUNA
GPGSMKVEKVFFVTSPIYYVNAAPHIGHVYSTLITDVIGRYHRVKGERVFALTGTDEHGQKVAEA

AKQKQVSPYDFTTAVAGEFKKCFEQMDYSIDYFIRTTNEQHKAVVKELWTKLEQKGDIYLGRYEG
WYSISDESFLTPQNITDGVDKDGNPCKVSLESGHVVTWVSEENYMFRLSAFRERLLEWYHANPGC
IVPEFRRREVIRAVEKGLPDLSVSRARATLHNWAIPVPGNPDHCVYVWLDALTNYLTGSRLRVDE
SGKEVSLVDDFNELERFPADVHVIGKDILKFHAIYWPAFLLSAGLPLPKKIVAHGWWTKDRKKIS
KSLGNVFDPVEKAEEFGYDALKYFLLRESGFSDDGDYSDKNMIARLNGELADTLGNLVMRCTSAK
INVNGEWPSPAAYTEEDESLIQLIKDLPGTADHYYLIPDIQKAIIAVFDVLRAINAYVTDMAPWK
LVKTDPERLRTVLYITLEGVRVTTLLLSPILPRKSVVIFDMLGVPEVHRKGIENFEFGAVPPGTR
LGPAVEGEVLFSKRSTENTKST

>3TU5A

MCDEDETTALVCDNGSGLVKAGFAGDDAPRAVFPSIVGRPRHQGVMVGMGQKDSYVGDEAQSKRG
ILTLKYPIEHGIITNWDDMEKIWHHTFYNELRVAPEEHPTLLTEAPLNPKANREKMTQIMFETFN
VPAMYVAIQAVLSLYASGRTTGIVLDSGDGVTHNVPIYEGYALPHAIMRLDLAGRDLTDYLMKIL
TERGYSFVTTAEREIVRDIKEKLCYVALDFENEMATAASSSSLEKSYELPDGQVITIGNERFRCP
ETLFQPSFIGMESAGIHETTYNSIMKCDIDIRKDLYANNVMSGGTTMYPGIADRMQKEITALAPS
TMKIKIIAPPERKYSVWIGGSILASLSTFQQMWITKQEYDEAGPSIVHRKCF

>3TU3A

MHHHHHHSSGVDLGTENLYFQSNAMIDTWLAQWGLRLPSSNDATLRLQPAEGPELVMERLEGGWL
FVVELGLVPSGLPLGVILQLLQVNSPFSSLAPVKLAADDAGRLVLWAEARDGVDDVDALNRLHDR
LREGHSRLVPLLEPTGELVPAQIQTSALVFV

>3TT1A

MEVKREHWATRLGLILAMAGNAVGLGNFLRFPVQAAENGGGAFMIPYIIAFLLVGIPLMWIEWAM
GRYGGAQGHGTTPAIFYLLWRNRFAKILGVFGLWIPLVVAIYFVYIESWTLGFAIKFLVGLVPEP
PPNATDPDSILRPFKEFLYSYIGVPKGDEPILKPSLFAYIVFLITMFINVSILIRGISKGIERFA
KIAMPTLFILAVFLVIRVFLLETPNGTAADGLNFLWTPDFEKLKDPGVWIAAVGQIFFTLSLGFG
AIITYASYVRKDQDIVLSGLTAATLNEAAEVILGGSISIPAAVAFFGVANAVAIAKAGAFNLGFI
TLPAIFSQTAGGTFLGFLWFFLLFFAGLTSSIAIMQPMIAFLEDELKLSRKHAVLWTAAIVFFSA
HLVMFLNKSLDEMDFWAGTIGVVFFGLTELIIFFWIFGADKAWEEINRGGIIKVPRIYYYVMRYI
TPAFLAVLLVVWAREYIPKIMEETHWTVWITRFYIIGLFLFLTFLVFLAERRRNHESAGTLVPR

>4A0XA

MRGETLKLKKDKRREAIRQQIDSNPFITDHELSDLFQVSIQTIRLDRTYLNIPELRKRIKLVAEK
NYDQISSIEEQEFIGDLIQVNPNVKAQSILDITSDSVFHKTGIARGHVLFAQANSLCVALIKQPT
VLTHESSIQFIEKVKLNDTVRAEARVVNQTAKHYYVEVKSYVKHTLVFKGNFKMFYDKRG

>3TS9A

GHMDTRENPFKEKLLEIMASIQTYCQKSPMSDFGTQHYEQWAIQMEKKAAKDGNRKDRVCAEHLR
KYNEALQINDTIRMIDAYSHLETFYTDEKEKKFAVLNDSKKSLKLDETDEFLMNLFFDNKKMLKK
LAENPKYE

>3VHXB

GSLLFQPDQNAPPIRLRHRRSRSAGDRWVDHKPASNMQTETVMQPHVPHAITVSVANEKALAKCE
KYMLTHQELASDGEIETKLIKGDIYKTRGGGQSVQFTDIETLKQESPNGSRKRRS

>3TRTA

GGSKPDCTAAMRDVRQQYESVAAKNLQEAEEWYKSKFADLSEAANRNNDALRQAKQESTEYRRQV
QSLTMEVDALKG

>4A0EA

GGGSWVCRFYQGKHRGVEVELPHGRCVFGSDPLQSDIVLSDSEIAPVHLVLMVDEEGIRLTDSAE

PLLQEGLPVPLGTLLRAGSCLEVGFLLWTFVAVGQPLPETLQVPTQRKEPTDRLPRSR
>4A03A
TMAHHHHHHVTNSTDGRADGRLRVVVLGSTGSIGTQALQVIADNPDRFEVVGLAAGGAHLDTLLR
QRAQTGVTNIAVADEHAAQRVGDIPYHGSDAATRLVEQTEADVVLNALVGALGLRPTLAALKTGA
RLALANKESLVAGGSLVLRAARPGQIVPVDSEHSALAQCLRGGTPDEVAKLVLTASGGPFRGWSA
ADLEHVTPEQAGAHPTWSMGPMNTLNSASLVNKGLEVIETHLLFGIPYDRIDVVVHPQSIIHSMV
TFIDGSTIAQASPPDMKLPISLALGWPRRVSGAAAACDFHTASSWEFEPLDTDVFPAVELARQAG
VAGGCMTAVYNAANEEAAAAFLAGRIGFPAIVGIIADVLHAADQWAVEPATVDDVLDAQRWARER
AQRAVSGM
>3TO8A
MGHHHHHHDSPVLMVYGLDQSKMNCDRVFNVFCLYGNVEKVKFMKSKPGAAMVEMADGYAVDRAI
THLNNNFMFGQKLNVCVSKQPAIMPGQSYGLEDGSCSYKDFSESRNNRFSTPEQAAKNRIQHPSN
VLHFFNAPLEVTEENFFEICDELGVKRPSSVKVFSGKSERSSSGLLEWESKSDALETLGFLNHYQ
MKNPNGPYPYTLKLCFSTAQHAS
>3TNXA
MHHHHHHSSGLVPRGSGMKETAAAKFERQHMDSPDLGTDDDDKMDFSIVGYSQNDLTSTERLIQL
FESWMLKHNKIYKNIDEKIYRFEIFKDNLKYIDETNKKNNSYWLGLNVFADMSNDEFKEKYTGSI
AGNYTTTELSYEEVLNDGDVNIPEYVDWRQKGAVTPVKNQGSCGSAWAFSAVSTIESIIKIRTGN
LNEYSEQELLDCDRRSYGCNGGYPWSALQLVAQYGIHYRNTYPYEGVQRYCRSREKGPYAAKTDG
VRQVQPYNEGALLYSIANQPVSVVLEAAGKDFQLYRGGIFVGPCGNKVDHAVAAVGYGPNYILIR
NSWGTGWGENGYIRIKRGTGNSYGVCGLYTSSFYPVKN
>3ZZOA
ECCTSRELVEFKMDRGDCEAVRAIENYPNGCEVTICADGVAQLGAYCGQGPCNIFGCNCDGGCLS
GDWSQEFVRRNQQYGIQIIKVTRLPFWRPL
>3TMUA
MRSLLILVLCFLPLAALGKVFGRCELAAAMKRHGLDNYRGYSLGNWVCAAKFESNFNTQATNRNT
DGSTDYGILQINSRWWCNDGRTPGSRNLCNIPCSALLSSDITASVNCAKKIVSDGNGMNAWVAWR
NRCKGTDVQAWIRGCRL
>3TKLB
GPLGSTSSTSQADKEIQKMLDEYEQAIKRAQENIKKGEELEKKLDKLERQGKDLEDKYKTYEENL
EGFEKLLTDSEELSLSEINEKMKAFSKDSEKLTQLMEKHKGDEKTVQSLQREHHDIKAKLANLQV
LHDAHTGKKSYVNEKGNPVSSLKDAHLAINKDQEVVEHKGQFYLLQKGQWDAIKNDPAALEKAQK
DYSQSKHDLATIKMEALIHKLSLEMEKQLETINDLIMSTDPKENEEATKLLHKHNGLNLKLANLQ
DMLAVHR
>3TIKA
KGKLPPVYPVTVPILGHIIQFGKSPLGFMQECKRQLKSGIFTINIVGKRVTIVGDPHEHSRFFLP
RNEVLSPREVYSFMVPVFGEGVAYAAPYPRMREQLNFLAEELTIAKFQNFVPAIQHEVRKFMAAN
WDKDEGEINLLEDCSTMIINTACQCLFGEDLRKRLDARRFAQLLAKMESSLIPAAVFLPILLKLP
LPQSARCHEARTELQKILSEIIIARKEEEVNKDSSTSDLLSGLLSAVYRDGTPMSLHEVCGMIVA
AMFAGQHTSSITTTWSMLHLMHPANVKHLEALRKEIEEFPAQLNYNNVMDEMPFAERCARESIRR
DPPLLMLMRKVMADVKVGSYVVPKGDIIACSPLLSHHDEEAFPEPRRWDPERDEKVEGAFIGFGA
GVHKCIGQKFGLLQVKTILATAFRSYDFQLLRDEVPDPDYHTMVVGPTASQCRVKYIRRKAAAA
>3TH0A
YDPDQYSIEADKKFKYSVKLSDYPTLQDAASAAVDGLLIDRDYNFYGGETVDFGGKVLTIECKAK

FIGDGNLIFTKLGKGSRIAGVFMESTTTPWVIKPWTDDNQWLTDAAAVVATLKQSKTDGYQPTVS
DYVKFPGIETLLPPNAKGQNITSTLEIRECIGVEVHRASGLMAGFLFRGCHFCKMVDANNPSGGK
DGIITFENLSGDWGKGNYVIGGRTSYGSVSSAQFLRNNGGFERDGGVIGFTSYRAGESGVKTWQG
TVGSTTSRNYNLQFRDSVVIYPVWDGFDLGADTDMNPELDRPGDYPITQYPLHQLPLNHLIDNLL
VRGALGVGFGMDGKGMYVSNITVEDCAGSGAYLLTHESVFTNIAIIDTNTKDFQANQIYISGACR
VNGLRLIGIRSTDGQSLTIDAPNSTVSGITGMVDPSRINVANLAEEGLGNIRANSFGYDSAAIKL
RIHKLSKTLDSGALYSHINGGAGSGSAYTQLTAISGSTPDAVSLKVNHKDCRGAEIPFVPDIASD
DFIKDSSCFLPYWENNSTSLKALVKKPNGELVRLTLATL

>3TGUB

SLKVAPKVAVSAAAERVKLCPGAEDLEITKLPNGLIIASLENFSPASRIGVFIKAGSRYETTANL
GTAHLLRLASPLTTKGASSFRITRGIEAVGGSLSVYSTREKMTYCVECLRDHVDTVMEYLLNVTT
APEFRPWEVTDLQPQLKVDKAVAFQSPQVGVLENLHAAAYKTALANPLYCPDYRIGKITSEQLHH
FVQNNFTSARMALVGIGVKHSDLKQVAEQFLNIRSGAGTSSAKATYWGGEIREQNGHSLVHAAVV
TEGAAVGSAEANAFSVLQHVLGAGPLIKRGSSVTSKLYQGVAKATTQPFDASAFNVNYSDSGLFG
FYTISQAAHAGEVIRAAMNQLKAAAQGGVTEEDVTKAKNQLKATYLMSVETAQGLLNEIGSEALL
SGTHTAPSVVAQKIDSVTSADVVNAAKKFVSGKKSMAASGDLGSTPFLDEL

>3ZXUB

MDFTSSSGVLDSERNTGSNDSDEPSSHSDVIETEELKLIKLQEHKNNLLRQRSELLDQLSQTRVV
EPRSVQLDDKLLLKLLRRNDNAVSDSSQSSNNPLPRVLPSLNIEQRKKYLDITLNDVTVTCEKDM
ILLRKGSFTASFRIAVENESIRSMAIDLNAFEVELQPIIQYAEDTQNVNVAMMAVVQFLRIKELH
EQMISKIVEASKFIRASNNTITLNDLEVSFHCYWNLPSPYPETLILTNKVQKILDFLIYQYGIQL
GVIKYGSTII

>2LHRA

SDDYVDEETYNLQKLLAPYHKAKTLERQVYELEKLQEKLPEKYKAEYKKKLDQTRVELADQVKSA
VTEFENVTPTNDQ

>3TEEA

QDINAQLTTWFSQRLAGFSDEVVVTLRSSPNLLPSCEQPAFSMTGSAKLWGNVNVVARCANEKRY
LQVNVQATGNYVAVAAPIARGGKLTPANVTLKRGRLDQLPPRTVLDIRQIQDAVSLRDLAPGQPV
QLTMIRQAWRVKAGQRVQVIANGEGFSVNAEGQAMNNAAVAQNARVRMTSGQIVSGTVDSDGNIL
INLDPNSSSVDKLAAALEHHHHHH

>3TDOA

MGRAHKETLDKLTNAAINKINLLNTSKVKYLVSSAFAGLYVGIGILLIFTIGGLLTDAGSPMTKI
VMGLSFAIALSLVIMTGTELFTGNNMVMSAGMLNKGVSIKDTSKIWAYSWVGNLIGALVLGIIFV
GTGLVDKGPVAEFFANTAASKASMPFTALFFRGILCNILVCVSVLCSFRTNSDTAKIIMIFLCLF
AFITSGFEHSVANMTIYSVSLFSPTISTVTIGGAIYNLVAVTLGNIVGGALFMGLGTYILGKEKL
NAAAENLY

>3TCJA

MSQFTLYKNKDKSSAKTYPYFVDVQSDLLDNLNTRLVIPLTPIELLDKKAPSHLCPTIHIDEGDF
IMLTQQMTSVPVKILSEPVNELSTFRNEIIAAIDFLITGI

>3ZXBA

FTCPECRPELCGDPGYCEYGTTKDACDCCPVCFQGPGGYCGGPEDVFGICADGFACVPLVGERDS
QDPEIVGTCVKIP

>3VGBA

TFAYKIDGNEVIFTLWAPYQKSVKLKVLEKGLYEMERDEKGYFTITLNNVKVRDRYKYVLDDASE

IPDPASRYQPEGVHGPSQIIQESKEFNNETFLKKEDLIIYEIHVGTFTPEGTFEGVIRKLDYLKD
LGITAIEIMPIAQFPGKRDWGYDGVYLYAVQNSYGGPEGFRKLVDEAHKKGLGVILDVVYNHVGP
EGNYMVKLGPYFSQKYKTPWGLTFNFDDAESDEVRKFILENVEYWIKEYNVDGFRLDAVHAIIDT
SPKHILEEIADVVHKYNRIVIAESDLNDPRVVNPKEKCGYNIDAQWVDDFHHSIHAYLTGERQGY
YTDFGNLDDIVKSYKDVFVYDGKYSNFRRKTHGEPVGELDGCNFVVYIQNHDQVGNRGKGERIIK
LVDRESYKIAAALYLLSPYIPMIFMGEEYGEENPFYFFSDFSDSKLIQGVREGRKKENGQDTDPQ
DESTFNASKLSWKIDEEIFSFYKILIKMRKELSIACDRRVNVVNGENWLIIKGREYFSLYVFSKS
SIEVKYSGTLLLSSNNSFPQHIEEGKYEFDKGFALYKL
>3ZX8A
MENDPRVRKFASDGAQWAIKWQKKGWSTLTSRQKQTARAAMGIKLSPVAQPVQKVTRLSAPVALA
YREVSTQPRVSTARDGITRSGSELITTLKKNTDTEPKYTTAVLNPSEPGTFNQLIKEAAQYEKYR
FTSLRFRYSPMSPSTTGGKVALAFDRDAAKPPPNDLASLYNIEGCVSSVPWTGFILTVPTDSTDR
FVADGISDPKLVDFGKLIMATYGQGAAQLGEVRVEYTVQLKNRTGSTSAQIGDFAGVKDGPRLVS
WSKTKGTAGWEHDCHFLGTGNFSLTLFYEKAPVSGLENADASDFSVLGEAAAGSVQWAGVKVAER
GQGVKMVTTEEQPKGKWQALRI
>3TBLA
MFEARLVQGSILKKVLEALKDLINEACWDISSSGVNLQSMDSSHVSLVQLTLRSEGFDTYRCDRN
LAMGVNLTSMSKILKCAGNEDIITLRAEDNADTLALVFEAPNQEKVSDYEMKLMDLDVEQLGIPE
QEYSCVVKMPSGEFARICRDLSHIGDAVVISCAKDGVKFSASGELGNGNIKLSQTSNVDKEEEAV
TIEMNEPVQLTFALRYLNFFTKATPLSSTVTLSMSADVPLVVEYKIADMGHLKYYLAPKIEDEEG
S
>3TAYA
GSLLDGPYQPTTFNPPTSYWILLAPTVEGVVIQGTNNIDRWLATILIEPNVQTTNRIYNLFGQQV
TLSVENTSQTQWKFIDVSKTTPTGNYTQHGSLFSTPKLYAVMKFSGRIYTYNGTTPNATTGYYST
TNYDTVNMTSFCDFYIIPRNQEEKCTEYINHGL
>3ZWSA
MATGDERFYAEHLMPTLQGLLDPESAHRLAVRFTSLGLLPRARFQDSDMLEVRVLGHKFRNPVGI
AAGFDKHGEAVDGLYKMGFGFVEIGSVTPKPQEGNPRPRVFRLPEDQAVINRYGFNSHGLSVVEH
RLRARQQKQAKLTEDGLPLGVNLGKNKTSVDAAEDYAEGVRVLGPLADYLVVNVSSPNTAGLRSL
QGKAELRRLLTKVLQERDGLRRVHRPAVLVKIAPDLTSQDKEDIASVVKELGIDGLIVTNTTVSR
PAGLQGALRSETGGLSGKPLRDLSTQTIREMYALTQGRVPIIGVGGVSSGQDALEKIRAGASLVQ
LYTALTFWGPPVVGKVKRELEALLKEQGFGGVTDAIGADHRR
>3T60A
MHLKIVCLSDEVREMYKNHKTHHEGDSGLDLFIVKDEVLKPKSTTFVKLGIKAIALQYKSNYYYK
CEKSENKKKDDDKSNIVNTSFLLFPRSSISKTPLRLANSIGLIDAGYRGEIIAALDNTSDQEYHI
KKNDKLVQLVSFTGEPLSFELVEELDETSRGEGGFGSTSNNKYLEHHHHHH
>3T5VA
GSPLPSDVRPPHILVKTLDYIVDNLLTTLPESEGFLWDRMRSIRQDFTYQNYSGPEAVDCNERIV
RIHLLILHIMVKSNVEFSLQQELEQLHKSLITLSEIYDDVRSSGGTCPNEAEFRAYALLSKIRDP
QYDENIQRLPKHIFQDKLVQMALCFRRVISNSAYTERGFVKTENCLNFYARFFQLMQSPSLPLLM
GFFLQMHLTDIRFYALRALSHTLNKKHKPIPFIYLENMLLFNNRQEIIEFCNYYSIEIINGDAAD
LKTLQHYSHKLSETQPLKKTYLTCLERRLQKTTYKGLINGGEDNLASSVYVKDPKK
>3T63M
PAQDNSRFVIRDRNWHPKALTPDYKTSIARSPRQALVSIPQSISETTGPNFSHLGFGAHDHDLLL

NFNNGGLPIGERIIVAGRVVDQYGKPVPNTLVEMWQANAGGRYRHKNDRYLAPLDPNFGGVGRCL
TDSDGYYSFRTIKPGPAPWRNGPNDWRPAHIYFGISGPSIATKLITQLYFEGDPLIPMCPIVKSI
ANPEAVQQLIAKLDMNNANPMDCLAYRFDIVLRGQRKTHFENC
>3T6BA
MADTQYILPNDIGVSSLDCREAFRLLSPTERLYAYHLSRAAWYGGLAVLLQTSPEAPYIYALLSR
LFRAQDPDQLRQHALAEGLTEEEYQAFLVYAAGVYSNMGNYKSFGDTKFVPNLPKEKLERVILGS
EAAQQHPEEVRGLWQTCGELMFSLEPRLRHLGLGKEGITTYFSGNCTMEDAKLAQDFLDSQNLSA
YNTRLFKEVDGEGKPYYEVRLASVLGSEPSLDSEVTSKLKSYEFRGSPFQVTRGDYAPILQKVVE
QLEKAKAYAANSHQGQMLAQYIESFTQGSIEAHKRGSRFWIQDKGPIVESYIGFIESYRDPFGSR
GEFEGFVAVVNKAMSAKFERLVASAEQLLKELPWPPTFEKDKFLTPDFTSLDVLTFAGSGIPAGI
NIPNYDDLRQTEGFKNVSLGNVLAVAYATQREKLTFLEEDDKDLYILWKGPSFDVQVGLHALLGH
GSGKLFVQDEKGAFNFDQETVINPETGEQIQSWYRSGETWDSKFSTIASSYEECRAESVGLYLCL
HPQVLEIFGFEGADAEDVIYVNWLNMVRAGLLALEFYTPEAFNWRQAHMQARFVILRVLLEAGEG
LVTITPTTGSDGRPDARVRLDRSKIRSVGKPALERFLRRLQVLKSTGDVAGGRALYEGYATVTDA
PPECFLTLRDTVLLRKESRKLIVQPNTRLEGSDVQLLEYEASAAGLIRSFSERFPEDGPELEEIL
TQLATADARFW
>3T5AA
MGSSHHHHHHSSGLVPRGSHMSVRSLPAALRACARLQPHDPAFTFMDYEQDWDGVAITLTWSQLY
RRTLNVAQELSRCGSTGDRVVISAPQGLEYVVAFLGALQAGRIAVPLSVPQGGVTDERSDSVLSD
SSPVAILTTSSAVDDVVQHVARRPGESPPSIIEVDLLDLDAPNGYTFKEDEYPSTAYLQYTSGST
RTPAGVVMSHQNVRVNFEQLMSGYFADTDGIPPPNSALVSWLPFYHDMGLVIGICAPILGGYPAV
LTSPVSFLQRPARWMHLMASDFHAFSAAPNFAFELAARRTTDDDMAGRDLGNILTILSGSERVQA
ATIKRFADRFARFNLQERVIRPSYWLAEATVYVATSKPGQPPETVDFDTESLSAGHAKPCAGGGA
TSLISYMLPRSPIVRIVDSDTCIECPDGTVGEIWVHGDNVANGYWQKPDESERTFGGKIVTPSPG
TPEGPWLRTGDSGFVTDGKMFIIGR
>2LGDA
GSMALPIIVKWGGQEYSVTTLSEDDTVLDLKQFLKTLTGVLPERQKLLGLKVKGKPAENDVKLGA
LKLKPNTKIMMMGTREES
>3T1HR
MSTKNAKPKKEAQRRPSRKAKVKATLGEFDLRDYRNVEVLKRFLSETGKILPRRRTGLSGKEQRI
LAKTIKRARILGLLPFTEKLVRK
>3T1HQ
MPKKVLTGVVVSDKMQKTVTVLVERQFPHPLYGKVIKRSKKYLAHDPEEKYKLGDVVEIIESRPI
SKRKRFRVLRLVESGRMDLVEKYLIRRQNYQSLSKRGGKA
>3ZUIA
DSESDCTGSEPVDAFQAFSEGKEAYVLVRSTDPKARDCLKGEPAGEKQDNTLPVMMTFKNGTDWA
STDWTFTLDGAKVTATLGNLTQNREVVYDSQSHHCHVDKVEKEVPDYEMWMLDAGGLEVEVECCR
QKLEELASGRNQMYPHLKDC
>3SX6A
MRGSAHVVILGAGTGGMPAAYEMKEALGSGHEVTLISANDYFQFVPSNPWVGVGWKERDDIAFPI
RHYVERKGIHFIAQSAEQIDAEAQNITLADGNTVHYDYLMIATGPKLAFENVPGSDPHEGPVQSI
CTVDHAERAFAEYQALLREPGPIVIGAMAGASCFGPAYEYAMIVASDLKKRGMRDKIPSFTFITS
EPYIGHLGIQGVGDSKGILTKGLKEEGIEAYTNCKVTKVEDNKMYVTQVDEKGETIKEMVLPVKF
GMMIPAFKGVPAVAGVEGLCNPGGFVLVDEHQRSKKYANIFAAGIAIAIPPVETTPVPTGAPKTG

YMIESMVSAAVHNIKADLEGRKGEQTMGTWNAVAFADMGDRGAAFIALPQLKPRKVDVFAYGRWV
HLAKVAFEKYFIRKMKMGVSEPFYEKVLFKMMGITRLKEEDTHRKAS
>3SWMA
HHHHHHMGIQETDPLTQLSLPPGFRFYPTDEELMVQYLCRKAAGYDFSLQLIAEIDLYKFDPWVL
PNKALFGEKEWYFFSPRDRKYPNGSRPNRVAGSGYWKATGTDKIISTEGQRVGIKKALVFYIGKA
PKGTKTNWIMHEYRLIEPSRRNGSTKLDDWVLCRIYKKQSSAQK
>3STTA
GSMEKSMSPFVKKHFVLVHTAFHGAWCWYKIVALMRSSGHNVTALDLGASGINPKQALQIPNFSD
YLSPLMEFMASLPANEKIILVGHALGGLAISKAMETFPEKISVAVFLSGLMPGPNIDATTVCTKA
GSAVLGQLDNCVTYENGPTNPPTTLIAGPKFLATNVYHLSPIEDLALATALVRPLYLYLAEDISK
EVVLSSKRYGSVKRVFIVATENDALKKEFLKLMIEKNPPDEVKEIEGSDHVTMMSKPQQLFTTLL
SIANKYK
>3SS3A
GSHMVAAGDNKIKQGLLPSLEDLLFYTIAEGQEKIPVHKFITALKSTGLRTSDPRLKECMDMLRL
TLQTTSDGVMLDKDLFKKCVQSNIVLLTQAFRRKFVIPDFMSFTSHIDELYESAKKQSGGKVADY
IPQLAKFSPDLWGVSVCTVDGQRHSIGDTKVPFCLQSCVKPLKYAIAVNDLGTEYVHRYVGKEPS
GLRFNKLFLNEDDKPHNPMVNAGAIVVTSLIKQGVNNAEKFDYVMQFLNKMAGNEYVGFSNATFQ
SERESGDRNFAIGYYLKEKKCFPEGTDMVGILDFYFQLCSIEVTCESASVMAATLANGGFCPITG
ERVLSPEAVRNTLSLMHSCGMYDFSGQFAFHVGLPAKSGVAGGILLVVPNVMGMMCWSPPLDKMG
NSVKGIHFCHDLVSLCNFHNYDNLRHFAKKLDPRREGGDQRHSFGPLDYESLQQELALKDTVWKK
VSPESSDDTSTTVVYRMESLGERS
>3SQRA
MKYFTVFTALTALFAQASASAIPAVRSTLTPRQNTTASCANSATSRSCWGEYSIDTNWYDVTPTG
VTREYWLSVENSTITPDGYTRSAMTFNGTVPGPAIIADWGDNLIIHVTNNLEHNGTSIHWHGIRQ
LGSLEYDGVPGVTQCPIAPGDTLTYKFQVTQYGTTWYHSHFSLQYGDGLFGPLIINGPATADYDE
DVGVIFLQDWAHESVFEIWDTARLGAPPALENTLMNGTNTFDCSASTDPNCVGGGKKFELTFVEG
TKYRLRLINVGIDSHFEFAIDNHTLTVIANDLVPIVPYTTDTLLIGIGQRYDVIVEANAAADNYW
IRGNWGTTCSTNNEAANATGILRYDSSSIANPTSVGTTPRGTCEDEPVASLVPHLALDVGGYSLV
DEQVSSAFTNYFTWTINSSSLLLDWSSPTTLKIFNNETIFPTEYNVVALEQTNANEEWVVYVIED
LTGFGIWHPIHLHGHDFFIVAQETDVFNSDESPAKFNLVNPPRRDVAALPGNGYLAIAFKLDNPG
SWLLHCHIAWHASEGLAMQFVESQSSIAVKMTDTAIFEDTCANWNAYTPTQLFAEDDSGI
>3SO6A
MEGMVFSLKYLGMTLVERPKGEELSAAAVKRIVATAKASGKKLQKVTLKVSPRGIILTDSLTSQL
IENVSIYRISYCTADKMHDKVFAYIAQSQQNESLECHAFLCTKRKVAQAVTLTVAQAFKVAFEFW
QVSLVPR
>3SMHA
SRNNPFYFPSRRFSTRYGNQNGRIRVLQRFDQRSRQFQNLQNHRIVQIEAKPNTLVLPKHADADN
ILVIQQGQATVTVANGNNRKSFNLDEGHALRIPSGFISYILNRHDNQNLRVAKISMPVNTPGQFE
DFFPASSRDQSSYLQGFSRNTLEAAFNAEFNEIRRVLLEENAGGEQEERGQRRWSTRSSENNEGV
IVKVSKEHVEELTKHAKSVSKKGSEEEGDITNPINLREGEPDLSNNFGKLFEVKPDKKNPQLQDL
DMMLTCVEIKEGALVLPHFNSKAMVIVVVNKGTGNLELVAVRKEQQQRGRREEEEDEDEEEEGSN
REVRRYTARLKEGDVFIMPAAHPVAINASSELHLLGFGINAENNHRIFLAGDKDNVIDQIEKQAK
DLAFPGSGEQVEKLIKNQKESHFVSARP
>3B18A

MSHTDLTPCTRVLASSGTVPIAEELLARVLEPYSCKGCRYLIDAQYSATEDSVLAYGNFTIGESA
YIRSTGHFNAVELILCFNQLAYSAFAPAVLNEEIRVLRGWSIDDYCQHQLSSMLIRKASSRFRKP
LNPQKFSARLLCRDLQVIERTWRYLKVPCVIEFWDENGGAASGEIELAALNIP
>3ZSCA
SLNDKPVGFASVPTADLPEGTVGGLGGEIVFVRTAEELEKYTTAEGKYVIVVDGTIVFEPKREIK
VLSDKTIVGINDAKIVGGGLVIKDAQNVIIRNIHFEGFYMEDDPRGKKYDFDYINVENSHHIWID
HITFVNGNDGAVDIKKYSNYITVSWNKFVDHDKVSLVGSSDKEDPEQAGQAYKVTYHHNYFKNLI
QRMPRIRFGMAHVFNNFYSMGLRTGVSGNVFPIYGVASAMGAKVHVEGNYFMGYGAVMAEAGIAF
LPTRIMGPVEGYLTLGEGDAKNEFYYCKEPEVRPVEEGKPALDPREYYDYTLDPVQDVPKIVVDG
AGAGKLVFEELNTAQ
>2LENA
MQLKPMEINPEMLNKVLYRLGVAGQWRFVDVLGLEEESLGSVPAPACALLLLFPLTAQHENFRKK
QIEELKGQEVSPKVYFMKQTIGNSCGTIGLIHAVANNQDKLGFEDGSVLKQFLSETEKMSPEDRA
KCFEKNEAIQAAHDAVAQEGQCRVDDKVNFHFILFNNVDGHLYELDGRMPFPVNHGASSEDTLLK
DAAKVCREFTEREQGEVRFSAVALCKAALEHHHHHH
>2LELA
VDMSNVVKTYDLQDGSKVHVFKDGKMGMENKFGKSMNMPEGKVMETRDGTKIIMKGNEIFRLDEA
LRKGHSEGG
>3SGZA
PLVCLADFKAHAQKQLSKTSWDFIEGEADDGITYSENIAAFKRIRLRPRYLRDMSKVDTRTTIQG
QEISAPICISPTAFHSIAWPDGEKSTARAAQEANICYVISSYASYSLEDIVAAAPEGFRWFQLYM
KSDWDFNKQMVQRAEALGFKALVITIDTPVLGNRRRDKRNQLNLEANILKAALRALKEEKPTQSV
PVLFPKASFCWNDLSLLQSITRLPIILKGILTKEDAELAMKHNVQGIVVSNHGGRQLDEVSASID
ALREVVAAVKGKIEVYMDGGVRTGTDVLKALALGARCIFLGRPILWGLACKGEDGVKEVLDILTA
ELHRCMTLSGCQSVAEISPDLIQFSRL
>3SGFY
MAAKIRRDDEVIVLTGKDKGKRGKVKNVLSSGKVIVEGINLVKKHQKPVPALNQPGGIVEKEAAI
QVSNVAIFNAATGKADRVGFRFEDGKKVRFFKSNSETIK
>3ZQDA
GRKLLTYQVKQGDTLNSIAADFRISTAALLQANPSLQAGLTAGQSIVIPGLPDPYTIPYHIAVSI
GAKTLTLSLNNRVMKTYPIAVGKILTQTPTGEFYIINRQRNPGGPFGAYWLSLSKQHYGIHGTNN
PASIGKAVSKGCIRMHNKDVIELASIVPNGTRVTINRGSHHHHHH
>3SCIE
RVVPSGDVVRFPNITNLCPFGEVFNATKFPSVYAWERKKISNCVADYSVLYNSTFFSTFKCYGVS
ATKLNDLCFSNVYADSFVVKGDDVRQIAPGQTGVIADYNYKLPDDFMGCVLAWNTRNIDATSTGN
YNYKYRFLRHGKLRPFERDISNVPFSPDGKPCTPPAFNCYWPLNDYGFYTTTGIGYQPYRVVVLS
FELLNAPATVCGPKLSTDLIKNQCVNFHHHHHH
>2YMAA
GSIGSNSIDLITKYEPIFLGSGIYFLRPFNTDERDKLMVTDNAMSNWDEITETYYQKFGNAINKM
LSLRLVSLPNGHILQPGDSCVWLAEVVDMKDRFQTTLSLNILNSQRAEIFFNKTFTFNEDNGNFL
SYKIGDHGESTELGQITHSNKADINTAEIRS
>3SBSA
MNTVPFTSAPIEVTIGIDQYSFNVKENQPFHGIKDIPIGHVHVIHFQHADNSSMRYGYWFDCRMG
NFYIQYDPKDGLYKMMEERDGAKFENIVHNFKERQMMVSYPKIDEDDTWYNLTEFVQMDKIRKIV

RKDENQFSYVDSSMTTVQENELLKSSLQKAGSKMEAKNEDDPAHSLNYTVINFKSREAIRPGHEM
EDFLDKSYYLNTVMLQGIFKNSSNYFGELQFAFLNAMFFGNYGSSLQWHAMIELICSSATVPKHM
LDKLDEILYYQIKTLPEQYSDILLNERVWNICLYSSFQKNSLHNTEKIMENKYPELLGKDNEDDA
LIYGISDEERDDEDDEHNPTIVGGLYYQRPLEHHHHHH
>2YJGA
MANIEIPYGKSKLAFDLPDERIQGILRSKAGSYKVNMSEEDIVKRALENPIGTKRLQDLAEGKKN
IVIITSDHTRPVPSRITLPLLLDEIRKKNKSANVKILIATGFHRGTTLQEMKAKFGEDLVENEQF
VVHDSRNSENMELIGTLPSGGKLEINKLAVEADLLVAEGFIEPHFFAGFSGGRKSILPGIASVQC
ILANHCSEFIKNPYARTGVLENNPIHRDMIYAAKKANLAFILNVVIDSSHKIVNAFAGHSEKAHL
KGCEFVSEIATVNAKPADIVITSNGGYPLDQNIYQSVKGMTAGEAACKDGGVIIIAAECADGHGG
EGFYRWFKESKDPQDVMNKILSRGRDETLPDQWEAQILARILINHKVIMVTDSKNYEYVKDMFMT
PAKDLGEALKIAESIVNNDSKINVIPDGVSVIVREKASWSHPQFEK
>3S4EA
ASQVGVIKPWLLLGSQDAAHDLDTLKKNKVTHILNVAYGVENAFLSDFTYKSISILDLPETNILS
YFPECFEFIEEAKRKDGVVLVHSNAGVSRAAAIVIGFLMNSEQTSFTSAFSLVKNARPSICPNSG
FMEQLRTYQEGKES
>3S44A
MKTITLYLDPASLPALNQLMDFTQNNEDKTHPRIFGLSRFKIPDNIITQYQNIHFVELKDNRPTE
ALFTILDQYPGNIELNIHLNIAHSVQLIRPILAYRFKHLDRVSIQQLNLYDDGSDEYVDLEKEEN
KDISAEIKQAEKQLSHYLLTGKIKFDNPTIARYVWQSAFPVKYHFLSTDYFEKAEFLQPLKEYLA
ENYQKMDWTAYQQLTPEQQAFYLTLVGFNDEVKQSLEVQQAKFIFTGTTTWEGNTDVREYYAQQQ
LNLLNHFTQAEGDLFIGDHYKIYFKGHPRGGEINDYILNNAKNITNIPANISFEVLMMTGLLPDK
VGGVASSLYFSLPKEKISHIIFTSNKQVKSKEDALNNPYVKVMRRLGIIDESQVIFWDSLKQLGG
GLEHHHHHH
>3S2SA
MGSSHHHHHHSSGLVPRGSHMASMTGGQQMGRGSMSKALISIDYTYDFVADDGKLTAGKPAQAIS
KAIAQVTQKAYDNGDYIFFTIDGHDEGDDFHPETKLFPPHNIKGTSGRDLYGALADFYQKHENDK
RVFWMDKRHYSAFSGTDLDIRLRERRVDTVVLTGVLTDICVLHTAIDAYNLGYQIEVVQSAVASL
SQENHQFALNHLQNVLGATIIE
>3S2QA
GSHMRKQQRMVVVRAEGGGGINPEIRKNEDKVVDSVVVTELSKNITPYCRCWRSGTFPLCDGSHV
KHNKANGDNVGPLLLKKQ
>3S0PA
ATKKAVAVLKGNSNVEGVVTLSQDDDGPTTVNVRITGLAPGLHGFHLHEYGDTTNGCMSTGAHFN
PNKLTHGAPGDEIRHAGDLGNIVANADGVAEVTLVDNQIPLTGPNSVVGRALVVHELEDDLGKGG
HELSLTTGNAGGRLACGVVGLTPI
>3AYQA
FAGGTVSQRCLSCICKMESGCRNVGCKMDMGSLSCGYFQIKEAYWIDCGRPGSSWKSCAASSYCA
SLCVQNYMKRYAKWAGCPLRCEGFAREHNGGPRGCKKGSTIGYWNRLQKISGCHGVQ
>3RZIA
MNWTVDIPIDQLPSLPPLPTDLRTRLDAALAKPAAQQPTWPADQALAMRTVLESVPPVTVPSEIV
RLQEQLAQVAKGEAFLLQGGDCAETFMDNTEPHIRGNVRALLQMAVVLTYGASMPVVKVARIAGQ
YAKPRSADIDALGLRSYRGDMINGFAPDAAAREHDPSRLVRAYANASAAMNLVRALTSSGLASLH
LVHDWNREFVRTSPAGARYEALATEIDRGLRFMSACGVADRNLQTAEIYASHEALVLDYERAMLR

LSDGDDGEPQLFDLSAHTVWIGERTRQIDGAHIAFAQVIANPVGVKLGPNMTPELAVEYVERLDP
HNKPGRLTLVSRMGNHKVRDLLPPIVEKVQATGHQVIWQCDPMHGNTHESSTGFKTRHFDRIVDE
VQGFFEVHRALGTHPGGIHVEITGENVTECLGGAQDISETDLAGRYETACDPRLNTQQSLELAFL
VAEMLRD
>3RQOA
RAPAPATPHAPDHSPAPNSPTLTRPPEGPKFPRVKNWELGSITYDTLCAQSQQDGPCTPRRCLGS
LVLPRKLQTRPSPGPPPAEQLLSQARDFINQYYSSIKRSGSQAHEERLQEVEAEVASTGTYHLRE
SELVFGAKQAWRNAPRCVGRIQWGKLQVFDARDCSSAQEMFTYICNHIKYATNRGNLRSAITVFP
QRAPGRGDFRIWNSQLVRYAGYRQQDGSVRGDPANVEITELCIQHGWTPGNGRFDVLPLLLQAPD
EAPELFVLPPELVLEVPLEHPTLEWFAALGLRWYALPAVSNMLLEIGGLEFSAAPFSGWYMSTEI
GTRNLCDPHRYNILEDVAVCMDLDTRTTSSLWKDKAAVEINLAVLHSFQLAKVTIVDHHAATVSF
MKHLDNEQKARGGCPADWAWIVPPISGSLTPVFHQEMVNYILSPAFRYQPDPW
>3RO9A
KVPVVGIVAALLPEMGIGFQGNLPWRLAKEMKYFREVTTLTNDNSKQNVVIMGRKTWESIPQKFR
PLPKRINVVVSRSFDGELRKVEDGIYHSNSLRNCLTALQSSLANENKIERIYIIGGGEIYRQSMD
LADHWLITKIMPLPETTIPQMDTFLQKQELEQRFYDNSDKLVDFLPSSIQLEGRLTSQEWNGELV
KGLPVQEKGYQFYFTLYTKKLEHHHHHHHH
>3RMJA
GIDPFTMTQTNRVIIFDTTLRDGEQSPGAAMTKEEKIRVARQLEKLGVDIIEAGFAAASPGDFEA
VNAIAKTITKSTVCSLSRAIERDIRQAGEAVAPAPKKRIHTFIATSPIHMEYKLKMKPKQVIEAA
VKAVKIAREYTDDVEFSCEDALRSEIDFLAEICGAVIEAGATTINIPDTVGYSIPYKTEEFFREL
IAKTPNGGKVVWSAHCHNDLGLAVANSLAALKGGARQVECTVNGLGERAGNASVEEIVMALKVRH
DLFGLETGIDTTQIVPSSKLVSTITGYPVQPNKAIVGANAFSHESGIHQDGVLKHRETYEIMSAE
SVGWATNRLSLGKLSGRNAFKTKLADLGIELESEEALNAAFARFK
>3AXXA
MEGNTILKIVLICTILAGLFGQVVPVYAENTTYQTPTGIYYEVRGDTIYMINVTSGEETPIHLFG
VNWFGFETPNHVVHGLWKRNWEDMLLQIKSLGFNAIRLPFCTESVKPGTQPIGIDYSKNPDLRGL
DSLQIMEKIIKKAGDLGIFVLLDYHRIGCTHIEPLWYTEDFSEEDFINTWIEVAKRFGKYWNVIG
ADLKNEPHSVTSPPAAYTDGTGATWGMGNPATDWNLAAERIGKAILKVAPHWLIFVEGTQFTNPK
TDSSYKWGYNAWWGGNLMAVKDYPVNLPRNKLVYSPHVYGPDVYNQPYFGPAKGFPDNLPDIWYH
HFGYVKLELGYSVVIGEFGGKYGHGGDPRDVIWQNKLVDWMIENKFCDFFYWSWNPDSGDTGGIL
QDDWTTIWEDKYNNLKRLMDSCSKSSSSTQSVIRSTTPTKSNTSKKICGPAILIIILAVFSLLLRR
APR
>3RKLA
MSKITINIKDNTIEYGHKEFVLSNLQEDIKNLAEIVYQLAKLIEKLSQYEEEVDTELYNLLHEYA
IYLAGATSMFIDSENKHHHHHH
>3RIKA
ARPCIPKSFGYSSVVCVCNATYCDSFDPPTFPALGTFSRYESTRSGRRMELSMGPIQANHTGTGL
LLTLQPEQKFQKVKGFGGAMTDAAALNILALSPPAQNLLLKSYFSEEGIGYNIIRVPMASCDFSI
RTYTYADTPDDFQLHNFSLPEEDTKLKIPLIHRALQLAQRPVSLLASPWTSPTWLKTNGAVNGKG
SLKGQPGDIYHQTWARYFVKFLDAYAEHKLQFWAVTAENEPSAGLLSGYPFQCLGFTPEHQRDFI
ARDLGPTLANSTHHNVRLLMLDDQRLLLPHWAKVVLTDPEAAKYVHGIAVHWYLDFLAPAKATLG
ETHRLFPNTMLFASEACVGSKFWEQSVRLGSWDRGMQYSHSIITNLLYHVVGWTDWNLALNPEGG
PNWVRNFVDSPIIVDITKDTFYKQPMFYHLGHFSKFIPEGSQRVGLVASQKNDLDAVALMHPDGS

AVVVVLNRSSKDVPLTIKDPAVGFLETISPGYSIHTYLWHRQ

>3RFYA

GSPGISGGGGGILLVANPVIPDVSVLISGPPIKDPEALLRYALPIDNKAIREVQKPLEDITDSLK
IAGVKALDSVERNVRQASRTLQQGKSIIVAGFAESKKDHGNEMIEKLEAGMQDMLKIVEDRKRDA
VAPKQKEILKYVGGIEEDMVDGFPYEVPEEYRNMPLLKGRASVDMKVKIKDNPNIEDCVFRIVLD
GYNAPVTAGNFVDLVERHFYDGMEIQRSDGFVVQTGDPEGPAEGFIDPSTEKTRTVPLEIMVTGE
KTPFYGSTLEELGLYKAQVVIPFNAFGTMAMAREEFENDSGSSQVFWLLKESELTPSNSNILDGR
YAVFGYVTDNEDFLADLKVGDVIESIQVVSGLENLANPSYKIAG

>2YFKA

METFKEYIEKLDKLEFEKMYENDFFLTWEKTRDELEAVFTVADTLRYLRENNISTKIFDSGLGIS
LFRDNSTRTRFSFASACNLLGLEVQDLDEGKSQISHGETVRETANMISFMADIIGIRDDMYIGKG
NAYMHEVSESVQEGYKDGVLEQRPTLVNLQCDIDHPTQAMADALHLIHEFGGIENLKGKKVAMTW
AYSPSYGKPLSVPQGIVGLMTRLGMDVVLAHPEGYEIMPEVEEVAKKNAAEFGGNFTKTNSMAEA
FKDADVVYPKSWAPFAAMEKRTELYGNGDQAGIDQLEQELLSQNKKHKDWECTEELMKTTKDGKA
LYMHCLPADITGVSCEEGEVEASVFDRYRVELYKEASYKPYVIAAMIFLSKVKNPQKTLTDLADK
ATPREVKDPNSSSVDKLAAALEHHHHHH

>2LBTA

GQAPPGPPASGPCADLQSAINAVTGGPIAFGNDGASLIPAAYEILNRVADKLKACPDARVTINGY
TDNTGSEGINIPLSAQRAKIVADYLVARGVAGDHIATVGLGSVNPIASNATPEGRAKNRRVEIVV
NHHHHHH

>3AXDA

MVSMKDFSGAELYTLEEYQYGKFEARMKMAAASGTVSSMFLYQNGSEIADGRPWVEVDIEVLGKS
PGSFQSNIITGKAGAQKTSEKHHAVSPAADQAFHTYGLEWTPNYVRWTVDGQEVRKTEGGQVSNL
TGTQGLRFNLWSSESAAWVGQFDESKLPLFQFINWVKVYKYTPGQGEGGSDFTLDWTDNFDTFDG
SRWGKGDYTFDGNRVDLTDKNIYSRDGMLILALTRKGQESFNGQVPRDDEPAPL

>2YEVB

MQRSFAALGLWGLSLAQEAHRVAITHPGGSFNQEVAFLFPWVYFFSFLIFLVVAGSLAYVTWKFR
ARPEDQEEPPQIHGNDRLEVVWTLIPLAIVFVLFGLTAKALIQVNRPIPGAMKVEVTGYQFWWDF
HYPELGLRNSNELVLPAGVPVELEITSKDVIHSFWVPGLAGKRDAIPGQTTRISFEPKEPGLYYG
FCAELCGASHARMLFRVVVLPKEEFDRFVEAAKASPAPVADERGQQVFQQNCAACHGVARSMPPA
VIGPELGLWGNRTSLGAGIVENTPENLKAWIRDPAGMKPGVKMPGFPQLSEEDLDALVRYLEGLK
VEGFDFGALPKF

>2YEVA

MAITAKPKAGVWAVLWDLLTTVDHKKIGLMYTATAFFAFALAGVFSLLIRTQLAVPNNQFLTGEQ
YNQILTLHGATMLFFFIIQAGLTGFGNFVVPLMLGARDVALPRVNAFSYWAFLGAIVLALMSYFF
PGGAPSVGWTFYYPFSAQSESGVDFYLAAILLLGFSSLLGNANFVATIYNLRAQGMSLWKMPIYV
WSVFAASVLNLFSLAGLTAATLLVLLERKIGLSWFNPAVGGDPVLFQQFFWFYSHPTVYVMLLPY
LGILAEVASTFARKPLFGYRQMVWAQMGIVVLGTMVWAHHMFTVGESTLFQIAFAFFTALIAVPT
GVKLFNIIGTLWGGKLQMKTPLYWVLGFIFNFLLGGITGVMLSMTPLDYQFHDSYFVVAHFHNVL
MAGSGFGAFAGLYYWWPKMTGRMYDERLGRLHFWLFLVGYLLTFLPQYALGYLGMPRRYYTYNAD
IAGWPELNLLSTIGAYILGLGGLVWIYTMWKSLRSGPKAPDNPWGGYTLEWLTASPPKAHNFDVK
LPTEFPSERPLYDWKKKGVELKPEDPAHIHLPNSSFWPFYSAATLFAFFVAVAALPVPNVWMWVF
LALFAYGLVRWALEDEYSHPVEHHTVTGKSNAWMGMAWFIVSEVGLFAILIAGYLYLRLSGAATP
PEERPALWLALLNTFLLVSSSFTVHFAHHDLRRGRFNPFRFGLLVTIILGVLFFLVQSWEFYQFY

HHSSWQENLWTAAFFTIVGLHGLHVVIGGFGLILAYLQALRGKITLHNHGTLEAASMYWHLVDAV
WLVIVTIFYVW
>3RBUA
RSGLNDIFEAQKIEWHEGSGSGSENLYFQGRSKSSNEATNITPKHNMKAFLDELKAENIKKFLYN
FTQIPHLAGTEQNFQLAKQIQSQWKEFGLDSVELAHYDVLLSYPNKTHPNYISIINEDGNEIFNT
SLFEPPPPGYENVSDIVPPFSAFSPQGMPEGDLVYVNYARTEDFFKLERDMKINCSGKIVIARYG
KVFRGNKVKNAQLAGAKGVILYSDPADYFAPGVKSYPDGWNLPGGGVQRGNILNLNGAGDPLTPG
YPANEYAYRRGIAEAVGLPSIPVHPIGYYDAQKLLEKMGGSAPPDSSWRGSLKVPYNVGPGFTGN
FSTQKVKMHIHSTNEVTRIYNVIGTLRGAVEPDRYVILGGHRDSWVFGGIDPQSGAAVVHEIVRS
FGTLKKEGWRPRRTILFASWDAEEFGLLGSTEWAEENSRLLQERGVAYINADSSIEGNYTLRVDC
TPLMYSLVHNLTKELKSPDEGFEGKSLYESWTKKSPSPEFSGMPRISKLGSGNDFEVFFQRLGIA
SGRARYTKNWETNKFSGYPLYHSVYETYELVEKFYDPMFKYHLTVAQVRGGMVFELANSIVLPFD
CRDYAVVLRKYADKIYSISMKHPQEMKTYSVSFDSLFSAVKNFTEIASKFSERLQDFDKSNPIVL
RMMNDQLMFLERAFIDPLGLPDRPFYRHVIYAPSSHNKYAGESFPGIYDALFDIESKVDPSKAWG
EVKRQIYVAAFTVQAAAETLSEVA
>2YDQA
GSVGPKTGEENQVLVPNLNPTPENLEVVGDGFKITSSINLVGEEEADENAVNALREFLTANNIEI
NSENDPNSTTLIIGEVDDDIPELDEALNGTTAENLKEEGYALVSNDGKIAIEGKDGDGTFYGVQT
FKQLVKESNIPEVNITDYPTVSARGIVEGFYGTPWTHQDRLDQIKFYGENKLNTYIYAPKDDPYH
REKWREPYPESEMQRMQELINASAENKVDFVFGISPGIDIRFDGDAGEEDFNHLITKAESLYDMG
VRSFAIYWDNIQDKSAAKHAQVLNRFNEEFVKAKGDVKPLITVPTEYDTGAMVSNGQPRAYTRIF
AETVDPSIEVMWTGPGVVTNEIPLSDAQLISGIYDRNMAVWWNYPVTDYFKGKLALGPMHGLDKG
LNQYVDFFTVNPMEHAELSKISIHTAADYSWNMDNYDYDKAWNRAIDMLYGDLAEDMKVFANHST
RMDNKTWAKSGREDAPELRAKMDELWNKLSSKEDASALIEELYGEFARMEEACNNLKANLPEVAL
EECSRQLDELITLAQGDKASLDMIVAQLNEDTEAYESAKEIAQNKLNTALSSFAVISEKVAQSFI
QEALS
>3R6TA
MGDTKEQRILRYVQQNAKPGDPQSVLEAIDTYCTQKEWAMNVGDAKGQIMDAVIREYSPSLVLEL
GAYCGYSAVRMARLLQPGARLLTMEINPDCAAITQQMLNFAGLQDKVTILNGASQDLIPQLKKKY
DVDTLDMVFLDHWKDRYLPDTLLLEKCGLLRKGTVLLADNVIVPGTPDFLAYVRGSSSFECTHYS
SYLEYMKVVDGLEKAIYQGPSSPDKS
>3R3JA
LHNYGYTSTKSVDNQIEELREKVVSKNKNEPEFLQAFEEVLSCLKPVFKKDNVYIGVLENIAEPE
RVIQFRVPWINDKGEHKMNRGFRVQYNSVLGPYKGGLRFHPAVNLSVIKFLGFEQIFKNSLTTLP
MGGGKGGSDFDPKGKSENEILKFCQSFMTNLFRYIGPNTDVPAGDIGVGGREIGYLFGQYKKLKN
SFEGVLTGKNIKWGGSNIRAEATGYGVVYFAENVLKDLNDNLENKKCLVSGSGNVAQYLVEKLIE
KGAIVLTMSDSNGYILEPNGFTKEQLNYIMDIKNNQRLRLKEYLKYSKTAKYFENQKPWNIPCDI
AFPCATQNEINENDADLFIQNKCKMIVEGANMPTHIKALHKLKQNNIILCPSKAANAGGVAVSGL
EMSQNSMRLQWTHQETDMKLQNIMKSIYEQCHNTSKIYLNESDLVAGANIAGFLKVADSFLEQGG
L
>3R18A
APSYPEYTREEVGRHRSPEERVWVTHGTDVFDVTDFVELHPGGPDKILLAAGGALEPFWALYAVH
GEPHVLELLQQYKVGELSPDEAPAAPDAQDPFAGDPPRHPGLRVNSQKPFNAEPPAELLAERFLT
PNELFFTRNHLPVPAVEPSSYRLRVDGPGGGTLSLSLAELRSRFPKHEVTATLQCAGNRRSEMSR

VRPVKGLPWDIGAISTARWGGARLRDVLLHAGFPEELQGEWHVCFEGLDADPGGAPYGASIPYGR
ALSPAADVLLAYEMNGTELPRDHGFPVRVVVPGVVGARSVKWLRRVAVSPDESPSHWQQNDNKGF
SPCVDWDTVDYRTAPAIQELPVQSAVTQPRPGAAVPPGELTVKGYAWSGGGREVVRVDVSLDGGR
TWKVARLMGDKAPPGRAWAWALWELTVPVEAGTELEIVCKAVDSSYNVQPDSVAPIWNLMGVLST
AWHRVRVSVQD
>3QVSA
MKVWLVGAYGIVSTTAMVGARAIERGIAPKIGLVSELPHFEGIEKYAPFSFEFGGHEIRLLSNAY
EAAKEHWELNRHFDREILEAVKSDLEGIVARKGTALNCGSGIKELGDIKTLEGEGLSLAEMVSRI
EEDIKSFADDETVVINVASTEPLPNYSEEYHGSLEGFERMIDEDRKEYASASMLYAYAALKLGLP
YANFTPSPGSAIPALKELAEKKGVPHAGNDGKTGETLVKTTLAPMFAYRNMEVVGWMSYNILGDY
DGKVLSARDNKESKVLSKDKVLEKMLGYSPYSITEIQYFPSLVDNKTAFDFVHFKGFLGKLMKFY
FIWDAIDAIVAAPLILDIARFLLFAKKKGVKGVVKEMAFFFKSPMDTNVINTHEQFVVLKEWYSN
LK
>3QSYA
MAWPKVQPEVNIGVVGHVDHGKTTLVQAITGIWTSKHSEELKRGMTIKLGYAETNIGVCESCKKP
EAYVTEPSCKSCGSDDEPKFLRRISFIDAPGHEVLMATMLSGAALMDGAILVVAANEPFPQPQTR
EHFVALGIIGVKNLIIVQNKVDVVSKEEALSQYRQIKQFTKGTWAENVPIIPVSALHKINIDSLI
EGIEEYIKTPYRDLSQKPVMLVIRSFDVNKPGTQFNELKGGVIGGSIIQGLFKVDQEIKVLPGLR
VEKQGKVSYEPIFTKISSIRFGDEEFKEAKPGGLVAIGTYLDPSLTKADNLLGSIITLADAEVPV
LWNIRIKYNLLERVVGAKEMLKVDPIRAKETLMLSVGSSTTLGIVTSVKKDEIEVELRRPVAVWS
NNIRTVISRQIAGRWRMIGWGLVEI
>3AUKA
MAASRANDAPIVLLHGFTGWGREEMFGFKYWGGVRGDIEQWLNDNGYRTYTLAVGPLSSNWDRAC
EAYAQLVGGTVDYGAAHAAKHGHARFGRTYLGLLPELKRGGRIHIIAHSQGGQTARMLVSLLENG
SQEEREYAKAHNVSLSPLFEGGHHFVLSVTTIATPHDGTTLVNMVDFTDRFFDLQKAVLEAAAVA
SNVPYTSQVYDFKLDQWGLRRQPGESFDHYFERLKRSPVWTSTDTARYDLSVSGAEKLNQWVQAS
PNTYYLSFATERTYRGALTGNYYPELGMNAFSAVVCAPFLGSYRNPTLGIDDRWLENDGIVNTVS
MNGPKRGSSDRIVPYDGALKKGVWNDMGTYNVDHLEIIGVDPNPSFDIRAFYLRLAEQLASLQP
>3QMXA
MRGSHHHHHHGSAVSAKIEIYTWSTCPFCMRALALLKRKGVEFQEYCIDGDNEAREAMAARANGK
RSLPQIFIDDQHIGGCDDIYALDGAGKLDPLLHS
>3QLIA
HHHHHHSSGLVPRGSMDIRALYDEKLTTPEEAVSSIASGSHLSMGMFAAEPPALLKALADRATRG
DIGDLRVYYFETAKIAGDTILRYELNNRIKPYSMFVTAVERALIRRGIEDGGRKVVNYVPSNFHQ
APRLLAEEIGIDTFMHTVSPMDCHGYFSLGVGNDYSSRIARSARRFIVEVNRYMPRVQGEAAAIH
ISEVDAIVENHVPLIEMPVRSAIPEYTSISHIIADLVPDGACLQMGVGALPNLVCGVLKDRNDLG
IHTEVLNPGLVDLIRRGVVTNQRKTLDRGRSVFTFAMGQQEMYEYLNDHPAIFSRPVDYVNDPHI
IAQNDNVVSINATLQIDLTGACNSEHMLGHQYSASGGQLDFVRGAYASKGGRSIIATPSTAAKGT
VSRIIPRIDGPVTTPRIDTHYIVTEFGAVNLKGLSSTERALRIIELAHPDFRDELTQAAKKMHLI
>2L97A
MEGLGFAIPANDAINIIEQLEKNGKVTRPALGIQMVNLSNVSTSDIRRLNIPSNVTSGVIVRSVQ
SNMPANGHLEKYDVITKVDDKEIASSTDLQSALYNHSIGDTIKITYYRNGKEETTSIKLNKLEHH
HHHH
>3QKGA

GPVPTPPDNIQVQENFNISRIYGKWYNLAIGSTSPWLKKIMDRMTVSTLVLGEGATEAEISMTST
RWRKGVCEETSGAYEKTDTDGKFLYHKSKWNITMESYVVHTNYDEYAIFLTKKFSRHHGPTITAK
LYGRAPQLRETLLQDFRVVAQGVGIPEDSIFTMADRGECVPGEQEPEPILIPRSAWSHPQFEK
>3QJJA
HHHHMRIEVKLLPLKDNPILPFNYNYEVYSQILEKVNSIEPTIAKLLSSPHGFWTFSRIIVRKRK
ILPDKGIEILSDDVSLYISSSNEDIIRAIAEAVEKSPEFKIGELSFLVGDIKAIKVKELGKENVF
STLSPIVVRTVKFEGNKLRHWDLYPHDELFMDRLRKVMILRYSEVMGETPKDRDFTIEVLKFKPT
RLMVGSSYIRGSLMVFRYAGSEEIARFGYENGFGEKTGLGFGMVKLIE
>2Y6YA
GAMTIGRAKVYATLSKIFYHLFYDEAIPKDCREIIEKFGEIDFNLRSVLVRELRGSVLIKDMPQS
LAEVYESVMKDFYERYGFQASELHADHIAVELAFMSKLVEREISLAQQMKEEELYKIRAAQHRFI
KAHLQPLVKNLPSAPLLNFVRDFVREDAKYLYSSLVGEKNEGADNN
>2L8LA
CLAEGTRIFDPVTGTTHRIEDVVDGRKPIHVVAAAKDGTLHARPVVSWFDQGTRDVIGLRIAGGA
ILWATPDHKVLTEYGWRAAGELRKGDRVAVRDVETGELRYSVIREVLPTRRARTFDLEVEELHTL
VAEGVVVHN
>3QDDA
HMPEETQTQDQPMEEEEVETFAFQAEIAQLMSLIINTFYSNKEIFLRELISNSSDALDKIRYESL
TDPSKLDSGKELHINLIPNKQDRTLTIVDTGIGMTKADLINNLGTIAKSGTKAFMEALQAGADIS
MIGQFGVGFYSAYLVAEKVTVITKHNDDEQYAWESSAGGSFTVRTDTGEPMGRGTKVILHLKEDQ
TEYLEERRIKEIVKKHSQFIGYPITLFVEKERDKEVSDDEAE
>3QBDA
HHHHHHMTDIGAPVTVQVAVDPPYPVVIGTGLLDELEDLLADRHKVAVVHQPGLAETAEEIRKRL
AGKGVDAHRIEIPDAEAGKDLPVVGFIWEVLGRIGIGRKDALVSLGGGAATDVAGFAAATWLRGV
SIVHLPTTLLGMVDAAVGGKTGINTDAGKNLVGAFHQPLAVLVDLATLQTLPRDEMICGMAEVVK
AGFIADPVILDLIEADPQAALDPAGDVLPELIRRAITVKAEVVAADEKESELREILNYGHTLGHA
IERRERYRWRHGAAVSVGLVFAAELARLAGRLDDATAQRHRTILSSLGLPVSYDPDALPQLLEIM
AGDKKTRAGVLRFVVLDGLAKPGRMVGPDPGLLVTAYAGVCAP
>3QB4A
MKRQGKRPSKNLKARCSRKALHVNFKDMGWDDWIIAPLEYEAFHCEGLCEFPLASHLEPTNHAVI
QTLMNSMDPESTPPTCCVPTRLSPISILFIDSANNVVYKQYEDMVVESCGCR
>2L8BA
TSGIHVLDELSVRALSRDIMKQNRVTVHPEKSVPRTAGYSDAVSVLAQDRPSLAIVSGQGGAAGQ
RERVAELVMMAREQGREVQIIADRRSQMNMKQDERLSGELITGRRQLLEGMAFTPGSTVIVDQG
EKLSLKETLTLLDGAARHNVQVLITDSGQRTGTGSALMAMKDAGVNTYRWQGGEQRPAT
